# Isolation and Identification of 12-Deoxyphorbol Esters from *Euphorbia resinifera* Berg Latex: Targeted and Biased Non-Targeted Identification of 12-Deoxyphorbol Esters by UHPLC-HRMS^E^

**DOI:** 10.3390/plants12223846

**Published:** 2023-11-14

**Authors:** Abdellah Ezzanad, Carolina De los Reyes, Antonio J. Macías-Sánchez, Rosario Hernández-Galán

**Affiliations:** 1 Departamento de Química Orgánica, Facultad de Ciencias, Campus Universitario Puerto Real, Universidad de Cádiz, Puerto Real, 11510 Cádiz, Spain; abdellah.ezzanad@gm.uca.es (A.E.); carolina.dereyes@uca.es (C.D.l.R.); 2 Instituto de Investigación en Biomoléculas (INBIO), Universidad de Cádiz, Puerto Real, 11510 Cádiz, Spain

**Keywords:** Euphorbiaceae, *Euphorbia resinifera* Berg, diterpenes, 12-deoxyphorbols, absolute configuration, UHPLC, HRMS^E^

## Abstract

Diterpenes from the *Euphorbia* genus are known for their ability to regulate the protein kinase C (PKC) family, which mediates their ability to promote the proliferation of neural precursor cells (NPCs) or neuroblast differentiation into neurons. In this work, we describe the isolation from *E. resinifera* Berg latex of fifteen 12-deoxyphorbol esters (**1**–**15**). A triester of 12-deoxy-16-hydroxyphorbol (**4**) and a 12-deoxyphorbol 13,20-diester (**13**) are described here for the first time. Additionally, detailed structural elucidation is provided for compounds **3**, **5**, **6**, **14** and **15**. The absolute configuration for compounds **3**, **4**, **6**, **13**, **14** and **15** was established by the comparison of their theoretical and experimental electronic circular dichroism (ECD) spectra. Access to the above-described collection of 12-deoxyphorbol derivatives, with several substitution patterns and attached acyl moieties, allowed for the study of their fragmentation patterns in the collision-induced dissociation of multiple ions, without precursor ion isolation mass spectra experiments (HRMS^E^), which, in turn, revealed a correlation between specific substitution patterns and the fragmentation pathways in their HRMS^E^ spectra. In turn, this allowed for a targeted UHPLC-HRMS^E^ analysis and a biased non-targeted UHPLC-HRMS^E^ analysis of 12-deoxyphorbols in *E. resinifera* latex which yielded the detection and identification of four additional 12-deoxyphorbols not previously isolated in the initial column fractionation work. One of them, identified as 12-deoxy-16-hydroxyphorbol 20-acetate 13-phenylacetate 16-propionate (**20**), has not been described before.

## 1. Introduction

*Euphorbia* is the largest genus in the Euphorbiaceae family, and it is well known for the extraordinary chemical diversity and intriguing biological activities of its constituents [1,2,3]. Diterpenes occurring in plants of this genus exhibit relevant activities such as antitumor, vasorelaxant, anti-multidrug resistance, antiviral, anti-inflammatory, cytotoxicity, cocarcinogenicity and skin-irritating effects [1,2,3,4,5,6,7]. One of the cellular targets of Euphorbiaceae diterpenes that mediates their bioactivities is protein kinase C (PKC). In previous works, we have shown that 12-deoxyphorbol and ingol esters, isolated from the latex of *Euphorbia resinifera* Berg, promote the proliferation of neural precursor cells (NPCs) or neuroblast differentiation into neurons by targeting and activating one or more PKC isozymes [8,9,10,11].

The fresh latex of cultivated *E. resinifera* Berg. (Euphorbium) is a convenient source of 12-deoxyphorbol ester and ingol-type diterpenoids. However, they occur at very low concentration levels [12,13]. Their isolation from complex mixtures using conventional column chromatography and semi-preparative or preparative high-resolution liquid chromatography (HPLC) is time-consuming and laborious [1,4,12,13]. Therefore, there is an interest in the development of methods for the identification of 12-deoxyphorbol esters, within complex mixtures, which could assist in their guided isolation.

HPLC coupled with tandem mass spectrometry (HPLC-MS/MS) is a very powerful tool for identifying relevant components within complex matrices [14,15]. Many classes of natural products, such as flavonoids, terpenoids, phenolic acids and saponins, have been identified and characterized using HPLC-MS/MS based on fragmentation patterns [16,17,18]. More specifically, several HPLC-MS/MS-based methods targeting isolated or commercial compounds have been developed in order to monitor the diterpene esters of tigliane [19,20], ingenane [21,22] and dafnane structural classes [23,24,25]. In a previous study [8], we identified and isolated two new 12-deoxy-16-hydroxyphorbol 13,16-diesters from *E. resinifera* latex using one-step ultra-high-performance liquid chromatography coupled with high-resolution mass spectrometry with the collision-induced dissociation of multiple ions, without precursor ion isolation (MS^E^, data-independent acquisition (DIA); HRMS^E^ in this manuscript) [26] assisted screening (UHPLC-HRMS^E^), where differences in the substitution patterns on the tigliane skeleton could be correlated with specific fragmentation patterns in the collision-induced dissociation of multiple ions, without precursor ion isolation mass spectra (HRMS^E^).

In this work, we describe a detailed examination of *E. resinifera* Berg latex, which has led to the isolation of fifteen 12-deoxyphorbol esters, including 16-hydroxyphorbol derivatives and a 12,20-dideoxy derivative. A 12-deoxyphorbol diester and a triester of 12-deoxy-16-hydroxyphorbol are described here for the first time. Additionally, detailed structural elucidation is provided for five tigliane derivatives which have been previously reported as components of *E. resinifera* latex, but with little spectroscopic and spectrometric support. For selected isolated compounds, absolute configuration has been established by the comparison of their theoretical and experimental electronic circular dichroism (ECD) spectra. Access to the above-described collection of 12-deoxyphorbol derivatives, with several substitution patterns and attached acyl moieties, allowed for the study of their fragmentation patterns in HRMS^E^ experiments, which, in turn, revealed a correlation between specific substitution patterns (sub-structural classes) in 12-deoxyphorbol esters and the fragmentation pathways in their HRMS^E^ spectra. In turn, this allowed for a targeted and a biased non-targeted UHPLC-HRMS^E^ analysis of 12-deoxyphorbols in *E. resinifera* latex. As a result, the biased non-targeted analysis yielded the detection and identification of four additional 12-deoxyphorbols not previously isolated in the initial column fractionation work.

## 2. Results and Discussion

### 2.1. Structural Elucidation of Isolated Diterpenes

**Figure 1:**
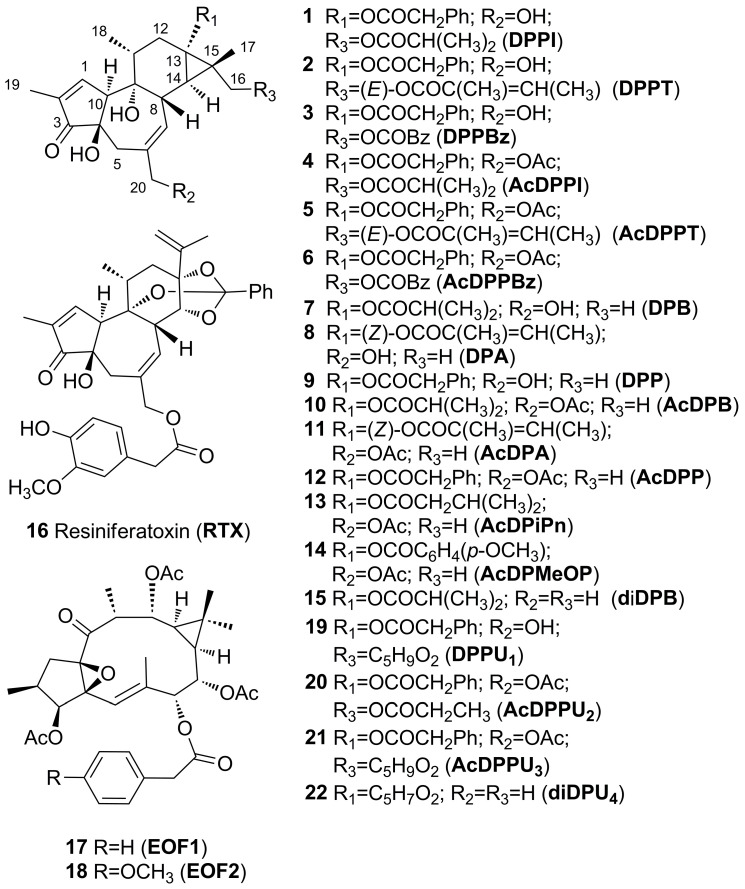
12-Deoxyphorbol esters (**1**–**15**) and other diterpenes (**16**–**18**) isolated from *E. resinifera.* 12-Deoxyphorbol esters (**19**–**22**) detected and identified from a biased untargeted UHPLC-HRMS^E^ analysis of *E. resinifera* latex.

The maceration in ethyl acetate (EtOAc) of *E. resinifera* Berg dried latex, evaporation of solvent from filtrate and trituration of the resulting solid residue with CH_3_CN yielded an additional clear solution where most triterpenes have been removed. The evaporation of solvent (CH_3_CN), column chromatography of the resulting crude mixture with increasing gradients of ethyl acetate in hexane and further purification of column chromatography fractions yielded compounds **1**–**18** (Figure 1).

The comparison of their spectroscopic and spectrometric data with those reported in the literature allowed for the identification of two known tetracyclic esters of 12-deoxy-16-hydroxyphorbol, 12-deoxy-16-hydroxyphorbol 16-isobutyrate-13-phenylacetate (DPPI (**1**)) and 12-deoxy-16-hydroxy-phorbol 16-tigliate-13-phenylacetate (DPPT (**2**)) [8].

**Figure 2:**
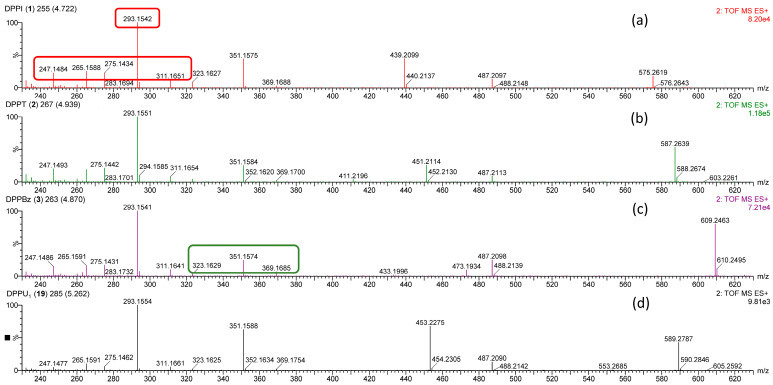
Comparison of HRMS^E^ spectra (DIA) [26] for (**a**) DPPI (**1**), (**b**) DPPT (**2**), (**c**) DPPBz (**3**) and (**d**) DPPU_1_ (**19**) (data acquired in positive ionization with a ramp trap collision energy of the high-energy function set at 60–120 eV). See proposed fragmentation route for selected ions in Scheme 1, together with color key.

**Scheme 1:**
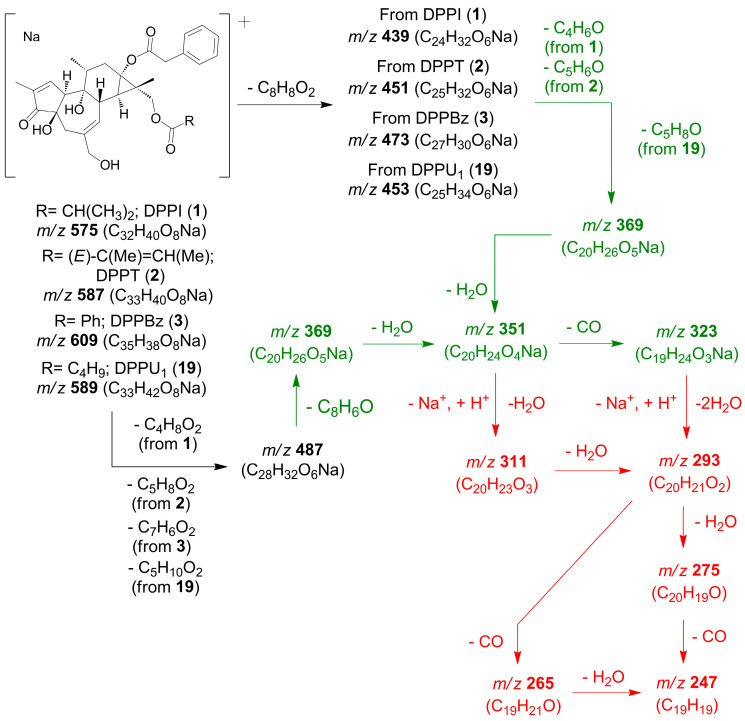
Proposed fragmentation route for selected ions on HRMS^E^ spectra (DIA, high-energy function) for 12-deoxy-16-hydroxyphorbol 13,16-diacyl derivatives (**1**–**3**, **19**, group A compounds; see Section 2.2). For each ion, nominal mass and elemental composition is presented; see a more detailed interpretation of the fragmentation route in Figure S20. In red, common daughter ions with group B compounds (see Section 2.2 and Scheme 2). In green, highlighted ions in Figure 2c.

On the other hand, compound **3** was isolated as an amorphous solid and presented a [M + Na]^+^ molecular ion in its HRMS^E^ spectrum at *m*/*z* 609.2463 (calcd for C_35_H_38_O_8_Na, 609.2464) (Table 1, Figure 2c), which provides a molecular formula C_35_H_38_O_8_. Daughter ions were observed in its HRMS^E^ spectrum at *m*/*z* 323.1629, 311.1641, 293.1541, 275.1431, 265.1591 and 247.1486, (Table 1, Figure 2c), which were also observed in similar mass spectra for DPPI (**1**) and DPPT (**2**) (Table S1, Figure 2a,b), and which are deemed characteristic of 13,16-diesters of 16-hydroxy-12-deoxyphorbol [8]. These ions could be assigned to losses of CO, water (1, 2 and 3 molecules), 2 molecules of water and CO and, finally, 3 molecules of water and CO, from a precursor ion at *m*/*z* 351.1572 (calculated), in turn, originated from the loss of ester groups at C-13 and C-16 from the parent molecular ion. The proposed fragmentation pathways leading to the above-mentioned ions can be found in Scheme 1 and Figure S20 for DPPI (**1**), DPPT (**2**) and compound **3**.

**Figure 3:**
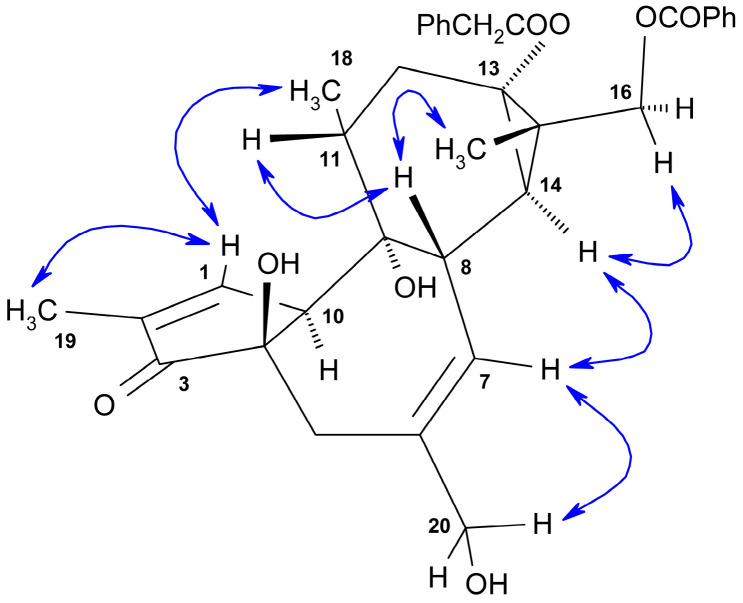
Selected NOESY correlations (blue arrows) for DPPBz (**3**).

Furthermore, compound **3** showed similar ^1^H and ^13^C NMR data to those of DPPI (**1**) and DPPT (**2**) but displayed the presence of signals characteristic of a benzoate ester group (Table 2 and Table 3), whose carbonyl group was correlated in the HMBC experiment with the H_2_-16 signals (Figure S3g,h). These data, together with previously discussed HRMS^E^ data and the observation of an ion at *m*/*z* 473.1934 (calcd 473.1940, C_27_H_30_O_6_Na), consistent with a loss of phenylacetic acid from the parent molecular ion (Table 1, Figure 2c), pointed out that a structure of 12-deoxy-16-hydroxyphorbol 16-benzoate 13-phenylacetate (DPPBz) could be assigned for compound **3**. On the one hand, NOESY correlations observed between H_2_-16 and H-14α located a benzoate group on C-16; on the other hand, NOESY correlations observed between H-8β, H-11β and H_3_-17 (Figure 3) confirmed the location of a phenylacetate group at C-13 and suggested that the relative configuration for DPPBz (**3**) was identical to the one previously observed for **1** (DPPI) and **2** (DPPT).

Compound **3** (DPPBz) was previously identified by Hergenhahn et al. as a component of *E. resinifera* latex (RL22) [27], but no spectroscopic or spectrometric data supporting this assignment could be found in the literature.

**Scheme 2:**
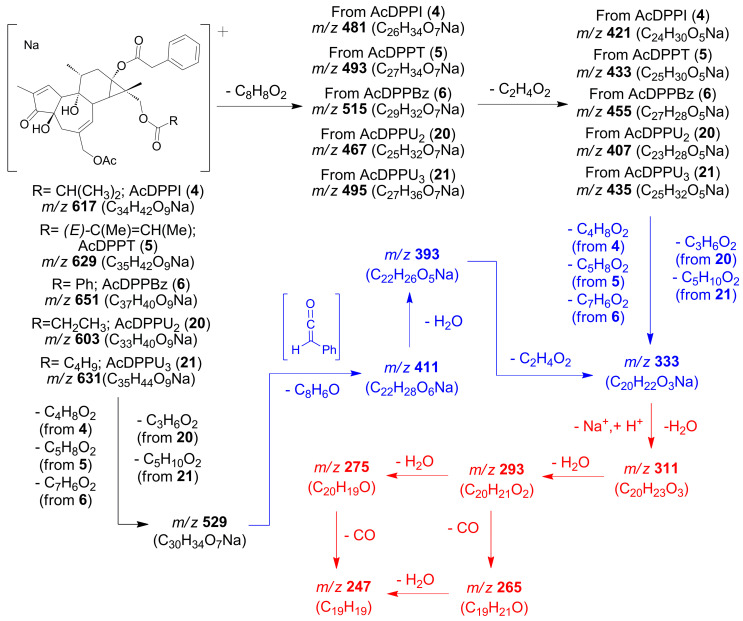
Proposed fragmentation route for selected ions on HRMS^E^ spectra (DIA, high-energy function) for 16-hydroxy-12-deoxyphorbol 20-acetate-13,16-diacyl derivatives (**4**–**6**, **20**–**21**, group B compounds; see Section 2.2). For each ion, nominal mass and elemental composition is presented; see a more detailed interpretation of the fragmentation route in Figure S22. In red, common daughter ions with group A compounds (see Section 2.2 and Scheme 1). In blue, highlighted ions in Figure 4a.

**Figure 4:**
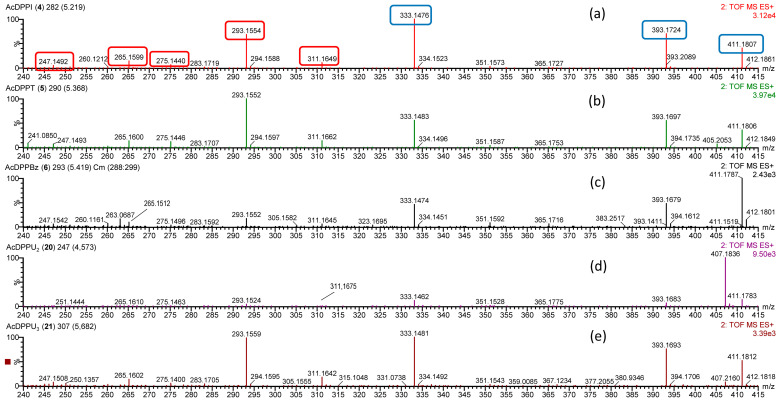
Comparison of HRMS^E^ spectra (DIA) [26] for (**a**) AcDPPI (**4**), (**b**) AcDPPT (**5**), (**c**) AcDPPBz (**6**), (**d**) AcDPPU_2_ (**20**) and (**e**) AcDPPU_3_ (**21**), *m*/*z* range 240–415 (*m*/*z* range 240–680 in Figure S21) (data acquired in positive ionization with a ramp trap collision energy of the high-energy function set at 60–120 eV). See proposed fragmentation route for selected ions in Scheme 2, together with color key.

Compounds **4**, **5** and **6** were isolated as amorphous solids and presented [M + Na]^+^ molecular ions in their HRMS^E^ spectra at *m*/*z* 617.2755 (calcd for C_34_H_42_O_9_Na, 617.2727), *m*/*z* 629.2745 (calcd for C_35_H_42_O_9_Na, 629.2727) and at *m*/*z* 651.2601 (calcd for C_37_H_40_O_9_Na, 651.2570) (Table 4, Figure S21b–d), which provides the molecular formulas C_34_H_42_O_9_ for compound **4**, C_35_H_42_O_9_ for compound **5** and C_37_H_40_O_9_ for compound **6**, respectively. Daughter ions were observed in their HRMS^E^ spectra at *m*/*z* 311.1647, 293.1542 and 275.1436 (calculated) (Table 4, Figure 4a–c), which were similar to the ones described above for 13,16 diesters of 16-hydroxy-12-deoxyphorbols (DPPI (**1**), DPPT (**2**) and DPPBz (**3**) (see, for instance, comparison between Figure 2b (DPPT (**2**)) and Figure 4a,b). On the other hand, daughter ions at *m*/*z* 411.1784, 393.1678 and 333.1467 (calculated) (Table 4, Figure 4a–c; highlighted in blue in Figure 4a) were also observed, which were not apparent in the HRMS^E^ spectra of compounds **1**, **2** or **3**. The latter group of ions could be assigned to losses of phenylketene, water and acetic acid from a precursor ion at *m*/*z* 529.2202 (calculated), which, in turn, originates from the loss of an ester group at C-16 from the parent molecular ions of compounds **4**, **5** and **6** (Table 4, Figure 4a–c and Figure S21b–d). The proposed fragmentation pathways leading to the above-mentioned ions can be found in Scheme 2 and Figure S22 for AcDPPI (**4**), AcDPPT (**5**) and Ac DPPBz (**6**).

The ^1^H and ^13^C NMR data of compounds **4**, **5** and **6** were very similar with those of DPPI (**1**), DPPT (**2**) and DPPBz (**3**), respectively, except for the presence of signals corresponding to an extra acetate group in each compound (Table 2 and Table 3). This is consistent with the analysis of the HRMS^E^ data discussed above, where ion *m*/*z* 333.1467 (calculated) could be understood to originate from a loss of acetic acid from the precursor at *m*/*z* 393.1678 (calculated) in compounds **4**, **5** and **6** (Scheme 2, Figure S22). Daughter ions at *m*/*z* 481.2222 (calcd for C_26_H_34_O_7_Na, 481.2202) for compound **4**, *m*/*z* 493.2221 (calcd for C_27_H_34_O_7_Na, 493.2202) for compound **5** and *m*/*z* 515.2083 (calcd for C_29_H_32_O_7_Na, 515.2046) for compound **6** were consistent with a loss of phenyl acetic acid from a molecular ion in each compound (Table 4, Figure S21; see Scheme 2 and Figure S22 for proposed fragmentation pathways), which, in turn, is consistent with the observation of the ^1^H and ^13^C NMR signals corresponding to a phenylacetate group (Table 2 and Table 3). Differences in ^1^H and ^13^C NMR for the above-mentioned compounds can be attributed to the presence of isobutyrate, tigliate and benzoate groups (Table 2 and Table 3). Further support for these observations can be drawn from the presence of an ion at *m*/*z* 529.2202 (calculated) in the HRMS^E^ spectra of compounds **4**, **5** and **6**, which would be consistent with losses of isobutyric, tiglic and benzoic acids from the molecular ions of previously mentioned compounds, respectively (Table 4, Figure S21; see Scheme 2 and Figure S22 for proposed fragmentation pathways). The HMBC correlations between H_2_-16 and C-1″ in compounds **4**, **5** and **6** located the isobutyrate/tigliate/benzoate groups at C-16 in each compound. The HMBC correlations between H_2_-20 and the carbonyl group of the acetate moiety in each compound located this group at C-20 (Figures S4g–S6g and S4i–S6i). Therefore, the phenylacetate moieties were located at C-13 in compounds **4**, **5** and **6**. NOESY correlations observed between H_2_-16 and H-14α, and between H-8β, H-11β and H_3_-17, analogous to the ones observed for DPPI (**1**), DPPT (**2**) [8] and DPPBz (**3**) (Figure 3), confirmed the location of isobutyrate/tigliate/benzoate moieties and supported the structural assignment for each compound as 12-deoxy-16-hydroxyphorbol 20-acetate 16-isobutyrate 13-phenylacetate (AcDPPI (**4**)), 12-deoxy-16-hydroxyphorbol 20-acetate 16-tigliate 13-phenylacetate (AcDPPT (**5**)) and 12-deoxy-16-hydroxyphorbol 20-acetate 16-benzoate 13-phenylacetate (AcDPPBz (**6**)).

**Figure 5:**
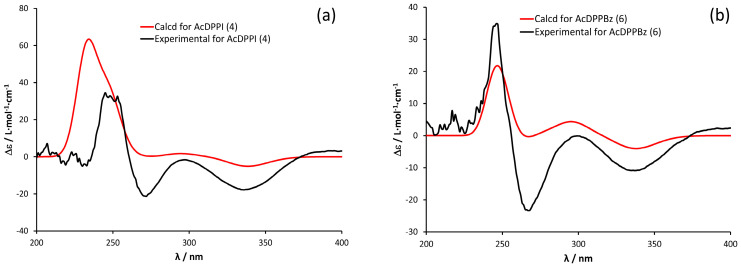
Experimental and calculated ECD spectra of (**a**) 12-deoxy-16-hydroxyphorbol 20-acetate-16-isobutyrate-13-phenylacetate (AcDPPI (**4**)); (**b**) 12-deoxy-16-hydroxyphorbol 20-acetate-13-phenylacetate-16-tigliate (AcDPPBz (**6**)).

The absolute configuration for compounds AcDPPI (**4**) and AcDPPBz (**6**) was determined by the comparison of their experimental electronic circular dichroism (ECD) spectra, with the computed ECD spectrum for each one of their 4*R*,8*S*,9*R*,10*S*,11*R*,13*S*,14*R*,15*S* stereoisomers, calculated from quantum mechanical time-dependent density functional theory (TDDFT) calculations with a 6–31 + G(d,p) level of theory, using the Gaussian 16 program [28]. As illustrated in Figure 5, the calculated and theoretical ECD curves matched well, leading to the assignment of the structure and absolute configuration of compounds **4** and **6**, respectively, as (4*R*,8*S*,9*R*,10*S*,11*R*,13*S*,14*R*,15*S*)-12-deoxy-16-hydroxyphorbol 20-acetate 16-isobutyrate 13-phenylacetate (AcDPPI) and (4*R*,8*S*,9*R*,10*S*,11*R*,13*S*,14*R*,15*S*)-12-deoxy-16-hydroxyphorbol 20-acetate 16-benzoate 13-phenylacetate (AcDPPBz). On the other hand, a comparison of the experimental ECD curves for compounds DPPI (**1**), DPPT (**2**), DPPBz (**3**), AcDPPT (**5**) and AcDPPBz (**6**) show similar magnitudes and signs of Cotton effects (Figure S19), which, in turn, allow for the assignation of the absolute configuration for compounds **1**, **2**, **3**, **5** and **6** as 4*R*,8*S*,9*R*,10*S*,11*R*,13*S*,14*R*,15*S* as well. The observed absolute configuration for compounds **1**–**6** matches what has been described previously for other tigliane derivatives [29].

As far as we know, compound **4** (AcDPPI) is described here for the first time. Compounds named as RL12 and RL11, which were attributed structures as the ones described here as AcDPPT (**5**) and AcDPPBz (**6**), respectively, have been reported previously [27,30], but with lesser spectroscopic and spectrometric support for their structural assignment.

The additional comparison of the spectroscopic and spectrometric data of the compounds isolated and not discussed above, with those reported in the literature, allowed for the identification of three known tetracyclic 13-esters of 12-deoxyphorbol, assigned as 12-deoxyphorbol 13-isobutyrate (DPB (**7**)) [31,32], 12-deoxyphorbol 13-angelate (DPA (**8**)) [33] and 12-deoxyphorbol 13-phenylacetate (DPP (**9**)) [33]. The examination of their HRMS^E^ spectra showed different fragmentation patterns to those described above for 16-hydroxy-12-deoxyphorbol 13,16-diacyl derivatives DPPI (**1**), DPPT (**2**) and DPPBz (**3**) (Table S1 and Table 1 and Figure 2). Selected ions of high-energy HRMS^E^ (DIA) [26] for DPB (**7**), DPA (**8**) and DPP (**9**), together with a comparison of their HRMS^E^ spectra, can be found in Table S2 and Figures S23 and S24.

**Scheme 3:**
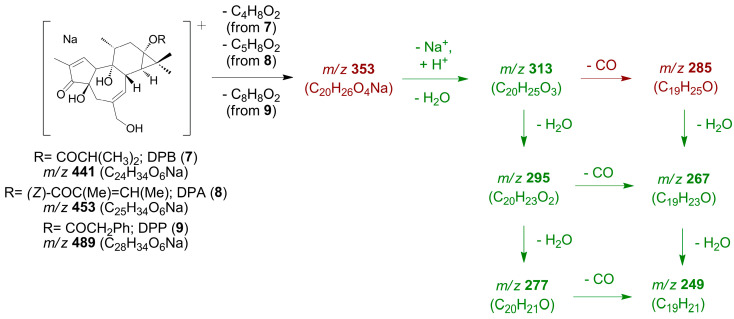
Proposed fragmentation route for selected ions on HRMS^E^ spectra (DIA, high-energy function) for 12-deoxyphorbol 13-acyl derivatives (**7**–**9**, group C compounds; see Section 2.2). For each ion, nominal mass and elemental composition is presented; see a more detailed interpretation of the fragmentation route in Figure S25. In green, common daughter ions with group D compounds (see Section 2.2 and Scheme 4). In deep red, highlighted ions in Figure S24a.

The proposed fragmentation pathways leading to previously mentioned ions can be found in Scheme 3 and Figure S25.

On the other hand, a further comparison of the spectroscopic and spectrometric data of the compounds isolated and not mentioned above, with those reported in the literature, allowed for the identification of three known tetracyclic 13,20-diesters of 12-deoxyphorbol, assigned as 12-deoxyphorbol 20-acetate 13-isobutyrate (AcDPB (**10**)) [32], 12-deoxyphorbol 20-acetate 13-angelate (AcDPA (**11**)) [33] and 12-deoxyphorbol 20-acetate 13-phenylacetate (AcDPP (**12**)) [33,34] (for HRMS^E^ data, see Table S3 and Figures S26 and S27).

Three additional compounds with similar spectroscopic characteristics to those mentioned above were also isolated.

**Figure 6:**
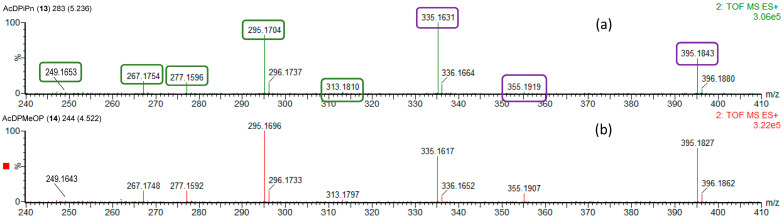
Comparison of HRMS^E^ spectra (DIA) [26] for (**a**) AcDPiPn (**13**) and (**b**) AcDPMeOP (**14**), *m*/*z* range 240–410 (*m*/*z* range 240–600 in Figure S28) (data acquired in positive ionization with a ramp trap collision energy of the high-energy function set at 10–40 eV). See proposed fragmentation route for selected ions in Scheme 4, together with color key.

**Scheme 4:**
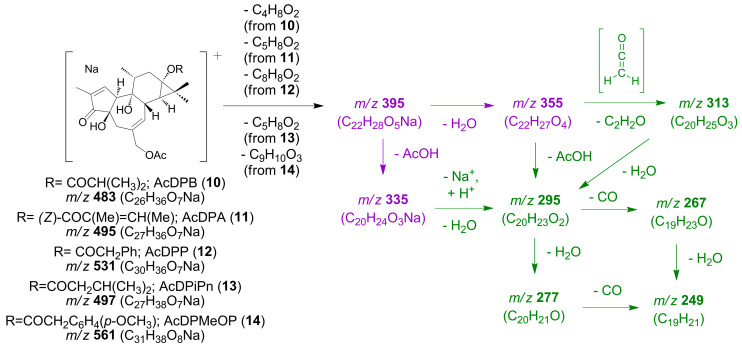
Proposed fragmentation route for selected ions on HRMS^E^ spectra (DIA, high-energy function) for 12-deoxyphorbol 20-acetate-13-acyl derivatives (**10**–**14**, group D compounds; see Section 2.2). For each ion, nominal mass and elemental composition is presented; see a more detailed interpretation of the fragmentation route in Figure S29. In green, common daughter ions with group C compounds (see Section 2.2 and Scheme 3). In purple, highlighted ions in Figure 6a.

Compounds **13** and **14**, both of them obtained as an amorphous powder, presented [M + Na]^+^ molecular ions in their HRMS^E^ spectra at *m*/*z* 497.2527 (calcd for C_27_H_38_O_7_Na, 497.2515) and 561.2455 (calcd for C_31_H_38_O_8_Na, 561.2464), respectively (Table 5, Figure S28), which provide the molecular formulas C_27_H_38_O_7_ for compound **13** and C_31_H_38_O_8_ for compound **14**. Daughter ions were observed in their HRMS^E^ spectra at *m*/*z* 313.1804, 295.1698, 277.1592, 267.1749 and 249.1643 (calculated), which could be assigned to losses of ketene, ketene and water (1 and 2 molecules), ketene and water and CO and, finally, ketene and 2 molecules of water and CO, respectively, from a precursor ion at *m*/*z* 355.1909 (calculated) (Table 5, Figure 6). Alternatively, the ion at *m*/*z* 295 (nominal mass) could be understood to originate from the loss of acetic acid from the parent ion at *m*/*z* 355.1909 (calculated) or from the loss of water from another parent ion at *m*/*z* 335.1623 (calculated). In turn, ions at *m*/*z* 355 and 335 (nominal masses) could originate from losses of water or acetic acid, respectively, from an ion at *m*/*z* 395.1834 (calculated). The latter ion would be prominently apparent (relative to [M + Na]^+^ ion) not only in the HRMS^E^ spectra of compounds **13** and **14**, (Figure S28) but as well as in the HRMS^E^ spectra of AcDPB (**10**), AcDPA (**11**) and AcDPP (**12**) (Figure S26). The formation of this common daughter ion could be understood by the loss of isobutyric, angelic and phenylacetic acid, respectively, from [M + Na]^+^ ions for the compounds AcDPB (**10**), AcDPA (**11**) and AcDPP (**12**) and by the loss of carboxilic acids of the formulas C_5_H_10_O_2_ and C_9_H_10_O_3_, respectively, from [M + Na]^+^ ions for compounds **13** and **14.** The proposed fragmentation pathways leading to previously described ions can be found in Scheme 4 and Figure S29.

Furthermore, compounds **13** and **14** exhibited ^1^H and ^13^C NMR spectra (Table 3 and Table 6) very similar to those of AcDPB (**10**), AcDPA (**11**) and AcDPP (**12**), with differences in the nature of the acyloxy fragment at C-13. The ^1^H NMR spectra of compound **13** showed a doublet at δ_H_ 0.96 ppm (6.9 Hz, 6H), correlated in the ^1^H-^1^H COSY spectrum with a multiplet at δ_H_ 2.08 ppm (1H), which, in turn, correlated with a signal at δ_H_ 2.21 ppm (7.2 Hz, 2H), which is consistent with a 3-methylbutanoate moiety (Figure S13c). On the other hand, the HSQC experiment showed correlations between these signals and those appearing in the ^13^C NMR spectrum at δc 22.8 and 22.7 ppm (*q*), 27.0 ppm (*d*) and 44.4 ppm (*t*), which were assigned to C-4′/C-5′, C-3′ and C-2′, respectively (Figure S13d,e). Correlations observed in the HMBC between signals assigned to H-2′ with those assigned to C-4′/C-5′ and between signals corresponding to H-3′ and a singlet at δ_C_ 176.9 ppm, assigned to C-1′ (Figure S13g,i), confirmed the presence of the 3-methylbutanoate moiety, which would be consistent with the observed loss of a carboxylic acid of the formula C_5_H_10_O_2_ from the [M + Na]^+^ molecular ion, in its HRMS^E^ spectra, as discussed above. A further examination of the NMR data showed that an acetate group can be located at C-20, based on a heteronuclear correlation observed between resonances at δ_H_ 4.48 ppm (H_2_-20) and δ_C_ 172.6 ppm (RCOO), which, in turn, was further correlated with a singlet at δ_H_ 2.02 ppm (3H); this, conversely, supported a C-13 location for 3-methylbutanoate substituent. Therefore, a structure of 12-deoxyphorbol 20-acetate 13-(3-methyl)butanoate (AcDPiPn) was assigned to compound **13**.

**Figure 7:**
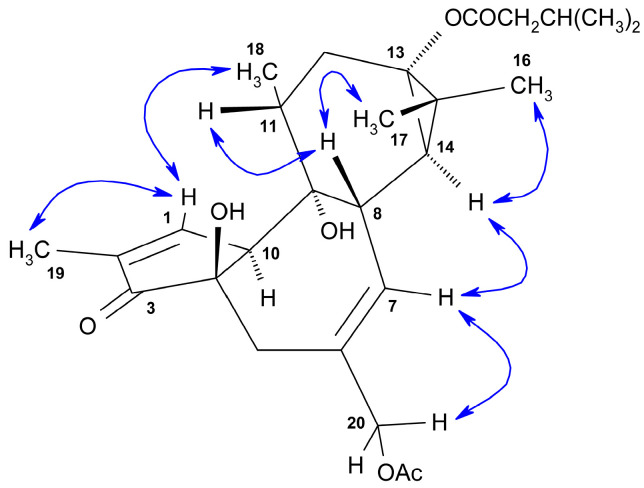
Selected NOESY correlations (blue arrows) for AcDPiPn (**13**).

On the other hand, the analysis of the ^1^H and ^13^C NMR spectra for compound **14** showed close similarities with those of compound **13**; the main differences being the presence of a group of ^1^H and ^13^C resonances correlated in the HMBC spectrum [δ_C_ 175.7 ppm (C-1′), δ_H_ 3.56 ppm (*s*, 2H, H-2′); δ_C_ 127.1 ppm (C-3′), δ_H_ 6.86 ppm (*d*, 8.4 Hz, 2H, H-4′/8′); δ_C_ 131.4 ppm (C-4′/8′), δ_H_ 7.18 ppm (*d*, 8.4 Hz, 2H, H-5′/7′); δ_C_ 160.6 ppm (C-6′), δ_H_ 3.77 ppm (s 3H, C-9′ (OCH_3_)] (Figure S14g–i) and assigned to a *p*-methoxyphenylacetoxy group, and the absence of signals consistent with a 3-methylbutanoate group. The presence of a *p*-methoxyphenylacetoxy group would be consistent with the observed loss of a carboxylic acid of the formula C_9_H_10_O_3_ from the [M + Na]^+^ molecular ion in its HRMS^E^ spectra, as described above. Therefore, a structure for compound **14** was proposed as 12-deoxyphorbol 20-acetate 13-(*p*-methoxy)phenylacetate (AcDPMeOP). NOESY correlations of H_3_-19/H-1/H_3_-18α and H-11β/H-8β/H_3_-17β, on the one hand, and of H_2_-20/H-7/H-14α/H_3_-16α, on the other (Figure 7 for AcDPiPn (**13**)), were consistent with a relative configuration 4*R(S)*,*8S(R)*,9*S(R)*,10*S(R)*,11*R(S)*,13*S(R)*,14*R(S)* for both AcDPiPn (**13**) and AcDPMeOP (**14**).

**Figure 8:**
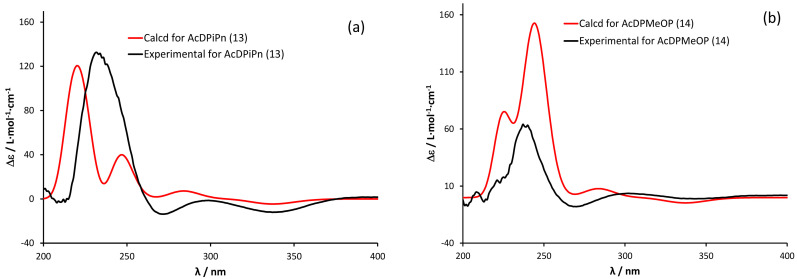
Calculated and experimental ECD spectra of (**a**) 12-deoxyphorbol 20-acetate-13-isopentanoate (**13**); (**b**) 12-deoxyphorbol 20-acetate-13-p-methoxy-phenylacetate (**14**).

The absolute configuration for compounds **13** and **14** was determined by the comparison of their experimental electronic circular dichroism (ECD), with the computed ECD spectrum for their 4*R*,8*S*,9*R*,10*S*,11*R*,13*S*,14*R* steroisomers, calculated from quantum mechanical time-dependent density functional theory (TDDFT) calculations with a 6–31 + G(d,p) level of theory, using the Gaussian 16 program [28]. As illustrated in Figure 8a,b, the calculated and theoretical ECD curves matched well, leading to the assignment of the structure and absolute configuration of compounds **13** and **14**, respectively, as (4*R*,8*S*,9*R*,10*S*,11*R*,13*S*,14*R*)-12-deoxyphorbol 20-acetate 13-(3-methyl)butanoate (AcDPiPn) and (4*R*,8*S*,9*R*,10*S*,11*R*,13*S*,14*R*)-12deoxyphorbol 20-acetate 13-(*p*-methoxy)phenylacetate (AcDPMeOP). These absolute configurations match what has been described previously for other tigliane derivatives [29]. Compound **13** is described here for the first time, while a compound with the proposed structure for compound **14** and labeled as RL10, obtained from *E. resinifera*, had been described previously [27], but without a detailed assignment of its spectroscopic and spectrometric data.

**Figure 9:**
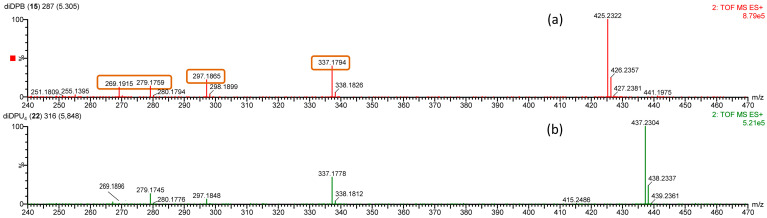
Comparison of HRMS^E^ spectra (DIA) [26] for (**a**) diDPB (**15**) and (**b**) diDPU_4_ (**22**), *m*/*z* range 240–470 (data acquired in positive ionization with a ramp trap collision energy of the high-energy function set at 10–40 eV). See proposed fragmentation route for selected ions in Scheme 5, together with color key.

**Scheme 5:**
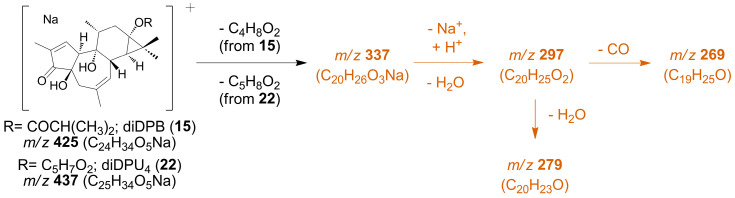
Proposed fragmentation route for selected ions on HRMS^E^ spectra (DIA, high-energy function) for 12,20-dideoxyphorbol 13-acyl derivatives (**15** and **22**, group E compounds; see Section 2.2). In brown, highlighted ions in Figure 9a. For each ion, nominal mass and elemental composition is presented; see a more detailed interpretation of the fragmentation route in Figure S30.

Finally, compound **15** was isolated as an amorphous powder and showed a [M + Na]^+^ molecular ion in its HRMS^E^ spectrum at *m*/*z* 425.2322 (calcd for C_24_H_34_O_5_Na, 425.2304) (Table 7, Figure 9), which allows for the assignment of molecular formula C_24_H_34_O_5_ for compound **15**. Daughter ions from this molecular ion were observed in its HRMS^E^ spectra at *m*/*z* 337.1780, 297.1855, 279.1749 and 269.1905 (calculated) which could be assigned to losses of a neutral fragment of formula C_4_H_8_O_2_ and further losses of water, 2 molecules of water and a loss of water and CO, respectively (Table 7, Figure 9). A proposed fragmentation pathway leading to previously described ions can be found in Scheme 5 and Figure S30. A similar loss of a neutral fragment of formula C_4_H_8_O_2_ can be observed in the HRMS^E^ of 12-deoxyphorbol 13-isobutyrate (DPB, **7**) (see Table S2, Figure S24).

**Figure 10:**
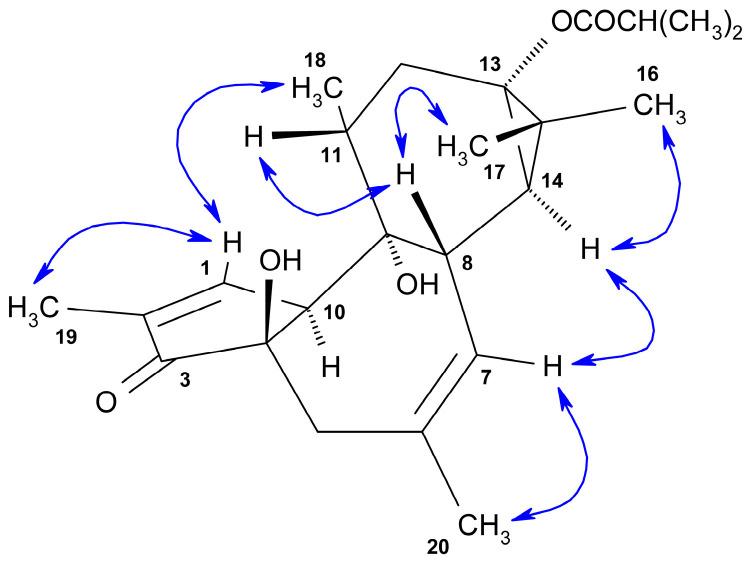
Selected NOESY correlations (blue arrows) for diDPB (**15**).

This compound presented ^1^H and ^13^C NMR spectra (Table 3 and Table 6) with similar signals to those presented by 12-deoxyphorbol 13-isobutyrate (DPB (**7**)); the main differences were the absence of a signal corresponding to a hydroxymethylene group at C-20 and the presence of a new methyl group (δ_H_ 1.73 ppm, δ_C_ 25.8 ppm). This ^1^H-NMR signal correlates, on the one hand, with the ^13^C-NMR resonances for C-5, C-6 and C-7 in the HMBC experiment (Figure S15f–h) and, on the other, with the resonance signals for H-5a, H-5b, H-7 and H-8 in the ^1^H-^1^H COSY spectrum (Figure S15c). Therefore, this is consistent with the presence of a methyl group at C-20 and supported the assignment of compound **15** as 12,20-dideoxyphorbol 13-isobutyrate (diDPB). NOESY correlations of H_3_-19/H-1/H_3_-18α, H-11β/H-8β/H_3_-17β and H_3_-20/H-7/H-14α/H_3_-16α (Figure 10) were in agreement with a relative configuration 4*R(S)*,8*S(R)*,9*R(S)*,10*S(R)*,11*R(S)*,13*S(R)*,14*R(S)* for compound **15**, which is in accordance with the one previously described for other tigliane derivatives [29].

**Figure 11:**
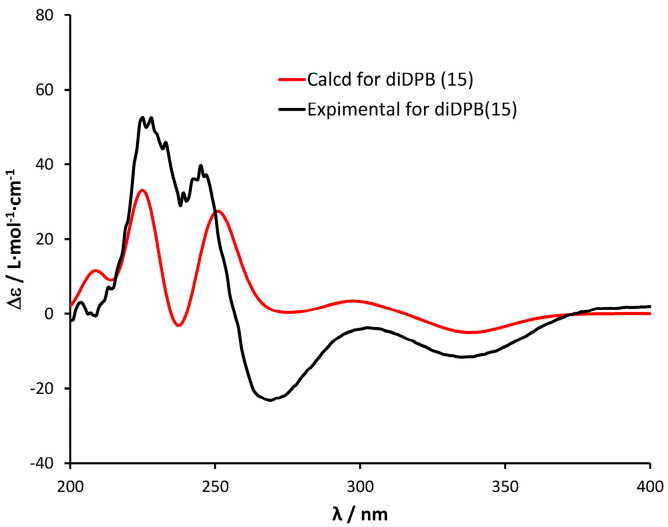
Calculated and experimental ECD spectra of 12,20-dideoxyphorbol 13-isobutyrate (**15**).

The absolute configuration for compound **15**, based on the data described above for AcDPiPn (**13**) and AcDPMeOP (**14**), was proposed as 4*R*,8*S*,9*R*,10*S*,11*R*,13*S*,14*R*. The comparison of the experimental electronic circular dichroism (ECD) spectra with the computed ECD spectra [28] for compound **15** (Figure 11) showed that both were in good agreement which, in turn, led to the assignment of the structure and absolute configuration of **15** as (4*R*,8*S*,9*R*,10*S*,11*R*,13*S*,14*R*)-12,20-dideoxyphorbol 13-isobutyrate (diDPB). As mentioned above, this absolute configuration also matches with what has been described previously for other tigliane derivatives [29]. Compound **15** had been previously described by Hergenhahn et al. [27,35] but no detailed discussion of its structural characterization could be found there. On the other hand, Kulyal et al. reported its isolation from the seed oil of *Jatropha curcas* but the spectroscopic data described were scarce and incorrect [36].

Some conclusions can be drawn from the ECD spectra presented in this work. On the one hand, no clear influence of substituents at C-16 in 12-deoxy-16-hydroxyphorbol esters described here (**1**–**6**), on the Cotton effects in the ECD spectra, can be drawn from the experimental data (see Figure 5 and Figure S19). Several Cotton effects, due to π → π* transitions, have been described for 12,13-disubstited phorbol esters (no ester at C-16) in the range between 200 and 260 nm in their ECD spectra [37]. Equivalent ones in 12-deoxy-16-hydroxyphorbol esters (**1**–**6**) seem to be overlapping in their experimental ECD spectra, which is consistent with what is observed in the corresponding calculated ECD spectra (see, for instance, Figure 5a for observed and calculated ECD spectra for AcDPPI (**4**)).

On the other hand, the calculated ECD spectra for 12-deoxyphorbol esters **13** and **14** and for 12,20-dideoxyphorbol ester **15**, predict positive, non-overlapped Cotton effects in the 220–240 nm and 240–260 nm ranges, which can be attributed to transitions π → π* [38]. These Cotton effects can be observed in the experimental ECD spectra for diDPB (**15**) (Figure 11).

Finally, resiniferatoxin (**16**) [39] and two known tricyclic esters of ingol type (**17**, **18**) [9,12,27] were also identified by a comparison of their spectroscopic and spectrometric data with those reported in the literature.

### 2.2. Identification of 12-Deoxyphorbol Derivatives by UHPLC-HRMS^E^: Targeted and Biased Non-Targeted Analysis

As a result of the analysis of the structural elucidation of 12-deoxyphorbols **1**–**15**, as described above, specific fragmentation patterns are observed in their high-energy HRMS^E^ spectra, which can be associated with specific structural classes (groups A–E), namely:

A. 12-Deoxy-16-hydroxyphorbol 13,16-diacyl derivatives (**1**–**3**) (Scheme 1).
B. 12-Deoxy-16-hydroxyphorbol 20-acetate 13,16-diacyl derivatives (**4**–**6**) (Scheme 2).
C. 12-Deoxyphorbol 13-acyl derivatives (**7**–**9**) (Scheme 3).
D. 12-Deoxyphorbol 20-acetate 13-acyl derivatives (**10**–**14**) (Scheme 4).
E. 12,20-Dideoxyphorbol 13-acyl derivatives (**15**) (Scheme 5).

Simplified schemes of the fragmentation routes for each one of the above-mentioned structural classes, with characteristic daughter ions (nominal masses and elemental composition), can be found in Scheme 1, Scheme 2, Scheme 3, Scheme 4 and Scheme 5; a more detailed description of the fragmentation patterns, including proposed structures and calculated masses for the daughter ions, can be found in Figures S20, S22, S25, S29 and S30 in the Supplementary Materials section.

These characteristic fragmentation patterns, combined with the inclusion of the characteristic [M + Na]^+^ ions for each compound, can be applied to a targeted analysis of isolated compounds **1**–**15** in the chromatographic fractions used for their isolation, which were initially selected for isolation studies exclusively on the grounds of an initial NMR screening (Table 8).

As expected, a comparison of the extracted ion chromatograms for ions associated with characteristic fragments for each structural class (A–E) (especially those observed to be more abundant in the HRMS^E^ spectra of isolated compounds, see Figure 2, Figure 4, Figure 6, Figure 9 and Figure S24) and [M + Na]^+^ ions for each compound reveals the presence of 12-deoxyphorbols **1**–**15** as components in chromatographic fractions F and G-6 to G-9 (Table 8), in accordance with the isolation experiment results.

On the other hand, this analysis can also be extended to the search for other components which present ions corresponding to characteristic fragmentations of each structural class A–E mentioned above, but not described in the isolation section (biased non-targeted analysis [40,41]).

The analysis of the HRMS^E^ spectra of these components reveals the presence of ions at a higher *m*/*z*, which could be attributed to the parent ions of the characteristic daughter ions described for the structural classes A–E (Scheme 1, Scheme 2, Scheme 3, Scheme 4 and Scheme 5). This, in turn, would allow a level-2 or 3 identification of these components detected and not described in the isolation section [42]. Following this approach, four additional components have been identified in the chromatographic fractions of *E. resinifera* (Table 8).

The analysis of 12-deoxy-16-hydroxyphorbol 13,16-diesters daughter ions (group A compounds, Scheme 1) leads to the identification of component **19** (DPPU_1_) in fraction G-9 (entry 25, Table 8) with an observed proposed parent ion [M + Na]^+^ at *m*/*z* 589.2787 (calcd for C_33_H_42_O_8_Na, 589.2777) (Figure 2d, Table 1). A structure of 12-deoxy-16-hydroxyphorbol 13,16-diester is attributed to this component, where the ester side chain at C-13 would be a phenylacetate substituent, as suggested by the presence of a daughter ion at *m*/*z* 487.2090 (calcd for C_28_H_32_O_6_Na, 487.2097). Carboxylate moiety at C-16 would be assigned elemental formula C_5_H_9_O_2_, which would correspond to C_5_H_10_O_2_ for the protonated carboxylic acid (Scheme 1 and Figure S20). Only one unsaturation would be deduced from such a formula, which, in turn, would be consistent with several possible structures such as pentanoate, 3-methylbutanoate, 2-methylbutanoate or 2,2-dimethylpropanoate; therefore, only a level-3 identification would be possible for DPPU_1_ (**19**) [42]. One of the tentative structures for DPPU_1_ (**19**), 12-deoxy-16-hydroxyphorbol 13-phenylacetate 16-(3-methyl)butanoate, has been described previously as candletoxin B from *E. poisonii* [43].

The analysis of 12-deoxy-16-hydroxyphorbol 20-acetate 13,16-diesters daughter ions (group B compounds, Scheme 2) leads to the identification of component **20** (AcDPPU_2_) in fraction G-7 (entry 13, Table 8) with an observed proposed parent ion [M + Na]^+^ at *m*/*z* 603.2540 (calcd for C_33_H_40_O_9_Na, 603.2570) (Figure 4d and Figure S21e, Table 9). A structure of 12-deoxy-16-hydroxyphorbol 20-acetate 13,16-diester is proposed for this component, where the ester side chain at C-13 would be a phenylacetate substituent, according to the presence of a daughter ion at *m*/*z* 529.2205 (calcd for C_30_H_34_O_7_Na, 529.2202). Carboxylate moiety at C-16 would be assigned elemental formula C_3_H_5_O_2_, which, in turn, would correspond to C_3_H_6_O_2_ for the protonated carboxylic acid (Scheme 2 and Figure S22). This formula is consistent with the presence of one unsaturation and, therefore, a propionate structure could be assigned to this carboxylate moiety at C-16. Consequently, AcDPPU_2_ (**20**) could be described as 12-deoxy-16-hydroxyphorbol 20-acetate 13-phenylacetate-16-propionate (level-2 identification [42]), which to our knowledge has not been described before.

On the other hand, the further analysis of 12-deoxy-16-hydroxyphorbol 20-acetate 13,16-diesters daughter ions (group B compounds, Scheme 2) leads to the identification of component **21** (AcDPPU_3_) in fraction G-8 (entry 18, Table 8) with an observed proposed parent ion [M + Na]^+^ at *m*/*z* 631.2911 (calcd for C_35_H_44_O_9_Na, 631.2883) (Figure 4e and Figure S21f, Table 9). A structure of 12-deoxy-16-hydroxyphorbol 20-acetate 13,16-diester is attributed to this component, where the ester side chain at C-13 would be a phenylacetate substituent, according to the presence of a daughter ion at *m*/*z* 529.2214 (calcd for C_30_H_34_O_7_Na, 529.2202). Carboxylate moiety at C-16 would be assigned elemental formula C_5_H_9_O_2_, which would correspond to C_5_H_10_O_2_ for the protonated carboxylic acid (Scheme 2 and Figure S22). This formula for the carboxylate moiety, which contains only one unsaturation, would be consistent with several possible structures such as pentanoate, 3-methylbutanoate, 2-methylbutanoate or 2,2-dimethylpropanoate, as described for DPPU_1_ (**19**), so only a level-3 identification would be possible for AcDPPU_3_ (**21**) [42]. One of the tentative structures for AcDPPU_3_ (**21**), 12-deoxy-16-hydroxyphorbol 20-acetate 13-phenylacetate 16-(3-methyl)butanoate, has been described previously as candletoxin A from *E. poisonii* [43].

Finally, the analysis of 12,20-dideoxy-phorbol 13-isobutyrate (**15**) daughter ions (group E compounds, Scheme 5) leads to the identification of component **22** (diDPU_4_) in fraction G-7 (entry 14, Table 8) with an observed proposed parent ion [M + Na]^+^ at *m*/*z* 437.2304 (calcd for C_25_H_34_O_5_Na, 437.2304) (Figure 9b, Table 7). A structure of 12,20-dideoxy-phorbol 13-ester is proposed for this component, where the elemental composition for ester moiety at C-13 (C_5_H_7_O_2_; C_5_H_8_O_2_ for the corresponding carboxylic acid) would be consistent with a tigliate, an angelate or a 3-methylbut-2-enate moiety, so only a level-3 identification would be possible for diDPU_4_ (**22**) [42]. One of the tentative structures for diDPU_4_ (**22**), 12,20-dideoxy-phorbol 13-angelate, has been described before for a component in *E. resinifera* [35].

## 3. Materials and Methods

### 3.1. General Experimental Procedures

Optical rotations were determined with a digital polarimeter. Infrared spectra were recorded on an FT-IR spectrophotometer and reported as wavenumber (cm^−1^). ECD and UV spectroscopic data were obtained with a J-1500 CD spectrometer (JASCO, Tokyo, Japan). NMR data were recorded on Agilent 400 and 500 MHz NMR spectrometers (Agilent, Santa Clara, CA, USA) with SiMe_4_ as the internal reference. Chemical shifts are expressed in ppm (δ) referenced to the solvent used. NMR assignments and correlation with proposed structures were made using a combination of 1D and 2D NMR techniques. HPLC was performed with a Merck-Hitachi LaChrom (Merck, Darmstadt, Germany) apparatus equipped with a pump (L-7100) and a differential refractometer detector (L-7490), and an Elite LaChrom-Hitachi apparatus (Merck, Darmstadt, Germany) equipped with a pump (L-2130), a UV–vis detector (L-2400) and a differential refractometer detector (L-2490). LiChroCART LiChrospher Si 60 (5 μm, 250 mm × 4 mm and 10 μm, 250 mm × 10 mm) and LiChroCART ChiraSpher NT, based on silica gel particles coated with the optically active polymer poly(*N*-acryloyl-*S*-phenylalanine ethyl ester), (5 μm, 250 mm × 4 mm) columns, were used for normal-phase chromatography for purification experiments. LiChroCART LiChrospher 100 (10 μm, 250 mm × 10 mm) column was used for reverse-phase HPLC for purification experiments. Where appropriate, compounds were detected at 250 nm.

### 3.2. UHPLC-HRMS^E^ Analysis Conditions

Analyses were performed on a separation system, ACQUITY UPLC H-Class system, with a binary solvent system and an automatic sample manager equipped with a UPLC BEH C18 (2.1 mm × 100 mm, 1.7 mm) column, maintained at a temperature of 55 °C. The mobile phases were prepared using eluent A (0.1% formic acid in water, *v*/*v*) and eluent B (methanol). These phases were delivered at a flow rate of 0.3 mL/min by using a linear gradient program as follows: 0–0.5 min, 25% A; 0.5–5 min, 25–0% A; 5–7 min, 0% A; 7–8 min, 0–25% A; and 8–10 min, 25% A [8]. The injection volume of all samples was 2 μL.

UHPLC system was hyphenated with a quadrupole time-of-flight tandem high-resolution mass spectrometer (Xevo-G2-S QTOF; Waters, Manchester, UK) equipped with an ESI source. The operating parameters in ESI and the data-independent acquisition mode (MS^E^; HRMS^E^ in this manuscript) [26] were set as follows in positive mode: sample cone voltage of 30 V; source temperature of 120 °C; cone gas flow of 10 L/h; desolvation gas flow of 850 L/h; capillary voltage and desolvation temperature were set at 0.7 kV and 400 °C, respectively. In the HRMS^E^ mode (positive mode), the trap collision energy of the low-energy function was set at 6 eV, while the ramp trap collision energy of the high-energy function was set at 10–40 eV or to 60–120 eV, depending on the experiment.

Mass accuracy and reproducibility were obtained by calibration of the mass spectrometer over a range of 100–1200 Da, using sodium formiate solution. Leucine-enkephalin (*m*/*z* 556.2771 in positive-ion mode) was used as the external reference of LockSpray infused at a constant flow of 20 μL/min. Calibration was made considering ions as atom aggregates.

Data acquisition and further processing were performed by MassLynx 4.1 software package (Waters, Manchester, UK) in positive-ion mode. Automated peak detection was performed using the “targeted-fragment ion confirmation” algorithm, while the “non-targeted” algorithm was used to carry out a biased non-targeted analysis [38,39]; both algorithms are included in the ChromaLynx XS module within the MassLynx package. Retention time and mass tolerance were adjusted to ±0.2 min and ±10 mDa for targeted analysis. Number of chromatograms to extract (x = 8) and noise elimination level (xs = 4) were parameters adjusted to improve peak detection. The Elemental Composition algorithm in the MassLynx 4.1 software package was used for the elemental composition annotation of ions. Further annotation of ions was assisted by the Mass Fragment module in the MassLynx 4.1 software package, which is an implementation of the EPIC algorithm [44].

### 3.3. Plant Material

*E. resinifera* specimens were identified by Prof. Ahmed Nafis (University Chouaïb Doukkali, Morocco). Latex from *E. resinifera* was collected in November 2018 in Demnate, Beni Mellal-Khenifera province (Morocco) and was obtained by making repeated cuts along branches of plants and collecting the white milky exudates.

### 3.4. Extraction and Isolation

An air-dried sample of *E. resinifera* Berg latex (250 g) was macerated in EtOAc (200 mL, 3 times) for 1 h at room temperature. The resulting solutions were combined and filtered using strips of filter paper to eliminate suspended particles of plant material. A short bed of TLC-grade silica gel (30 g) was used for the second filtration to eliminate viscous immiscible liquids. The solvent from the resulting solution was evaporated under reduced pressure to yield a yellowish extract. This extract was then triturated with acetonitrile and the resulting mixture was filtered using strips of filter paper to remove a white precipitate (120 g), mainly containing triterpenoids. The solvent from the filtrate was evaporated under reduced pressure to yield a residue (70.4 g) which was then subjected to column chromatography over silica gel (200–300 mesh), eluted sequentially with an increasingly polar gradient of ethyl acetate in petroleum ether (from 1:0 to 1:1, *v*/*v*), EtOAc and MeOH, to give 8 fractions (A–H). These fractions were examined by proton nuclear magnetic resonance (^1^H-NMR). Fractions F and G and further sub-fractions presented analytical data consistent with the presence of 12-deoxyphorbol derivatives, as described below.

Fraction F (2003 mg) was separated on a silica gel column (200–300 mesh) and eluted with an increasingly polar gradient of ethyl acetate in petroleum ether (from 1:20 to 1:1, *v*/*v*) to afford two major diterpenic components: 3,8,12-tri-*O*-acetyl-7-*O*-(4-methoxyphenyl)acetylingol (EOF1 (**17**), 199.46 mg) and 3,8,12-tri-*O*-acetyl-7-*O*-phenylacetylingol (EOF2 (**18**), 100 mg). Further purification via normal-phase HPLC yielded 12-deoxyphorbol 20-acetate 13-isobutyrate (AcDPB (**10**), 179.9 mg), 12-deoxyphorbol 20-acetate 13-angelate (AcDPA (**11**), 187.1 mg), 12-deoxyphorbol 20-acetate 13-phenylacetate (AcDPP (**12**), 41.0 mg) and 12-deoxyphorbol 20-acetate 13-(3-methyl)butanoate (AcDPiPn (**13**), 8.3 mg)

Fraction G (300 mg) was purified on a silica gel column (200–300 mesh), using an increasingly polar gradient of ethyl acetate in petroleum ether (from 7:3 to 1:0, *v*/*v*) to obtain ten sub-fractions (G-1–G-10). Fractions G6 to G9 presented analytical data consistent with the presence of 12-deoxyphorbol derivatives.

Sub-fractions G-6 to G-9 were further purified through semi-preparative reverse-phase HPLC. The mobile phases were prepared using eluent A (AcN), eluent B (MeOH) and eluent C (H_2_O). These phases were delivered at a flow rate of 3 mL/min by using a linear gradient program as follows: 0–55 min, 30–70% A, 10–15% B; 55–65 min, 70–100% A, 15–0% B; 65–70 min, 100–30% A, 0–10% B.

Sub-fraction G-6 (94 mg) was purified to afford 12-deoxy-16-hydroxyphorbol 20-acetate 16-benzoate 13-phenylacetate (AcDPPBz (**6**), 1.0 mg), 12-deoxyphorbol 20-acetate 13-(*p*-methoxyphenyl)acetate (AcDPMeOP (**14**), 0.5 mg) and 12,20-dideoxyphorbol 13-isobutyrate (diDPB (**15**), 4 mg)

Sub-fraction G-7 (66 mg) was purified to yield 12-deoxy-16-hydroxyphorbol 20-acetate 16-isobutyrate 13-phenylacetate (AcDPPI, (**4**), 5,8 mg), 12-deoxy-16-hydroxyphorbol 20-acetate 13-phenylacetate 16-tigliate (AcDPPT (**5**), 10 mg), AcDPPBz (**6**) (6 mg), AcDPP (**12**) (0.5 mg) and AcDPMeOP (**14**) (1.3 mg).

Similarly, AcDPPI (**5**) (3.0 mg), AcDPPT (**5**) (5.8 mg), AcDPP (**12**) (11.8 mg) and resiniferatoxin (RTX (**16**)) (15.2 mg) were obtained via the reverse-phase HPLC purification of sub-fraction G-8 (50 mg).

Finally, fraction G-9 (40 mg) yielded 12-deoxyphorbol 16-isobutyrate 13-phenylacetate (DPPI (**1**), 1.5 mg), 12-deoxyphorbol 13-phenylacetate 16-tigliate (DPPT (**2**), 2.3 mg), 12-deoxy-16-hydroxyphorbol 16-benzoate 13-phenylacetate (DPPBz (**3**), 3.3 mg), 12-deoxyphorbol 13-isobutyrare (DPB (**7**), 10.2 mg), 12-deoxyphorbol 13-angelate (DPA (**8**), 8.3 mg) and 12-deoxyphorbol 13-phenylacetate (DPP (**9**), 4.5 mg),

(4*R*,*8S*,9*R*,10*S*,11*R*,13*S*,14*R*,15*S*)-12-Deoxy-16-hydroxyphorbol 16-isobutyrate 13-phenylacetate (DPPI (**1**)) [8]: UV (MeOH) λmax (log ε) 291 (0.44); ECD (c, 6.0 × 10^−4^ M, MeOH) λ max (Δε) 210 (−0.62), 227 (3.94), 268 (−0.43), 302 (0.02), 337 (−0.17).

(4*R*,*8S*,9*R*,10*S*,11*R*,13*S*,14*R*,15S)-12-Deoxy-16-hydroxyphorbol 13-phenylacetate 16-tigliate (DPPT (**2**)) [8]: UV (MeOH) λmax (log ε) 294 (0.54); ECD (c, 8.2 × 10^−4^ M, MeOH) λ max (Δε) 205 (0.15), 220 (−0.14), 227 (0.05), 232 (−0.07), 244 (1.58), 267 (−1.06), 299 (−0.18).

^1^H-NMR and ^13^C-NMR spectra can be found in Supplementary Materials for com- pounds **1** and **2** (Figures S1a–S2a and S1b–S2b). HRESIMS data for compounds **1** and **2** can be found in Table S1 and Figure 2a,b.

(4*R*,*8S*,9*R*,10*S*,11*R*,13*S*,14*R*,15*S*)-12-Deoxy-16-hydroxyphorbol 16-benzoate 13-phenylacetate (DPPBz (**3**)): amorphous powder; [α]${}_{D}^{20}$ + 27.4 (c 0.34, CHCl_3_); UV (MeOH) λmax (log ε) 291 (−0.55); ECD (c, 6.6 × 10^−4^ M, MeOH) λ max (Δε) 217 (0.35), 225 (0.04), 246 (1.55), 267 (−1.04), 299 (−0.01), 337 (−0.48); IR (film) ν_max_ 3416.78, 2925.08, 1712.19, 1633.64, 1454.77, 1386.12, 1335.45, 1244.85, 1100.33, 1015.65, 947.54, 927.99, 754.5, 699.95, 665.06, 626.32, 534.64, 469.85, 456.65 cm^−1^; ^1^H and ^13^C NMR data, see Table 2 and Table 3; HRESIMS *m*/*z* 609.2463 [M + Na]^+^ (calcd. for C_35_H_38_O_8_Na 609.2464); for further details, see Table 1 and Figure 2c.

(4*R*,8*S*,9*R*,10*S*,11*R*,13*S*,14*R*,15*S*)-12-Deoxy-16-hydroxyphorbol 20-acetate 16-isobutyrate 13-phenylacetate (AcDPPI (**4**)): amorphous powder; [α]${}_{D}^{20}$ + 5.4 (c 0.2, CHCl_3_); UV (MeOH) λmax (log ε) 291 (0.05), 319 (−0.17); ECD (c, 7.6 × 10^−4^ M, MeOH) λ max (Δε) 233 (−0.19), 245 (1.37), 248 (1.31), 251 (1.19), 272 (−0.85), 297 (−0.06), 336 (−0.71); IR (film) ν_max_ 3411.04, 3124.62, 2924.66, 2578.29, 1711.84, 1535.18, 1378.03, 1364.35, 1332.08, 1296.70, 1242.28, 1200.80, 1135.05, 1107.46, 1020.51, 820.26, 755.8, 498.17, 474.99 cm^−1^; ^1^H and ^13^C NMR data, see Table 2 and Table 3; HRESIMS *m*/*z* 617.2755 [M + Na]^+^ (calcd for C_34_H_42_O_9_Na 617.2727); for further details, see Table 4, Figure 4a and Figure S21b.

(4*R*,8*S*,9*R*,10*S*,11*R*,13*S*,14*R*,15*S*)-12-Deoxy-16-hydroxyphorbol 20-acetate 13-phenylacetate 16-tigliate (AcDPPT (**5**)): amorphous powder; [α]${}_{D}^{20}$ + 26.9 (c 0.25, CHCl_3_); UV (MeOH) λmax (log ε) 289 (−0.25), 318 (−0.55); ECD (c, 7.6 × 10^−4^ M, MeOH) λ max (Δε) 205 (0.15), 220 (−0.14), 227 (0.05), 232 (−0.07), 244 (1.58), 267 (−1.06), 299 (−0.18); IR (film) ν_max_ 3408.38, 3099.26, 2925.57, 2582.32, 1705.14, 1526.07, 1380.41, 1362.73, 1270.85, 1200.37, 1141.67, 1107.62, 1077.14, 1056.42, 1017.71, 821.66, 755.56, 498.35, 493.63, 470.18, 457.25 cm^−1^; ^1^H and ^13^C NMR data, see Table 2 and Table 3; HRESIMS *m*/*z* 629.2745 [M + Na]^+^ (calcd. for C_35_H_42_O_9_Na 629.2727); for further details, see Table 4, Figure 4b and Figure S21c.

(4*R*,8*S*,9*R*,10*S*,11*R*,13*S*,14*R*,15*S*)-12-Deoxy-16-hydroxyphorbol 20-acetate 16-benzoate 13-phenylacetate (AcDPPBz (**6**)): amorphous powder; [α]${}_{D}^{20}$ + 11.94 (c 0.18, CHCl_3_); UV (MeOH) λmax (log ε) 290 (−0.49); ECD (c, 7.4 × 10^−4^ M, MeOH) λ max (Δε) 205 (0.15), 220 (−0.14), 227 (0.06), 232 (−0.07), 244 (1.58), 267 (−1.06), 299 (−0.18); IR (film) ν_max_ 3479.26, 3187.11, 2983.15, 2629.86, 1746.08, 1565.88, 1405.59, 1391.64, 1358.72, 1322.63, 1267.13, 1224.82, 1157.75, 1129.61, 1040.92, 836.67, 770.92, 508.13, 484.49, 450.85 cm^−1^; ^1^H and ^13^C NMR data, see Table 2 and Table 3; HRESIMS *m*/*z* 651.2601 [M + Na]^+^ (calcd. for C_37_H_40_O_9_Na 651.2570); for further details, see Table 4, Figure 4c and Figure S21d.

^1^H-NMR, ^13^C-NMR, COSY, HSQC, HMBC and NOESY2D spectra can be found in Supplementary Materials for compounds **3, 4, 5** and **6** (Figures S3a–S6a, S3b–S6b, S3c–S6c, S3(d–f)–S6(d–f), S3(g–i)–S6(g–i)).

^1^H-NMR and ^13^C-NMR spectra can be found in Supplementary Materials for com-pounds **7**, **8**, **9**, **10**, **11** and **12** (Figures S7a–S12a and S7b–S12b). HRESIMS data for compounds **7**–**9** can be found in Table S2, Figures S23a–c and S24a–c. HRESIMS data for compounds **10**, **11** and **12** can be found in Table S3 and Figures S26a–c and S27a–c.

(4*R*,8*S*,9*R*,10*S*,11*R*,13*S*,14*R*)-12-Deoxyphorbol 20-acetate 13-(3-methyl)butanoate (AcDPiPn (**13**)): amorphous powder; [α]${}_{D}^{20}$ + 7.8 (c 0.08, CHCl_3_); UV (MeOH) λmax (log ε) 289 (−0.56); ECD (c, 14.3 × 10^−4^ M, MeOH) λ max (Δε) 213 (−0.05). 232 (2.52), 272 (−0.26), 299 (−0.03). 337 (−0.23), 391 (0.03); IR (film) ν_max_ 3395.34, 3078.68, 2930.26, 2318.33, 1712.41, 1530.44, 1514.07, 1492.18, 1246.98, 845.9, 756.19, 483.77, 468.75, 454.88 cm^−1^; ^1^H and ^13^C NMR data, see Table 3 and Table 6; HRESIMS *m*/*z* 497.2527 [M + Na]^+^ (calcd for C_27_H_38_O_7_Na 497.2515); for further details, see Table 5, Figure 6a and Figure S28a.

(4*R*,8*S*,9*R*,10*S*,11*R*,13*S*,14*R*)-12-Deoxyphorbol 20-acetate 13-(*p*-methoxy)phenylacetate (AcDPMeOP (**14**)): amorphous powder; [α]${}_{D}^{20}$ + 11.5 (c 0.46, CHCl_3_); UV (MeOH) λmax (log ε) 290 (−0.34); ECD (c, 3.1 × 10^−4^ M, MeOH) λ max (Δε) 213 (−0.05), 232 (2.52), 272 (−0.26), 299 (−0.03), 337 (−0.23), 391 (0.03); IR (film) ν_max_ 3364.12, 3085.33, 2926.02, 2344.07, 1698.80, 1544.00, 1380.39, 1347.06, 1331.46, 1301.59, 1243.45, 1203.83, 1165.4, 1106.33, 1014.30, 821.79, 756.00, 498.08, 486.81, 480.33, 476.94, 465.02, 457.12 cm^−1^; ^1^H and ^13^C NMR data, see Table 3 and Table 6; HRESIMS *m*/*z* 561.2455 [M + Na]^+^ (calcd for C_31_H_38_O_8_Na 561.2464); for further details, see Table 5, Figure 6b and Figure S28b.

^1^H-NMR, ^13^C-NMR, COSY, HSQC and HMBC spectra can be found in Supplementary Materials for compounds **13** and **14** (Figures S13a–S14a, S13b–S14b, S13c–S14c, S13(d–f)–S14(d–f) and S13(g–i)–S14(g–i)).

NOESY2D spectrum can be found in Supplementary Materials for compound **13** (Figure S13j).

^13^C-DEPT spectrum can be found in Supplementary Materials for compound **13** (Figure S13k).

(4*R*,8*S*,9*R*,10*S*,11*R*,13*S*,14*R*)-12,20-Dideoxyphorbol 13-isobutyrate (diDPB (**15**)): amorphous powder; [α]${}_{D}^{20}$ + 5.3 (c 0.13, CHCl_3_); UV (MeOH) λmax (log ε) 288 (−0.61); ECD (c, 8.3 × 10^−4^ M, MeOH) λ max (Δε) 209 (−0.01), 225 (1.92), 238 (1.06), 245 (1.45), 269 (−0.85), 303 (−0.14), 335 (−0.43); IR (film) ν_max_ 3361.39, 3047.89, 2900.96, 2295.15, 1695.29, 1515.14, 1498.93, 1477.26, 1234.51, 837.44, 748.63, 478.93, 464.06, 450.33 cm^−1^; ^1^H and ^13^C NMR data, see Table 3 and Table 6; HRESIMS *m*/*z* 425.2322 [M + Na]^+^ (calcd for C_24_H_34_O_5_Na 425.2304); for further details, see Table 7 and Figure 9a.

^1^H-NMR, ^13^C-NMR, COSY, HSQC, HMBC, NOESY2D and ^13^C-DEPT spectra can be found in Supplementary Materials for compound **15** (Figures S15a–j).

^1^H-NMR and ^13^C-NMR spectra can be found in Supplementary Materials for com- pounds **16**, **17** and **18** (Figures S16a–S18a and S16b–S18b).

### 3.5. UHPLC-HRMS^E^ Identification of 12-Deoxyphorbol Esters from E. resinifera

Fraction F and sub-fractions G-6, G-7, G8 and G-9 were analyzed by UHPLC-HRMS^E^ taking into account the specific fragmentation patterns associated with the classes of 12-deoxyphorbol derivatives found from the analysis of isolated compounds, as described above (see Scheme 1, Scheme 2, Scheme 3, Scheme 4 and Scheme 5 and Figures S20, S22, S25, S29 and S30). A comparison of the total ion current (TIC) and extracted ion chromatograms (XICs) at selected *m*/*z* for each analyzed fraction can be found in Figures S31–S41. Identified components in each fraction and sub-fraction, detected on the basis of the alignment of peaks in XICs for molecular ion clusters and selected characteristic fragmentations, together with retention times, can be found in Table 8. Further biased non-targeted analysis [40,41], based on the alignment of peaks in the XICs for characteristic fragmentations for every structural class of 12-deoxyphorbol esters described here (see Scheme 1, Scheme 2, Scheme 3, Scheme 4 and Scheme 5 and Figures S20, S22, S25, S29 and S30), with peaks from XICs for proposed molecular ion clusters in each component found in the biased non-targeted analysis [38,39], allowed for the identification of components DPPU_1_ (**19**), AcDPPU_2_ (**20**), AcDPPU_3_ (**21**) and diDPU_4_ (**22**) (Figure 1), which were not isolated and are described here only on the basis of their HRESIMS data (see structural discussion and identification level [42] in results and discussion section)

DPPU_1_ (**19**): HRESIMS *m*/*z* 589.2787 [M + Na]^+^ (calcd for C_33_H_42_O_8_Na 589.2777); for further details, see Table 1 and Figure 2d.

AcDPPU_2_ (**20**): HRESIMS *m*/*z* 603.2540 [M + Na]^+^ (calcd for C_33_H_40_O_9_Na, 603.2570); for further details, see Figure 4d and Figure S21e, Table 9.

AcDPPU_3_ (**21**): HRESIMS *m*/*z* 631.2911 [M + Na]^+^ (calcd for C_35_H_44_O_9_Na, 631.2883); for further details, see Figure 4e and Figure S21f, Table 9.

diDPU_4_ (**22**): HRESIMS *m*/*z* 437.2304 [M + Na]^+^ (calcd for C_25_H_34_O_5_Na 437.2304); for further details, see Table 7 and Figure 9b.

## 4. Conclusions

Fifteen 12-deoxyphorbol esters (**1**–**15**) have been isolated from *E. resinifera* Berg latex, together with resiniferatoxin (**16**) and ingol derivatives EOF1 (**17**) and EOF2 (**18**). AcDPPI (**4**), a triester of 12-deoxy-16-hydroxyphorbol, and AcDPiPn (**13**), a 12-deoxyphorbol 13,20-diester, are described here for the first time. Additionally, detailed structural elucidation is provided for DPPBz (**3**), AcDPPT (**5**), AcDPPBz (**6**), AcDPMeOP (**14**) and diDPB (**15**), including analysis of their HRMS^E^. The absolute configuration for compounds **4**, **6, 13**, **14** and **15** was established by a comparison of their theoretical and experimental electronic circular dichroism (ECD) spectra. Access to the above-described collection of 12-deoxyphorbol derivatives, with several substitution patterns and attached acyl moieties, allowed for the study of their fragmentation patterns in the collision-induced dissociation of multiple ions, without precursor ion isolation mass spectra experiments (HRMS^E^) [8], which, in turn, revealed a correlation between specific substitution patterns (structural classes A–E) and the fragmentation pathways in their HRMS^E^ spectra. In turn, this allowed for a targeted UHPLC-HRMS^E^ analysis and a biased non-targeted UHPLC-HRMS^E^ analysis [40,41] of 12-deoxyphorbols in *E. resinifera* latex which yielded the detection and identification (level 2 or 3 [42]) of four additional 12-deoxyphorbols, DPPU_1_ (**19**), AcDPPU_2_ (**20**), AcDPPU_3_ (**21**) and diDPU_4_ (**22**), not previously isolated in the initial column fractionation work. To our knowledge, AcDPPU_2_ (**20**), identified as 12-deoxy-16-hydroxyphorbol 20-acetate-13-phenylacetate-16-propionate (identification level 2 [42]), has not been described before.

## Figures and Tables

**Table 1:** Selected ions of high-energy HRMS^E^ experiment (DIA) [26] of DPPBz (**3**) and DPPU_1_ (**19**) (data acquired in ESI positive ionization with a ramp trap collision energy of the high-energy function set at 60–120 eV).

Compound	Observed *m*/*z* (%)	Calculated *m*/*z*	Δm (mDa)	Elemental Composition	Annotation
DPPBz (**3**)	609.2463 (80)	609.2464	−0.1	C_35_H_38_O_8_Na	[M + Na]^+^
	487.2098 (25)	487.2097	+0.1	C_28_H_32_O_6_Na	[M − C_7_H_6_O_2_ + Na]^+^
	473.1934 (9)	473.1940	−0.6	C_27_H_30_O_6_Na	[M − C_8_H_8_O_2_ + Na]^+^
	369.1685 (7)	369.1678	+0.7	C_20_H_26_O_5_Na	[M − C_8_H_8_O_2_ − C_7_H_6_O_2_ + Na]^+^
	351.1574 (24)	351.1572	+0.2	C_20_H_24_O_4_Na	[M − C_7_H_6_O_2_ − C_8_H_8_O − H_2_O + Na]^+^
	323.1629 (2)	323.1623	+0.6	C_19_H_24_O_3_Na	[M − C_7_H_6_O_2_ − C_8_H_8_O − H_2_O − CO + Na]^+^
	311.1641 (10)	311.1647	−0.6	C_20_H_23_O_3_	[M − C_7_H_6_O_2_ − C_8_H_8_O − 2H_2_O + H]^+^
	293.1541 (100)	293.1542	−0.1	C_20_H_21_O_2_	[M − C_7_H_6_O_2_ − C_8_H_8_O − 3H_2_O + H]^+^
	275.1431 (16)	275.1436	−0.5	C_20_H_19_O	[M − C_7_H_6_O_2_ − C_8_H_8_O − 4H_2_O + H]^+^
	265.1591 (16)	265.1592	−0.1	C_19_H_21_O	[M − C_7_H_6_O_2_ − C_8_H_8_O − 3H_2_O − CO + H]^+^
	247.1486 (14)	247.1487	−0.1	C_19_H_19_	[M − C_7_H_6_O_2_ − C_8_H_8_O − 4H_2_O − CO + H]^+^
DPPU_1_ (**19**)	589.2787 (42)	589.2777	+1.0	C_33_H_42_O_8_Na	[M + Na]^+^
	487.2090 (13)	487.2097	−0.7	C_28_H_32_O_6_Na	[M − C_5_H_10_O_2_ + Na]^+^
	453.2275 (68)	453.2253	+2.2	C_25_H_34_O_6_Na	[M − C_8_H_8_O_2_ + Na]^+^
	369.1754 (2)	369.1678	+7.6	C_20_H_26_O_5_Na	[M − C_8_H_8_O_2_ − C_5_H_8_O + Na]^+^
	351.1588 (62)	351.1572	+1.6	C_20_H_24_O_4_Na	[M − C_8_H_8_O_2_ − C_5_H_8_O − H_2_O + Na]^+^
	323.1625 (2)	323.1623	+0.2	C_19_H_24_O_3_Na	[M − C_8_H_8_O_2_ − C_5_H_8_O − H_2_O − CO + Na]^+^
	311.1661 (5)	311.1647	+1.4	C_20_H_23_O_3_	[M − C_8_H_8_O_2_ − C_5_H_8_O − 2H_2_O + H]^+^
	293.1554 (100)	293.1542	+1.2	C_20_H_21_O_2_	[M − C_8_H_8_O_2_ − C_5_H_8_O − 3H_2_O + H]^+^
	275.1462 (6)	275.1436	+2.6	C_20_H_19_O	[M − C_8_H_8_O_2_ − C_5_H_8_O − 4H_2_O + H]^+^
	265.1591 (10)	265.1592	−0.1	C_19_H_21_O	[M − C_8_H_8_O_2_ − C_5_H_8_O − 3H_2_O − CO + H]^+^
	247.1477 (5)	247.1487	−1.0	C_19_H_19_	[M − C_8_H_8_O_2_ − C_5_H_8_O − 4H_2_O − CO + H]^+^

**Table 2:** ^1^H NMR of compounds **3**, **4**, **5** and **6**.

Pos.	3 *	4 *	5 *	6 *
	δ_H_ (*J* in Hz)	δ_H_ (*J* in Hz)	δ_H_ (*J* in Hz)	δ_H_ (*J* in Hz)
1	7.54 *m*	7.52 *m*	7.53 *m*	7.54 *m*
5	2.52 *d* (19.1)	2.53 *^c^*	2.54 *d* (19.0)	2.55 *d* (19.1)
	2.43 *d* (19.1)	2.34 *d* (18.9)	2.34 *d* (19.0)	2.35 *d* (19.1)
7	5.60 *brd* (5.6)	5.65 *dd* (5.8, 2.3)	5.66 *dd* (5.6, 2.2)	5.69 *brd* (5.5)
8β	3.13 *t* (5.6)	3.10 *^d^*	3.11 *^f^*	3.14 *^h^*
10α	3.17 *t* (2.6)	3.12 *^d^*	3.13 *^f^*	3.16 *^h^*
11β	2.05 *m*	2.02 *^e^*	2.02 *^g^*	2.05 *m*
12	2.21 *dd* (14.9, 7.0)	2.12 *dd* (14.8, 7.1)	2.15 *dd* (14.8, 7.0)	2.20 *dd* (15.0, 7.0)
	1.60 *dd* (14.9, 11.1)	1.56 *dd* (14.8, 10.9)	1.57 *dd* (14.8, 10.9)	1.60 *dd* (15.0, 11.0)
14α	1.24 *d* (5.6)	1.17 *d* (5.8)	1.18 *d* (5.7)	1.27 *d* (5.6)
16α	4.35 *d* (11.4)	4.08 *d* (11.3)	4.14 *d* (11.4)	4.35 *d* (11.4)
	4.00 *d* (11.4)	3.86 *d* (11.3)	3.86 *d* (11.4)	4.03 *d* (11.4)
17β	1.22 *s*	1.13 *s*	1.14 *s*	1.22 *s*
18	0.90 *d* (7.0)	0.88 *d* (6.5)	0.88 *d* (6.5)	0.90 *d* (6.6)
19	1.74 *dd* (2.8, 1.4)	1.75 *dd* (2.4, 1.4)	1.74 *dd* (2.9, 1.4)	1.74 *dd* (2.9, 1.4)
20	3.94 *d* (13.4) 3.90 *d* (13.4)	4.46 *s*	4.47 *s*	4.47 *s*
COCH_3_		2.01 *s ^e^*	2.01 *s ^g^*	2.01 *s*
2′	3.61 *s*	3.63 *s*	3.62 *s*	3.62 *s*
4′–8′	7.19–7.14 5H	7.33–7.20 5H	7.32–7.19 5H	7.19–7.12 5H
2″		2.53 *^c^*	-	-
3″	8.00 *^a^*	1.15 *d* (7.0) **	6.84 *m*	8.00 *^i^*
4″	7.48 *^b^*	1.14, *d* (7.0) **	1.81 *s*	7.49 *^j^*
5″	7.62 *tt* (7.4, 1.3)	-	1.80 *d* (6.8)	7.62 *tt* (7.4, 1.3)
6″	7.48 *^b^*	-	-	7.49 *^j^*
7″	8.00 *^a^*	-	-	8.00 *^i^*

* 400 MHz, methanol-d_4_; *^a^*^–*j*^ overlapped signals; ** interchangeable signals.

**Table 3:** ^13^C NMR of compounds **3**, **4**, **5**, **6**, **13**, **14** and **15**.

Position	3 *	4 *	5 *	6 *	13 **	14 *	15 **
	δ_C_	δ_C_	δ_C_	δ_C_	δ_C_	δ_C_	δ_C_
1	160.9	160.6	160.6	160.6	160.8	160.8	161.1
2	134.9 *^a^*	134.96 *^b^*	135.02 *^d^*	135.0	134.9	134.9	134.8
3	210.6	210.2	210.2	210.2	210.3	210.3	210.8
4	74.8	74.5	74.5	74.5	74.6	74.6	74.9
5	38.6	39.0	39.0	39.1	39.1	39.0	42.9
6	142.5	137.5	137.5	137.5	137.2	137.1	138.0
7	129.7 *^†^*	133.4	133.4	133.3	134.4	134.3	129.3
8	39.8	39.6	39.6	39.6	40.3	40.3	40.1
9	77.7	77.6	77.6	77.6	77.6	77.5	77.8
10	57.2	56.9	57.2	57.2	57.2	57.2	57.3
11	37.5	37.4	37.4	37.5	37.5	37.5	37.4
12	33.1	32.9	33.1	33.1	33.1	32.97 *^h^*	33.1
13	65.2	65.1	65.1	65.2	65.0	65.4	65.0
14	31.6	31.5	31.4	31.4	33.1	33.0 *^h^*	33.6
15	28.3	28.4	28.3	28.4	24.2	24.7	24.3
16	71.1	70.2	70.5 *^e^*	71.0	23.5	23.3 *^i^*	23.5
17	11.8	11.7	11.8	11.9	15.8	15.8 *^i^*	15.9
18	18.9	18.9	18.9	18.9	19.0	19.0	19.07
19	10.2	10.2	10.2	10.2	10.2	10.2	10.2
20	68.3	70.5	70.6 *^e^*	70.6	70.7	70.7	25.8
COCH_3_	-	172.7	172.6	172.6	172.6	172.6	-
COCH_3_	-	20.7	20.8	20.8	20.8	20.8	-
1′	175.2	175.2	175.1	175.1	176.9	175.7	180.6
2′	42.4	42.3	42.3	42.4	44.4	41.5	35.4
3′	135.0 *^a^*	134.99 *^b^*	134.94 *^d^*	134.8	27.0	127.1	19.11 *^j^*
4′	130.4	130.5	130.4	130.3	22.8 *^g^*	131.4 *^§^*	19.14 *^j^*
5′	129.7 *^†^*	129.7	129.6	129.6 *^‡^*	22.7 *^g^*	115.0 *^#^*	-
6′	128.4	128.3	128.2	128.2	-	160.6	-
7′	129.7 *^†^*	129.7	129.6	129.6 *^‡^*	-	115.0 *^#^*	-
8′	130.4	130.5	130.4	130.3	-	131.4 *^§^*	-
Ph-OCH_3_	-	-	-	-	-	55.7	-
1″	167.9	178.6	169.5	167.9	-	-	-
2″	131.7	35.2	129.7	131.6	-	-	-
3″	130.7	19.37 *^c^*	138.7	130.6	-	-	-
4″	129.7 *^†^*	19.42 *^c^*	14.4 *^f^*	129.6 *^‡^*	-	-	-
5″	134.3	-	12.2 *^f^*	134.2	-	-	-
6″	129.7 *^†^*	-	-	129.6 *^‡^*			
7″	130.7			130.6			

* 100 MHz, methanol-d_4_; ** 125 MHz, methanol-d_4_; *^†^*^, *‡*, *§*, *#*^ overlapped signals; *^a–j^* interchangeable signals.

**Table 4:** Selected ions of high-energy HRMS^E^ experiment (DIA) [26] of AcDPPI (**4**), AcDPPT (**5**) and AcDPPBz (**6**) (data acquired in ESI positive ionization with a ramp trap collision energy of the high-energy function set at 60–120 eV).

Compound	Observed *m*/*z* (%)	Calculated *m*/*z*	Δm (mDa)	Elemental Composition	Annotation
AcDPPI (**4**)	617.2755 (2)	617.2727	+2.8	C_34_H_42_O_9_Na	[M + Na]^+^
	529.2220 (18)	529.2202	+1.8	C_30_H_34_O_7_Na	[M − C_4_H_8_O_2_ + Na]^+^
	481.2222 (37)	481.2202	+2.0	C_26_H_34_O_7_Na	[M − C_8_H_8_O_2_ + Na]^+^
	421.2007 (100)	421.1991	+1.6	C_24_H_30_O_5_Na	[M − C_8_H_8_O_2_ − C_2_H_4_O + Na]^+^
	411.1807 (11)	411.1784	+2.3	C_22_H_28_O_6_Na	[M − C_4_H_8_O_2_ − C_8_H_6_O + Na]^+^
	393.1724 (18)	393.1678	+4.6	C_22_H_26_O_5_Na	[M − C_4_H_8_O_2_ − C_8_H_6_O − H_2_O + Na]^+^
	333.1476 (26)	333.1467	+0.9	C_20_H_22_O_3_Na	[M − C_4_H_8_O_2_ − C_8_H_6_O − H_2_O − C_2_H_4_O_2_ + Na]^+^
	311.1649 (3)	311.1647	+0.2	C_20_H_23_O_3_	[M − C_8_H_8_O_2_ − C_2_H_4_O − C_4_H_8_O_2_ − H_2_O + H]^+^
	293.1554 (18)	293.1542	+1.2	C_20_H_21_O_2_	[M − C_8_H_8_O_2_ − C_2_H_4_O − C_4_H_8_O_2_ − 2H_2_O + H]^+^
	275.1440 (2)	275.1436	+0.4	C_20_H_19_O	[M − C_8_H_8_O_2_ − C_2_H_4_O − C_4_H_8_O_2_ − 3H_2_O + H]^+^
	265.1599 (4)	265.1592	+0.7	C_19_H_21_O	[M − C_8_H_8_O_2_ − C_2_H_4_O − C_4_H_8_O_2_ − 2H_2_O − CO + H]^+^
	247.1492 (1)	247.1487	+0.5	C_19_H_19_	[M − C_8_H_8_O_2_ − C_2_H_4_O − C_4_H_8_O_2_ − 3H_2_O − CO + H]^+^
AcDPPT (**5**)	629.2745 (4)	629.2727	+1.8	C_35_H_42_O_9_Na	[M + Na]^+^
	529.2225 (41)	529.2202	+2.3	C_30_H_34_O_7_Na	[M − C_5_H_8_O_2_ + Na]^+^
	493.2221 (34)	493.2202	+1.9	C_27_H_34_O_7_Na	[M − C_8_H_8_O_2_ + Na]^+^
	433.2011 (100)	433.1991	+2.0	C_25_H_30_O_5_Na	[M − C_8_H_8_O_2_ − C_2_H_4_O + Na]^+^
	411.1806 (16)	411.1784	+2.2	C_22_H_28_O_6_Na	[M − C_5_H_8_O_2_ − C_8_H_6_O + Na]^+^
	393.1697 (25)	393.1678	+1.9	C_22_H_26_O_5_Na	[M − C_5_H_8_O_2_ − C_8_H_6_O − H_2_O + Na]^+^
	333.1483 (25)	333.1467	+1.6	C_20_H_22_O_3_Na	[M − C_5_H_8_O_2_ − C_8_H_6_O − H_2_O − C_2_H_4_O_2_ + Na]^+^
	311.1662 (6)	311.1647	+1.5	C_20_H_23_O_3_	[M − C_8_H_8_O_2_ − C_2_H_4_O − C_5_H_8_O_2_ − H_2_O + H]^+^
	293.1552 (44)	293.1542	+1.0	C_20_H_21_O_2_	[M − C_8_H_8_O_2_ − C_2_H_4_O − C_5_H_8_O_2_ − 2H_2_O + H]^+^
	275.1446 (5)	275.1436	+1.0	C_20_H_19_O	[M − C_8_H_8_O_2_ − C_2_H_4_O − C_5_H_8_O_2_ − 3H_2_O + H]^+^
	265.1600 (6)	265.1592	+0.8	C_19_H_21_O	[M − C_8_H_8_O_2_ − C_2_H_4_O − C_5_H_8_O_2_ − 2H_2_O − CO + H]^+^
	247.1446 (5)	247.1487	−4.1	C_19_H_19_	[M − C_8_H_8_O_2_ − C_2_H_4_O − C_5_H_8_O_2_ − 3H_2_O − CO + H]^+^
AcDPPBz (**6**)	651.2601 (100)	651.2570	+3.1	C_37_H_40_O_9_Na	[M + Na]^+^
	529.2211 (87)	529.2202	+0.9	C_30_H_34_O_7_Na	[M − C_7_H_6_O_2_ + Na]^+^
	515.2083 (17)	515.2046	+3.7	C_29_H_32_O_7_Na	[M − C_8_H_8_O_2_ + Na]^+^
	455.1880 (34)	455.1834	+4.6	C_27_H_28_O_5_Na	[M − C_8_H_8_O_2_ − C_2_H_4_O + Na]^+^
	411.1787 (52)	411.1784	+0.3	C_22_H_28_O_6_Na	[M − C_7_H_6_O_2_ − C_8_H_6_O + Na]^+^
	393.1679 (25)	393.1678	+0.1	C_22_H_26_O_5_Na	[M − C_7_H_6_O_2_ − C_8_H_6_O − H_2_O + Na]^+^
	333.1474 (24)	333.1467	+0.7	C_20_H_22_O_3_Na	[M − C_7_H_6_O_2_ − C_8_H_6_O − H_2_O − C_2_H_4_O_2_ + Na]^+^
	311.1645 (3)	311.1647	−0.2	C_20_H_23_O_3_	[M − C_8_H_8_O_2_ − C_2_H_4_O − C_7_H_6_O_2_ − H_2_O + H]^+^
	293.1552 (9)	293.1542	+1.0	C_20_H_21_O_2_	[M − C_8_H_8_O_2_ − C_2_H_4_O − C_7_H_6_O_2_ − 2H_2_O + H]^+^
	275.1496 (2)	275.1436	+6.0	C_20_H_19_O	[M − C_8_H_8_O_2_ − C_2_H_4_O − C_7_H_6_O_2_ − 3H_2_O + H]^+^
	265.1512 (5)	265.1592	−8.0	C_19_H_21_O	[M − C_8_H_8_O_2_ − C_2_H_4_O − C_7_H_6_O_2_ − 2H_2_O − CO + H]^+^
	247.1542 (3)	247.1487	+5.5	C_19_H_19_	[M − C_8_H_8_O_2_ − C_2_H_4_O − C_7_H_6_O_2_ − 3H_2_O − CO + H]^+^

**Table 5:** Selected ions of high-energy HRMS^E^ experiment (DIA) [26] of AcDPiPn (**13**) and AcDPMeOP (**14**) (data acquired in ESI positive ionization with a ramp trap collision energy of the high-energy function set at 10–40 eV).

Compound	Observed *m*/*z* (%)	Calculated *m*/*z*	Δm (mDa)	Elemental Composition	Annotation
AcDPiPn (**13**)	497.2527 (100)	497.2515	+1.2	C_27_H_38_O_7_Na	[M + Na]^+^
	395.1843 (17)	395.1834	+0.9	C_22_H_28_O_5_Na	[M − C_5_H_10_O_2_ + Na]^+^
	355.1919 (0.6)	355.1909	+0.1	C_22_H_27_O_4_	[M − C_5_H_10_O_2_ − H_2_O + H]^+^
	335.1631 (34)	335.1623	+0.8	C_20_H_24_O_3_Na	[M − C_5_H_10_O_2_ − AcOH + Na]^+^
	313.1810 (1)	313.1804	+0.6	C_20_H_25_O_3_	[M − C_5_H_10_O_2_ − H_2_O − C_3_H_2_O + H]^+^
	295.1704 (28)	295.1698	+0.6	C_20_H_23_O_2_	[M − C_5_H_10_O_2_ − AcOH − H_2_O + H]^+^
	277.1599 (5)	277.1592	+0.4	C_20_H_21_O	[M − C_5_H_10_O_2_ − AcOH − 2H_2_O + H]^+^
	267.1754 (6)	267.1749	+0.5	C_19_H_23_O	[M − C_5_H_10_O_2_ − AcOH − H_2_O − CO + H]^+^
	249.1653 (0.4)	249.1643	+1.0	C_19_H_21_	[M − C_5_H_10_O_2_ − AcOH − 2H_2_O − CO + H]^+^
AcDPMeOP (**14**)	561.2455 (100)	561.2464	−0.9	C_31_H_38_O_8_Na	[M + Na]^+^
	395.1827 (26)	395.1834	−0.7	C_22_H_28_O_5_Na	[M − C_9_H_10_O_3_ + Na]^+^
	355.1907 (4)	355.1909	−0.2	C_22_H_27_O_4_	[M − C_9_H_10_O_3_ − H_2_O + H]^+^
	335.1617 (22)	335.1623	−0.6	C_20_H_24_O_3_Na	[M − C_9_H_10_O_3_ − AcOH + Na]^+^
	313.1797 (1)	313.1804	−0.7	C_20_H_25_O_3_	[M − C_9_H_10_O_3_ − H_2_O − C_3_H_2_O + H]^+^
	295.1696 (35)	295.1698	−0.2	C_20_H_23_O_2_	[M − C_9_H_10_O_3_ − AcOH − H_2_O + H]^+^
	277.1592 (5)	277.1592	+0.0	C_20_H_21_O	[M − C_9_H_10_O_3_ − AcOH − 2H_2_O + H]^+^
	267.1748 (5)	267.1749	−0.1	C_19_H_23_O	[M − C_9_H_10_O_3_ − AcOH − H_2_O − CO + H]^+^
	249.1643 (0.4)	249.1643	+0.0	C_19_H_21_	[M − C_9_H_10_O_3_ − AcOH − 2H_2_O − CO + H]^+^

**Table 6:** ^1^H NMR of compounds **13**, **14** and **15**.

Pos.	13 *	14 *	15 *
	δ_H_ (*J* in Hz)	δ_H_ (*J* in Hz)	δ_H_ (*J* in Hz)
1	7.54 *m*	7.52 *m*	7.54 *m*
5	2.54 *d* (19.1)	2.54 *d* (19.0)	2.51 *d* (19.1)
	2.37 *d* (19.1)	2.37 *d* (19.0)	2.20 *d* (19.1)
7	5.69 *bd* (5.7)	5.66 *d* (5.6)	5.33 *d* (5.2)
8β	3.08 *brs*	3.08 *t* (5.6)	3.01 *t* (5.7)
10α	3.13 *t* (2.6)	3.13 *t* (2.9)	3.16 *brs*
11β	2.03 *^a^*	2.00 *^c^*	2.00 *m*
12	2.09 *dd* (14.5, 7.0)	2.09 *dd* (14.4, 6.8)	2.09 *dd* (14.3, 6.9)
	1.54 *dd* (14.5, 10.8)	1.52 *dd* (14.4, 10.6)	1.48 *dd* (14.3, 10.5)
14α	0.86 *d* (5.7)	0.86 *d* (5.6)	0.78 *d* (5.6 Hz)
16α	1.18 *s*	1.18 *s*	1.18 *s*
17β	1.07 *s*	1.07 *s*	1.07 *s*
18	0.89 *d* (6.6)	0.90 *d* (6.4)	0.87 *d* (6.2)
19	1.73 *m*	1.74 *dd* (2.9, 1.4)	1.74 *m*
20	4.48 *s*	4.48 *s*	1.73 *m*
COCH_3_	2.02 *s ^a^*	2.02 *s ^c^*	-
2′	2.21 *d* (7.2)	3.56 *s*	2.55 *hept* (7.0)
3′	2.08 *^a^*	-	1.15 *d* (7.0) *^f^*
4′	0.96 *d* (6.9) *^b^*	6.86 *d* (8.4) *^d^*	1.15 *d* (7.0) *^f^*
5′	0.96 *d* (6.9) *^b^*	7.18 *d* (8.4) *^e^*	-
6′	-	-	-
7′	-	7.18 *d* (8.4) *^e^*	-
8′	-	6.86 *d* (8.4) *^d^*	-
9′	-	3.77 (*s*)	-

* 400 MHz, methanol-d_4_; *^a^*^–*f*^ overlapped signals.

**Table 7:** Selected ions of high-energy HRMS^E^ experiment (DIA) [26] of diDPB (**15**) and diDPU_4_ (**22**) (data acquired in ESI positive ionization with a ramp trap collision energy of the high-energy function set at 10–40 eV).

Compound	Observed *m*/*z* (%)	Calculated *m*/*z*	Δm (mDa)	Elemental Composition	Annotation
diDPB (**15**)	425.2322 (100)	425.2304	+1.8	C_24_H_34_O_5_Na	[M + Na]^+^
	337.1794 (40)	337.1780	+1.4	C_20_H_26_O_3_Na	[M − C_4_H_8_O_2_ + Na]^+^
	297.1865 (22)	297.1855	+1.0	C_20_H_25_O_2_	[M − C_4_H_8_O_2_ − H_2_O + H]^+^
	279.1759 (14)	279.1749	+1.0	C_20_H_23_O	[M − C_4_H_8_O_2_ − 2H_2_O + H]^+^
	269.1915 (12)	269.1905	+1.0	C_19_H_25_O	[M − C_4_H_8_O_2_ − H_2_O − CO + H]^+^
diDPU_4_ (**22**)	437.2304 (100)	437.2304	+0.0	C_25_H_34_O_5_Na	[M + Na]^+^
	337.1778 (34)	337.1780	−0.2	C_20_H_26_O_3_Na	[M − C_5_H_8_O_2_ + Na]^+^
	297.1848 (6)	297.1855	−0.7	C_20_H_25_O_2_	[M − C_5_H_8_O_2_ − H_2_O + H]^+^
	279.1745 (13)	279.1749	−0.4	C_20_H_23_O	[M − C_5_H_8_O_2_ − 2H_2_O + H]^+^
	269.1896 (13)	269.1905	−0.9	C_19_H_25_O	[M − C_5_H_8_O_2_ − H_2_O − CO + H]^+^

**Table 8:** 12-Deoxyphorbol esters identified in the UHPLC-HRMS^E^ analysis of chromatographic fractions of *E. Resinifera*. Compounds **1**–**15** were identified as components in a targeted analysis. Compounds **19**–**22** were identified as components in a biased non-targeted analysis [38,39].

Entry	Fraction *	Component	r. t. **	Figure ^†^
1	F	AcDPB (**10**)	4.41–4.52	Figure S31a
2		AcDPA (**11**)	5.05	Figure S31b
3		AcDPP (**12**)	4.62	Figure S31c
4		AcDPiPn (**13**)	5.18	Figure S31d
5	G-6	AcDPPBz (**6**)	5.55	Figure S32a
6		AcDPMeOP (**14**)	4.46	Figure S33a
7		diDPB (**15**)	5.59	Figure S34a
8	G-7	AcDPPI (**4**)	5.20	Figure S35a
9		AcDPPT (**5**)	5.35	Figure S35b
10		AcDPPBz (**6**)	5.53	Figure S35c
11		AcDPP (**12**)	4.57	Figure S36a
12		AcDPMeOP (**14**)	4.39	Figure S36b
13		AcDPPU2 (**20**)	4.54	Figure S35d
14		diDPU4 (**22**)	5.85	Figure S37a
15	G-8	AcDPPI (**4**)	5.18	Figure S38a
16		AcDPPT (**5**)	5.35	Figure S38b
17		AcDPP (**12**)	4.57	Figure S39a
18		AcDPPU3 (**21**)	5.68	Figure S38c
19	G-9	DPPI (**1**)	4.70	Figure S40a
20		DPPT (**2**)	4.89	Figure S40b
21		DPPBz (**3**)	5.04	Figure S40c
22		DPB (**7**)	3.73–3.80	Figure S41a
23		DPA (**8**)	4.39	Figure S41b
24		DPP (**9**)	3.98	Figure S41c
25		DPPU1 (**19**)	5.27	Figure S40d

* Chromatographic fraction from *E. resinifera*; ** r.t. = retention time (min.); ^†^ Figure in Supplementary Material with identification of given 12-deoxyphorbol ester (**1**–**15**; **19**–2**2**) for each entry.

**Table 9:** Selected ions of high-energy HRMS^E^ experiment (DIA) [26] of AcDPPU_2_ (**20**) and AcDPPU_3_ (**21**) (data acquired in ESI positive ionization with a ramp trap collision energy of the high-energy function set at 60–120 eV).

Compound	Observed *m*/*z* (%)	Calculated *m*/*z*	Δm (mDa)	Elemental Composition	Annotation
AcDPPU_2_ (**20**)	603.2540 (4)	603.2570	−3.0	C_33_H_40_O_9_Na	[M + Na]^+^
	529.2205 (29)	529.2202	+0.3	C_30_H_34_O_7_Na	[M − C_3_H_6_O_2_ + Na]^+^
	467.2045 (24)	467.2046	−0.1	C_25_H_32_O_7_Na	[M − C_8_H_8_O_2_ + Na]^+^
	407.1836 (100)	407.1834	+0.2	C_23_H_28_O_5_Na	[M − C_8_H_8_O_2_ − C_2_H_4_O_2_ + Na]^+^
	411.1783 (14)	411.1784	−0.1	C_22_H_28_O_6_Na	[M − C_3_H_6_O_2_ − C_8_H_6_O + Na]^+^
	393.1683 (7)	393.1678	+0.5	C_22_H_26_O_5_Na	[M − C_3_H_6_O_2_ − C_8_H_6_O − H_2_O + Na]^+^
	333.1462 (12)	333.1467	−0.5	C_20_H_22_O_3_Na	[M − C_8_H_8_O_2_ − C_2_H_4_O_2_ − C_3_H_6_O_2_ + Na]^+^
	311.1675 (1)	311.1647	+2.8	C_20_H_23_O_3_	[M − C_8_H_8_O_2_ − C_2_H_4_O − C_3_H_6_O_2_ − H_2_O + H]^+^
	293.1524 (4)	293.1542	−1.8	C_20_H_21_O_2_	[M − C_8_H_8_O_2_ − C_2_H_4_O − C_3_H_6_O_2_ − 2H_2_O + H]^+^
	275.1463 (1)	275.1436	+2.7	C_20_H_19_O	[M − C_8_H_8_O_2_ − C_2_H_4_O − C_3_H_6_O_2_ − 3H_2_O + H]^+^
	265.1610 (1)	265.1592	+1.8	C_19_H_21_O	[M − C_8_H_8_O_2_ − C_2_H_4_O − C_3_H_6_O_2_ − 2H_2_O − CO + H]^+^
AcDPPU_3_ (**21**)	631.2911 (3)	631.2883	+2.8	C_35_H_44_O_9_Na	[M + Na]^+^
	529.2214 (18)	529.2202	+1.2	C_30_H_34_O_7_Na	[M − C_5_H_10_O_2_ + Na]^+^
	495.2373 (33)	495.2359	+1.4	C_27_H_36_O_7_Na	[M − C_8_H_8_O_2_ + Na]^+^
	435.2162 (100)	435.2147	+1.5	C_25_H_32_O_5_Na	[M − C_8_H_8_O_2_ − C_2_H_4_O + Na]^+^
	411.1812 (7)	411.1784	+2.8	C_22_H_28_O_6_Na	[M − C_5_H_10_O_2_ − C_8_H_6_O + Na]^+^
	393.1683 (10)	393.1678	+0.5	C_22_H_26_O_5_Na	[M − C_5_H_10_O_2_ − C_8_H_6_O − H_2_O + Na]^+^
	333.1481 (4)	333.1467	+1.4	C_20_H_22_O_3_Na	[M − C_5_H_10_O_2_ − C_8_H_6_O − H_2_O − C_2_H_4_O_2_ + Na]^+^
	311.1642 (2)	311.1647	−0.5	C_20_H_23_O_3_	[M − C_8_H_8_O_2_ − C_2_H_4_O − C_5_H_10_O_2_ − H_2_O + H]^+^
	293.1559 (13)	293.1542	+1.7	C_20_H_21_O_2_	[M − C_8_H_8_O_2_ − C_2_H_4_O − C_5_H_10_O_2_ − 2H_2_O + H]^+^
	275.1400 (1)	275.1436	−3.6	C_20_H_19_O	[M − C_8_H_8_O_2_ − C_2_H_4_O − C_5_H_10_O_2_ − 3H_2_O + H]^+^
	265.1602 (2)	265.1592	+1.0	C_19_H_21_O	[M − C_8_H_8_O_2_ − C_2_H_4_O − C_5_H_10_O_2_ − 2H_2_O − CO + H]^+^
	247.1508 (1)	247.1487	+2.1	C_19_H_19_	[M − C_8_H_8_O_2_ − C_2_H_4_O − C_5_H_10_O_2_ − 3H_2_O − CO + H]^+^

## Data Availability

Data are contained within the article and Supplementary Materials.

## Supplementary Materials

The following supporting information can be downloaded at https://www.mdpi.com/article/10.3390/plants12223846/s1. ^1^H and ^13^C NMR spectra for compounds **1**–**18**. COSY, HSQC, HMBC spectra for compounds **3**–**6**, **13**–**15**; NOESY2D spectra for compounds **3**–**6**, **13**, **14** and **15**; ^13^C DEPT spectra for compounds **13** and **15**; experimental ECD spectra of compounds **1**–**3**, **5**–**6**; detailed proposed fragmentation routes for selected ions in HRMS^E^ of compounds **1**–**15**, **19**–**22**; HRMS^E^ spectra for compounds **1**–**15**, **19**–**22**; TIC and XIC for selected *m*/*z* used in targeted and biased non-targeted analysis of chromatographic fractions of *E. resinifera*. Tables with experimental data for selected ions from high-energy HRMS^E^ spectra for compounds **1**–**2**, **8**–**9**, **10**–**12**.
