# Supplementary material for: Isolation and Identification of 12-Deoxyphorbol Esters from Euphorbia resinifera Berg Latex: Targeted and Biased Non-Targeted Identification of 12-Deoxyphorbol Esters by UHPLC-HRMSE
Source: Plants (Basel). 2023 Nov 14;12(22):3846. doi: 10.3390/plants12223846 (PMC10674858; doi:10.3390/plants12223846)
Supplement: Supplementary file 1 [file plants-12-03846-s001.zip › plants-2653286-supplementary.pdf]

Isolation and identification of 12-deoxyphorbol esters from *Euphorbia resinifera* Berg latex. Targeted and biased non-targeted identification of 12-deoxyphorbol esters by UHPLC-HRMS^E.

Abdellah Ezzanad[1,2], Carolina De los Reyes[1,2], Antonio J. Macías-Sánchez[1,2,*], Rosario Hernández Galán[1,2,*]

[1] *Departamento de Química Orgánica, Facultad de Ciencias, Campus Universitario Puerto Real, Universidad de Cádiz, 11510 Puerto Real, Cádiz, Spain*

[2] *Instituto de Investigación en Biomoléculas (INBIO), Universidad de Cádiz, 11510 Puerto Real, Cádiz, Spain*

Corresponding Authors

Antonio J. Macías-Sánchez - *Departamento de Química Orgánica, Facultad de Ciencias, Campus Universitario Puerto Real, Universidad de Cádiz, 11510 Puerto Real, Cádiz, Spain; Instituto de Investigación en Biomoléculas (INBIO), Universidad de Cádiz, 11510 Puerto Real, Cádiz, Spain*; https://orcid.org/0000-0001-6002-4977; Tel: +34956012704; E-mail: antoniojose.macias@uca.es

Rosario Hernández Galán - *Departamento de Química Orgánica, Facultad de Ciencias, Campus Universitario Puerto Real, Universidad de Cádiz, 11510 Puerto Real, Cádiz, Spain; Instituto de Investigación en Biomoléculas (INBIO), Universidad de Cádiz, 11510 Puerto Real, Cádiz, Spain*; https://orcid.org/0000-0003-1887-4796; Tel: +34956012771; E-mail: rosario.hernandez@uca.es

Supplementary Material

Table of Contents

1- NMR spectra

Figure S1a. ^{1}H NMR spectrum of DPPI (**1**) in CD_3OD (600 MHz).. 9
Figure S1b. ^{13}C NMR spectrum of DPPI (**1**) in MeOD (150 MHz) 9
Figure S2a. ^{1}H-NMR spectrum DPPT (**2**) in CD_3OD (500 MHz). 10
Figure S2b. ^{13}C-NMR spectrum of DPPT (**2**) in CD_3OD (150 MHz) 10
Figure S3a. ^{1}H-NMR spectrum of DPPBz (**3**) in CD_3OD (400 MHz) 11
Figure S3b. ^{13}C-NMR spectrum of DPPBz (**3**) in CD_3OD (100 MHz) 11
Figure S3c. COSY spectrum of DPPBz (**3**) in CD_3OD. 12
Figure S3d. HSQC spectrum of DPPBz (**3**) in CD_3OD. 12
Figure S3e. Expansion (δ_H 4.7-0.6, δ_C 80-2) of HSQC spectrum of DPPBz (**3**) in CD_3OD. 13
Figure S3f. Expansion (δ_H 8.3-5.4, δ_C 162-120) of HSQC spectrum of **3** in CD_3OD. 13
Figure S3g. HMBC spectrum of DPPBz (**3**) in CD_3OD 14
Figure S3h. Expansion (δ_H 8.9-0.2, δ_C 218-119) of HMBC spectrum of **3** in CD_3OD. 14
Figure S3i. Expansion (δ_H 8.4-0.4, δ_C 82-5) of HMBC spectrum of DPPBz (**3**) in CD_3OD. 15
Figure S3j. NOESY2D spectrum of DPPBz (**3**) in CD_3OD. 15
Figure S4a. ^{1}H-NMR spectrum of AcDPPI (**4**) in CD_3OD. (400 MHz) 16
Figure S4b. ^{13}C-NMR spectrum ofAcDPPI (**4**) in CD_3OD. (100 MHz) 16
Figure S4c. COSY spectrum of AcDPPI (**4**) in CD_3OD. 17
Figure S4d. HSQC spectrum of AcDPPI (**4**) in CD_3OD. 17
Figure S4e Expansion (δ_H 4.7-0.5, δ_C 77-2) of HSQC spectrum of AcDPPI (**4**) in CD_3OD. 18
Figure S4f Expansion (δ_H 4.7-0.5, δ_C 77-2) of HSQC spectrum of AcDPPI (**4**) in CD_3OD. 18
Figure S4g. HMBC spectrum of AcDPPI (**4**) in CD_3OD. 19
Figure S4h. Expansion (δ_H 8.0-0.4, δ_C 91-5) of HMBC spectrum of AcDPPI (**4**) in CD_3OD 19
Figure S4i. Expansion (δ_H 8.0-0.4, δ_C 220-120) of HMBC spectrum of **4** in CD_3OD. 20
Figure S4j. NOESY2D spectrum of AcDPPI (**4**) in CD_3OD.. 20
Figure S5a. ^{1}H NMR spectrum of AcDPPT (**5**) in CD_3OD (400 MHz) 21
Figure S5b. ^{13}C NMR spectrum of AcDPPT (**5**) in CD_3OD (100 MHz). 21
Figure S5c. COSY spectrum of AcDPPT (**5**) in CD_3OD. 22
Figure S5d. HSQC spectrum of AcDPPT (**5**) in CD_3OD. 22
Figure S5e. Expansion (δ_H 4.7-0.5, δ_C 78-4) of HSQC spectrum of AcDPPT (**5**) in CD_3OD. 23
Figure S5f. Expansion (δ_H 7.9-5.0, δ_C 165-120) of HSQC spectrum of **5** in CD_3OD. 23
Figure S5g. HMBC spectrum of AcDPPT (**5**) in CD_3OD. 24
Figure S5h. Expansion (δ_H 8.5-0.5, δ_C 80-2) of HMBC spectrum of **5** in CD_3OD 24
Figure S5i. Expansion (δ_H 8.5-0.5, δ_C 215-120) of HMBC spectrum of **5** in CD_3OD 25
Figure S5j. NOESY2D spectrum of AcDPPT (**5**) in CD_3OD. 25
Figure S6a. ^{1}H-NMR spectrum of AcDPPBz (**6**) in CD_3OD (400 MHz). 26
Figure S6b. ^{13}C-NMR spectrum of AcDPPBz (**6**) in CD_3OD (125 MHz). 26
Figure S6c. COSY spectrum of AcDPPBz (**6**) inCD_3OD. 27

Figure S6d. HSQC spectrum of AcDPPBz (**6**) in CD_3OD. 27
Figure S6e. Expansion (δ_H 4.7-0.6, δ_C 80-4) of HSQC spectrum of **6** in CD_3OD. 28
Figure S6f. Expansion (δ_H 8.5-5.5, δ_C 165-123) of HSQC spectrum of **6** in CD_3OD. 28
Figure S6g. HMBC spectrum of AcDPPBz (**6**) in CD_3OD. 29
Figure S6h. Expansion (δ_H 8.3-0.5, δ_C 80-3) of HMBC spectrum of **6** in CD_3OD. 29
Figure S6i. Expansion (δ_H 9.0-0.5, δ_C 215-120) of HMBC spectrum of **6** in CD_3OD. 30
Figure S6j. NOESY2D spectrum of AcDPPBz (**6**) in CD_3OD. 30
Figure S7a. ^{1}H-NMR spectrum of DPB (**7**) in $CDCl_3$ (400 MHz). 31
Figure S7b. ^{13}C-NMR spectrum of DPB (**7**) in $CDCl_3$ (400 MHz). 31
Figure S8a. ^{1}H-NMR spectrum of DPA (**8**) in $CDCl_3$ (400 MHz). 32
Figure S8b. ^{13}C-NMR spectrum of DPA (**8**) in $CDCl_3$ (100 MHz). 32
Figure S9a. ^{1}H-NMR spectrum of DPP (**9**) in $CDCl_3$ (400 MHz). 33
Figure S9b. ^{13}C NMR spectrum of DPP (**9**) in $CDCl_3$ (100 MHz). 33
Figure S10a. ^{1}H-NMR spectrum of AcDPB (**10**) in CD_3OD (400 MHz). 34
Figure S10b. ^{13}C-NMR spectrum of AcDPB (**10**) in CD_3OD (100 MHz). 34
Figure S11a. ^{1}H-NMR spectrum of AcDPA (**11**) in CD_3OD (400 MHz). 35
Figure S11b. ^{13}C-NMR spectrum of AcDPA (**11**) in CD_3OD (100 MHz). 35
Figure S12a. ^{1}H-NMR spectrum of AcDPP (**12**) in CD_3OD (400 MHz). 36
Figure S12b. ^{13}C-NMR spectrum of AcDPP (**12**) in CD_3OD (100 MHz). 36
Figure S13a. ^{1}H-NMR spectrum of AcDPiPn (**13**) in CD_3OD (500 MHz). 37
Figure S13b. ^{13}C-NMR spectrum of AcDPiPn (**13**) in CD_3OD (125 MHz). 37
Figure S13c. COSY spectrum of AcDPiPn (**13**) in CD_3OD. 38
Figure S13d. HSQC spectrum of AcDPiPn (**13**) in CD_3OD. 38
Figure S13e. Expansion (δ_H 3.8-0.2, δ_C 62-3) of HSQC spectrum of **13** in CD_3OD. 39
Figure S13f. Expansion (δ_H 7.8-4.2, δ_C 170-65) of HSQC spectrum of **13** inCD_3OD. 39
Figure S13g. HMBC spectrum of AcDPiPn (**13**) in CD_3OD. 40
Figure S13h. Expansion (δ_H 8.7-0.2, δ_C 90-3) of HMBC spectrum of **13** in CD_3OD. 40
Figure S13i. Expansion (δ_H 8.7-0.2, δ_C 215-128) of HMBC spectrum of **13** in CD_3OD. 41
Figure S13j. NOESY2D spectrum of AcDPiPn (**13**) in CD_3OD. 41
Figure S13k. DEPT spectrum of AcDPiPn (**13**) in CD_3OD. 42
Figure S14a. ^{1}H-NMR spectrum of AcDPMeOP (**14**) in CD_3OD (400 MHz). 42
Figure S14b. ^{13}C-MNR spectrum of AcDPMeOP (**14**) in CD_3OD (100 MHz). 43
Figure S14c. COSY spectrum of AcDPMeOP (**14**) in CD_3OD.. 43
Figure S14d. HSQC spectrum of AcDPMeOP (**14**) in CD_3OD. 44
Figure S14e. Expansion (δ_H 4.7-0.1, δ_C 77-2) of HSQC spectrum of **14** in CD_3OD. 44
Figure S14f. Expansion (δ_H 7.7-5.5, δ_C 165-110) of HSQC spectrum of **14** in CD_3OD. 45
Figure S14g. HMBC spectrum of AcDPMeOP (**14**) in CD_3OD.. 45
Figure S14h. Expansion (δ_H 7.7-0.5, δ_C 90-3) of HMBC spectrum of **14** in CD_3OD. 46
Figure S14i. Expansion (δ_H 8.0-0.5, δ_C 215-105) of HMBC spectrum of **14** in CD_3OD. 46
Figure S15a. ^{1}H-NMR spectrum of diDPB (**15**) in CD_3OD (500 MHz). 47
Figure S15b. ^{13}C-NMR spectrum of diDPB (**15**) in CD_3OD (125 MHz). 47

Figure S15c. COSY spectrum of diDPB (**15**) in CD_3OD. 48
Figure S15d. HSQC spectrum of diDPB (**15**) in CD_3OD. 48
Figure S15e. Expansion (δ_H 3.6-0.4, δ_C 65-5) of HSQC spectrum of diDPB (**15**) in CD_3OD...49
Figure S15f. HMBC spectrum of diDPB (**15**) in CD_3OD. 49
Figure S15g. Expansion (δ_H 3.6-0.4, δ_C 65-5) of HMBC spectrum of **15** in CD_3OD.........50
Figure S15h. Expansion (δ_H 8.0-0.3, δ_C 210-120) of HMBC spectrum of **15** in CD_3OD........50
Figure S15i. NOESY2D spectrum of diDPB (**15**) in CD_3OD. 51
Figure S15j. ^{13}C-DEPT spectrum of diDPB (**15**) in CD_3OD. 51
Figure S16a. ^{1}H NMR spectrum of RTX (**16**) in $CDCl_3$ (400 MHz). 52
Figure S16b. ^{13}C NMR spectrum of RTX (**16**) in $CDCl_3$ (100 MHz). 52
Figure S17a. ^{1}H-NMR spectrum of EOF1 (**17**) in CD_3OD (400 MHz). 53
Figure S17b. ^{13}C-NMR spectrum of EOF1 (**17**) in CD_3OD. (100 MHz). 53
Figure S18a. ^{1}H-NMR spectrum of EOF2 (**18**) in CD_3OD (400 MHz). 54
Figure S18b. ^{13}C-NMR spectrum of EOF2 (**18**) in CD_3OD (100 MHz). 54

2- ECD spectra.

Figure S19. Experimental ECD spectra of DPPI (**1**), DPPT (**2**), DPPBz (**3**), AcDPPT (**5**) and AcDPPBz (**6**)........ 55

3- UHPLC-HRMS^{E} data. Figures.

Figure S20. Proposed fragmentation route for selected ions on HRMS^{E} spectrum (Data Independent Acquisition (DIA)) [1], in ESI positive mode, for DPPI (**1**), DPPT (**2**), DPPBz (**3**) and DPPU$_1$ (**19**). In red, common daughter ions with group B compounds (see section 2.2 and Figure S22)........ 56
Figure S21. Comparison of HRMS^{E} spectra (DIA) [1] for (**a**) DPPT (**2**), (**b**) AcDPPI (**4**), (**c**) AcDPPT (**5**), (**d**) AcDPPBz (**6**), (**e**) AcDPPU$_2$ (**20**) and (**f**) AcDPPU$_3$ (**21**), m/z range 240-680 (data acquired in ESI positive mode with a ramp trap collision energy of the high-energy function set at 60-120 eV).)........ 57
Figure S22. Proposed fragmentation route for selected ions on HRMS^{E} spectrum (DIA) [1], in ESI positive mode, for AcDPPI (**4**), AcDPPT (**5**), AcDPPBz (**6**), AcDPPU$_2$ (**20**) and AcDPPU$_3$ (**21**). In red, common daughter ions with group A compounds (see section 2.2 and Figure S20)........ 58
Figure S23. Comparison of HRMS^{E} spectra (DIA) [1] for (**a**) DPB (**7**), (**b**) DPA (**8**) and (**c**) DPP (**9**), m/z range 240-540 (data acquired in ESI positive mode with a ramp trap collision energy of the high-energy function set at 10-40 eV)........ 59
Figure S24. Comparison of HRMS^{E} spectra (DIA) [1] for: (a) DPB (7), (b) DPA (8) and, (c) DPP (9) X23); m/z range 240-365 (m/z range 240-540 in Figure S23) (data acquired in positive ionization with a ramp trap collision energy of the high-energy function set at 10-40 eV). Compounds **7**, **8** and **9** show daughter ions from their corresponding $[M+Na]^+$ molecular ions (see Figure S23) in their HRMS^{E} spectra at m/z 313.1804, 295.1698, 277.1592, 267.1749 and 249.1643 (calculated) (in green in Scheme 3, Figures S24 and S25) which could be assigned to losses of water (1, 2 and 3 molecules), 2 molecules of water and CO and 3 molecules of water

and CO, respectively, from a precursor ion at m/z 353.1729 (calculated) (in red in Scheme 3, Figures S24 and S25) (Table S2). Last mentioned ion, which is relatively abundant in HRMS^E spectra of DPB (**7**) DPA (**8**) and DPP (**9**) (Table S2, Figures S23 and S23a) originates from a loss of an ester group at C-13 from parent molecular ions in each compound (Figure S23)……..…… 60

Figure S25. Proposed fragmentation route for selected ions on HRMS^E spectrum (DIA) [1], in ESI positive mode, for DPB (**7**), DPA (**8**) and DPP (**9**). In green, common daughter ions with group D compounds (see section 2.2 and Figure S29)…..……………………………..…… 61

Figure S26. Comparison of HRMS^E spectra (DIA) [1], for (**a**) AcDPB (**10**), (**b**) AcDPA (**11**), (**c**) and AcDPP (**12**), m/z range 240-560 (data acquired in ESI positive mode with a ramp trap collision energy of the high-energy function set at 10-40 eV). ……………………………… 62

Figure S27. Comparison of HRMS^E spectra (DIA) [1] for (**a**) AcDPB (**10**), (**b**) AcDPA (**11**), (**c**) and AcDPP (**12**), m/z range 240-410 (data acquired in ESI positive mode with a ramp trap collision energy of the high-energy function set at 10-40 eV). ……………………………… 63

Figure S28. Comparison of HRMS^E spectra (DIA) [1] for (**a**) AcDPiPn (**13**) and (**b**) AcDPMeOP (**14**), m/z range 240-600 (m/z range 240-410 in **Figure 7**) (data acquired in in ESI positive mode with a ramp trap collision energy of the high-energy function set at 10-40 eV). ………………………………………………………………………………………..…… 64

Figure S29. Proposed fragmentation route for selected ions on HRMS^E spectrum (DIA) [1], in ESI positive mode, for AcDPB (**10**), AcDPA (**11**), AcDPP (**12**), AcDPiPn (**13**) and AcDPMeOP (**14**). In green, common daughter ions with group C compounds (see section 2.2 and Figure S25)…………………………………………..……………………………….…. 65

Figure S30. Proposed fragmentation route for selected ions on HRMS^E spectrum (DIA) [1], in ESI positive mode, for diDPB (**15**) and diDPU$_4$ (**22**)……………………………………… 66

Figure S31. Total Ion Current (TIC) and eXtracted Ion Chromatograms (XICs), at selected *m/z* previously observed in high energy HRMS^E of 12-deoxyphorbol 20-acetate-13-acyl derivatives, obtained from UHPLC-HRMS^E experiments for chromatographic fraction F of *E. resinifera* (data acquired in ESI positive mode with a ramp trap collision energy of the high-energy function set at 10-40 eV; high-energy function (2: TOF MS ES+)). XICs for 12-deoxyphorbol 20-acetate-13-acyl derivatives [M+Na]$^+$ molecular ions: *m/z* 483.2359 calculated mass for (**a**) $C_{26}H_{36}O_7Na$ (AcDPB (**10**)); (**b**) *m/z* 495.2359 calculated mass for $C_{27}H_{36}O_7Na$ (AcDPA (**11**)); (**c**) *m/z* 531.236, calculated mass for $C_{30}H_{36}O_7Na$ (AcDPP (**12**)) and (**d**) *m/z* 497.2515, calculated mass for $C_{27}H_{38}O_7Na$ (AcDPiPn (**13**)). XICs for selected featured ions of 12-deoxyphorbol 20-acetate-13-acyl derivatives HRMS^E spectra (see Scheme 4 and Figure S29) at: (**e**) *m/z* 395.1834; (**f**) *m/z* 335.1623; (**g**) *m/z* 295.1698. (**h**) UHPLC-HRMS^E chromatographic profile (TIC) of fraction F from *E. resinifera* extract…...……………………………….……… 67

Figure S32. Total Ion Current (TIC) and eXtracted Ion Chromatograms (XICs), at selected m/z previously observed in high energy HRMS^E of 12-deoxy-16-hydroxyphorbol 20-acetate-13,16-diacyl derivatives, obtained from UHPLC-HRMS^E experiments for chromatographic fraction G-6 of *E. resinifera* (data acquired in ESI positive mode with a ramp trap collision energy of the high-energy function set at 60-120 eV; high-energy function (2: TOF MS ES$^+$)). XIC for 12-deoxy-16-hydroxyphorbol 20-acetate-16-benzoate-13-phenylacetate (AcDPPBz (**6**)) [M+Na]$^+$ molecular ion: (**a**) *m/z* 651.2565, calculated mass for $C_{37}H_{40}O_9Na$. XICs for selected characteristic ions of 16-hydroxy-12-deoxyphorbol 20-acetate-13,16-diacyl derivatives HRMS^E spectra (see Scheme 2 and Figure S22) at (**b**) *m/z* 529.2202, (**c**) *m/z* 411.1784, (**d**) *m/z* 393.1678, (**e**) *m/z* 333.1467 and *m/z* (**f**) 293.1542. (**g**) TIC of UHPLC-HRMS^E chromatographic profile of fraction G-6 from *E. resinifera* extract……..............………………………………………….……… 68

Figure S33. Total Ion Current (TIC) and eXtracted Ion Chromatograms (XICs), at selected *m/z* previously observed in high energy HRMS^E of 12-deoxyphorbol 20-acetate-13-acyl derivatives, obtained from UHPLC-HRMS^E experiments for chromatographic fraction G-6 of *E. resinifera* (data acquired in ESI positive mode with a ramp trap collision energy of the high-energy function set at 10-40 eV; high-energy function (2: TOF MS ES+)). XIC for 12-deoxyphorbol 20-acetate-13-(*p*-methoxyphenyl)acetate (AcDPMeOP (**14**)) $[M+Na]^+$ molecular ion: (**a**) *m/z* 561.2464 calculated mass for $C_{31}H_{38}O_8Na$. XICs for selected featured ions of 12-deoxyphorbol 20-acetate-13-acyl derivatives HRMS^E spectra (see Scheme 4 and Figure S29) at: (**b**) *m/z* 395.1834; (**c**) *m/z* 335.1623; (**d**) *m/z* 295.1698. (**e**) UHPLC-HRMS^E chromatographic profile (TIC) of fraction G-6 from *E. resinifera* extract………………………………..…………………………….……… 69

Figure S34. Total Ion Current (TIC) and eXtracted Ion Chromatograms (XICs), at selected *m/z* previously observed in high energy HRMS^E of 12,20-dideoxyphorbol 13-acyl derivatives, obtained from UHPLC-HRMS^E experiments for chromatographic fraction G-6 of *E. resinifera* (data acquired in ESI positive mode with a ramp trap collision energy of the high-energy function set at 10-40 eV; high-energy function (2: TOF MS ES+)). XIC for 12,20-dideoxyphorbol 13-isobutyrate (diDPB (**15**)) $[M+Na]^+$ molecular ion: (**a**) *m/z* 425.2304 calculated mass for $C_{24}H_{34}O_5Na$. XICs for selected featured ions of 12,20-dideoxyphorbol 13-acyl derivatives HRMS^E spectra (see Scheme 5 and Figure S30) at: (**b**) *m/z* 337.1780; (**c**) *m/z* 297.1855; (**d**) *m/z* 279.1749 and (**e**) *m/z* 269.1905. (**f**) UHPLC-HRMS^E chromatographic profile (TIC) of fraction G-6 from *E. resinifera* extract……………………..……………………………………… 70

Figure S35. Total Ion Current (TIC) and eXtracted Ion Chromatograms (XICs), at selected m/z previously observed in high energy HRMS^E of 12-deoxy-16-hydroxyphorbol 20-acetate-13,16-diacyl derivatives, obtained from UHPLC-HRMS^E experiments for chromatographic fraction G-7 of *E. resinifera* (data acquired in ESI positive mode with a ramp trap collision energy of the high-energy function set at 60-120 eV; high-energy function (2: TOF MS ES$^+$)). XIC for 12-deoxy-16-hydroxyphorbol 20-acetate-13,16-diacyl derivatives $[M+Na]^+$ molecular ions: (**a**) *m/z* 617.2727, calculated mass for $C_{34}H_{42}O_9Na$ (AcDPPI (**4**)); (**b**) *m/z* 629.2727, calculated mass for $C_{35}H_{42}O_9Na$ (AcDPPT (**5**)); (**c**) *m/z* 651.2565, calculated mass for $C_{37}H_{40}O_9Na$ (AcDPPBz (**6**)); (**d**) *m/z* 603.2570, calculated mass for $C_{33}H_{40}O_9Na$ (AcDPPU$_2$ (**20**)). XICs for selected characteristic ions of 16-hydroxy-12-deoxyphorbol 20-acetate-13,16-diacyl derivatives HRMS^E spectra (see Scheme 2 and Figure S22) at (**e**) *m/z* 529.2202, (**f**) *m/z* 411.1784, (**g**) *m/z* 393.1678, (**h**) *m/z* 333.1467 and (**i**) *m/z* 293.1542. (**j**) TIC of UHPLC-HRMS^E chromatographic profile of fraction G-7 from *E. resinifera* extract……………………………...……………………… 71

Figure S36. Total Ion Current (TIC) and eXtracted Ion Chromatograms (XICs), at selected *m/z* previously observed in high energy HRMS^E of 12-deoxyphorbol 20-acetate-13-acyl derivatives, obtained from UHPLC-HRMS^E experiments for chromatographic fraction G-7 of *E. resinifera* (data acquired in ESI positive mode with a ramp trap collision energy of the high-energy function set at 10-40 eV; high-energy function (2: TOF MS ES+)). XICs for 12-deoxyphorbol 20-acetate-13-acyl derivatives $[M+Na]^+$ molecular ions: (**a**) *m/z* 531.236, calculated mass for $C_{30}H_{36}O_7Na$ (AcDPP (**12**)); (**b**) *m/z* 561.2464 calculated mass for $C_{31}H_{38}O_8Na$ (AcDPMeOP (**14**)). XICs for selected featured ions of 12-deoxyphorbol 20-acetate-13-acyl derivatives HRMS^E spectra (see Scheme 4 and Figure S29) at: (**c**) *m/z* 395.1834; (**d**) *m/z* 335.1623; (**e**) *m/z* 295.1698. (**f**) UHPLC-HRMS^E chromatographic profile (TIC) of fraction G-7 from *E. resinifera* extract…………………..……………………………………………………………….……… 72

Figure S37. Total Ion Current (TIC) and eXtracted Ion Chromatograms (XICs), at selected *m/z* previously observed in high energy HRMS^E of 12,20-dideoxyphorbol 13-acyl derivatives, obtained from UHPLC-HRMS^E experiments for chromatographic fraction G-7 of *E. resinifera* (data acquired in ESI positive mode with a ramp trap collision energy of the high-energy function set at 10-40 eV; high-energy function (2: TOF MS ES+)). XIC for 12,20-dideoxyphorbol 13-acyl derivatives [M+Na]$^+$ molecular ion: (**a**) *m/z* 437.2304 calculated mass for $C_{25}H_{34}O_5Na$ (diDPU$_4$ (**22**)). XICs for selected featured ions of 12,20-dideoxyphorbol 13-acyl derivatives HRMS^E spectra (see Scheme 5 and Figure S30) at: (**b**) *m/z* 337.1780; (**c**) *m/z* 297.1855; (**d**) *m/z* 279.1749 and (**e**) *m/z* 269.1905. (**f**) UHPLC-HRMS^E chromatographic profile (TIC) of fraction G-7 2019-Ac-FR11 from *E. resinifera* extract………………………………..………………………… 73

Figure S38. Total Ion Current (TIC) and eXtracted Ion Chromatograms (XICs), at selected m/z previously observed in high energy HRMS^E of 12-deoxy-16-hydroxyphorbol 20-acetate-13,16-diacyl derivatives, obtained from UHPLC-HRMS^E experiments for chromatographic fraction G-8 of *E. resinifera* (data acquired in ESI positive mode with a ramp trap collision energy of the high-energy function set at 60-120 eV; high-energy function (2: TOF MS ES$^+$)). XIC for 12-deoxy-16-hydroxyphorbol 20-acetate-13,16-diacyl derivatives [M+Na]$^+$ molecular ions: (**a**) *m/z* 617.2727, calculated mass for $C_{34}H_{42}O_9Na$ (AcDPPI (**4**)); (**b**) *m/z* 629.2727, calculated mass for $C_{35}H_{42}O_9Na$ (AcDPPT (**5**)); (**c**) *m/z* 631.2883, calculated mass for $C_{35}H_{44}O_9Na$ (AcDPPU$_3$ (**21**)). XICs for selected characteristic ions of 16-hydroxy-12-deoxyphorbol 20-acetate-13,16-diacyl derivatives HRMS^E spectra (see Scheme 2 and Figure S22) at (**d**) *m/z* 529.2202, (**e**) *m/z* 411.1784, (**f**) *m/z* 393.1678, (**g**) *m/z* 333.1467 and (**h**) *m/z* 293.1542. (**i**) TIC of UHPLC-HRMS^E chromatographic profile of fraction G-8 from *E. resinifera* extract……………………………………………………………..………………………..… 74

Figure S39. Total Ion Current (TIC) and eXtracted Ion Chromatograms (XICs), at selected *m/z* previously observed in high energy HRMS^E of 12-deoxyphorbol 20-acetate-13-acyl derivatives, obtained from UHPLC-HRMS^E experiments for chromatographic fraction G-8 of *E. resinifera* (data acquired in ESI positive mode with a ramp trap collision energy of the high-energy function set at 10-40 eV; high-energy function (2: TOF MS ES+)). XIC for 12-deoxyphorbol 20-acetate-13-phenylacetate (AcDPP (**12**)) [M+Na]$^+$ molecular ion: (**a**) *m/z* 531.2359 calculated mass for $C_{30}H_{36}O_7Na$. XICs for selected featured ions of 12-deoxyphorbol 20-acetate-13-acyl derivatives HRMS^E spectra (see Scheme 4 and Figure S29) at: (**b**) *m/z* 395.1834; (**c**) *m/z* 335.1623; (**d**) *m/z* 295.1698. (**e**) UHPLC-HRMS^E chromatographic profile (TIC) of fraction G-8 from *E. resinifera* extract………………...……………………………………………………………...……… 75

Figure S40. Total Ion Current (TIC) and eXtracted Ion Chromatograms (XICs), at selected m/z previously observed in high energy HRMS^E of 12-deoxy-16-hydroxyphorbol 13,16-diacyl derivatives, obtained from UHPLC-HRMS^E experiments for chromatographic fraction G-9 of *E. resinifera* (data acquired in ESI positive mode with a ramp trap collision energy of the high-energy function set at 60-120 eV; high-energy function (2: TOF MS ES$^+$)). XIC for 12-deoxy-16-hydroxyphorbol 13,16-diacyl derivatives [M+Na]$^+$ molecular ions: (**a**) *m/z* 575.2621, calculated mass for $C_{32}H_{40}O_8Na$ (DPPI (**1**)); (**b**) *m/z* 587.2621, calculated mass for $C_{33}H_{40}O_8Na$ (DPPT (**2**)); (**c**) *m/z* 609.2464, calculated mass for $C_{35}H_{38}O_8Na$ (DPPBz (**3**)); (**d**) *m/z* 589.2777, calculated mass for $C_{33}H_{42}O_8Na$ (DPPU$_1$ (**19**)). XICs for selected characteristic ions of 12-deoxy-16-hydroxyphorbol 13,16-diacyl derivatives HRMS^E spectra (see Scheme 1 and Figure S20) at (**e**) *m/z* 487.2097, (**f**) *m/z* 369.1678, (**g**) *m/z* 351.1572, (**h**) *m/z* 323.1623 and (**i**) *m/z* 293.1542. (**j**) TIC of UHPLC-HRMS^E chromatographic profile of fraction G-9 from *E. resinifera* extract…..…..…………………………..…………………………………………….……… 76

Figure S41. Total Ion Current (TIC) and eXtracted Ion Chromatograms (XICs), at selected *m/z* previously observed in high energy HRMS^E of 12-deoxyphorbol 13-acyl derivatives, obtained from UHPLC-HRMS^E experiments for chromatographic fraction G-9 of *E. resinifera* (data acquired in ESI positive mode with a ramp trap collision energy of the high-energy function set at 10-40 eV; high-energy function (2: TOF MS ES+)). XICs for 12-deoxyphorbol 13-acyl derivatives $[M+Na]^+$ molecular ions: (**a**) *m/z* 441.2253 calculated mass for $C_{24}H_{34}O_6Na$ (DPB (**7**)); (**b**) *m/z* 453.2253 calculated mass for $C_{25}H_{34}O_6Na$ (DPA (**8**)) and (**c**) *m/z* 489.2253, calculated mass for $C_{28}H_{34}O_6Na$ (DPP (**9**)). XICs for selected featured ions of 12-deoxyphorbol 13-acyl derivatives HRMS^E spectra (see Scheme 3 and Figure S25) at: (**d**) *m/z* 395.1834; (**e**) *m/z* 335.1623; (**f**) *m/z* 295.1698. (**g**) UHPLC-HRMS^E chromatographic profile (TIC) of fraction from *E. resinifera* extract.. 77

4- UHPLC-HRMS^E data. Tables.

Table S1. Selected ions of high energy HRMS^E experiment (DIA) [1] of DPPI (**1**) and DPPT (**2**) (data acquired in ESI positive ionization with a ramp trap collision energy of the high-energy function set at 60-120 eV). 78

Table S2. Selected ions of high energy HRMS^E experiment (DIA) [1] of DPA (**8**) and DPP (**9**) (data acquired in ESI positive ionization with a ramp trap collision energy of the high-energy function set at 10-40 eV). 79

Table S3. Selected ions of high energy HRMS^E experiment (DIA) [1] of AcDPB (**10**), AcDPA (**11**) and AcDPP (**12**) (data acquired in ESI positive ionization with a ramp trap collision energy of the high-energy function set at 10-40 eV). 80

References. 81

1- NMR Spectra

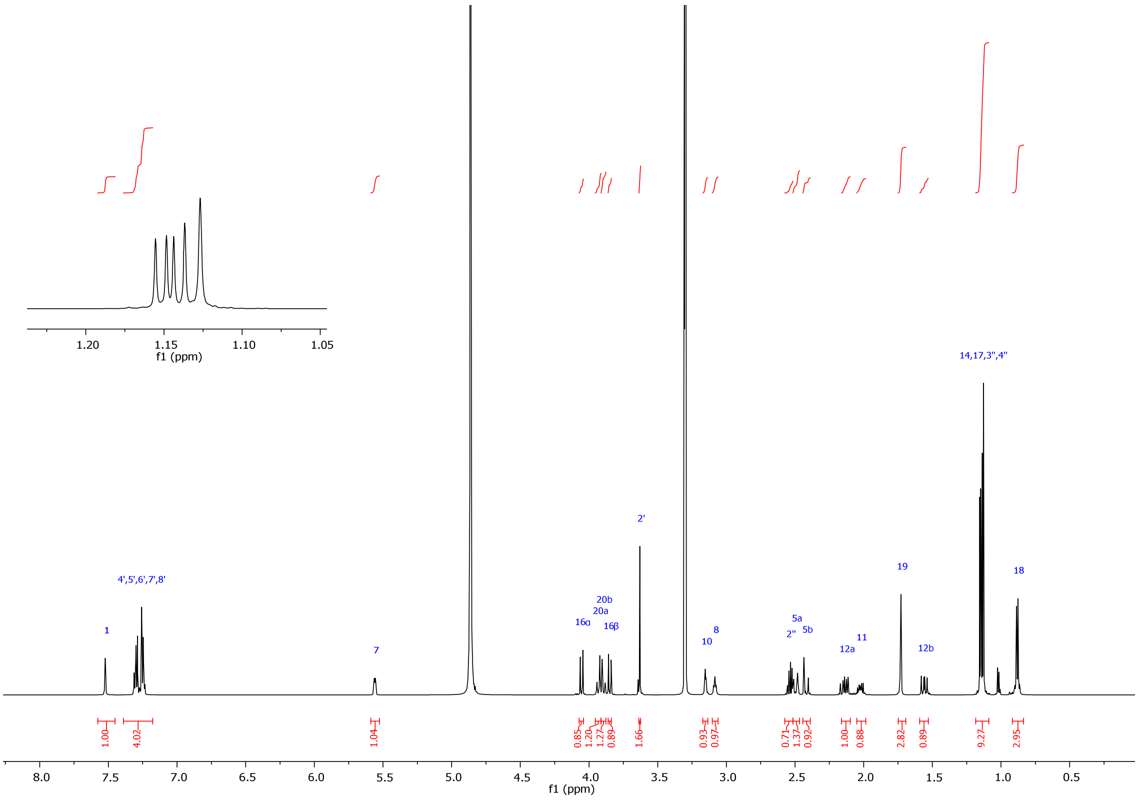

Figure S1a. ^{1}H NMR spectrum of DPPI (**1**) in CD_3OD (600 MHz).

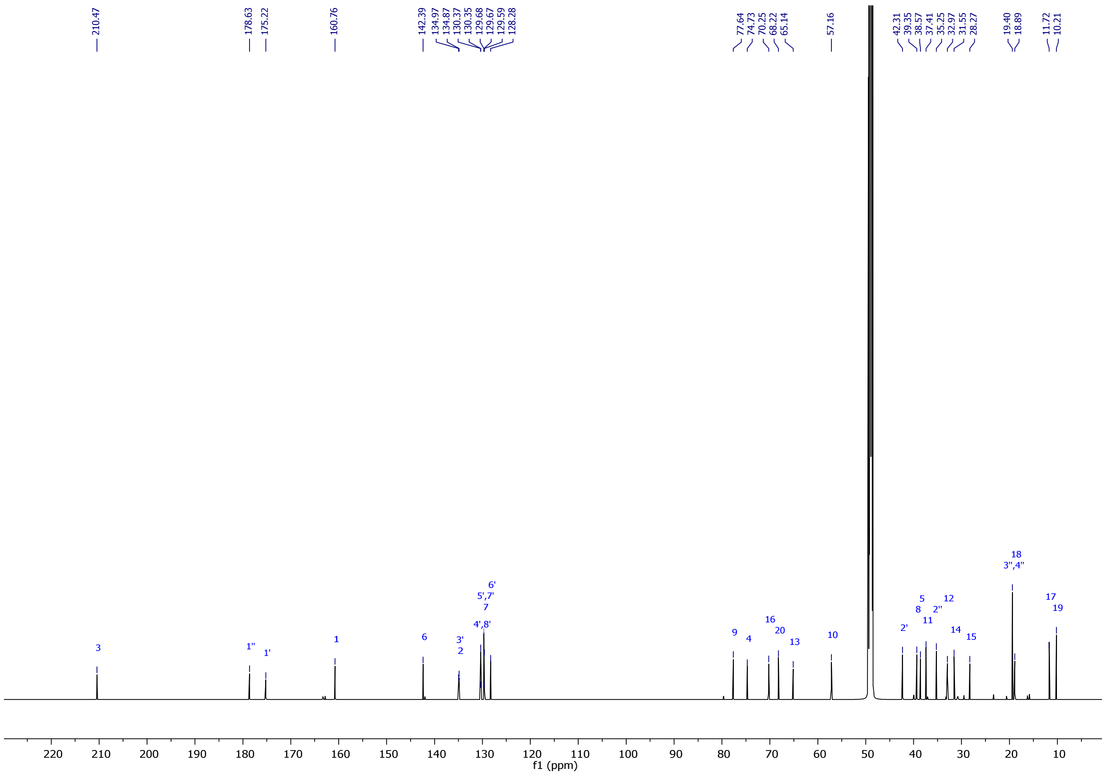

Figure S1b. ^{13}C NMR spectrum of DPPI (**1**) in MeOD (150 MHz)

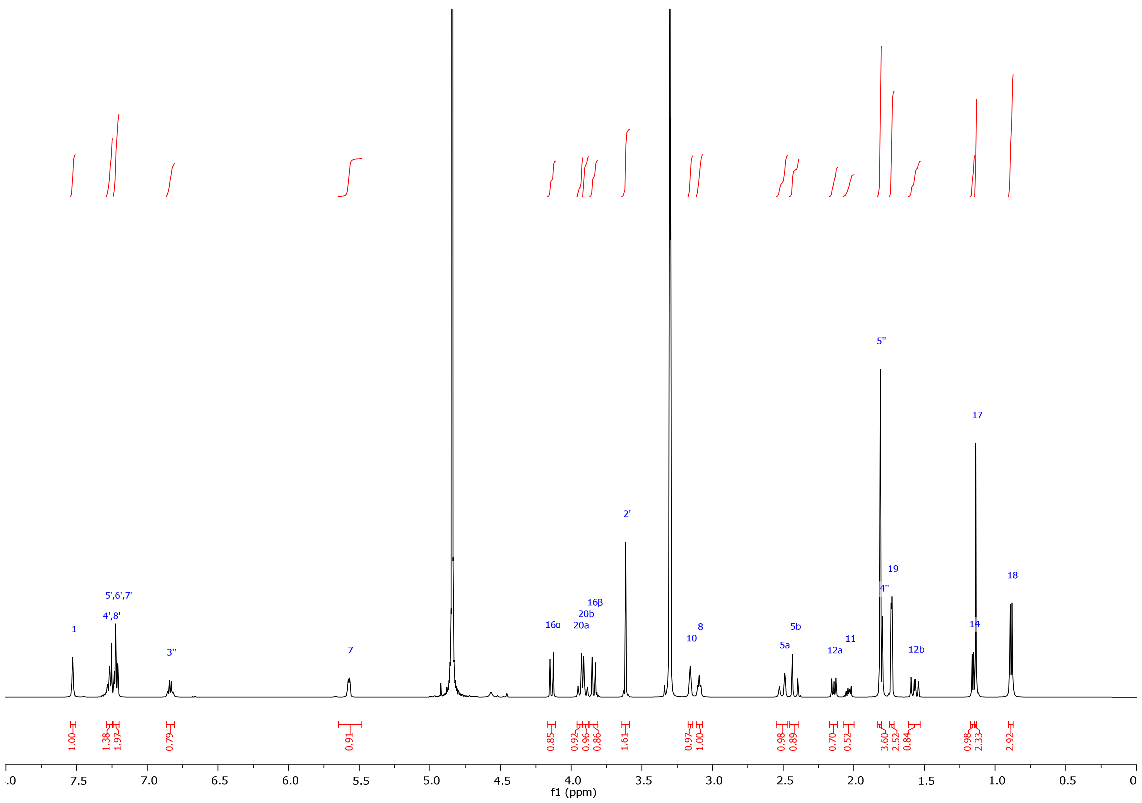

Figure S2a. ^{1}H-NMR spectrum DPPT (**2**) in CD_3OD (500 MHz).

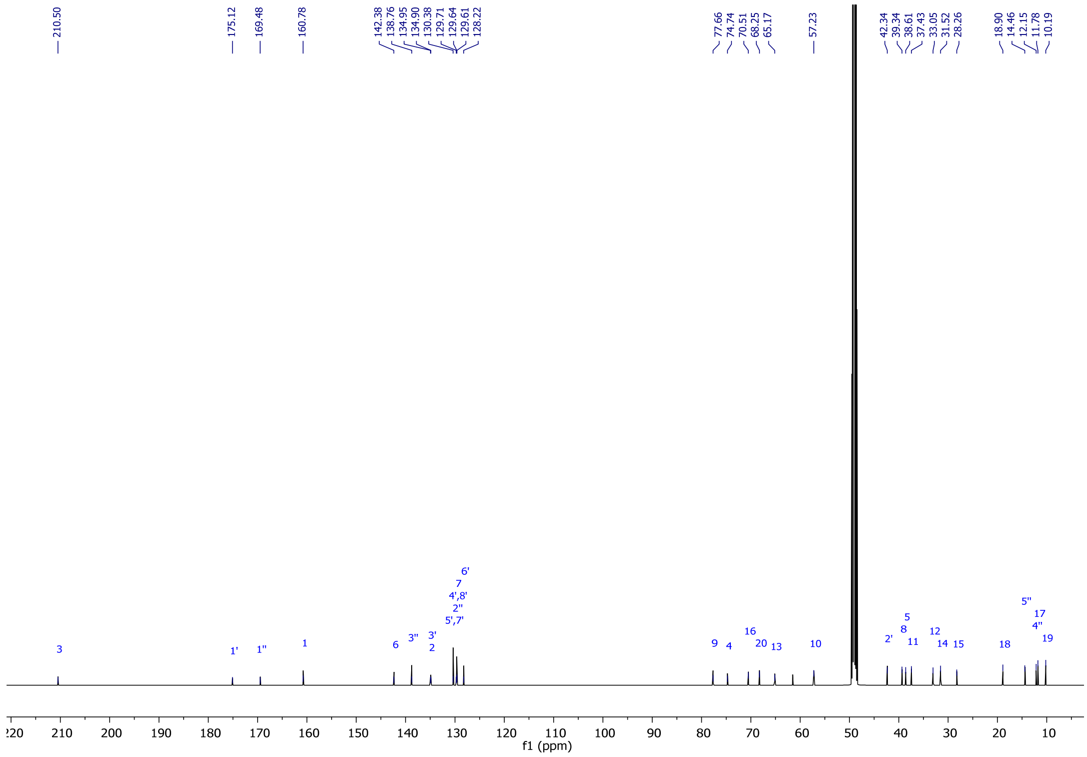

Figure S2b. ^{13}C-NMR spectrum of DPPT (**2**) in CD_3OD (150 MHz).

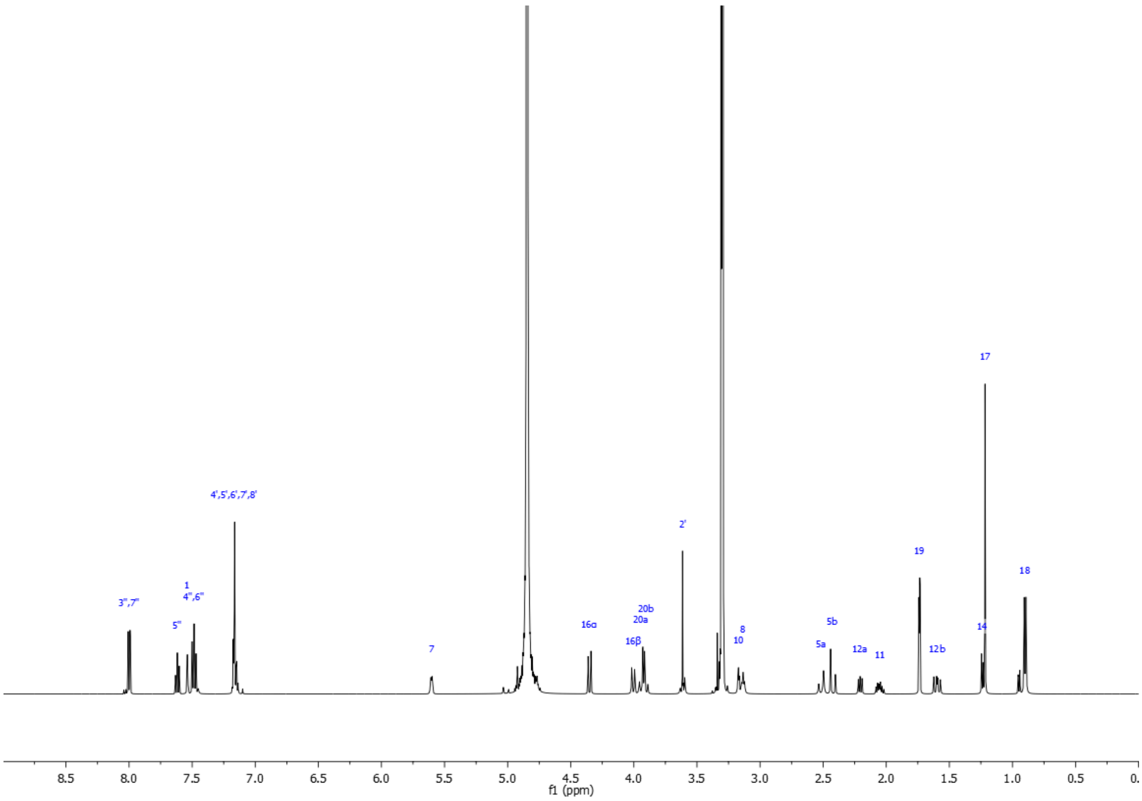

Figure S3a. ^{1}H-NMR spectrum of DPPBz (**3**) in CD_3OD (400 MHz).

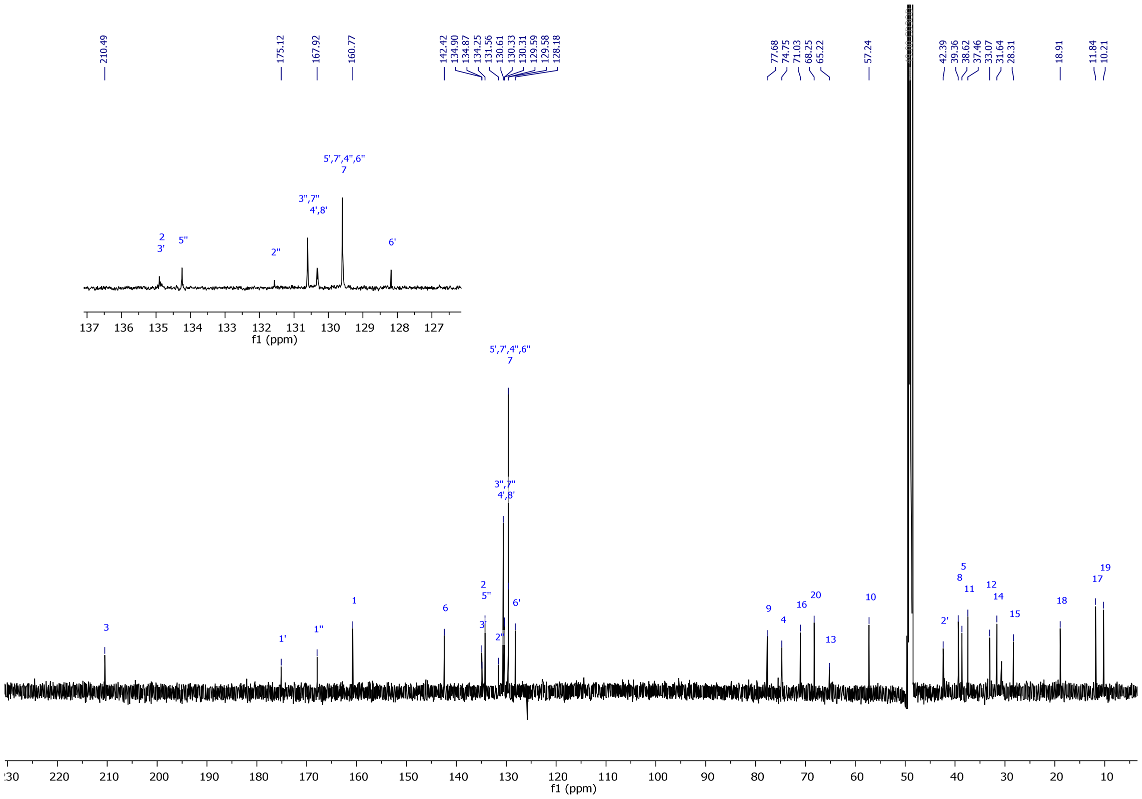

Figure S3b. ^{13}C-NMR spectrum of DPPBz (**3**) in CD_3OD (100 MHz).

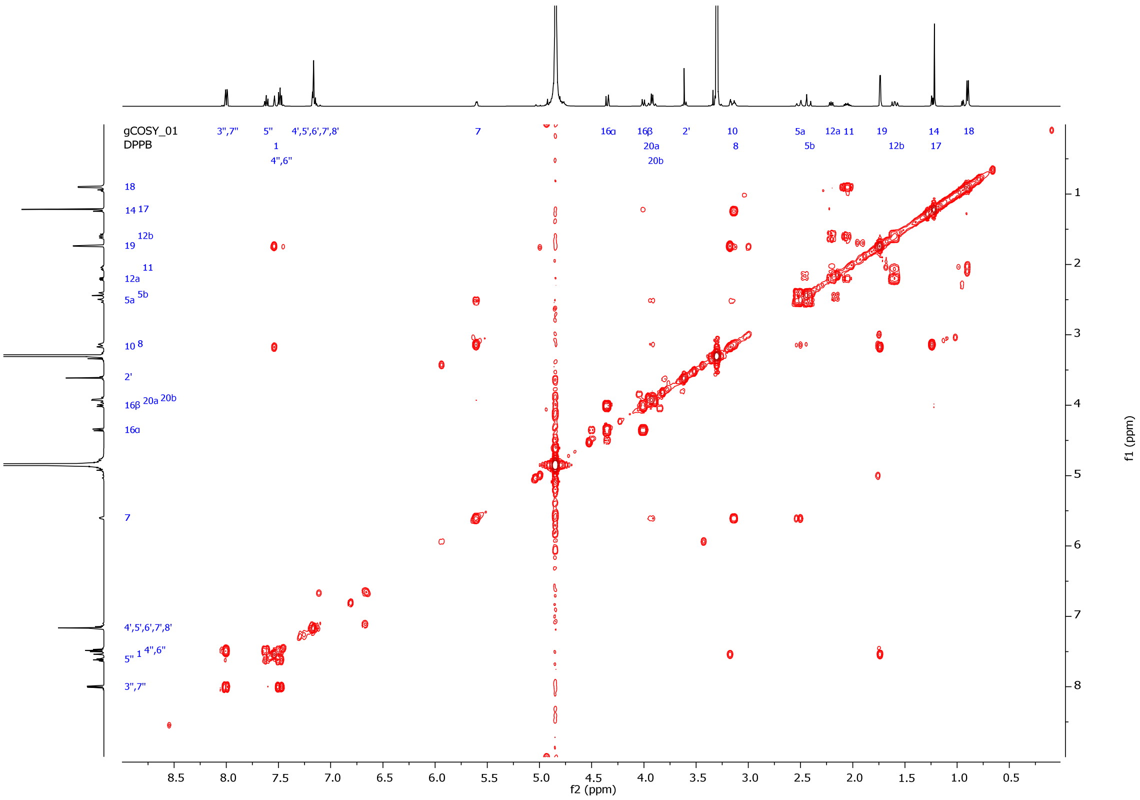

Figure S3c. COSY spectrum of DPPBz (**3**) in CD_3OD.

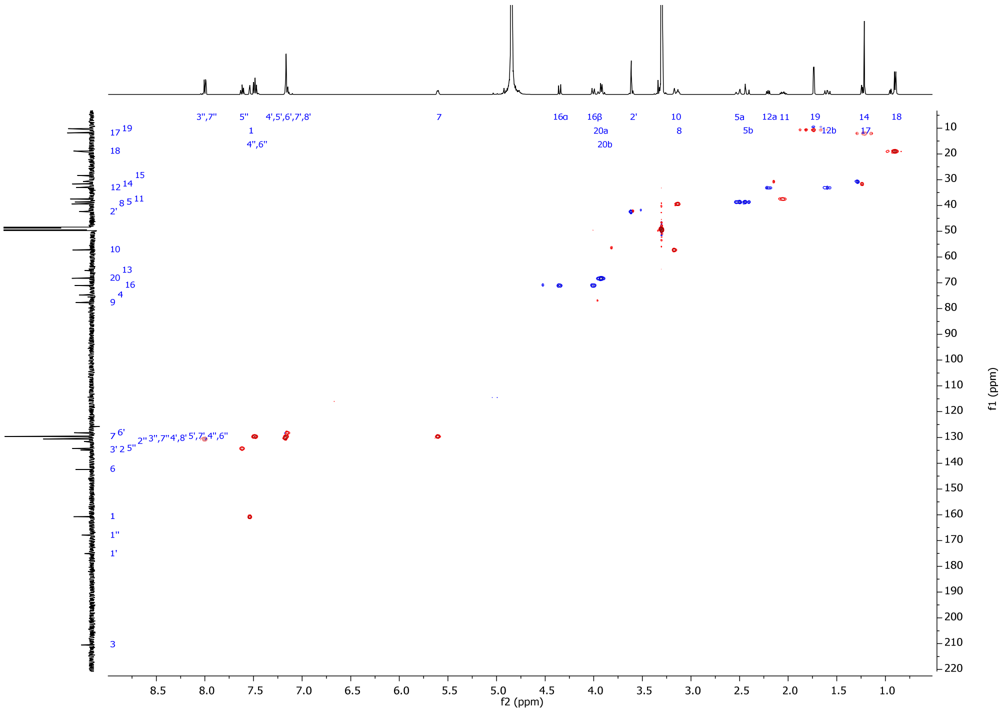

Figure S3d. HSQC spectrum of DPPBz (**3**) in CD_3OD.

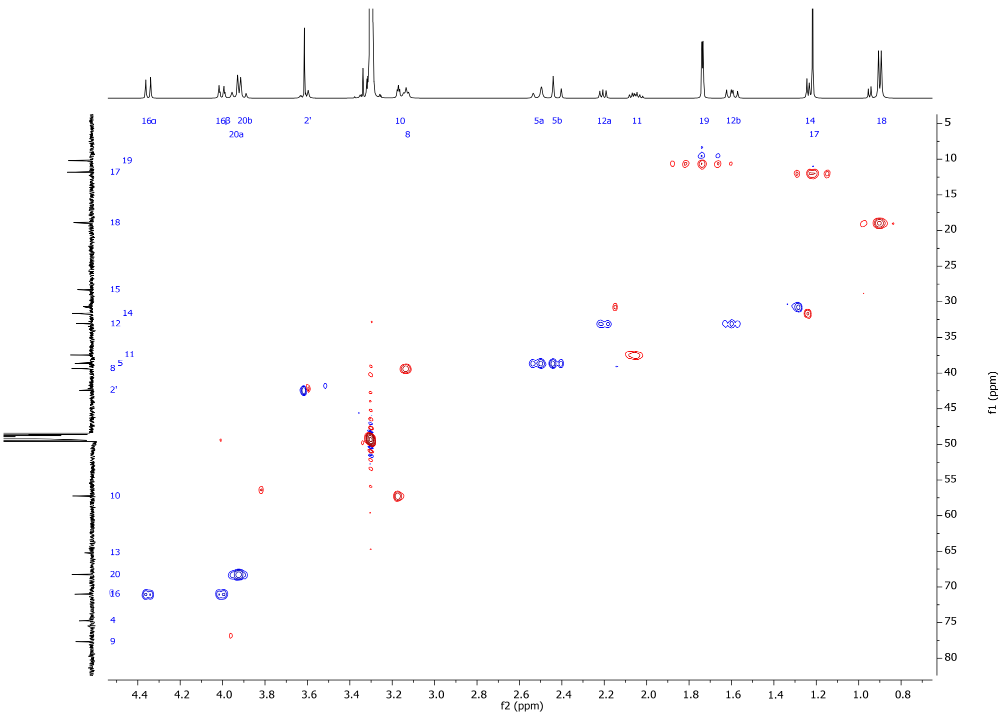

Figure S3e. Expansion (δ_H 4.7-0.6, δ_C 80-2) of HSQC spectrum of DPPBz (**3**) in CD_3OD.

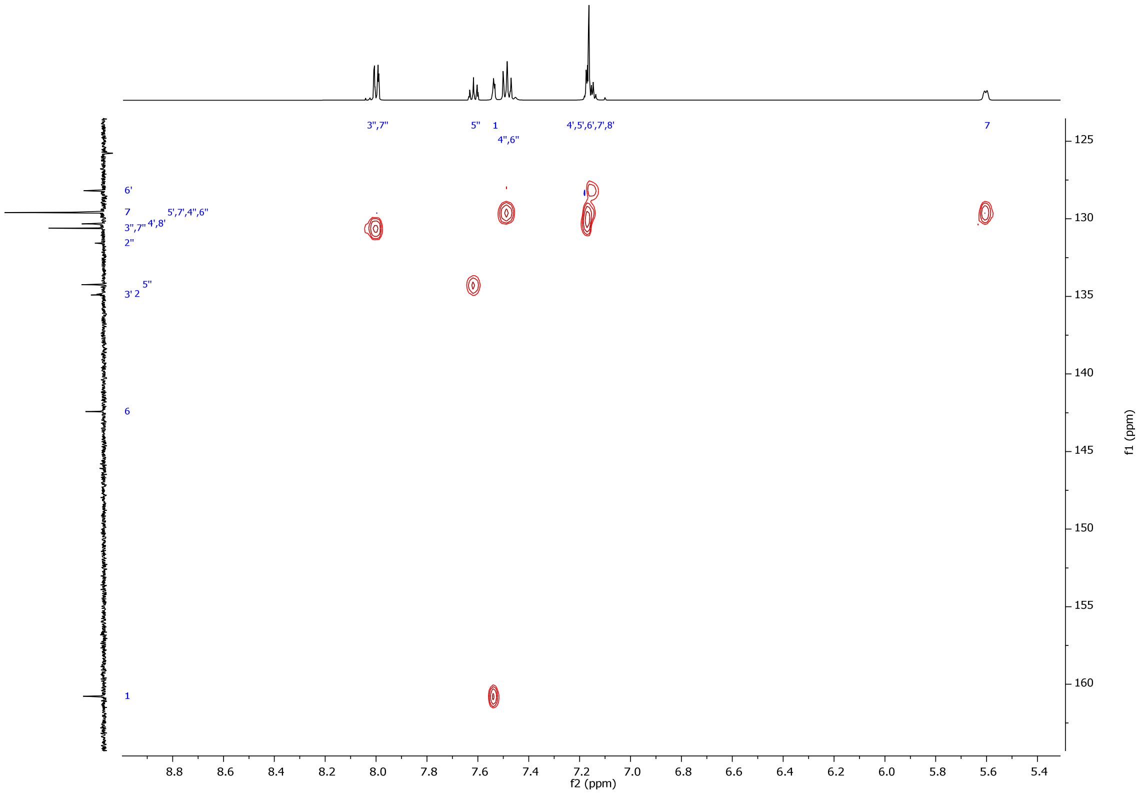

Figure S3f. Expansion (δ_H 8.3-5.4, δ_C 162-120) of HSQC spectrum of DPPBz (**3**) in CD_3OD.

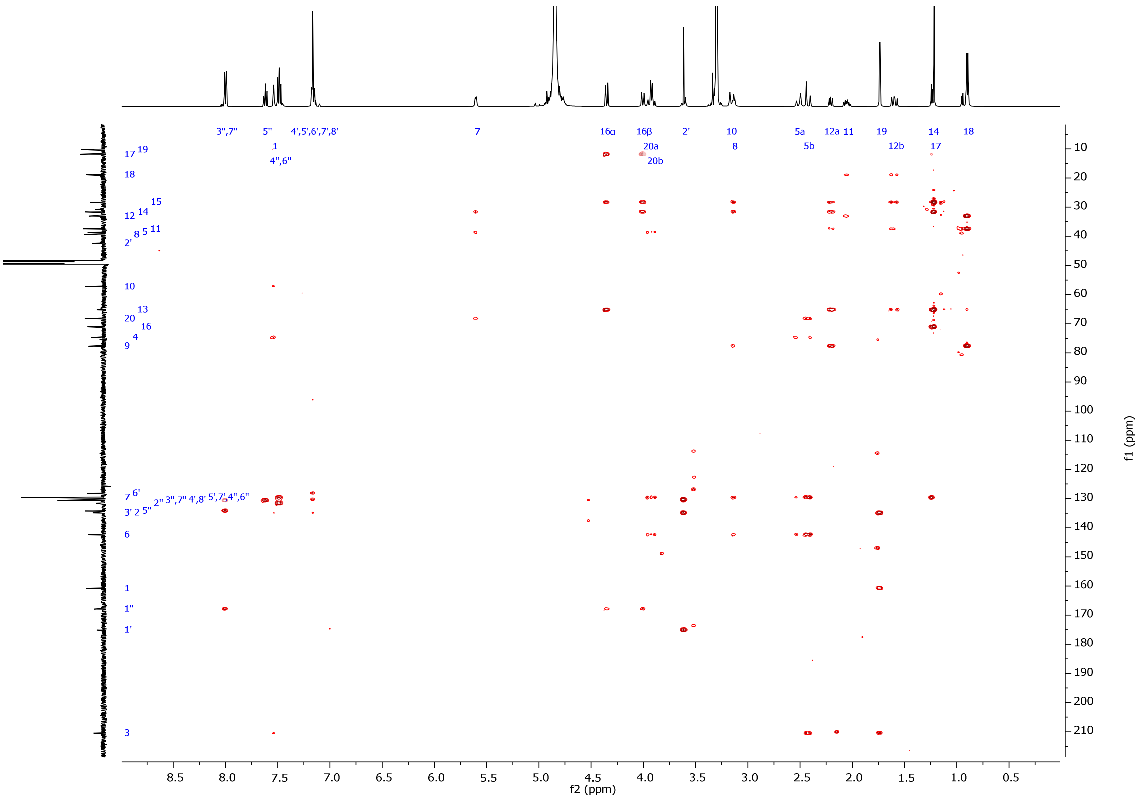

Figure S3g. HMBC spectrum of DPPBz (**3**) in CD_3OD.

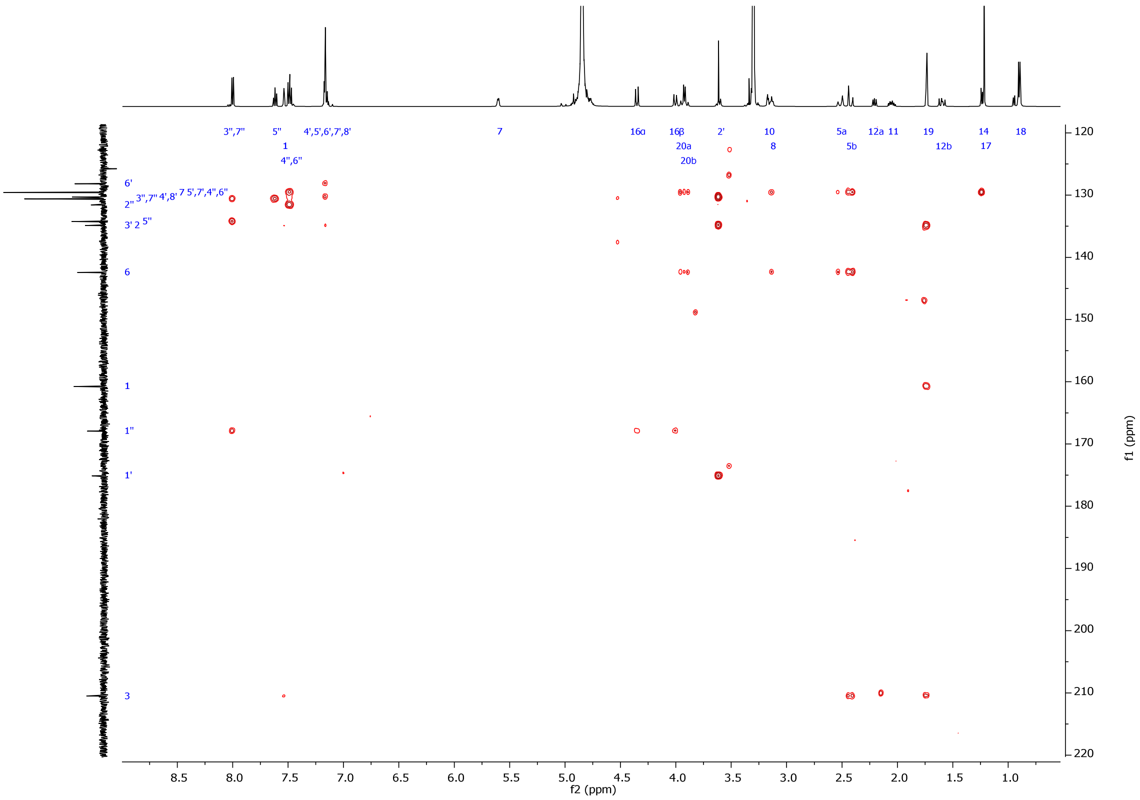

Figure S3h. Expansion (δ_H 8.9-0.2, δ_C 218-119) of HMBC spectrum of DPPBz (**3**) in CD_3OD.

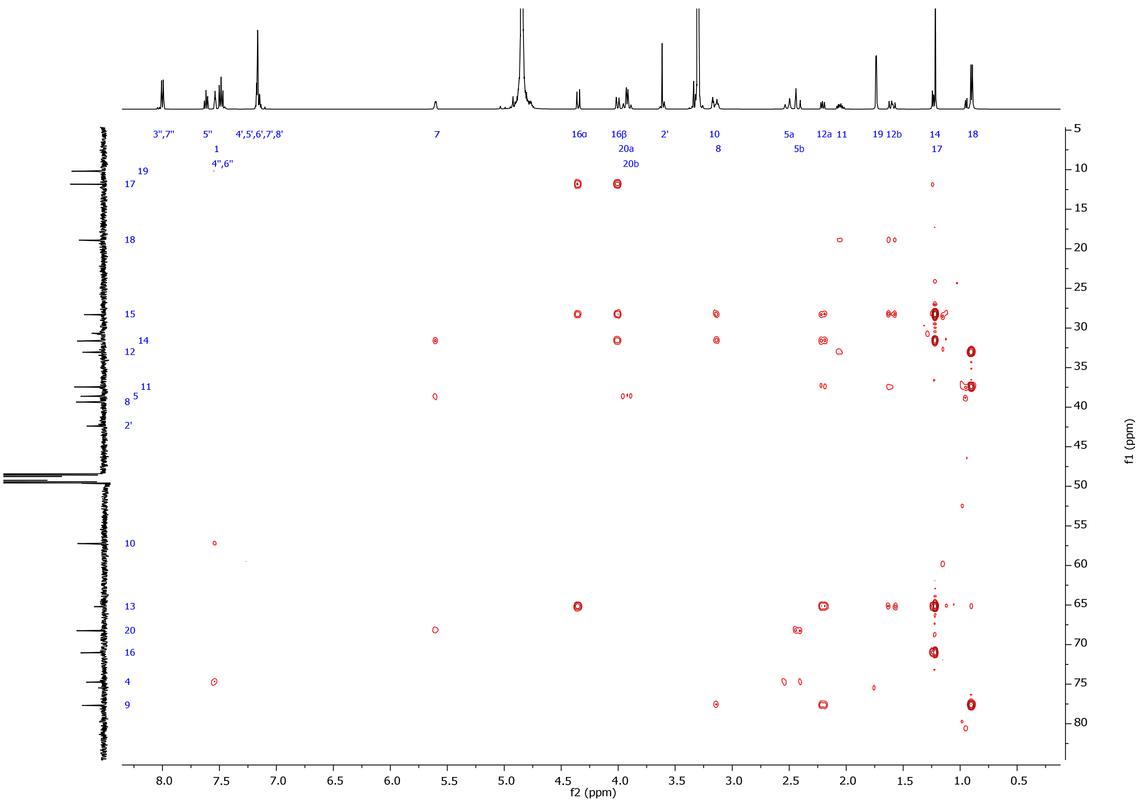

Figure S3i. Expansion (δ_H 8.4-0.4, δ_C 82-5) of HMBC spectrum of DPPBz (**3**) in CD_3OD.

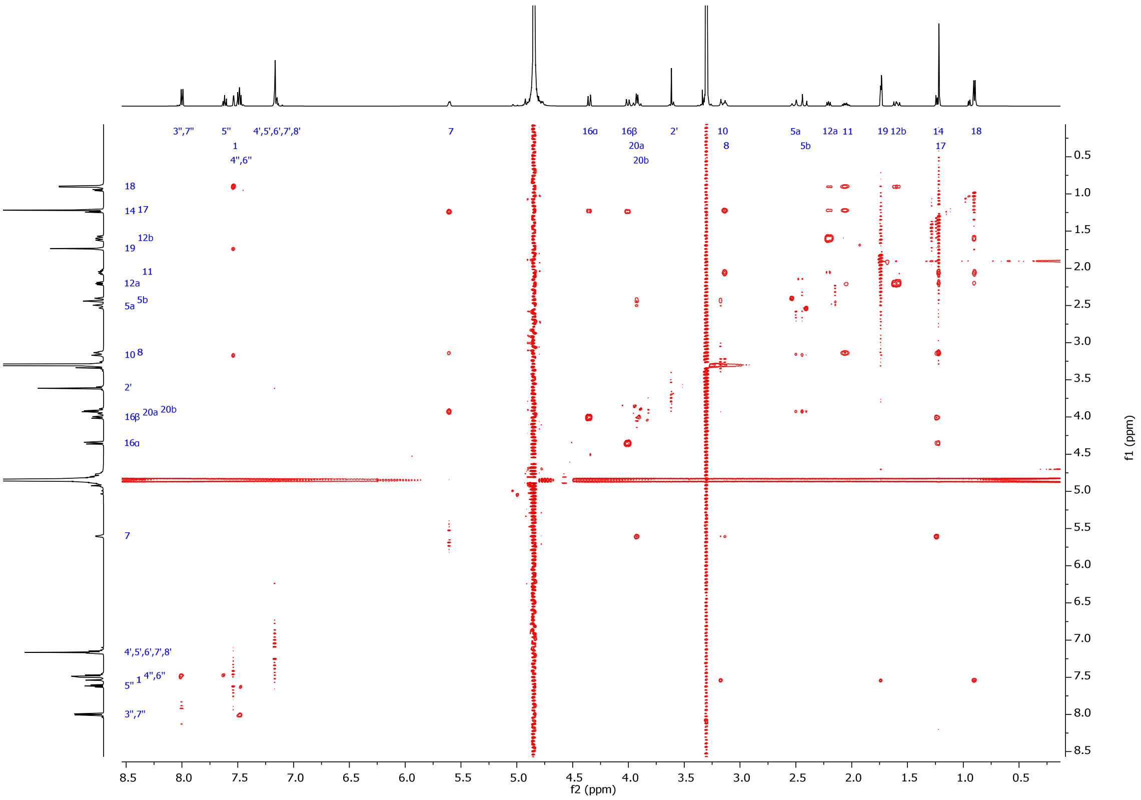

Figure S3j. NOESY2D spectrum of DPPBz (**3**) in CD_3OD

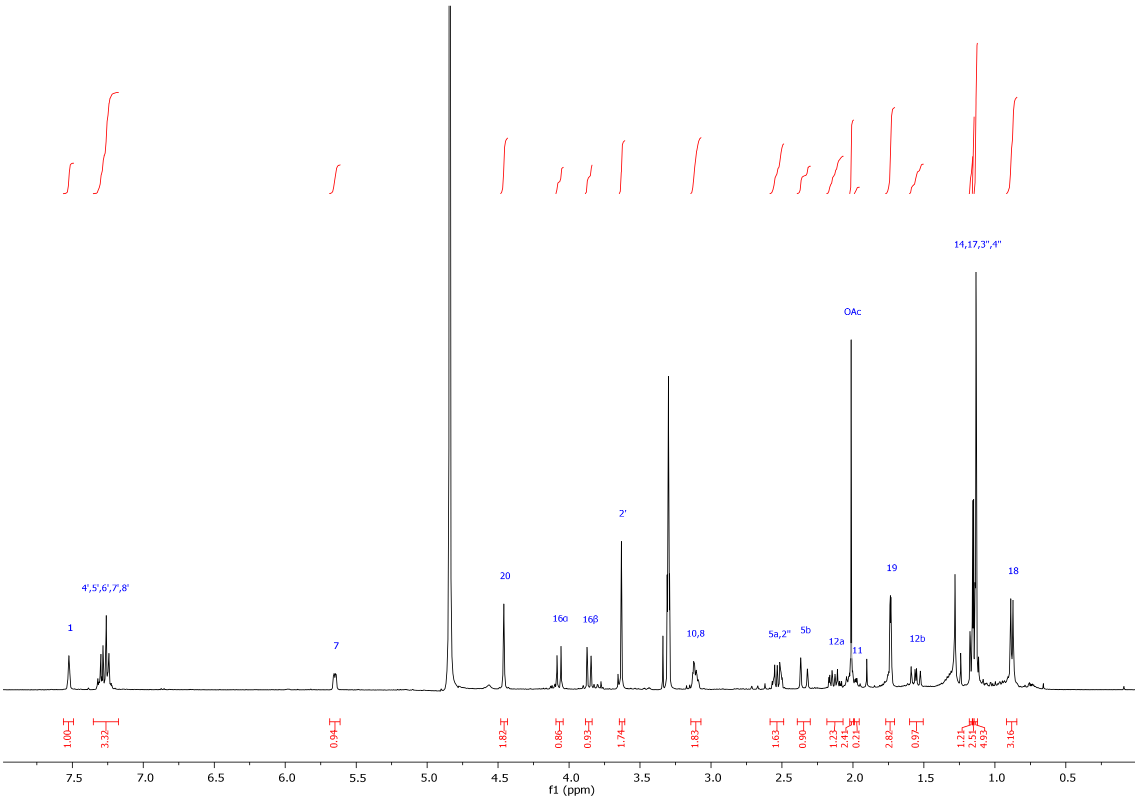

Figure S4a. ^{1}H-NMR spectrum of AcDPPI (**4**) in CD_3OD. (400 MHz).

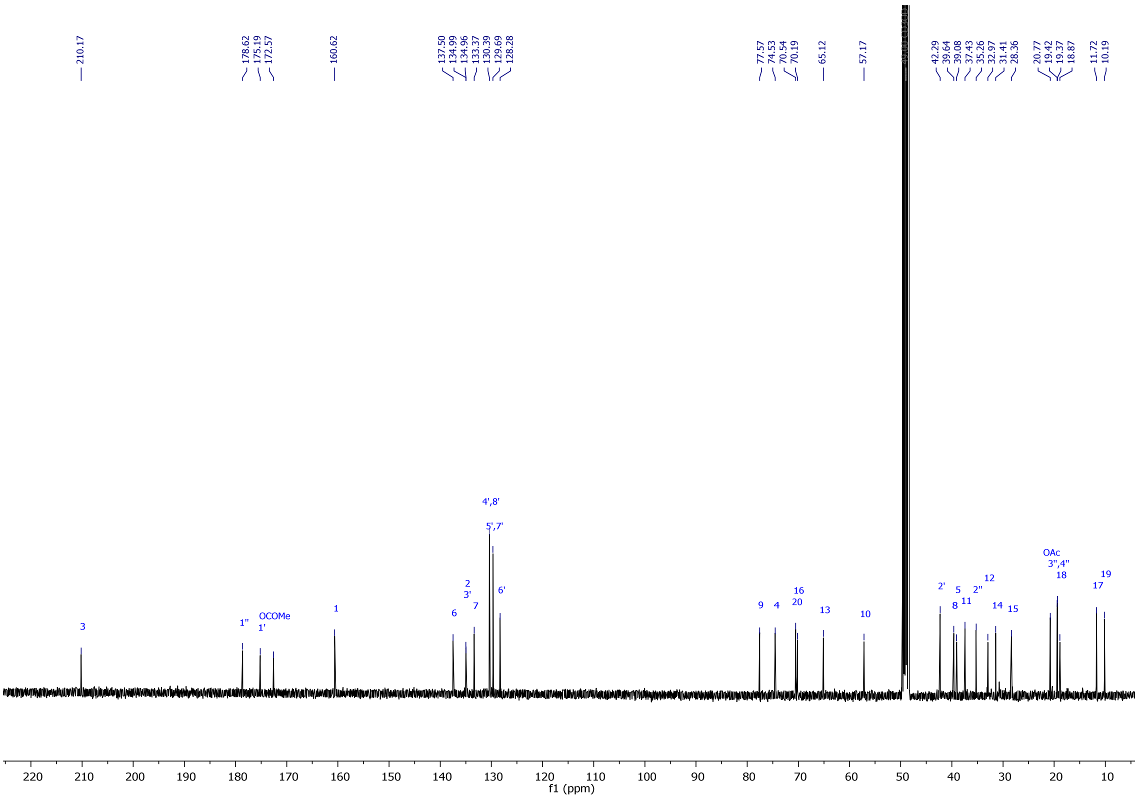

Figure S4b. ^{13}C-NMR spectrum ofAcDPPI (**4**) in CD_3OD. (100 MHz).

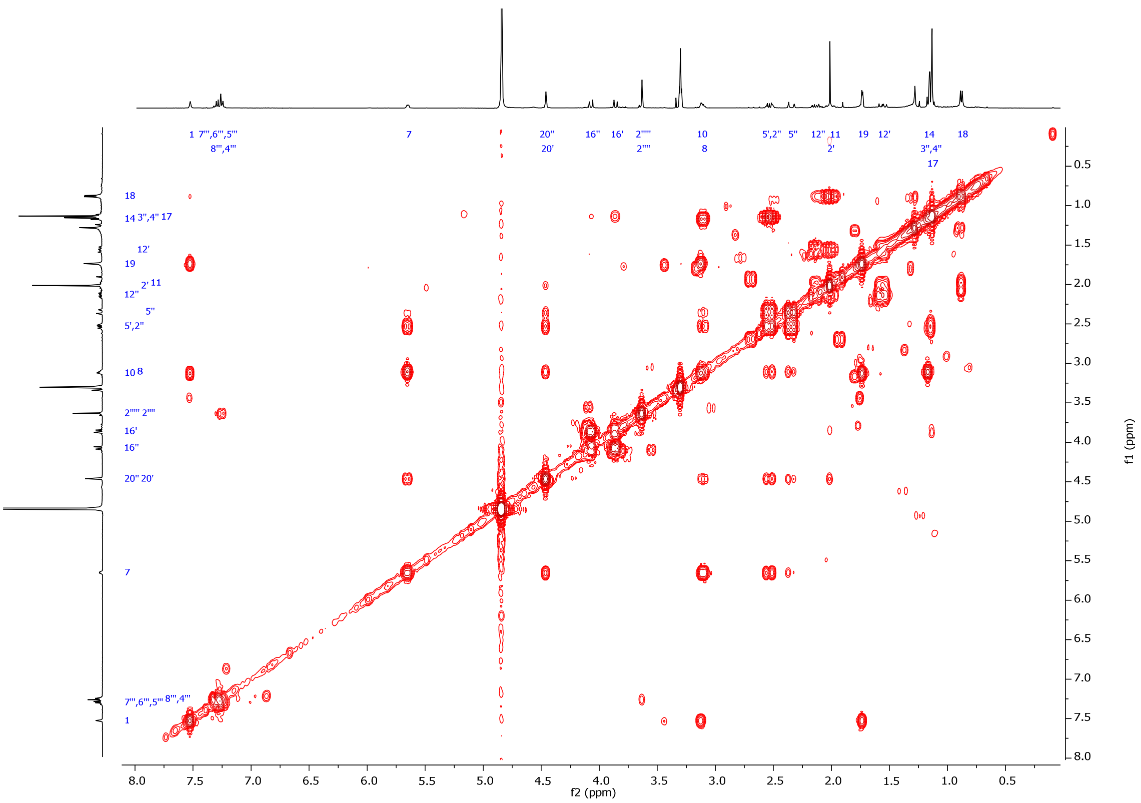

Figure S4c. COSY spectrum of AcDPPI (**4**) in CD_3OD.

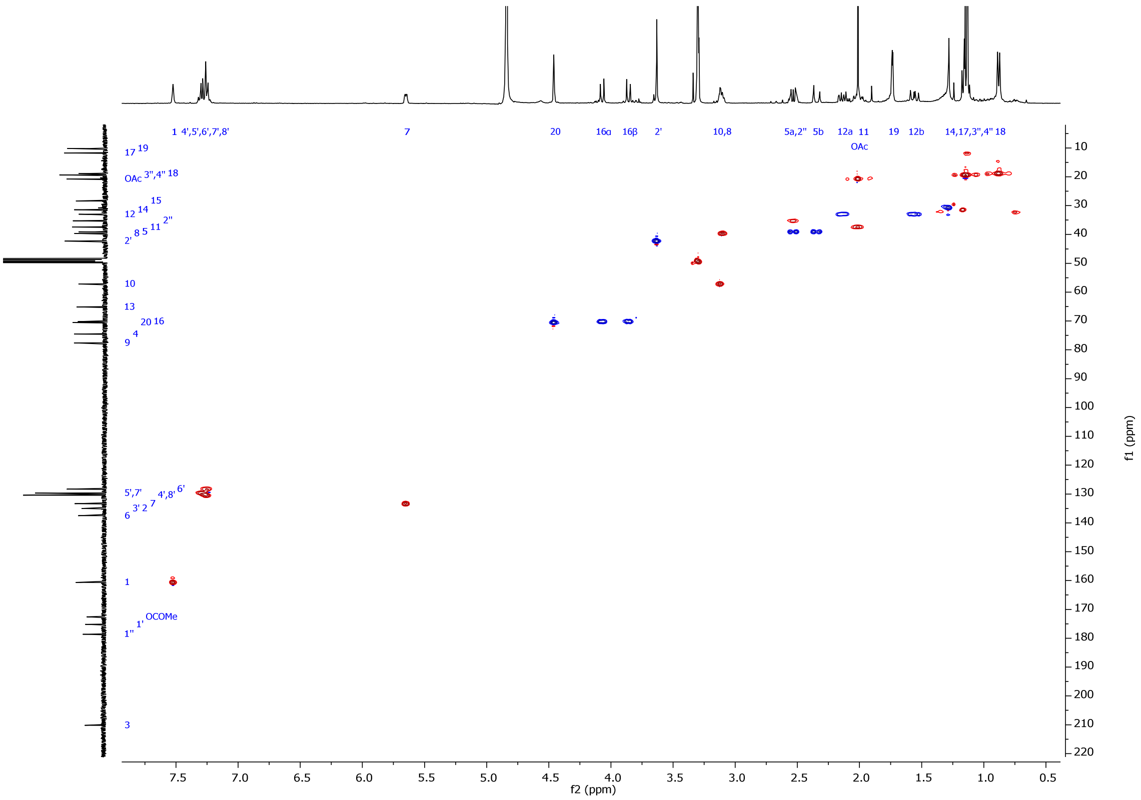

Figure S4d. HSQC spectrum of AcDPPI (**4**) in CD_3OD.

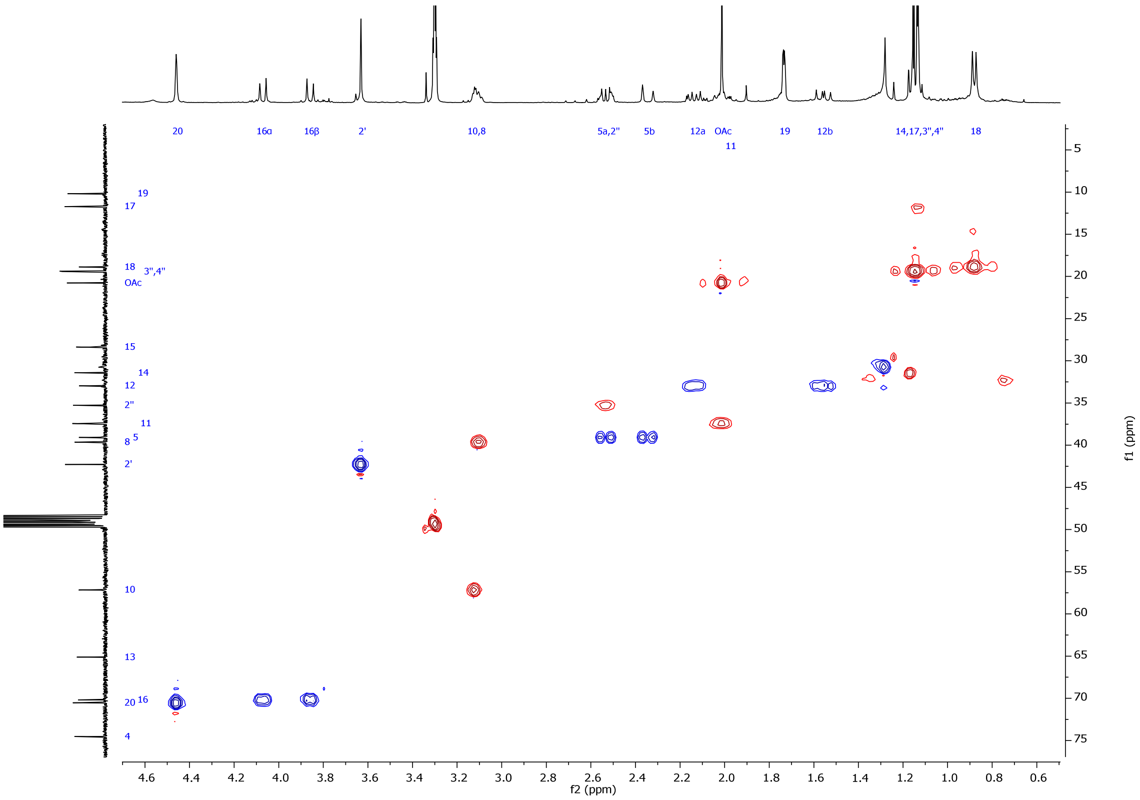

Figure S4e. Expansion (δ_H 4.7-0.5, δ_C 77-2) of HSQC spectrum of AcDPPI (**4**) in CD_3OD.

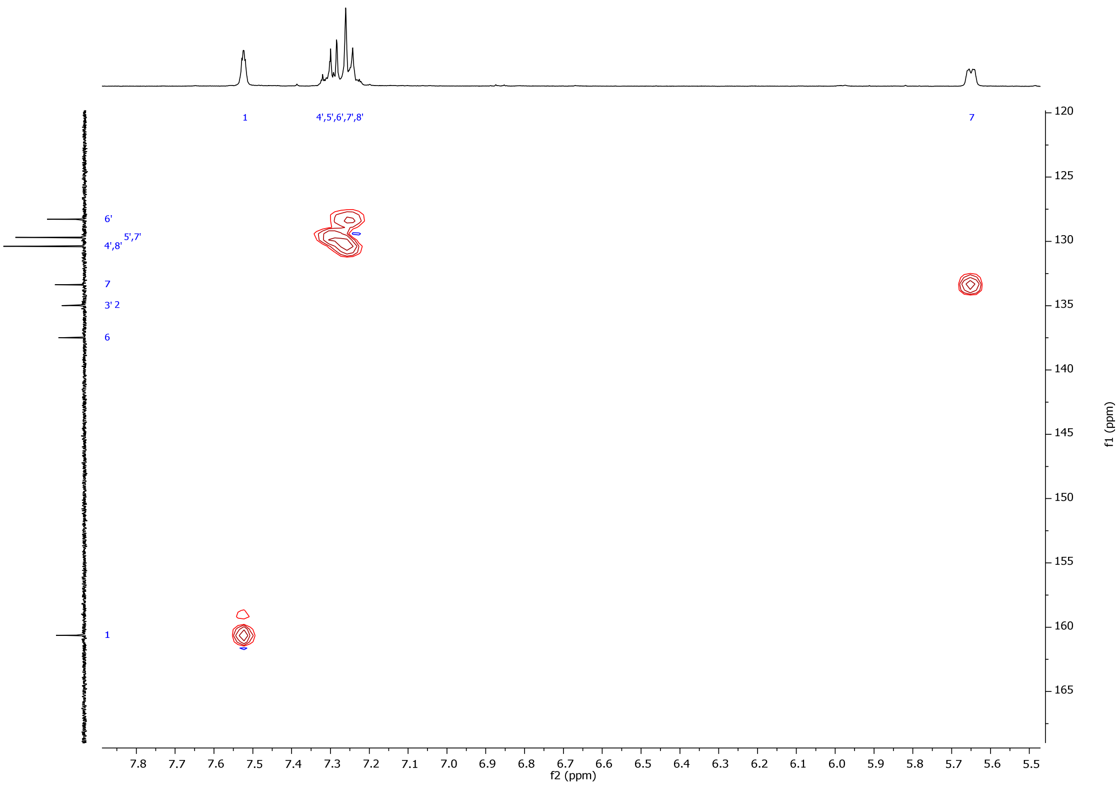

Figure S4f. Expansion (δ_H 7.8-5.5, δ_C 165-120) of HSQC spectrum of AcDPPI (**4**) in CD_3OD.

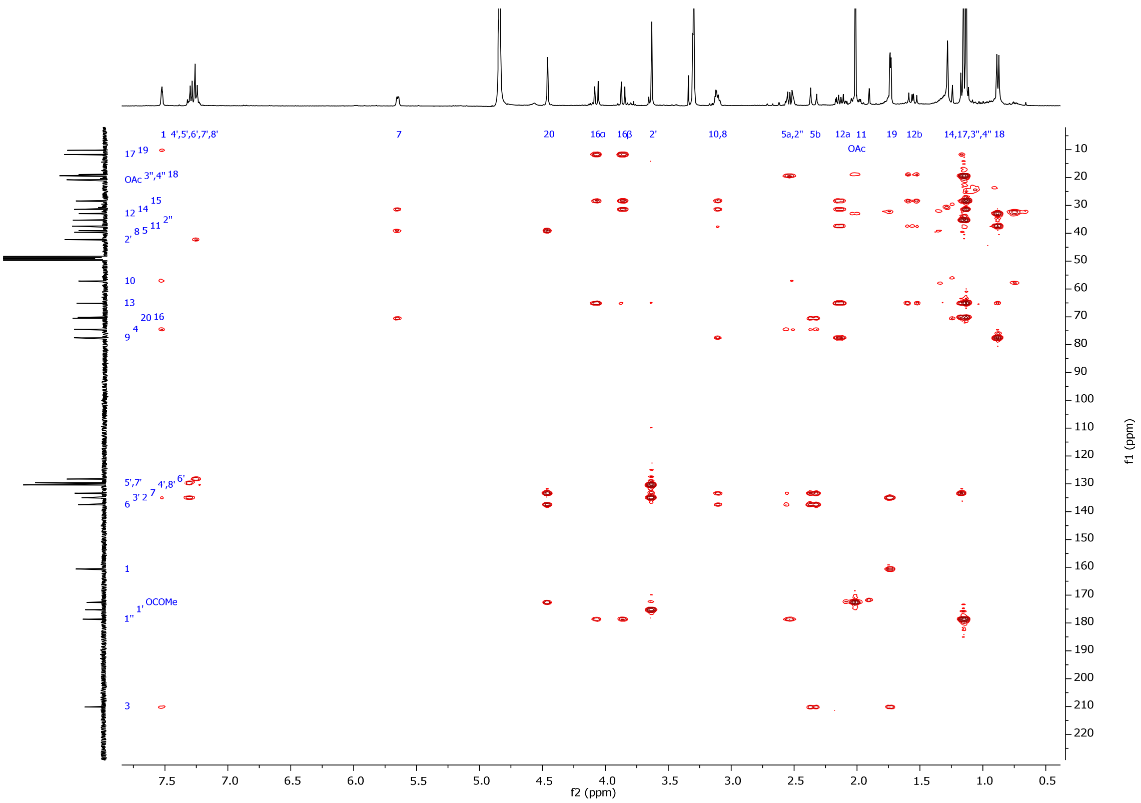

Figure S4g. HMBC spectrum of AcDPPI (**4**) in CD_3OD.

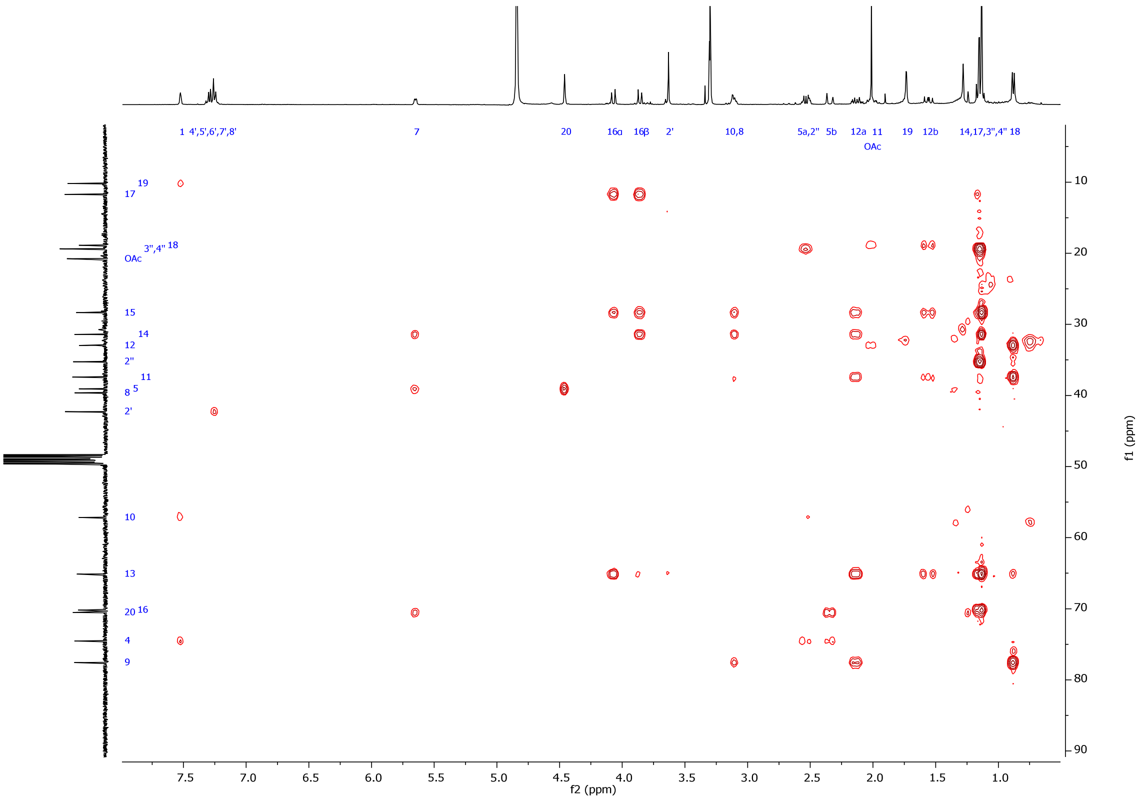

Figure S4h. Expansion (δ_H 8.0-0.4, δ_C 91-5) of HMBC spectrum of AcDPPI (**4**) in CD_3OD

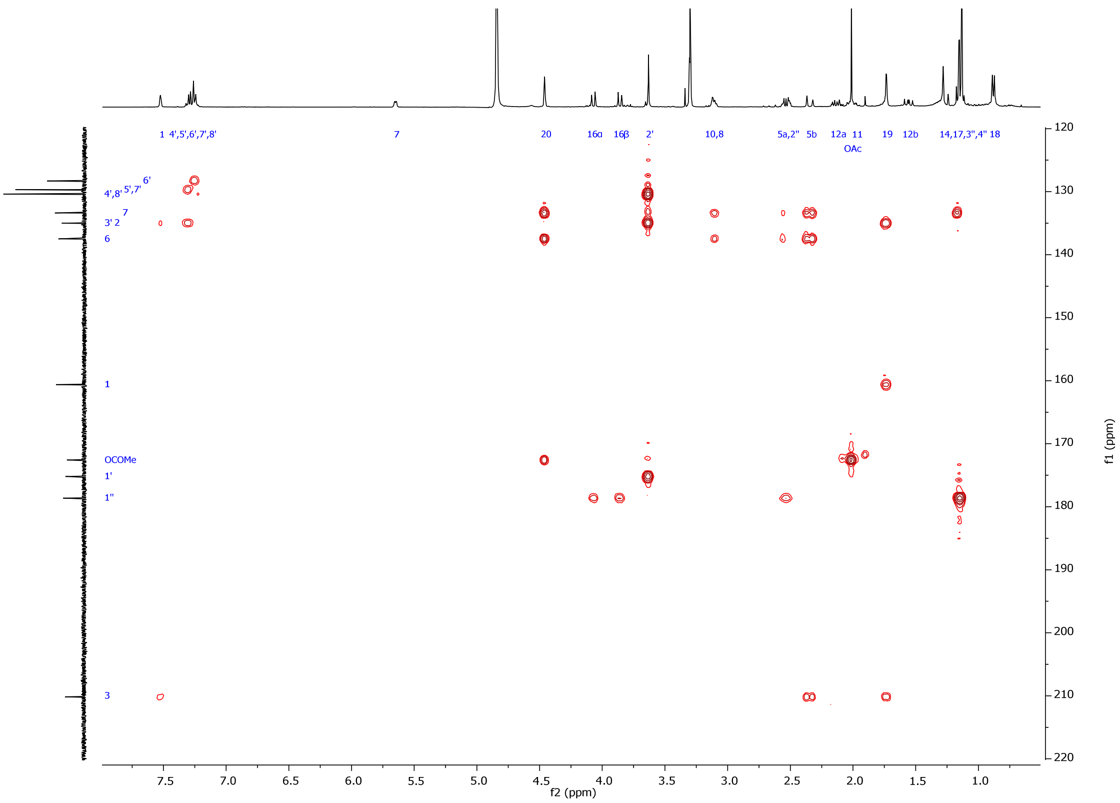

Figure S4i. Expansion (δ_H 8.0-0.4, δ_C 220-120) of HMBC spectrum of AcDPPI (**4**) in CD_3OD

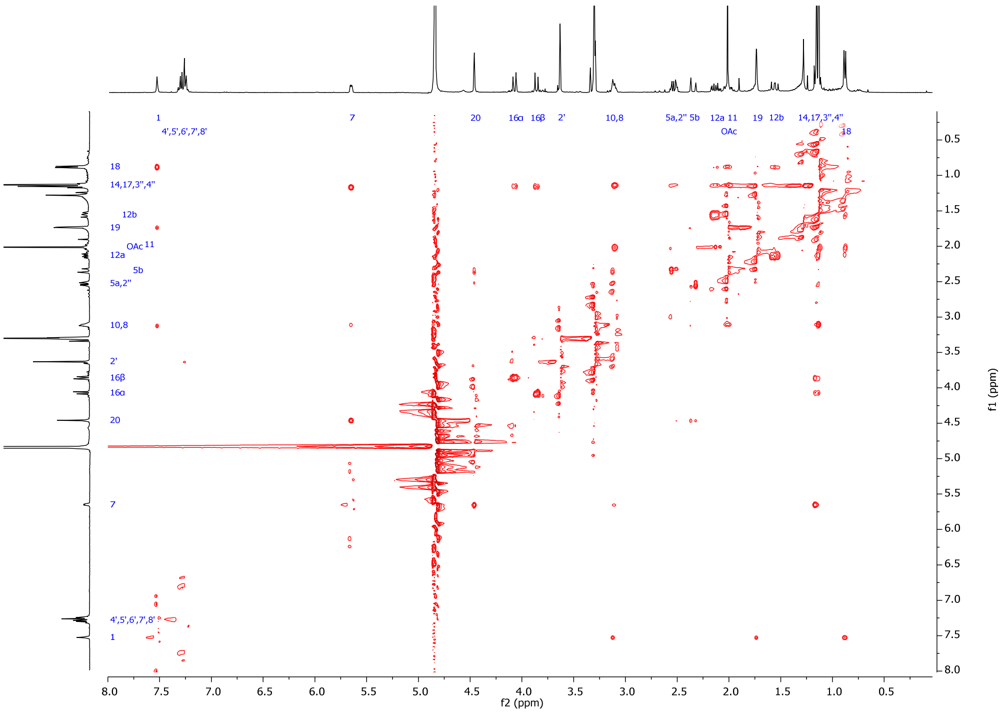

Figure S4j. NOESY2D spectrum of AcDPPI (**4**) in CD_3OD.

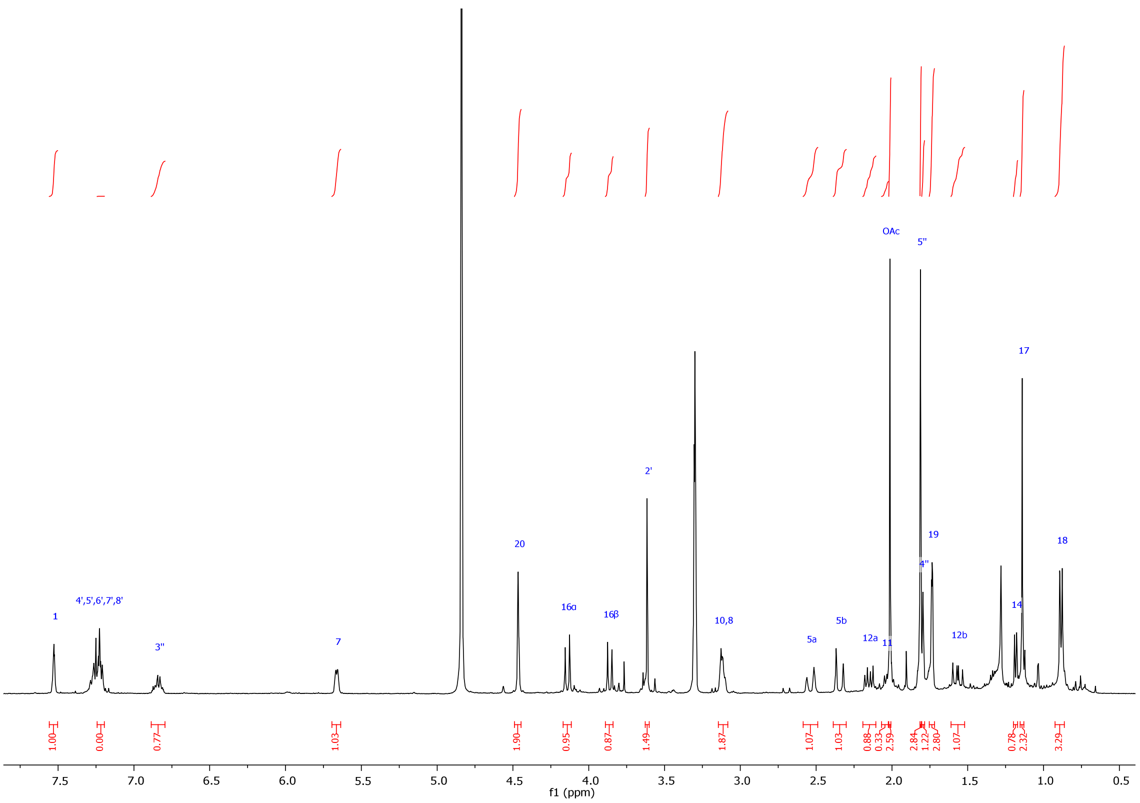

Figure S5a. ^{1}H NMR spectrum of AcDPPT (**5**) in CD₃OD (400 MHz).

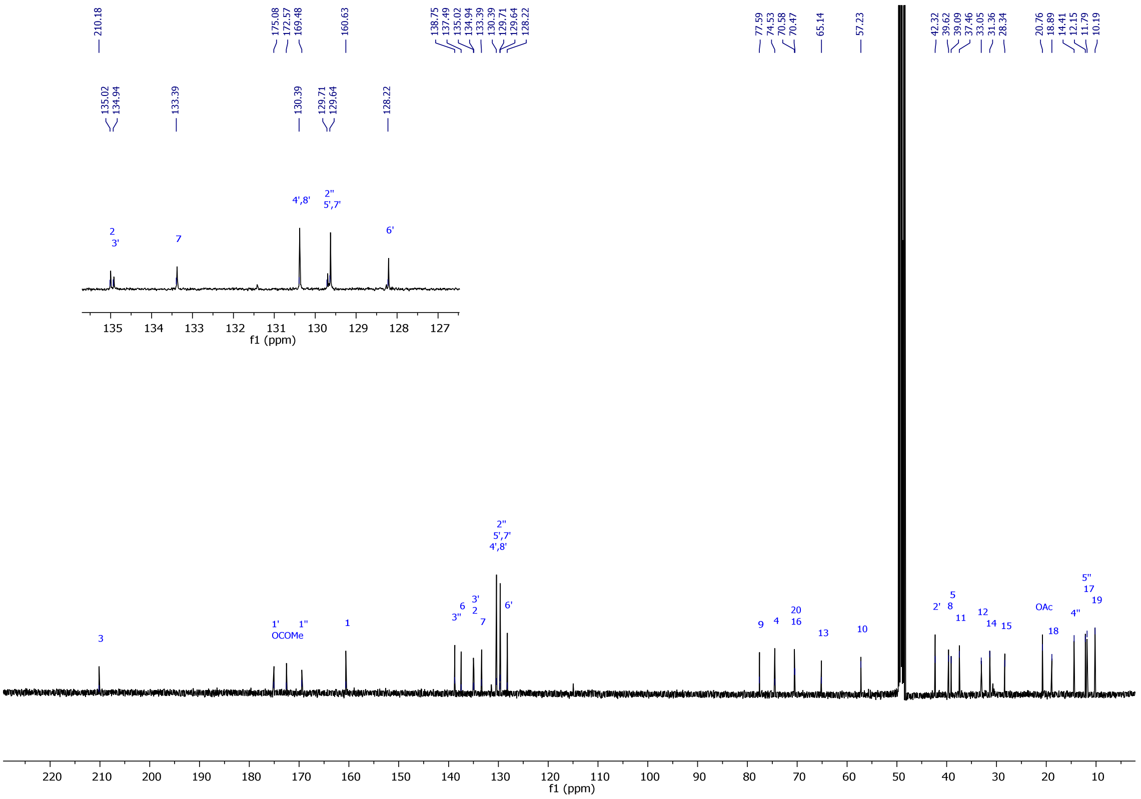

Figure S5b. ^{13}C NMR spectrum of AcDPPT (**5**) in CD₃OD (100 MHz).

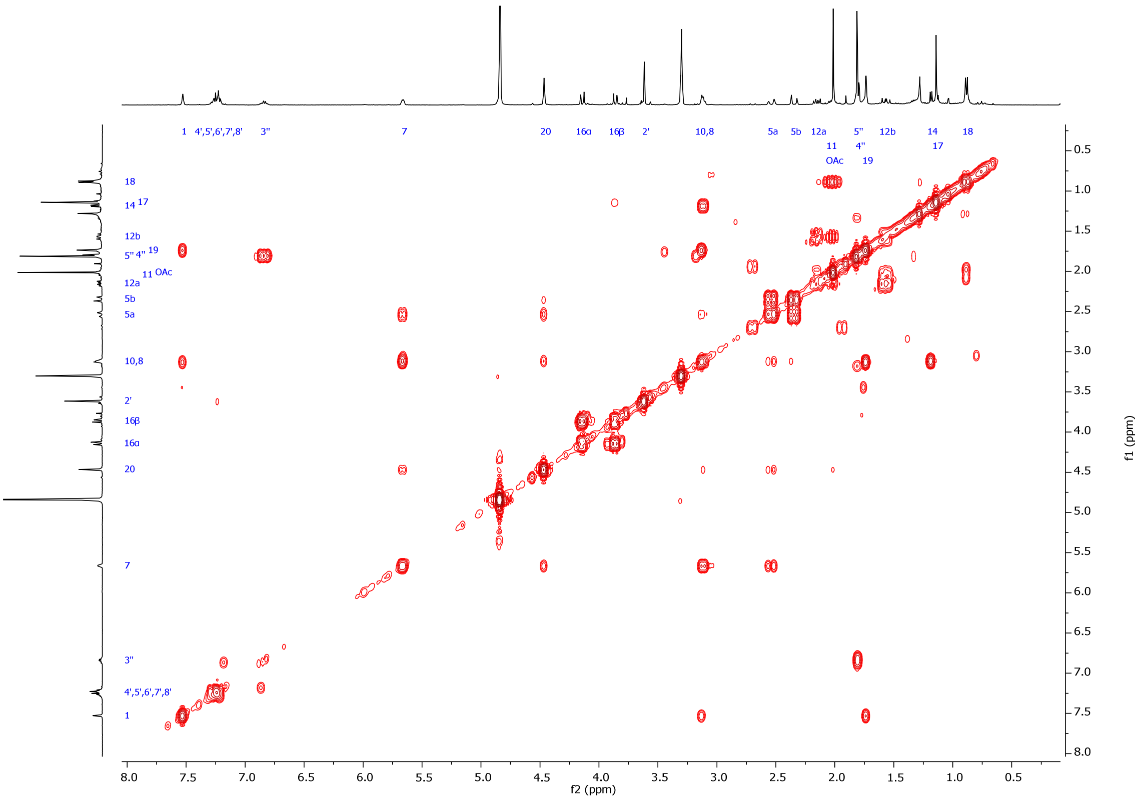

Figure S5c. COSY spectrum of AcDPPT (**5**) in CD_3OD.

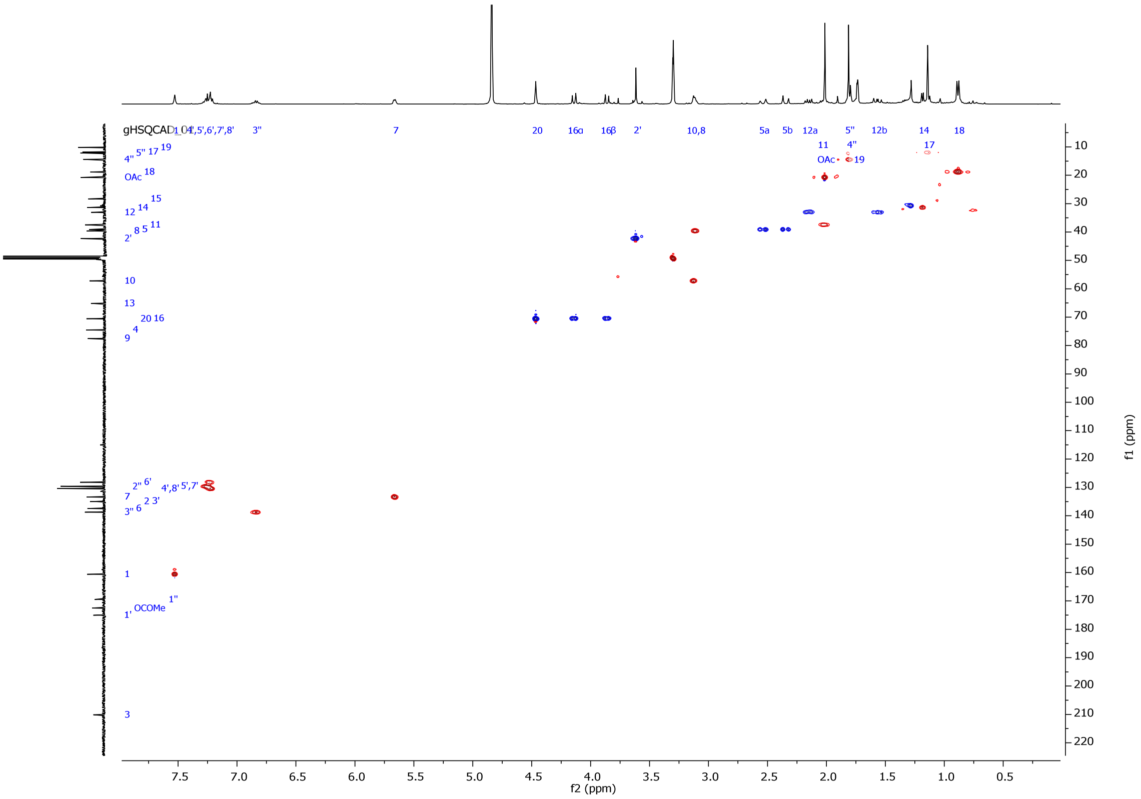

Figure S5d. HSQC spectrum of AcDPPT (**5**) in CD_3OD.

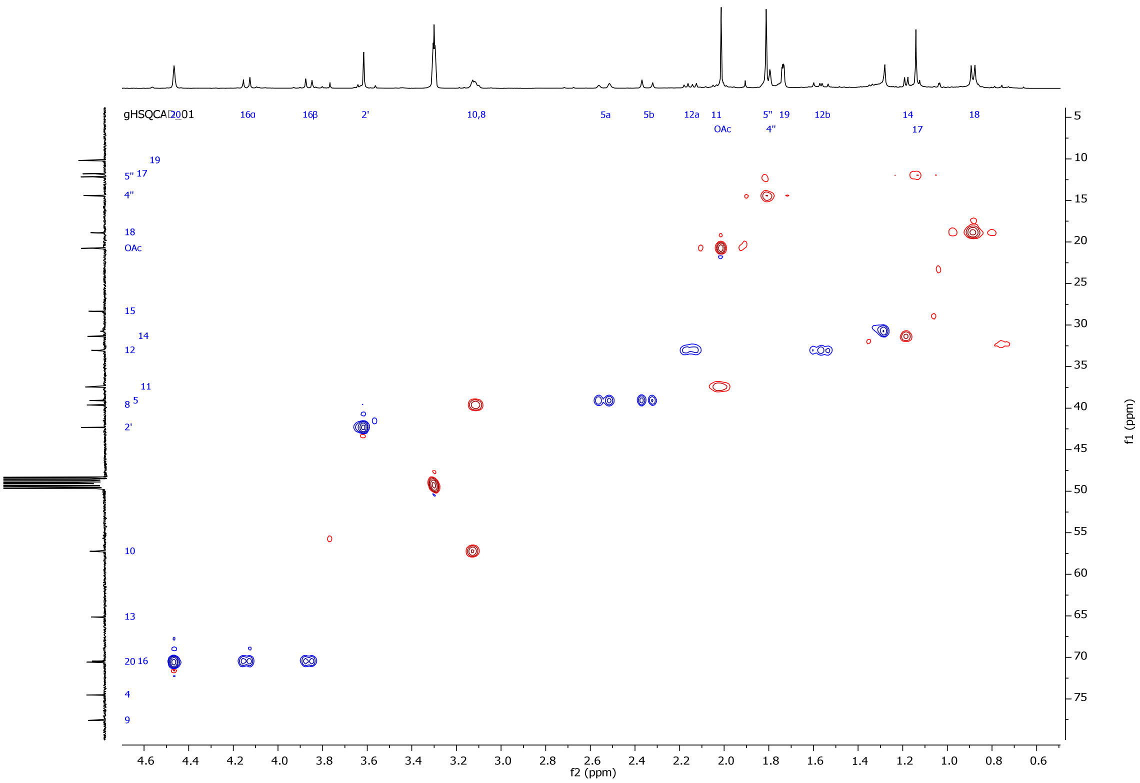

Figure S5e. Expansion (δ_H 4.7-0.5, δ_C 78-4) of HSQC spectrum of AcDPPT (**5**) in CD_3OD.

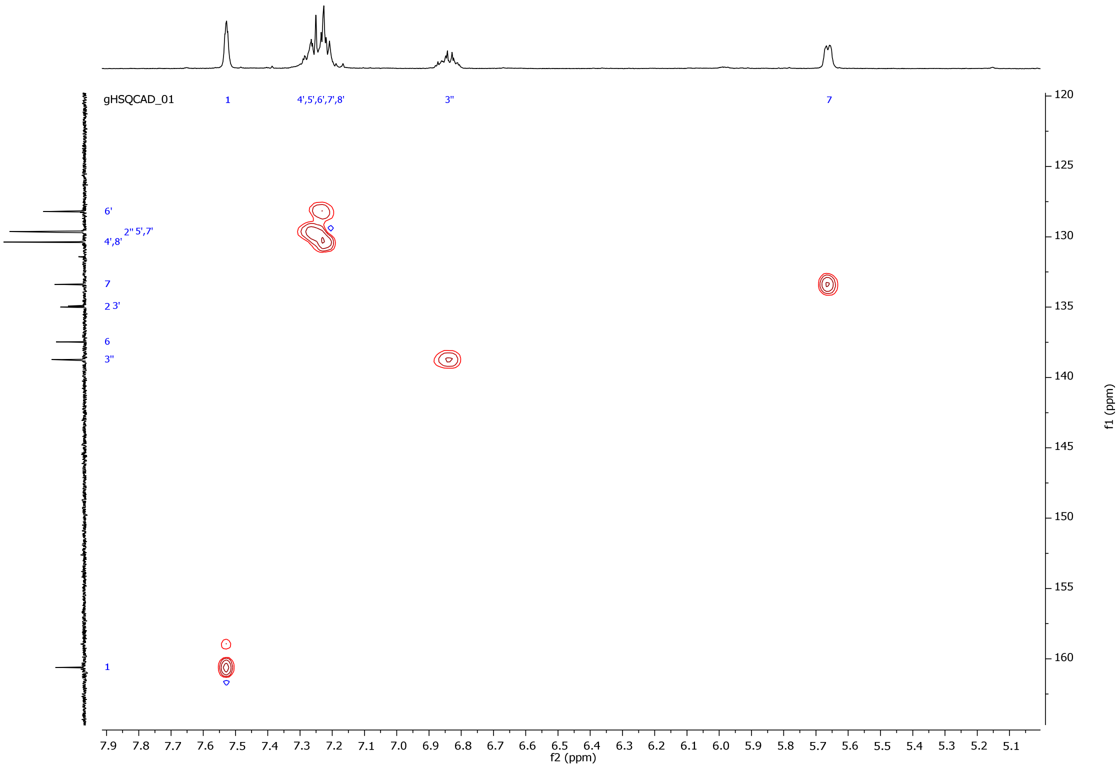

Figure S5f. Expansion (δ_H 7.9-5.0, δ_C 165-120) of HSQC spectrum of AcDPPT (**5**) in CD_3OD.

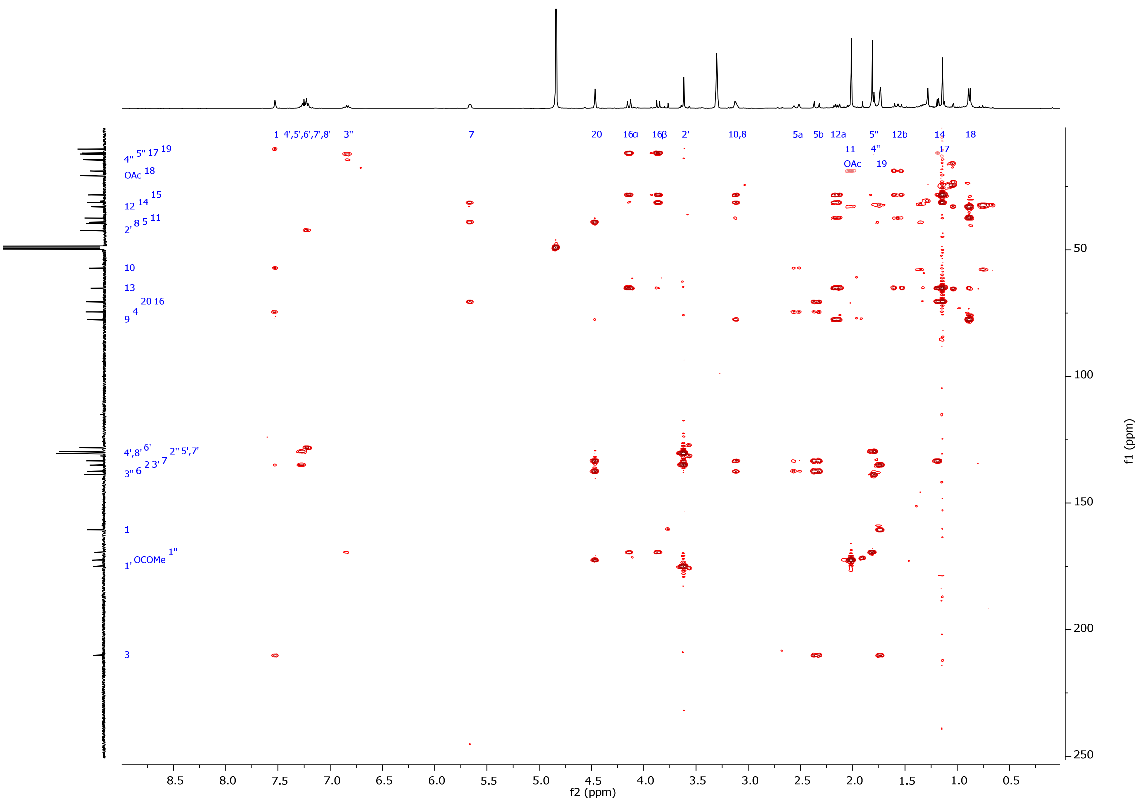

Figure S5g. HMBC spectrum of AcDPPT (**5**) in CD_3OD.

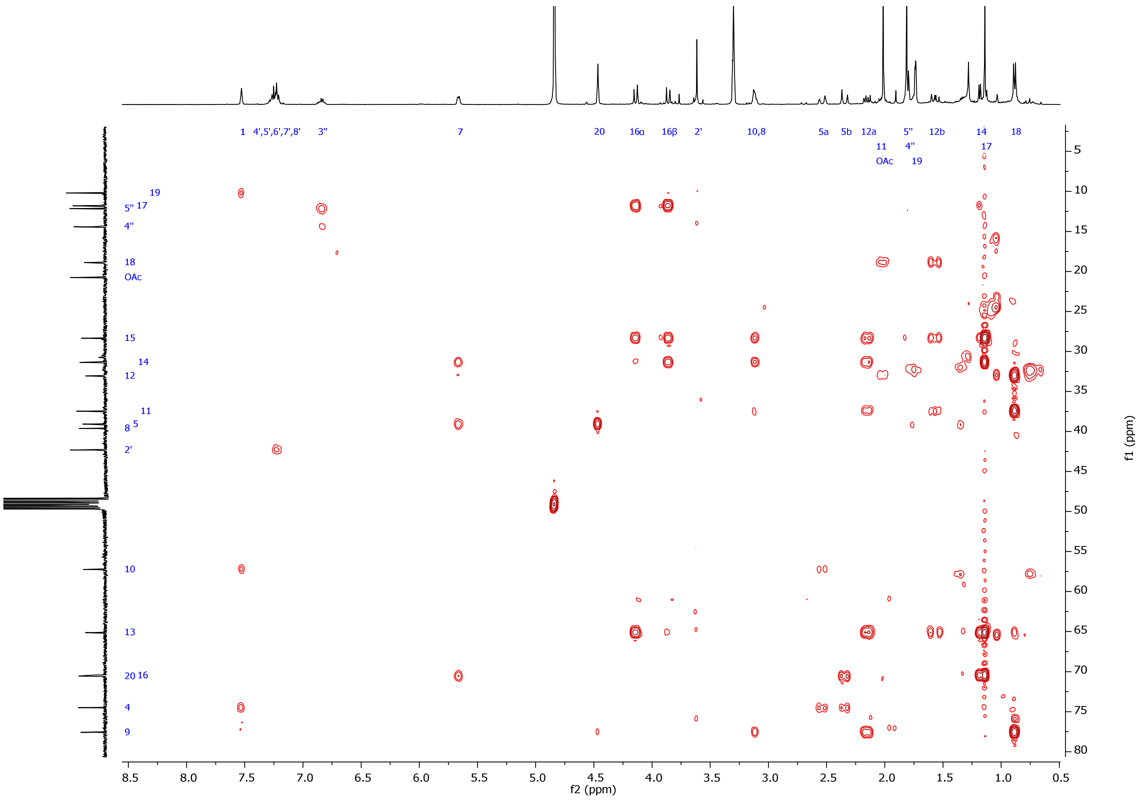

Figure S5h. Expansion (δ_H 8.5-0.5, δ_C 80-2) of HMBC spectrum of AcDPPT (**5**) in CD_3OD.

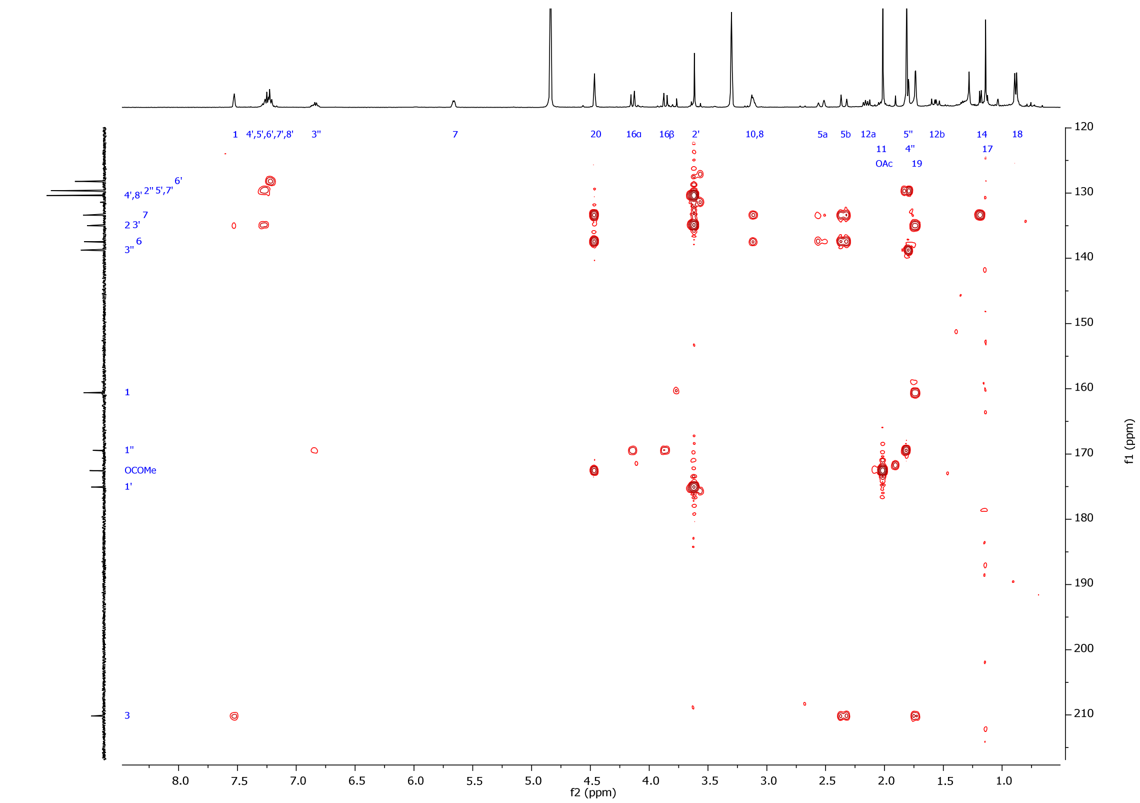

Figure S5i. Expansion (δ_H 8.5-0.5, δ_C 215-120) of HMBC spectrum of AcDPPT (**5**) in CD_3OD.

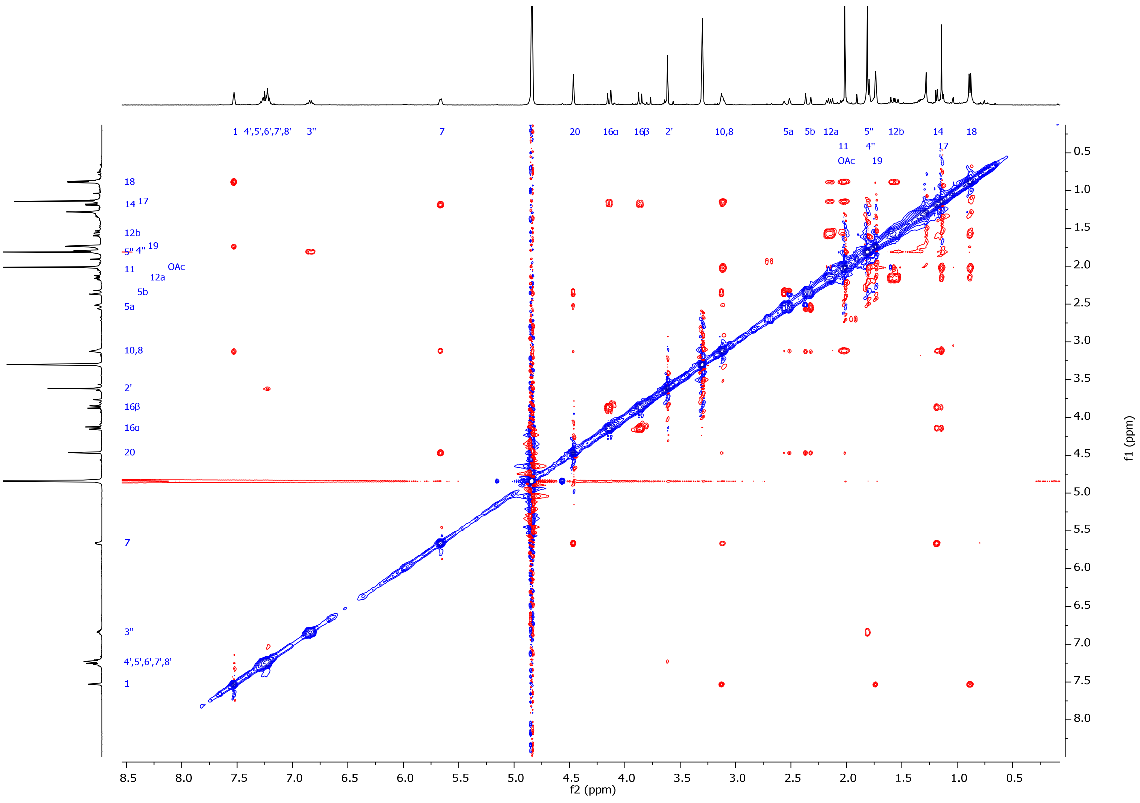

Figure S5j. NOESY2D spectrum of AcDPPT (**5**) in CD_3OD.

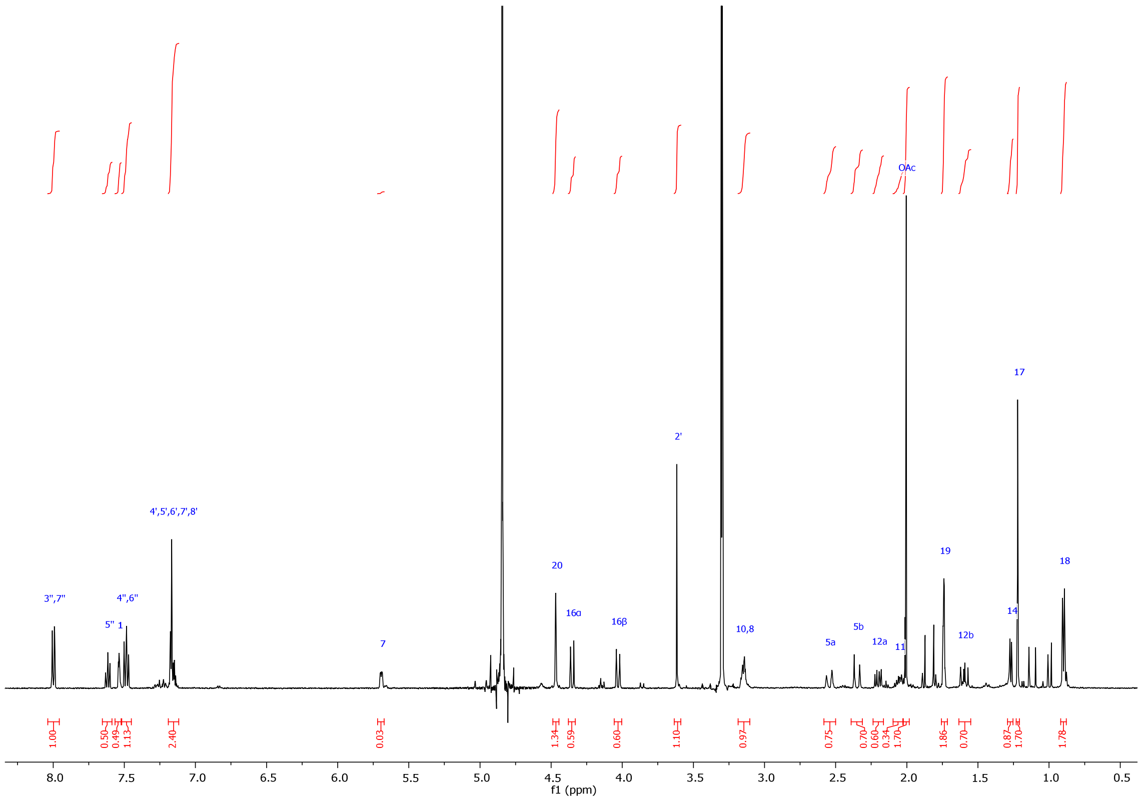

Figure S6a. ^{1}H-NMR spectrum of AcDPPBz (**6**) in CD_3OD (400 MHz).

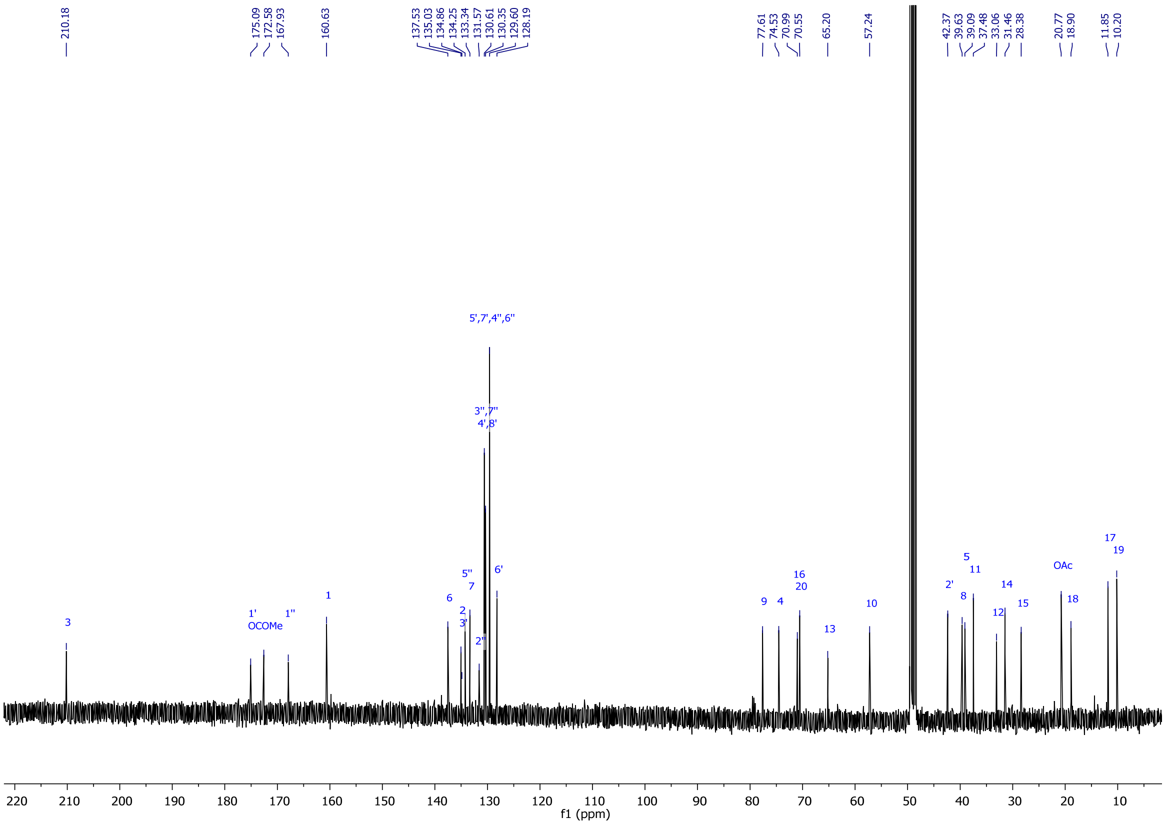

Figure S6b. ^{13}C-NMR spectrum of AcDPPBz (**6**) in CD_3OD (125 MHz).

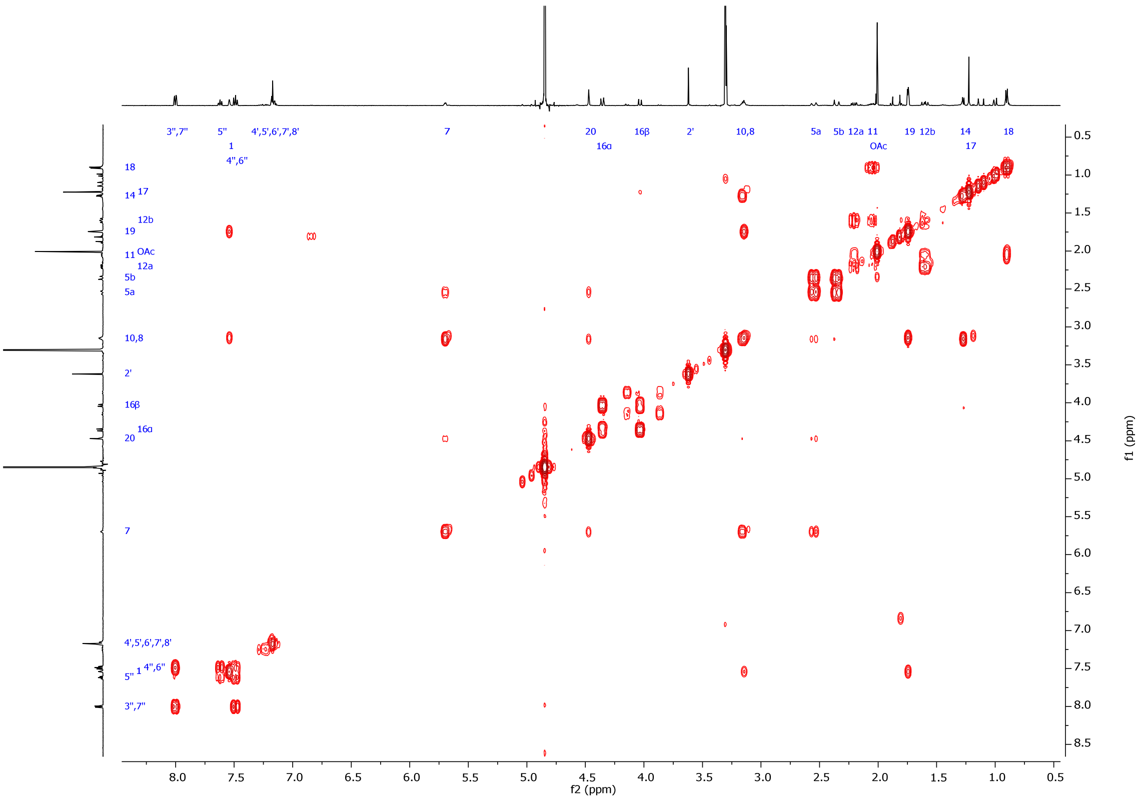

Figure S6c. COSY spectrum of AcDPPBz (**6**) inCD_3OD.

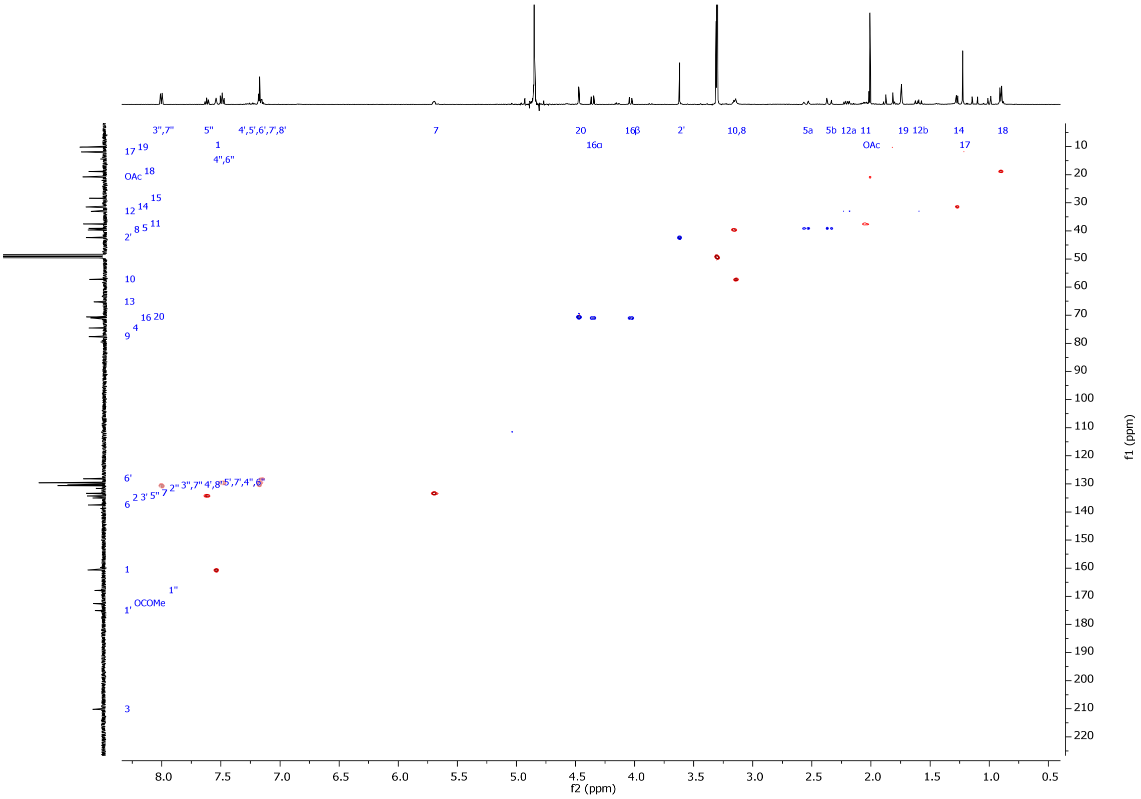

Figure S6d. HSQC spectrum of AcDPPBz (**6**) in CD_3OD.

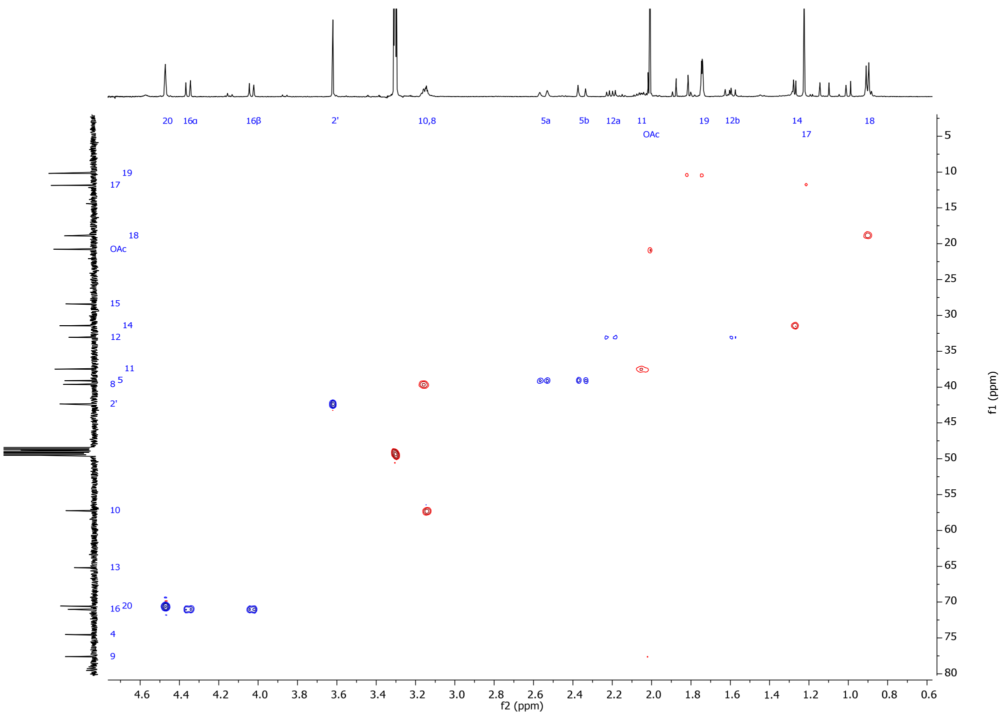

Figure S6e. Expansion (δ_H 4.7-0.6, δ_C 80-4) of HSQC spectrum of AcDPPBz (**6**) in CD_3OD.

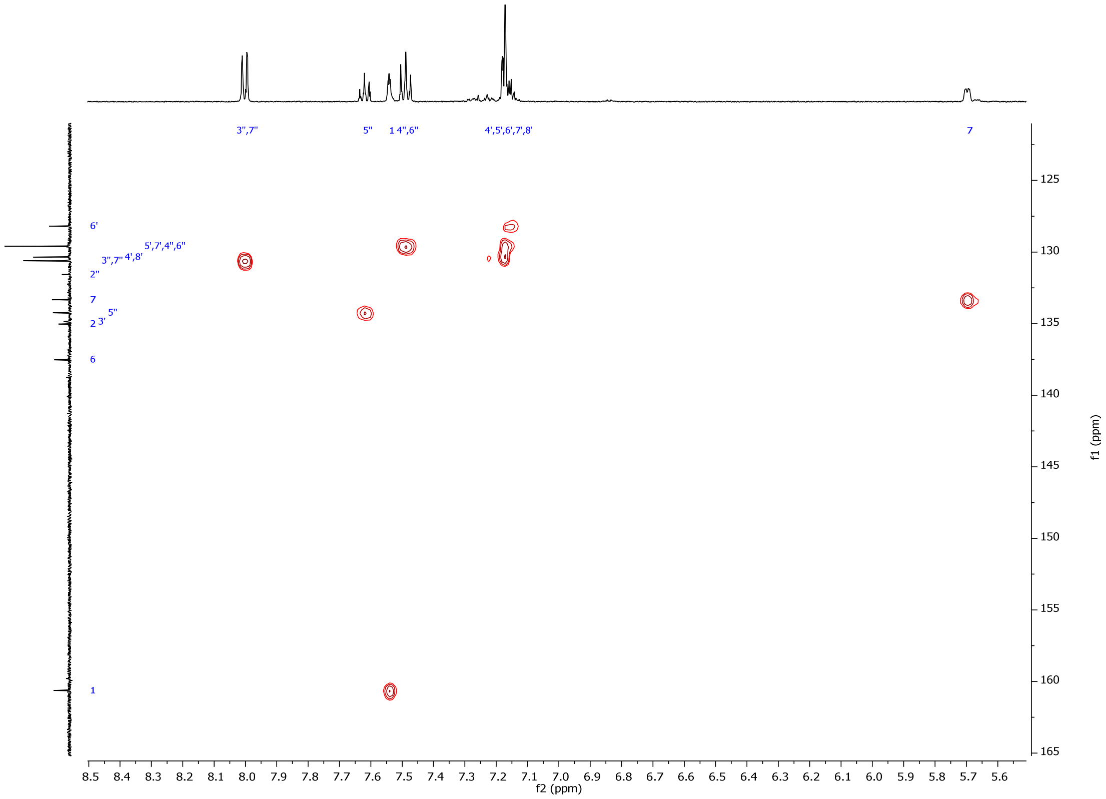

Figure S6f. Expansion (δ_H 8.5-5.5, δ_C 165-123) of HSQC spectrum of AcDPPBz (**6**) in CD_3OD.

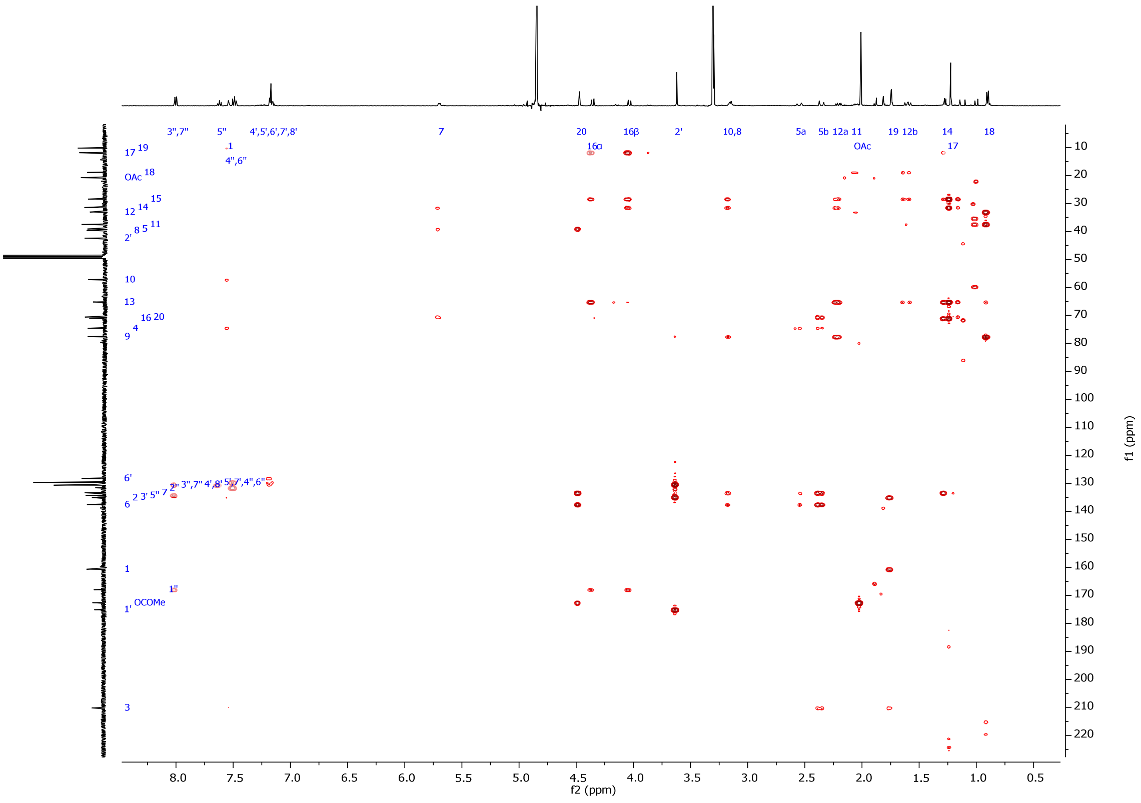

Figure S6g. HMBC spectrum of AcDPPBz (**6**) in CD_3OD.

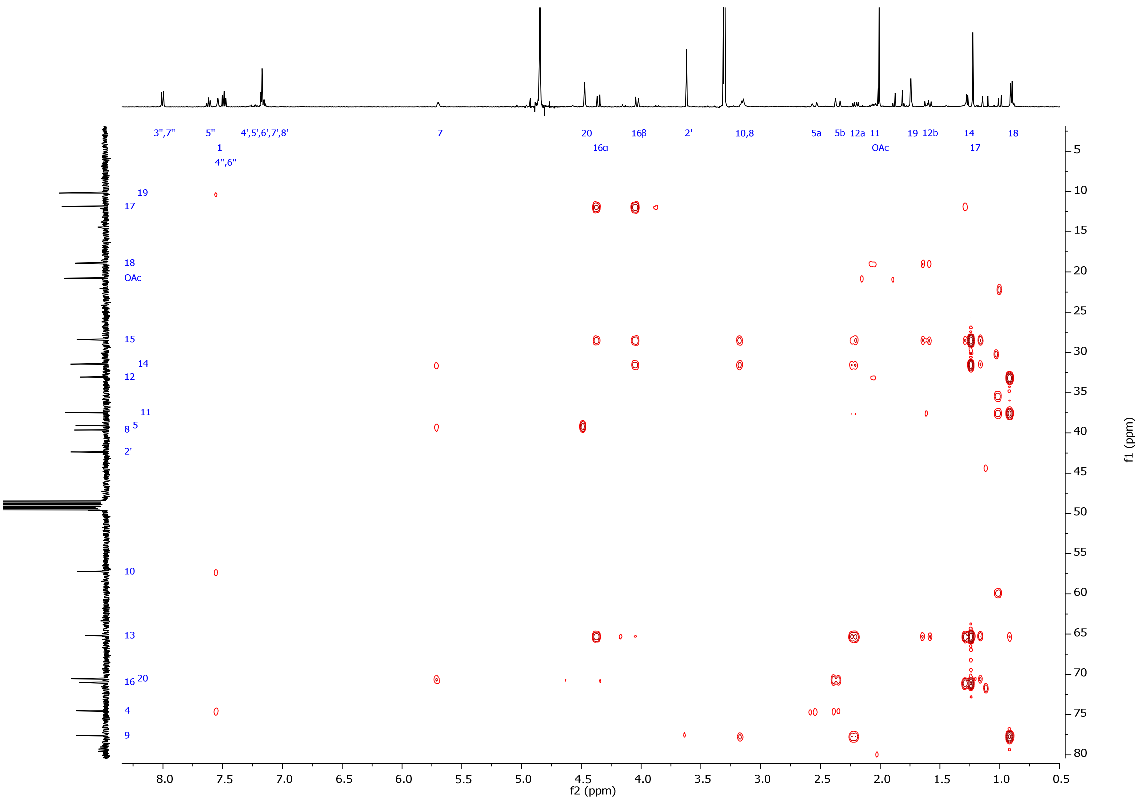

Figure S6h. Expansion (δ_H 8.3-0.5, δ_C 80-3) of HMBC spectrum of AcDPPBz (**6**) in CD_3OD.

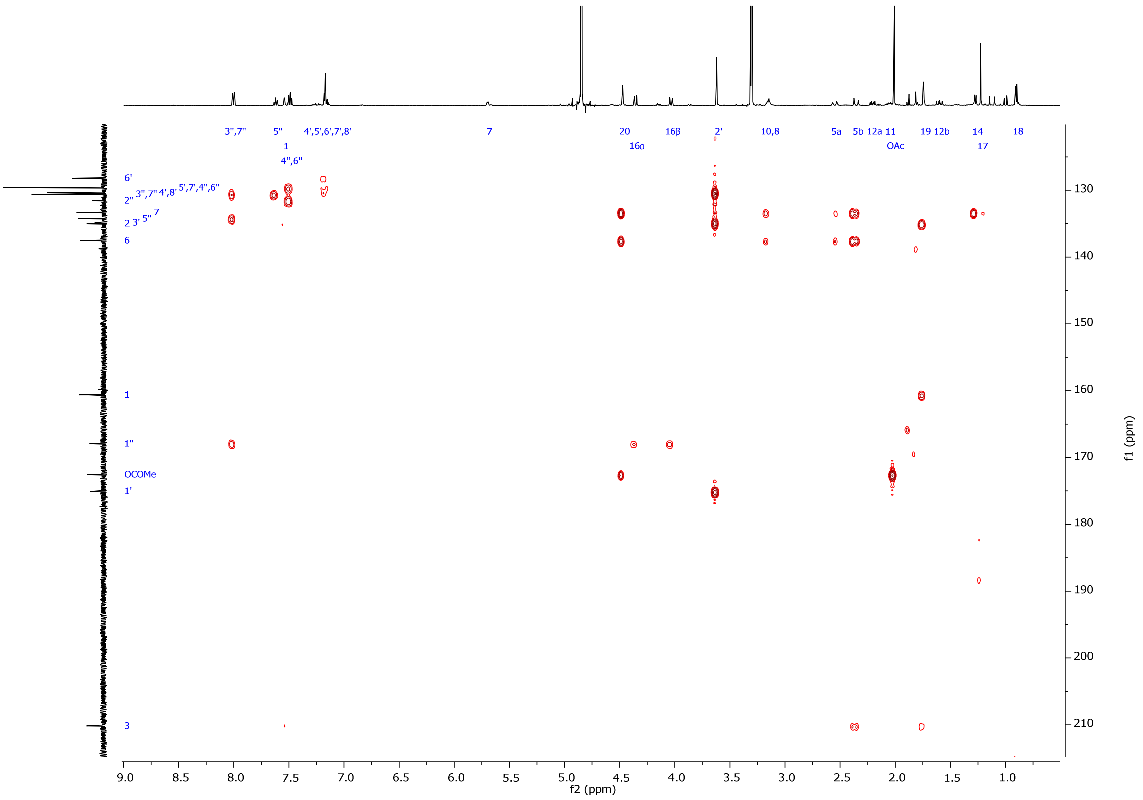

Figure S6i. Expansion (δ_H 9.0-0.5, δ_C 215-120) of HMBC spectrum of AcDPPBz (**6**) in CD_3OD.

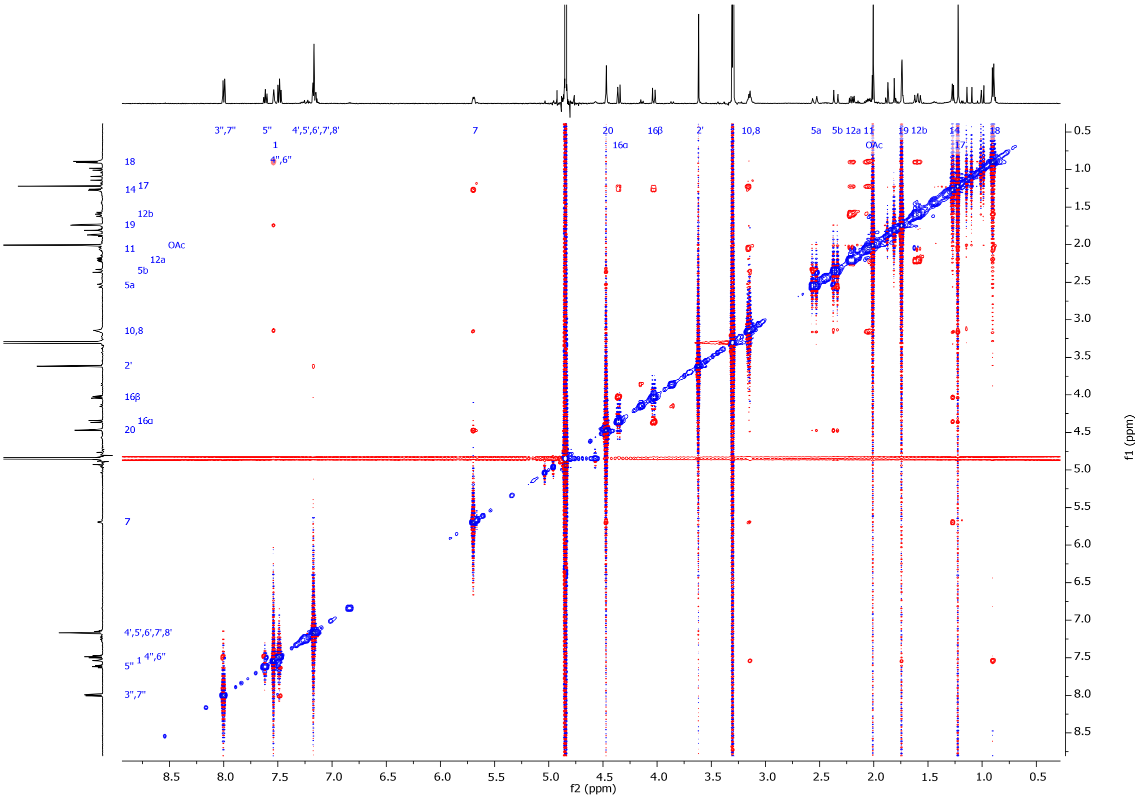

Figure S6j. NOESY2D spectrum of AcDPPBz (**6**) in CD_3OD.

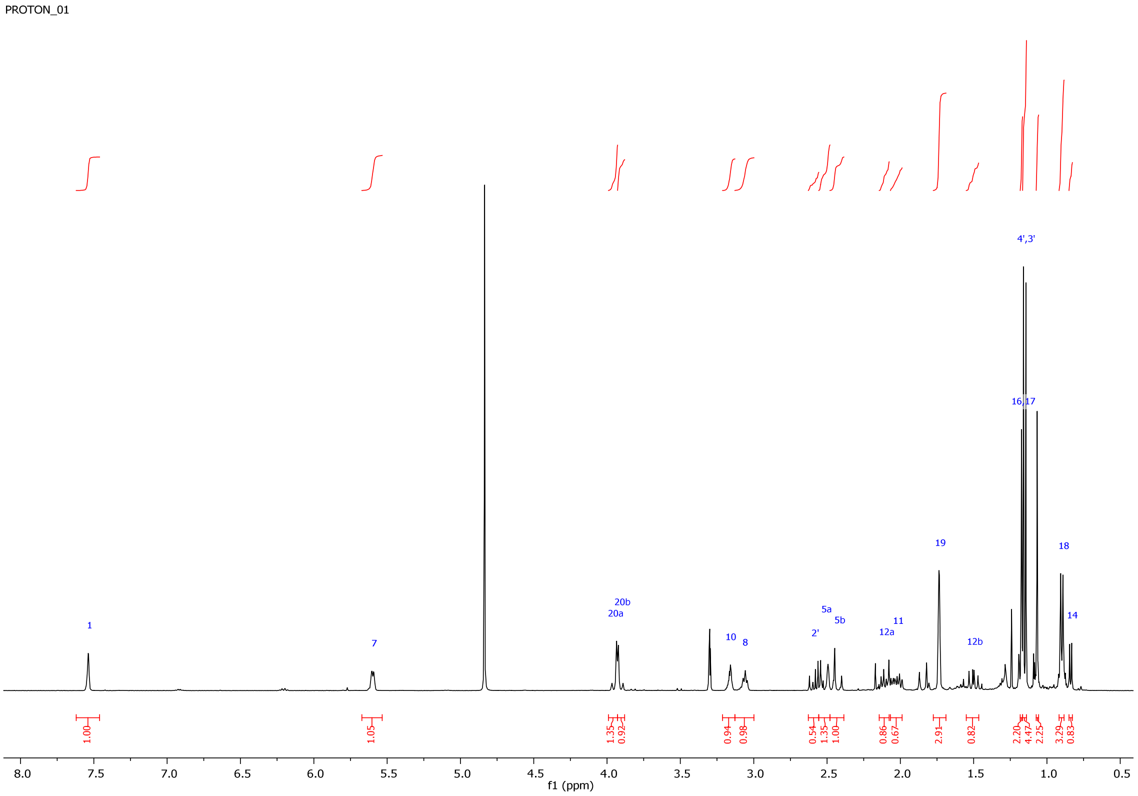

Figure S7a. ^{1}H-NMR spectrum of DPB (**7**) in $CDCl_3$ (400 MHz).

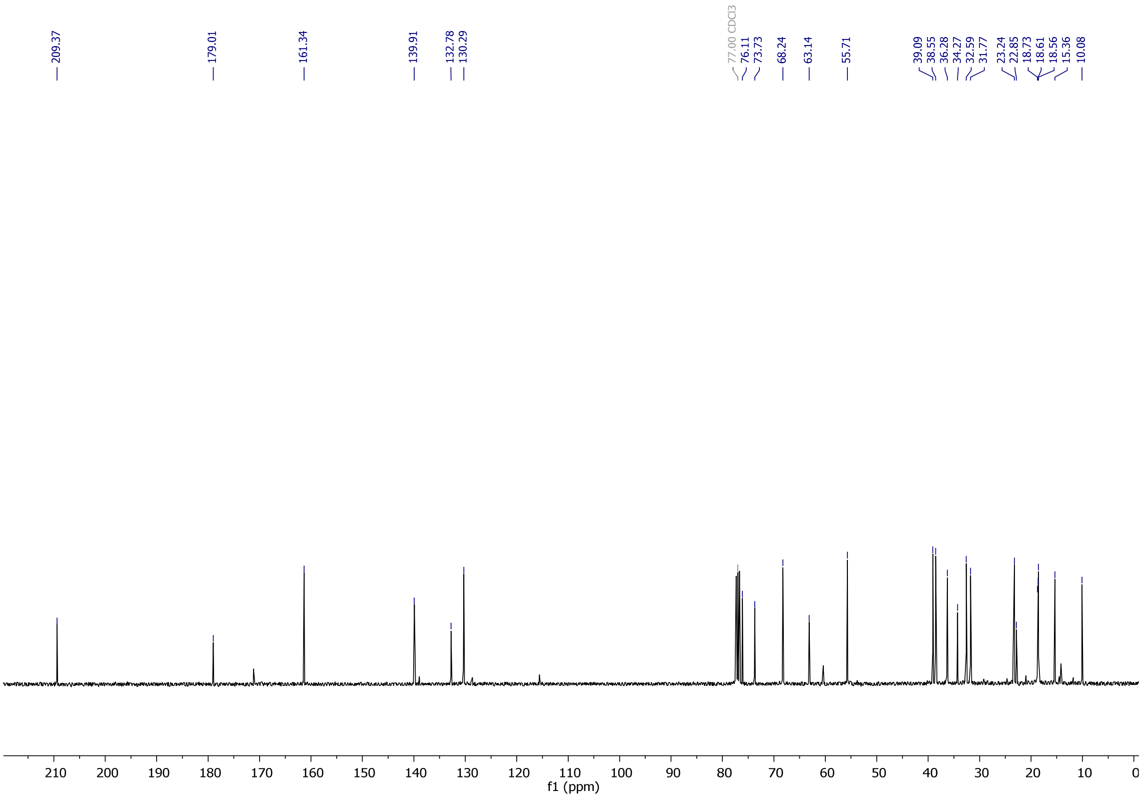

Figure S7b. ^{13}C-NMR spectrum of DPB (**7**) in $CDCl_3$ (100 MHz).

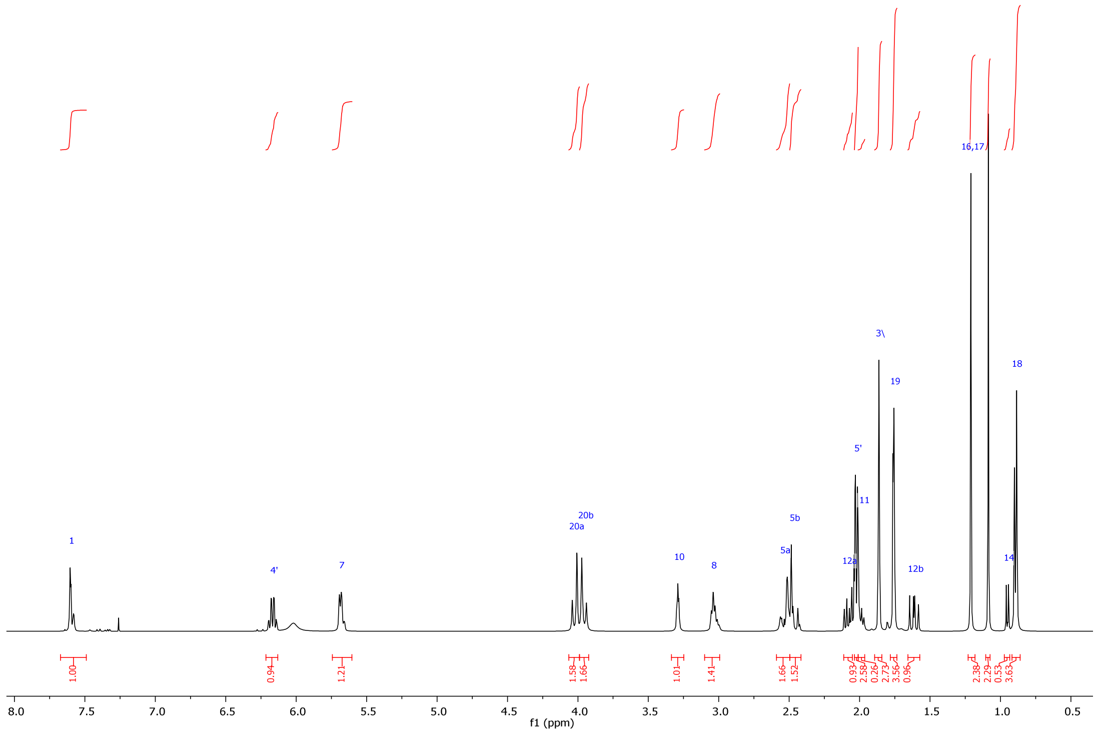

Figure S8a. ^{1}H-NMR spectrum of DPA (**8**) in $CDCl_3$ (400 MHz).

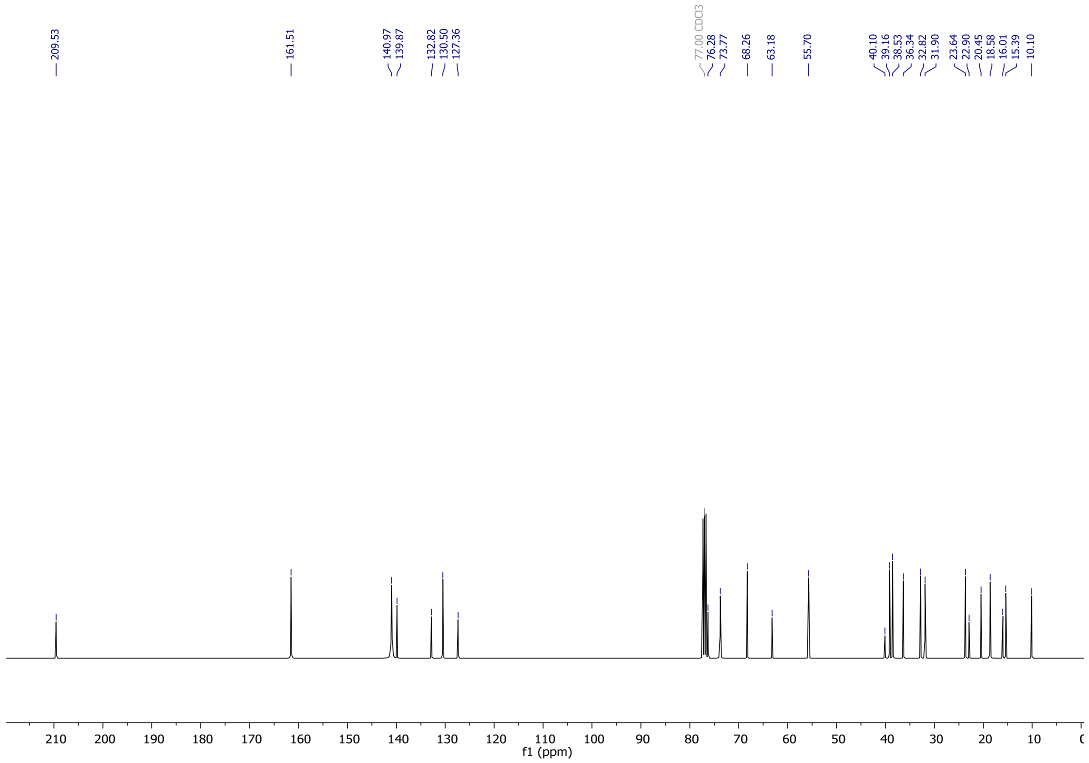

Figure S8b. ^{13}C-NMR spectrum of DPA (**8**) in $CDCl_3$ (100 MHz).

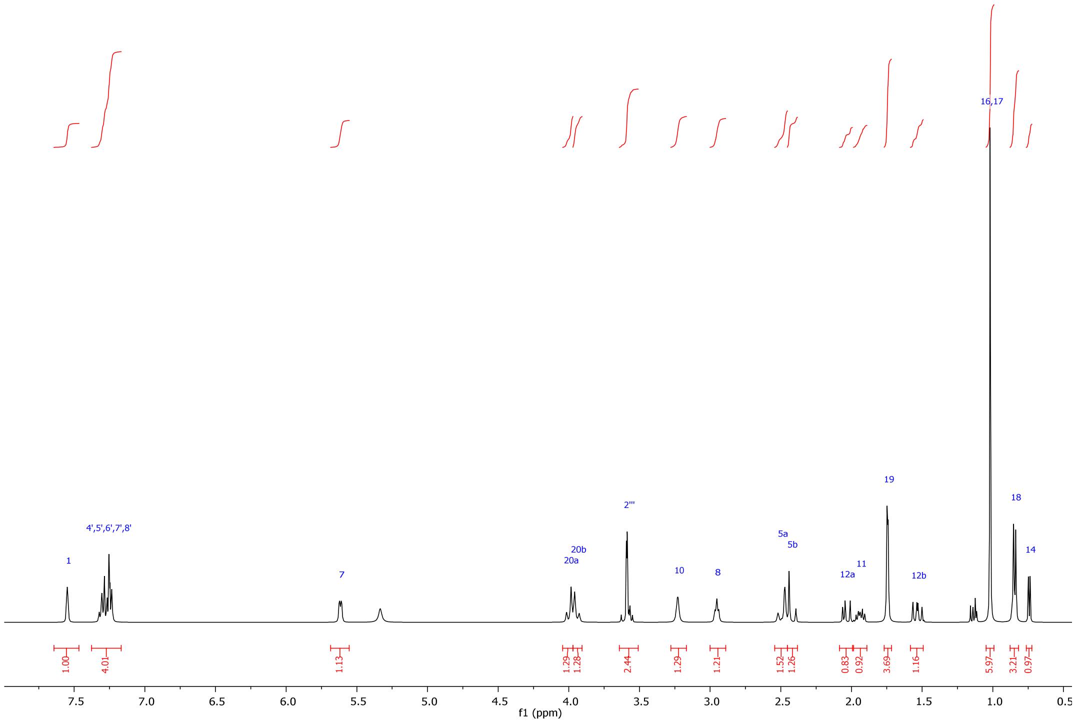

Figure S9a. ^{1}H-NMR spectrum of DPP (**9**) in $CDCl_3$ (400 MHz).

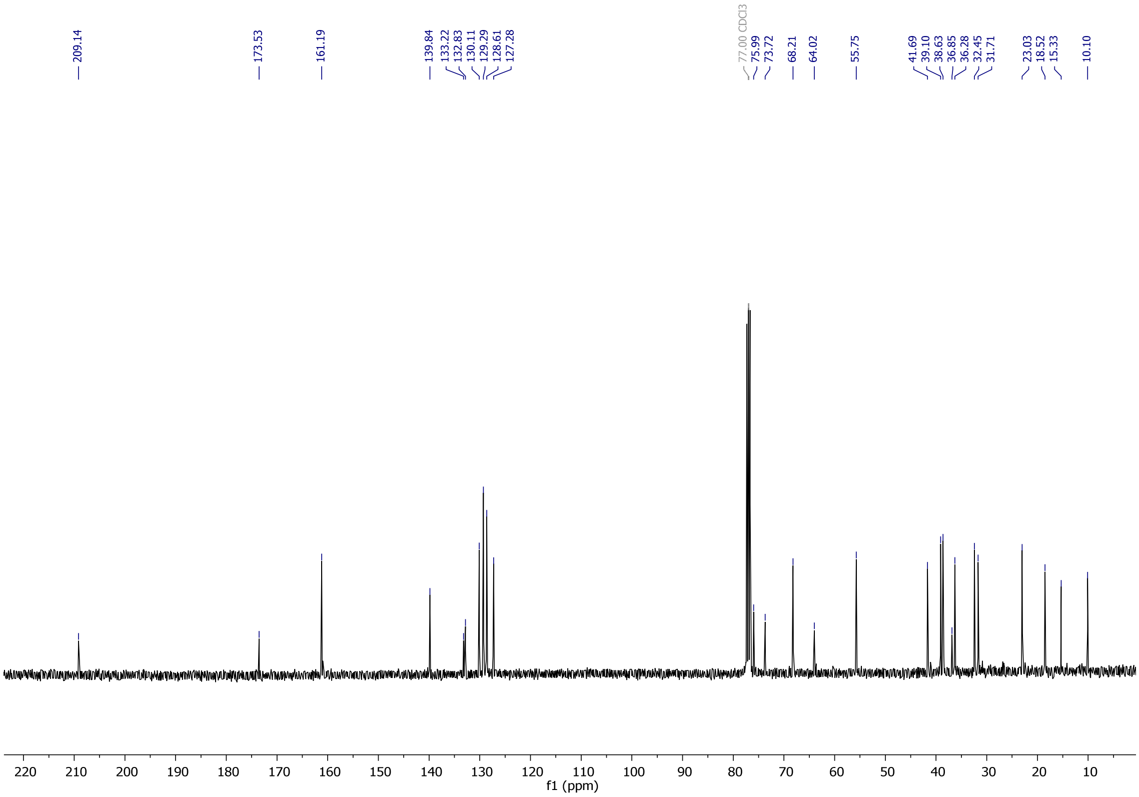

Figure S9b. ^{13}C-NMR of DPP (**9**) in $CDCl_3$ (100 MHz).

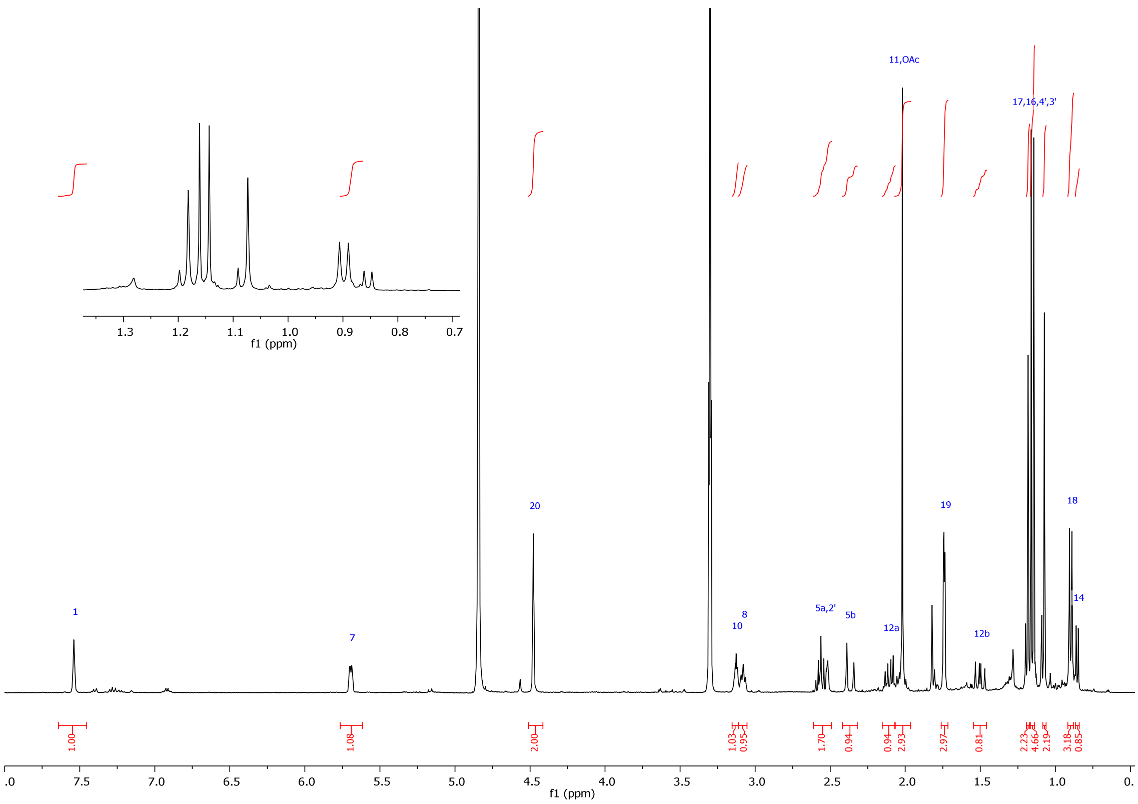

Figure S10a. ^{1}H-NMR spectrum of AcDPB (**10**) in CD_3OD (400 MHz).

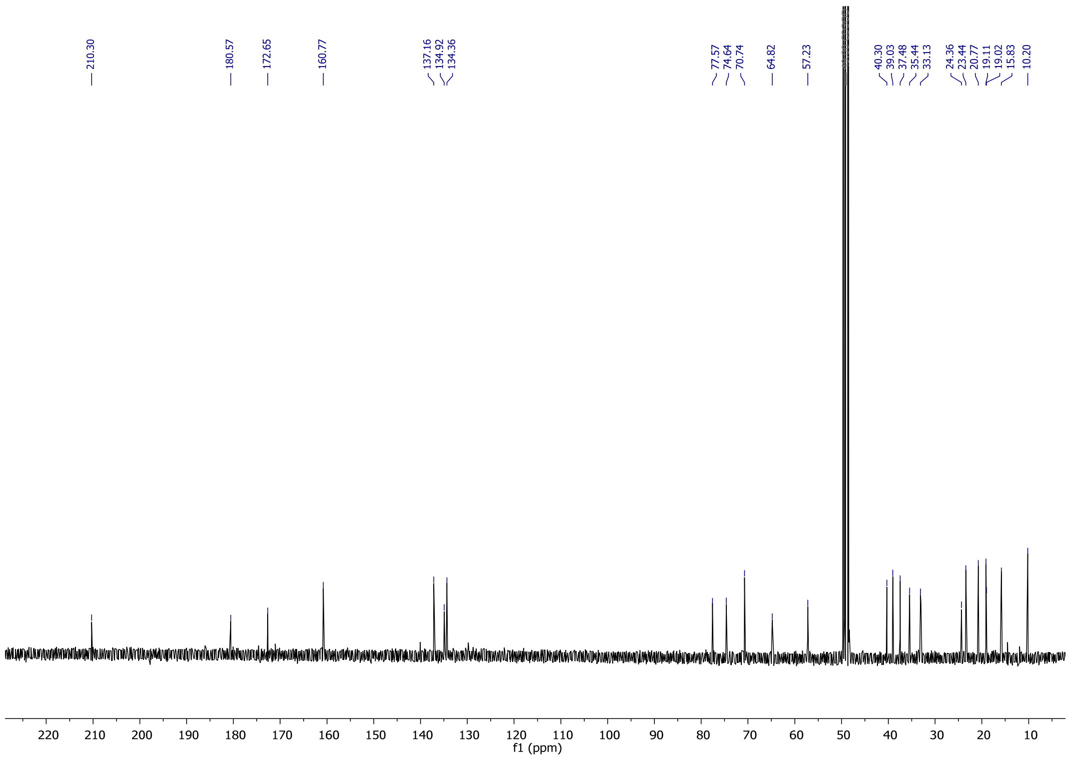

Figure S10b. ^{13}C-NMR spectrum of AcDPB (**10**) in CD_3OD (100 MHz).

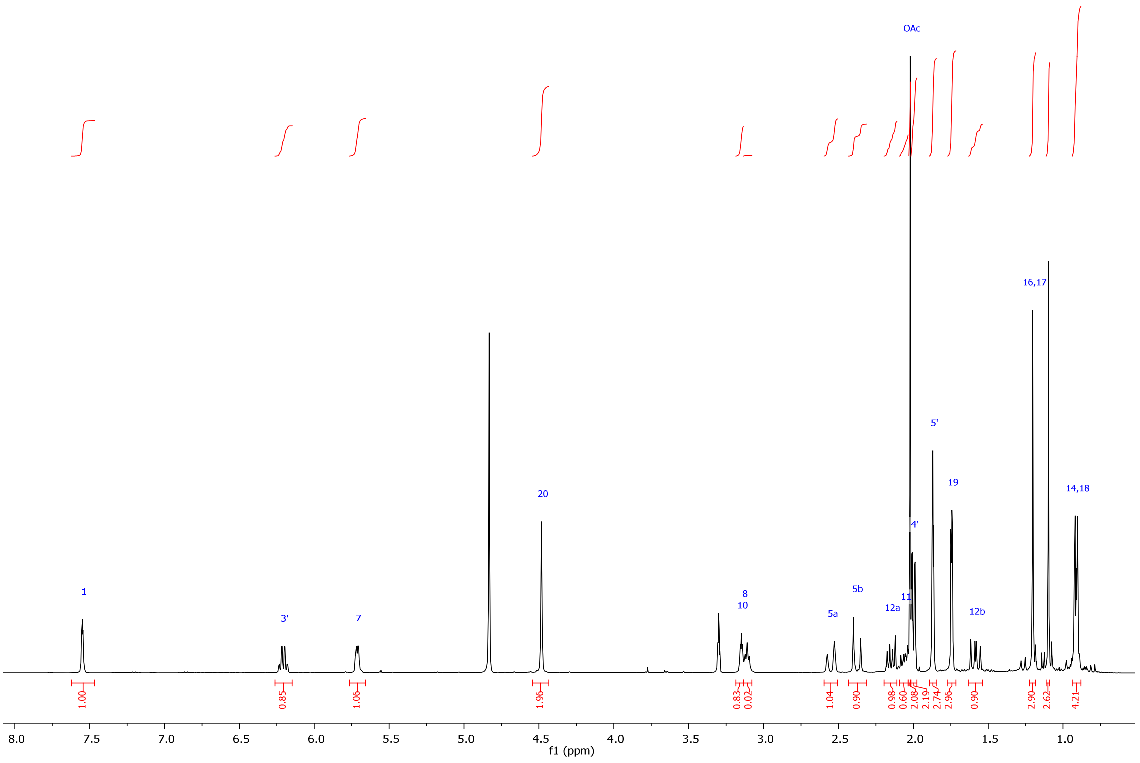

Figure S11a. ^{1}H-NMR spectrum AcDPA (**11**) in CD_3OD (400 MHz).

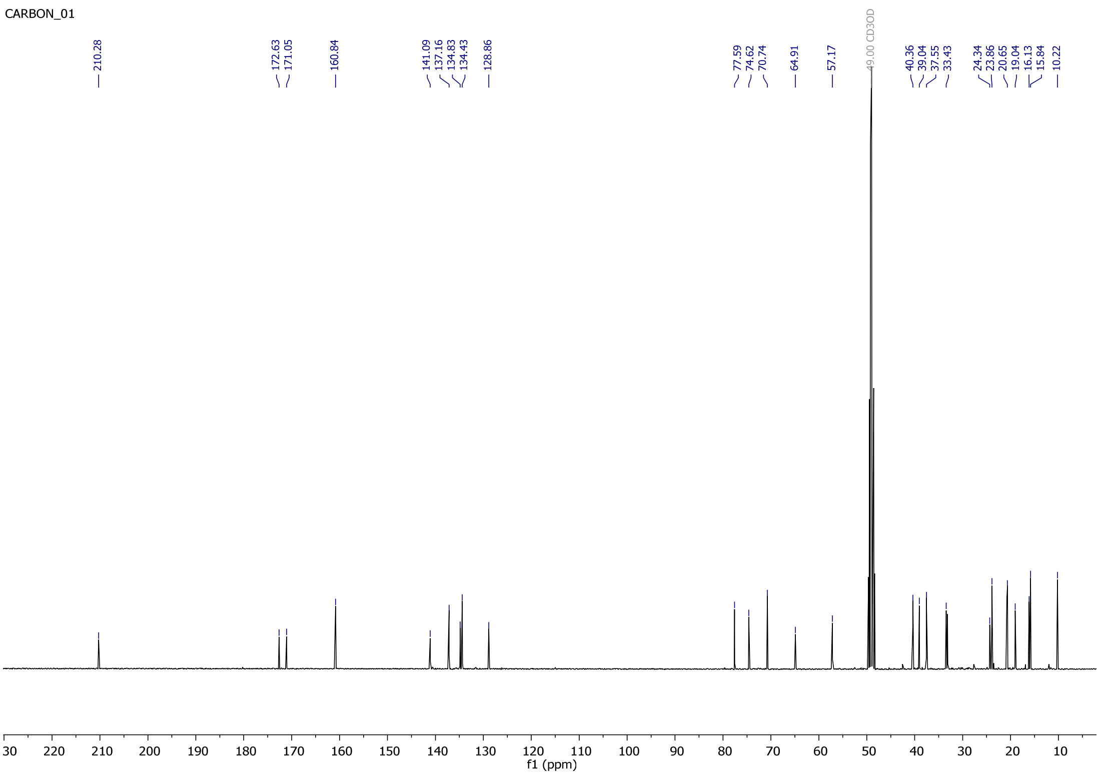

Figure S11b. ^{13}C-NMR spectrum of AcDPA (**11**) in CD_3OD (100 MHz).

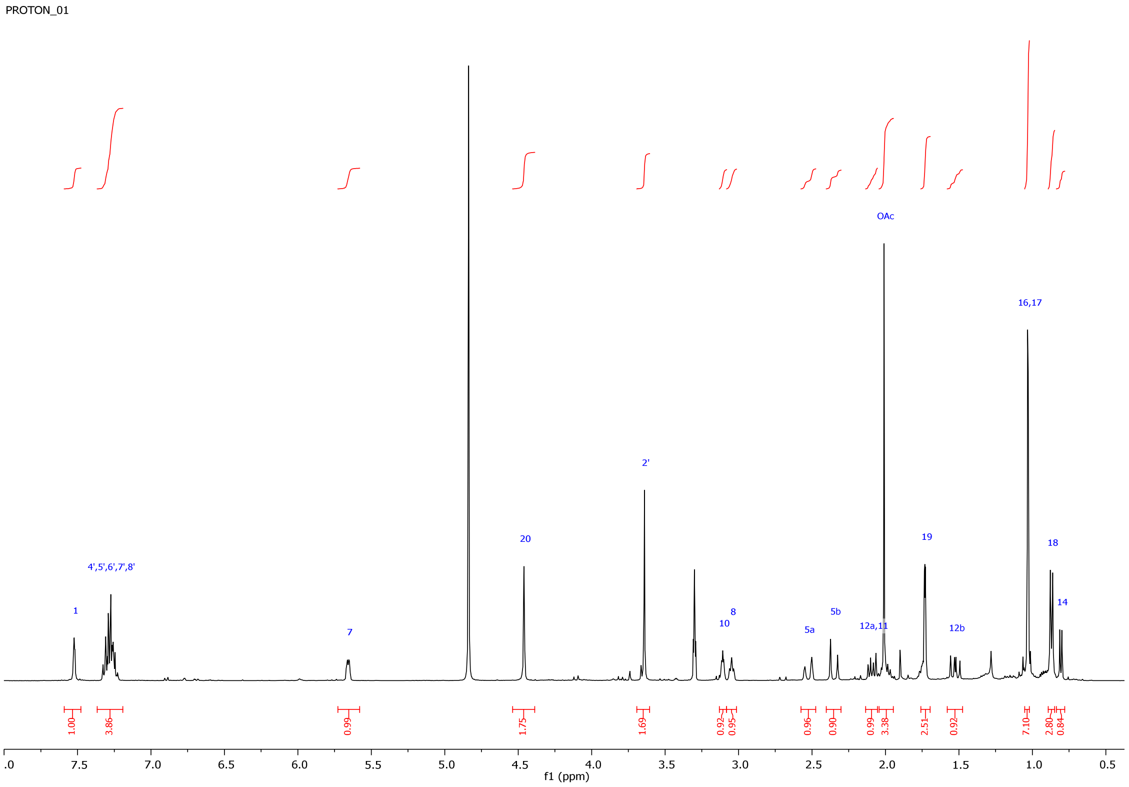

Figure S12a. ^{1}H-NMR spectrum of AcDPP (**12**) in CD_3OD (400 MHz).

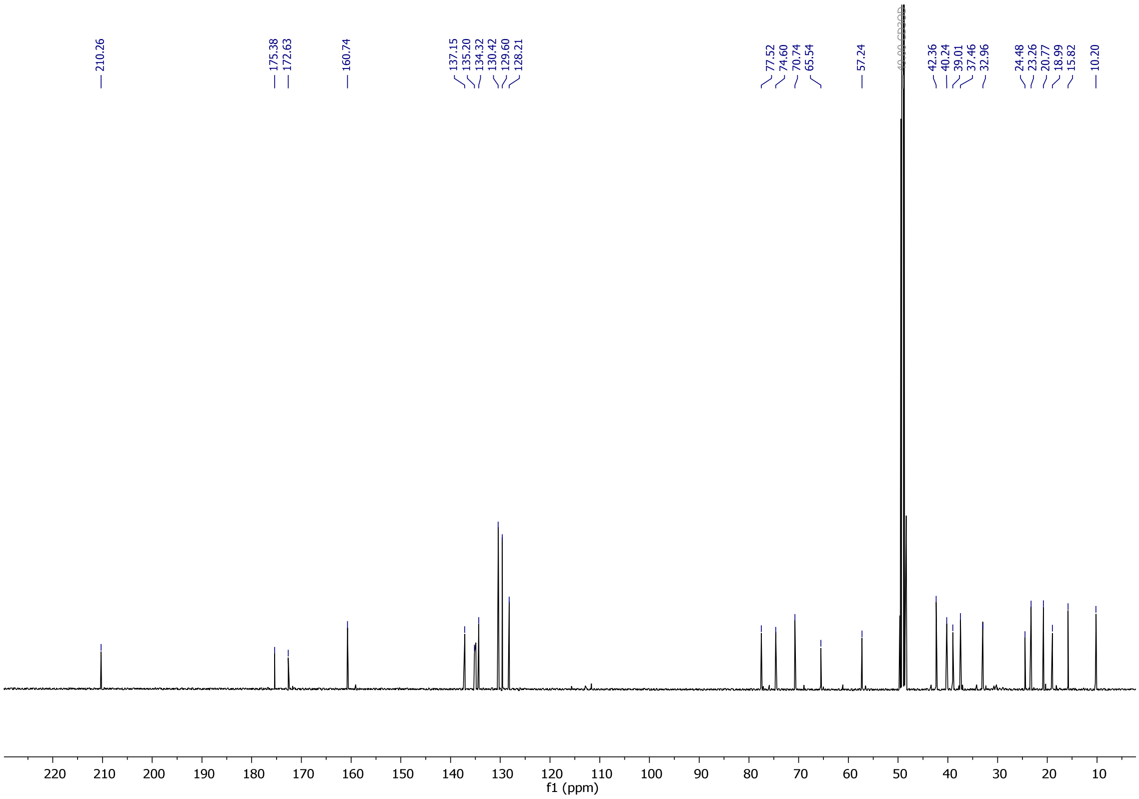

Figure S12b. ^{13}C-NMR spectrum of AcDPP (**12**) in CD_3OD (100 MHz).

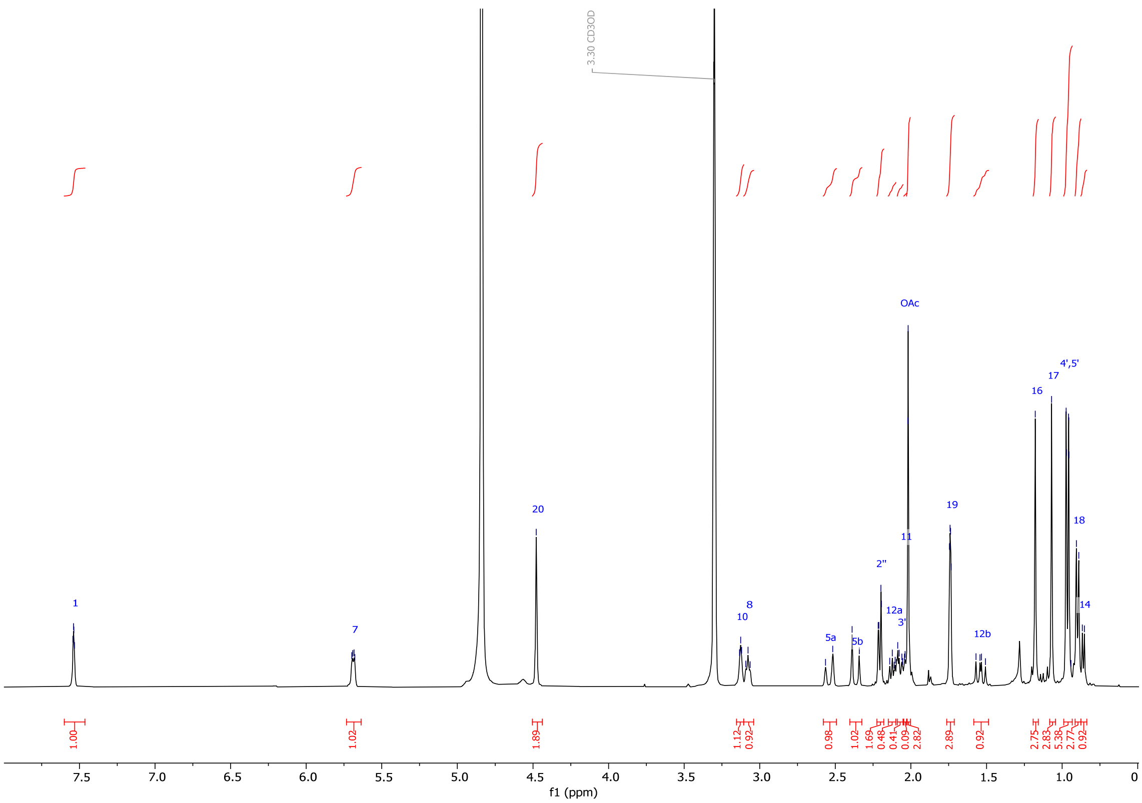

Figure S13a. ^{1}H-NMR spectrum of AcDPiPn (**13**) in CD_3OD (500 MHz).

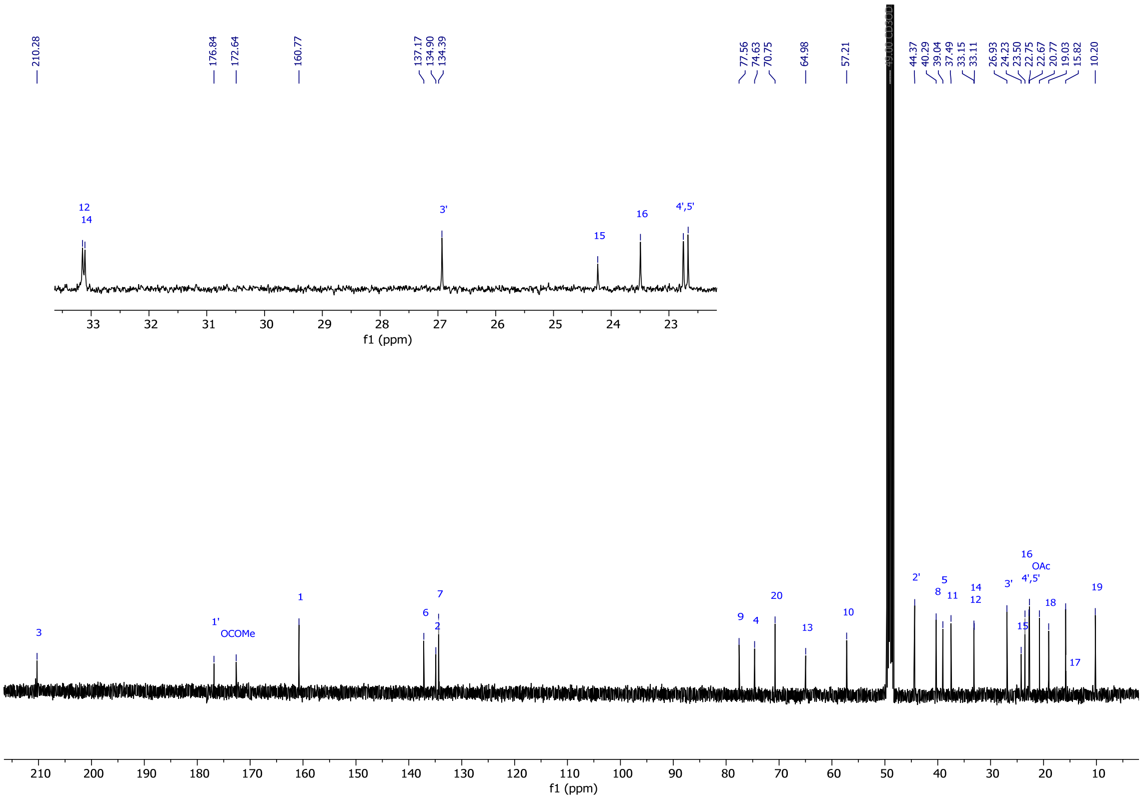

Figure S13b. ^{13}C-NMR spectrum of AcDPiPn (**13**) in CD_3OD (125 MHz).

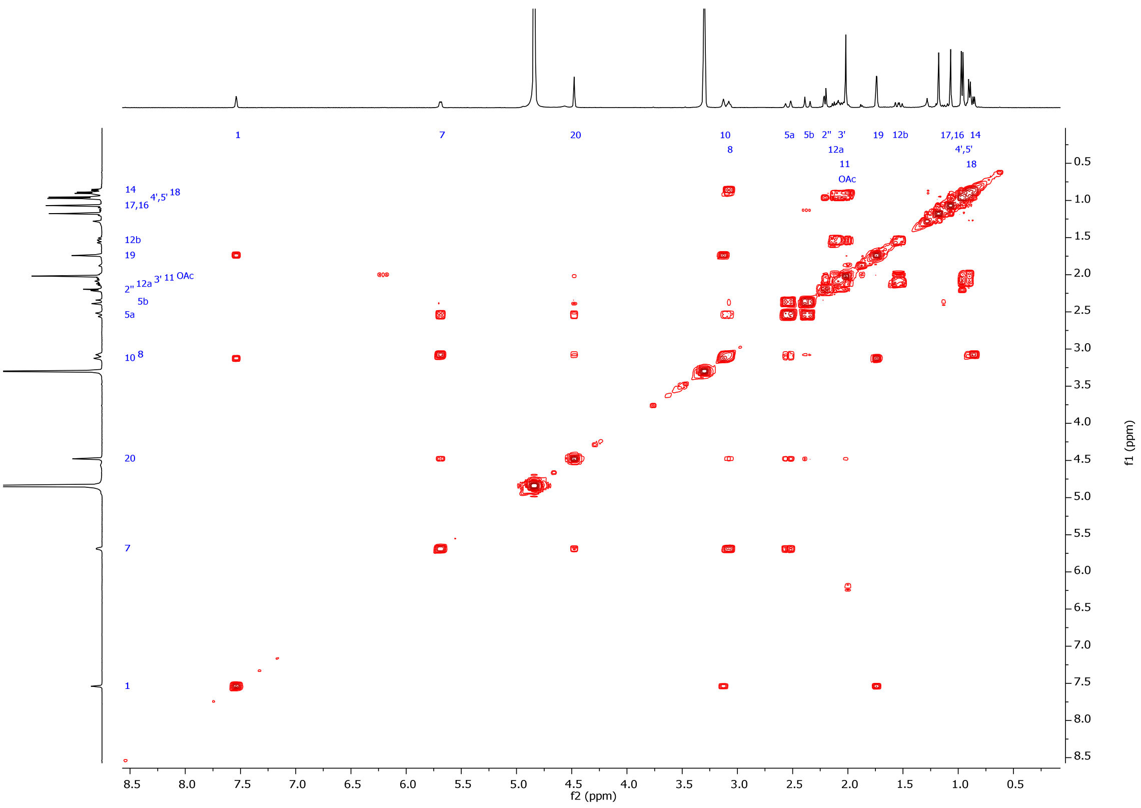

Figure S13c. COSY spectrum of AcDPiPn (**13**) in CD_3OD.

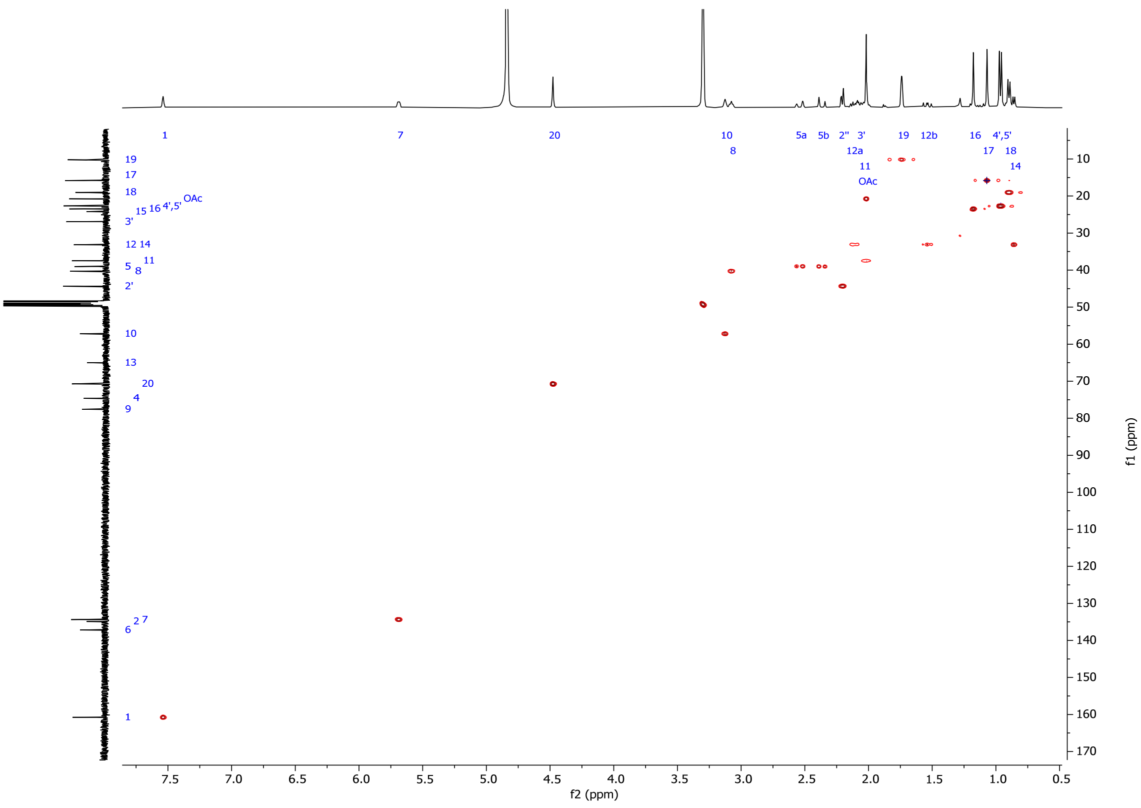

Figure S13d. HSQC spectrum of AcDPiPn (**13**) in CD_3OD.

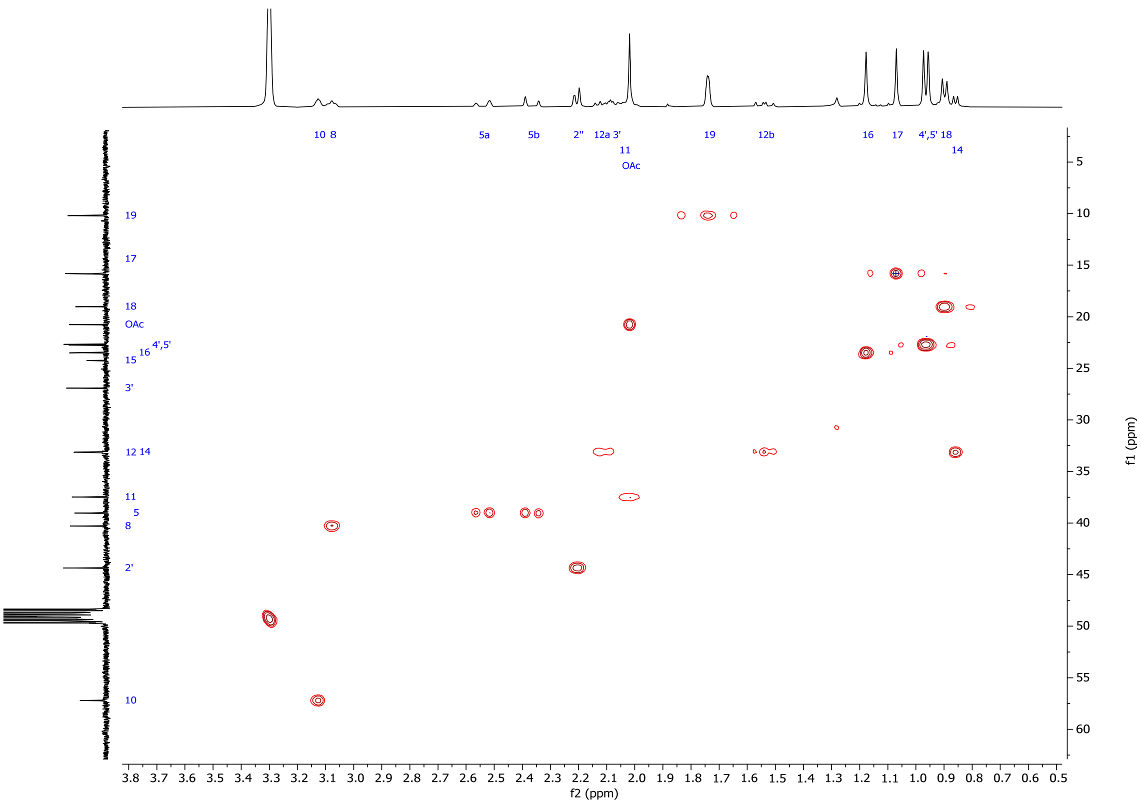

Figure S13e. Expansion (δ_H 3.8-0.2, δ_C 62-3) of HSQC spectrum of AcDPiPn (**13**) in CD_3OD.

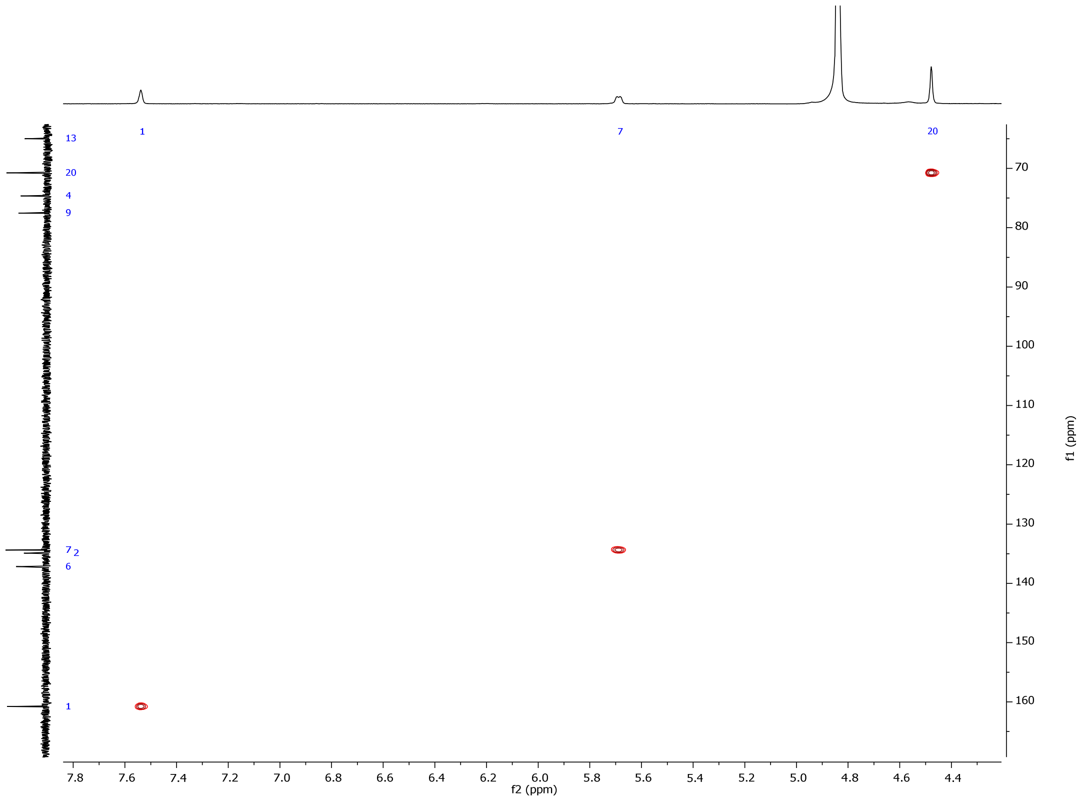

Figure S13f. Expansion (δ_H 7.8-4.2, δ_C 170-65) of HSQC spectrum of AcDPiPn (**13**) in CD_3OD.

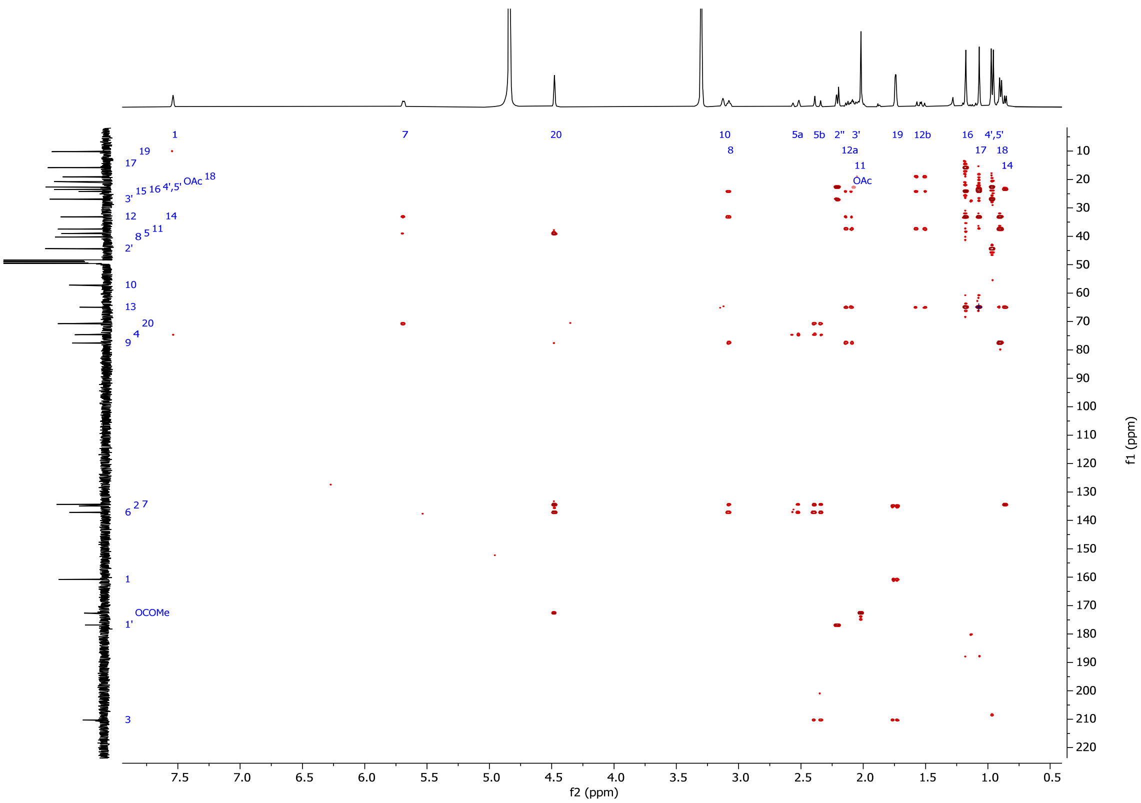

Figure S13g. HMBC spectrum of AcDPiPn (**13**) in CD_3OD.

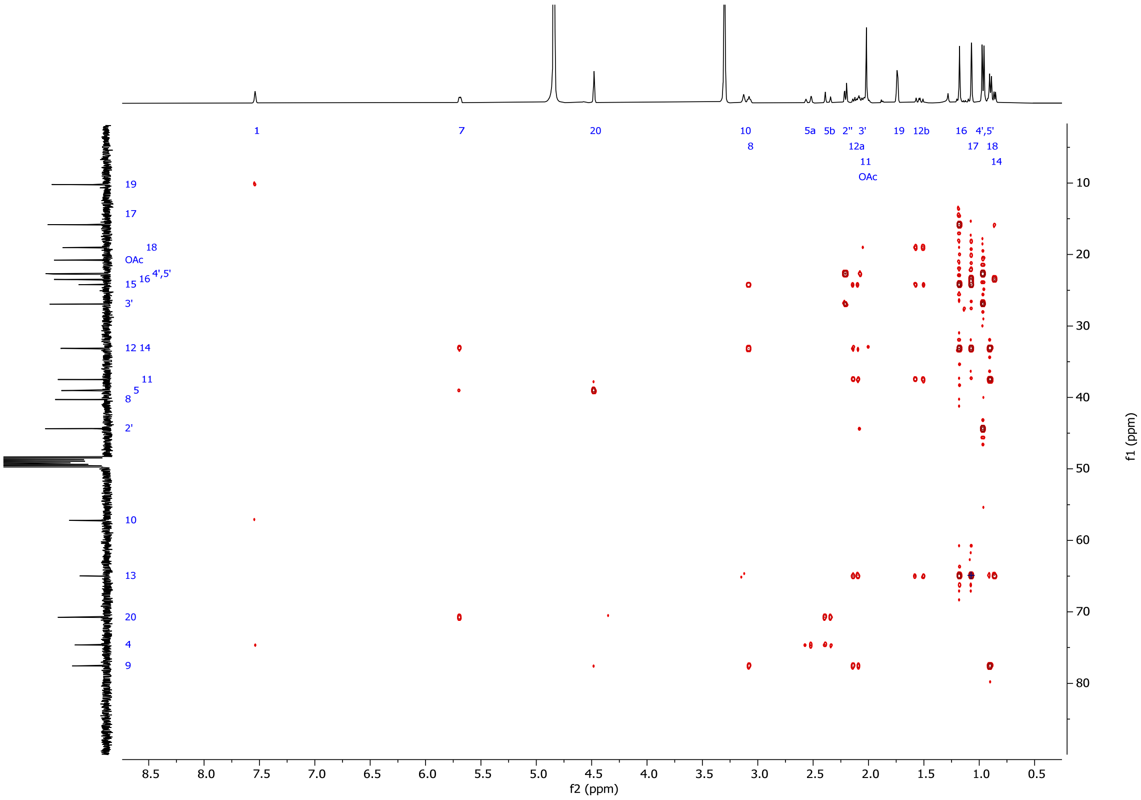

Figure S13h. Expansion (δ_H 8.7-0.2, δ_C 90-3) of HMBC spectrum of AcDPiPn (**13**) in CD_3OD.

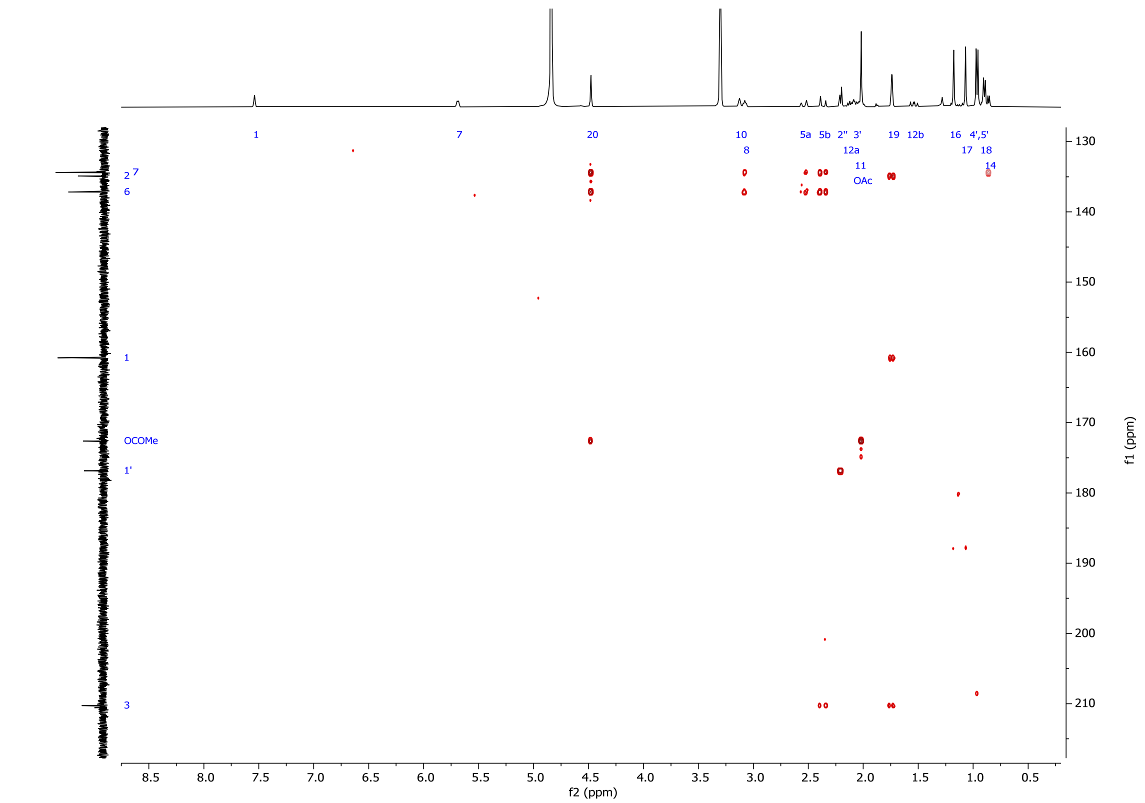

Figure S13i. Expansion (δ_H 8.7-0.2, δ_C 215-128) of HMBC spectrum of AcDPiPn (**13**) in CD_3OD.

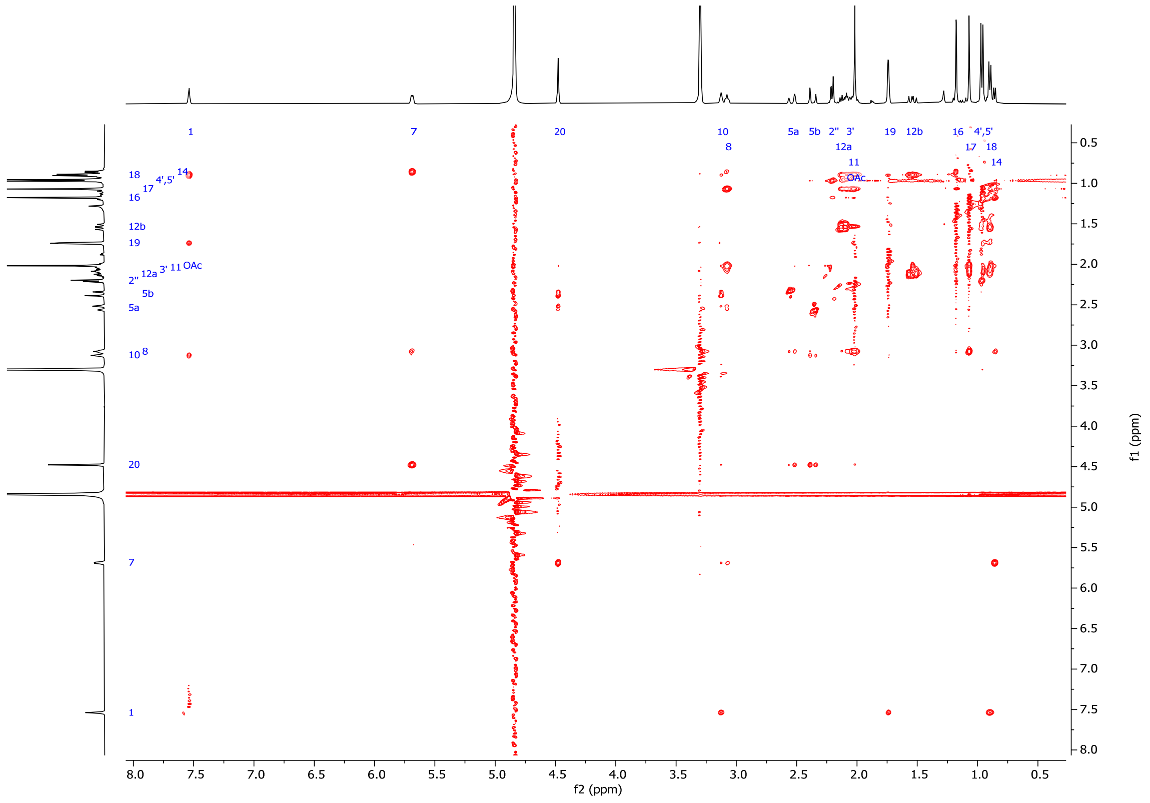

Figure S13j. NOESY2D spectrum of AcDPiPn (**13**) in CD_3OD.

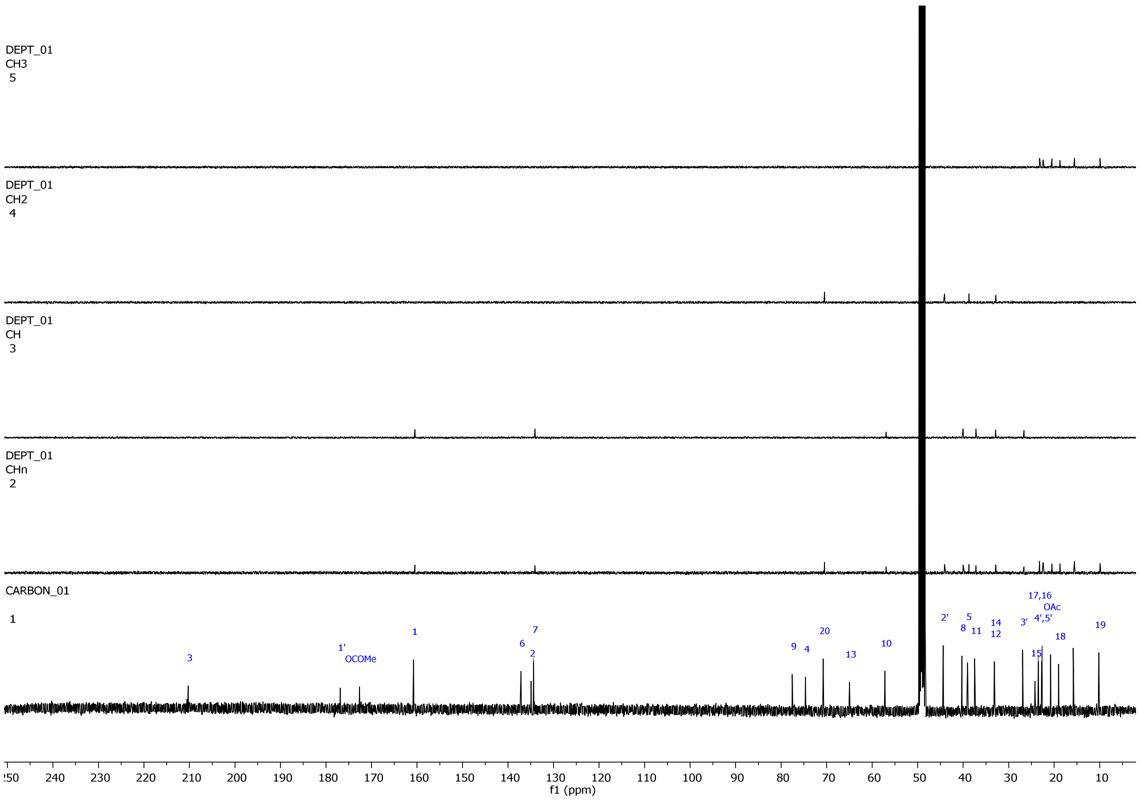

Figure S13k. DEPT spectrum of AcDPiPn (**13**) in CD_3OD.

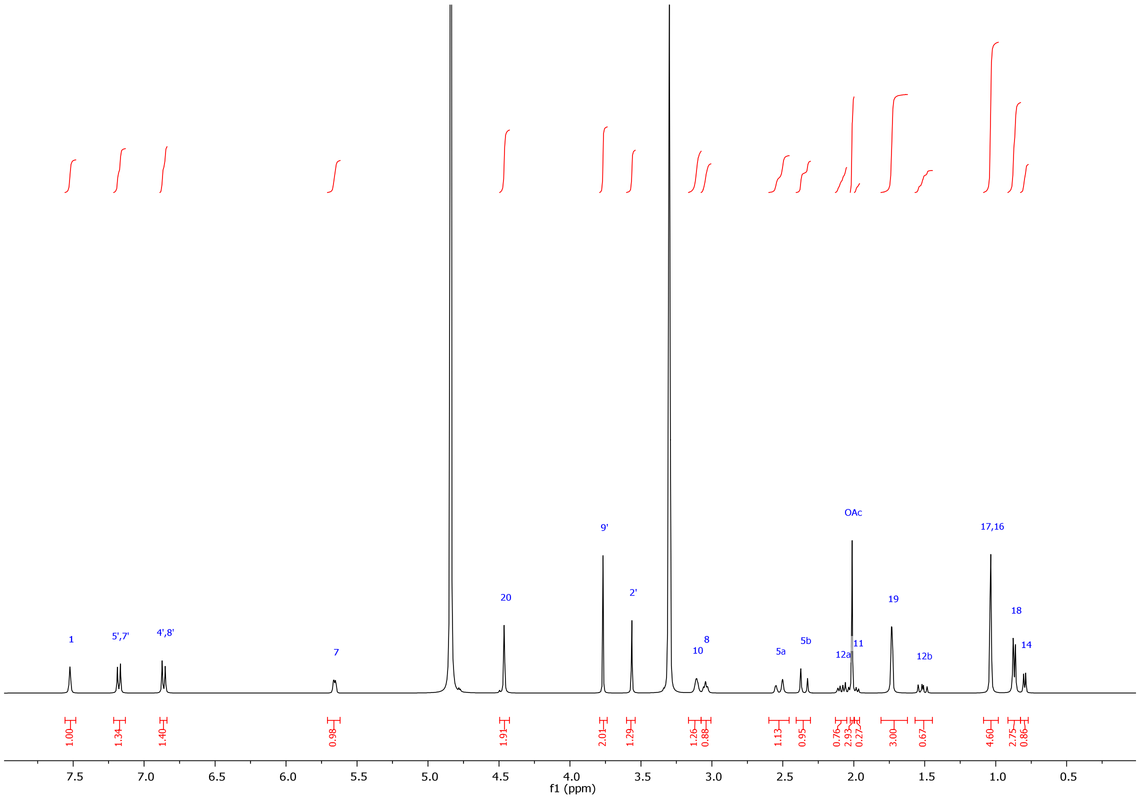

Figure S14a. ^{1}H-NMR spectrum of AcDPMeOP (**14**) in CD_3OD (400 MHz).

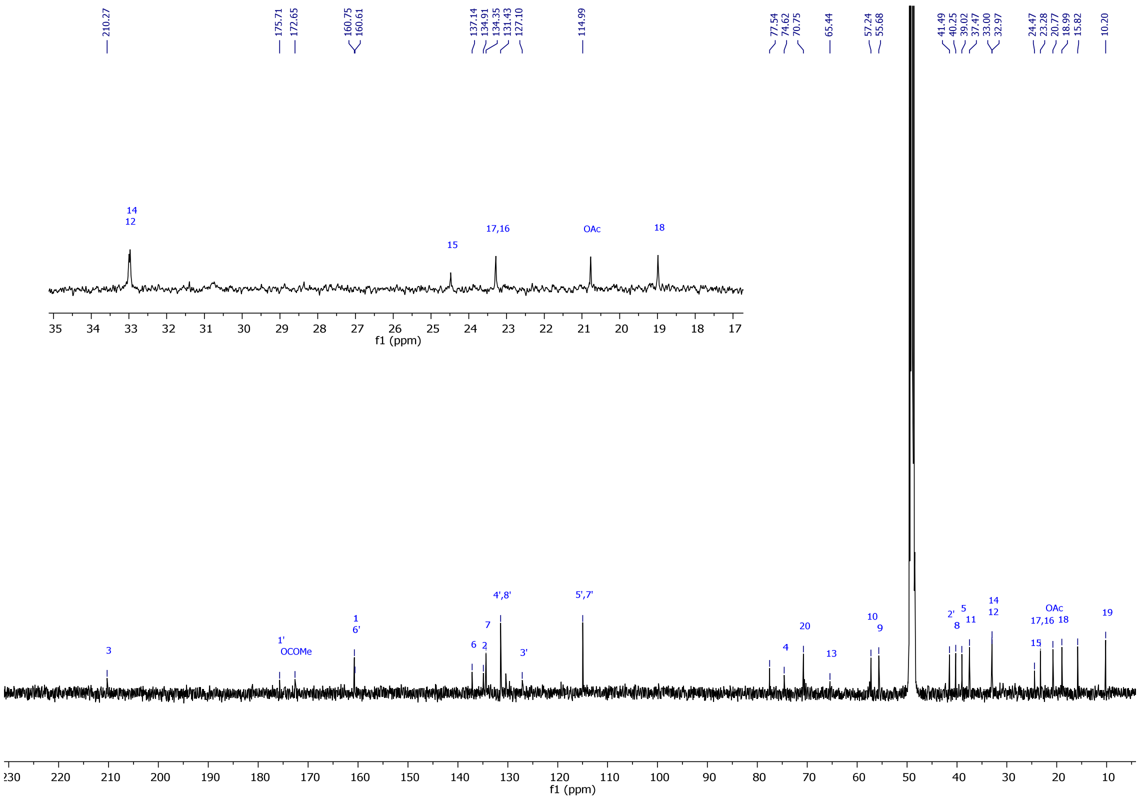

Figure S14b. ^{13}C-NMR spectrum of AcDPMeOP (**14**) in CD_3OD (100 MHz).

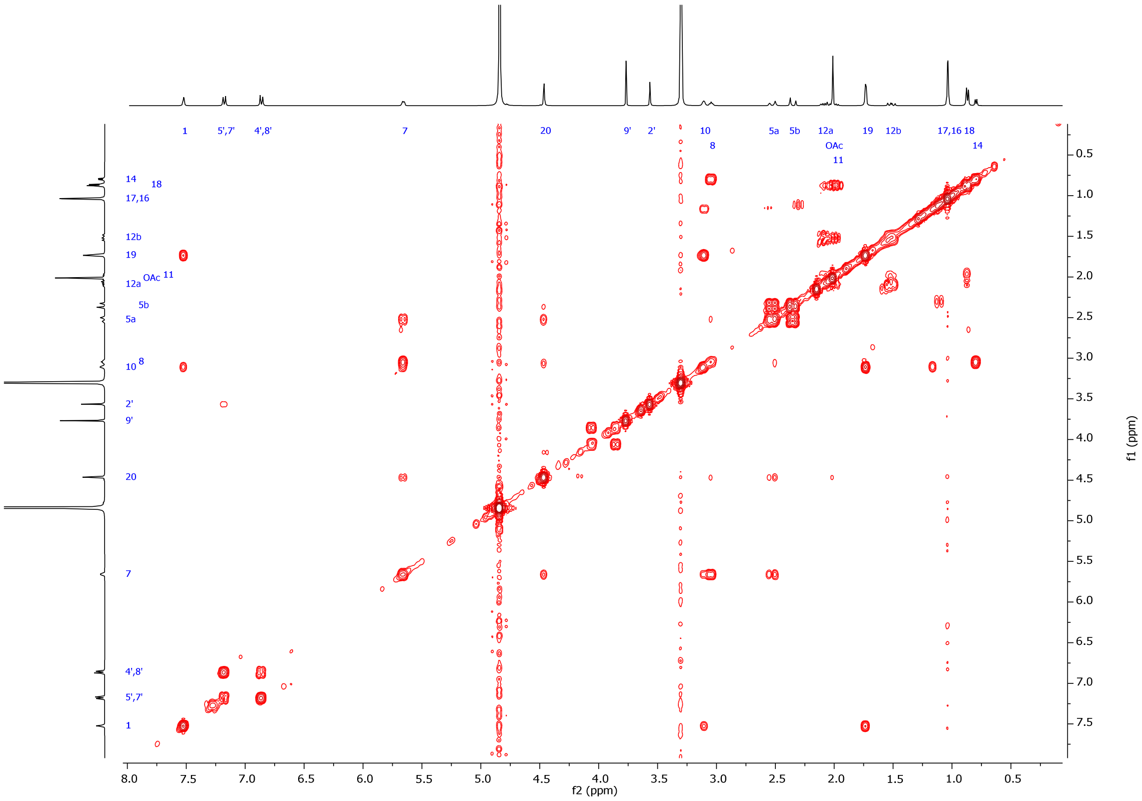

Figure S14c. COSY spectrum of AcDPMeOP (**14**) in CD_3OD.

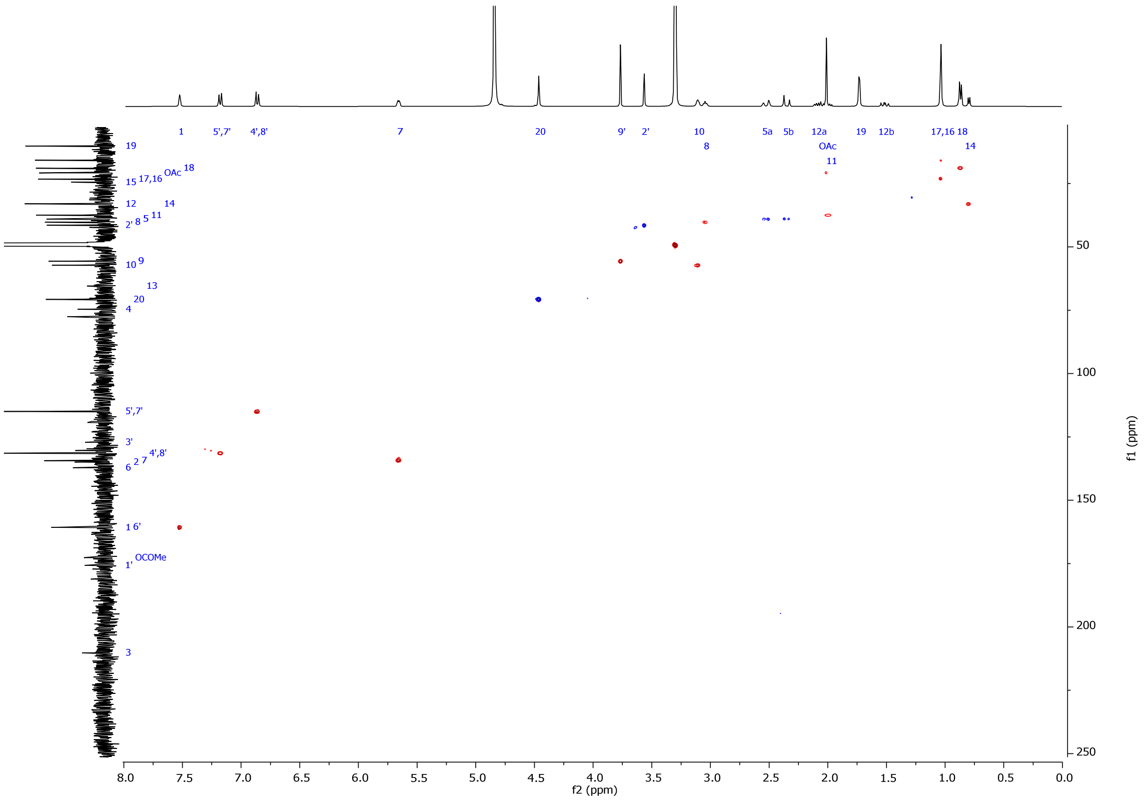

Figure S14d. HSQC spectrum of AcDPMeOP (**14**) in CD_3OD.

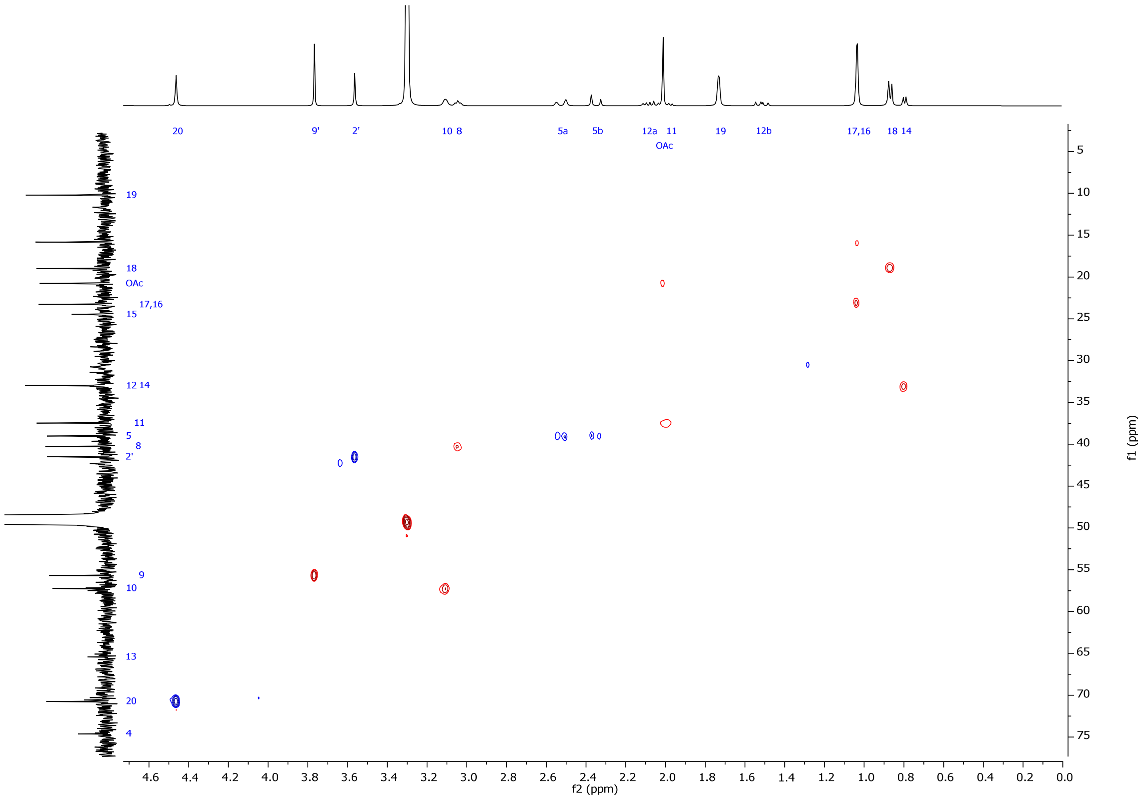

Figure S14e. Expansion (δ_H 4.7-0.1, δ_C 77-2) of HSQC spectrum of AcDPMeOP (**14**) in CD_3OD.

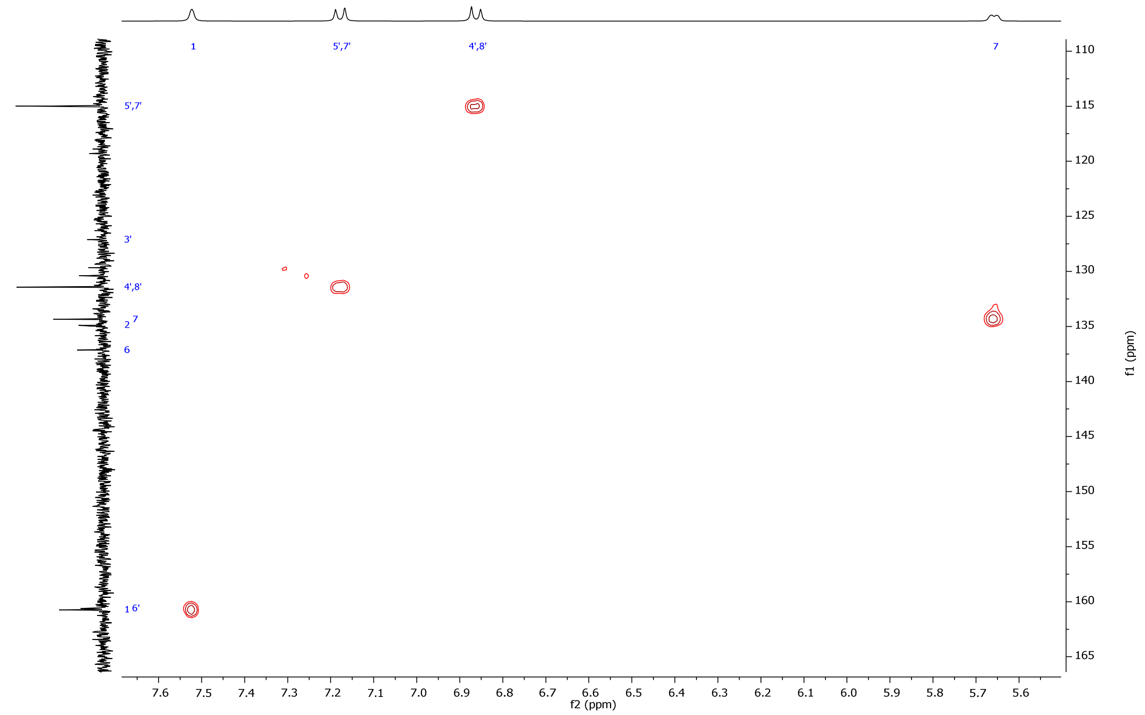

Figure S14f. Expansion (δ_H 7.7-5.5, δ_C 165-110) of HSQC spectrum of AcDPMeOP (**14**) in CD_3OD.

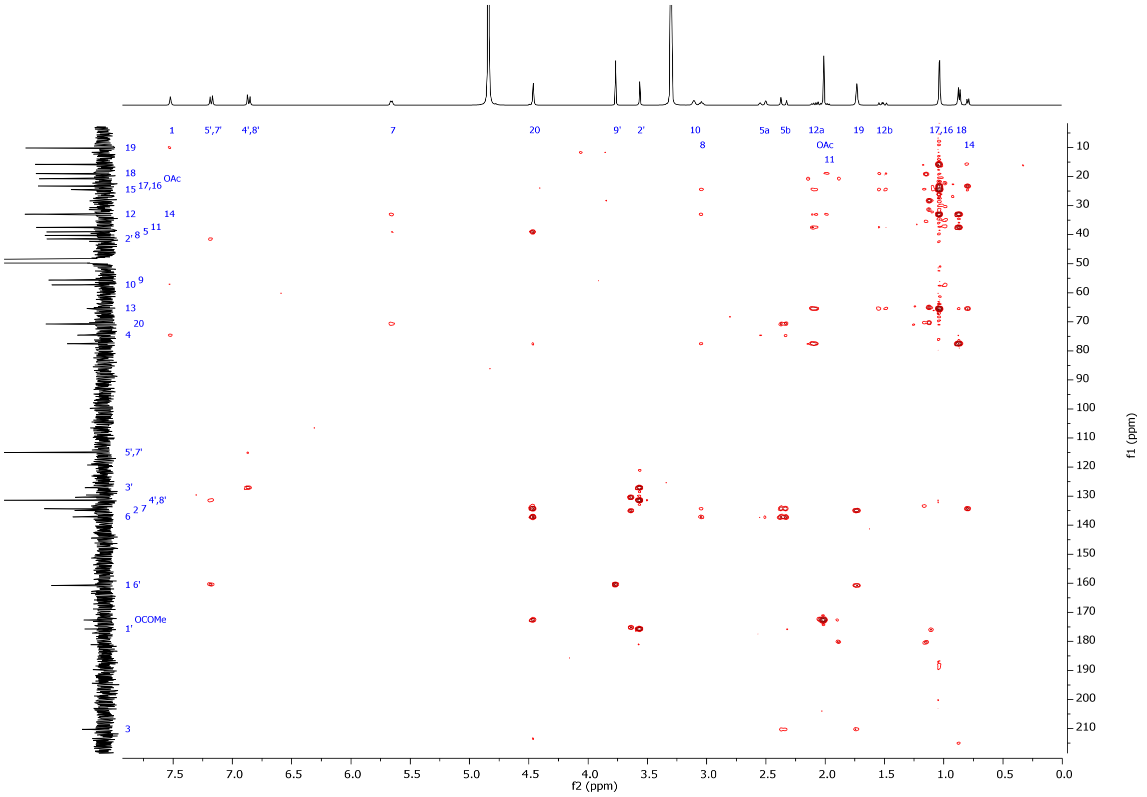

Figure S14g. HMBC spectrum of AcDPMeOP (**14**) in CD_3OD.

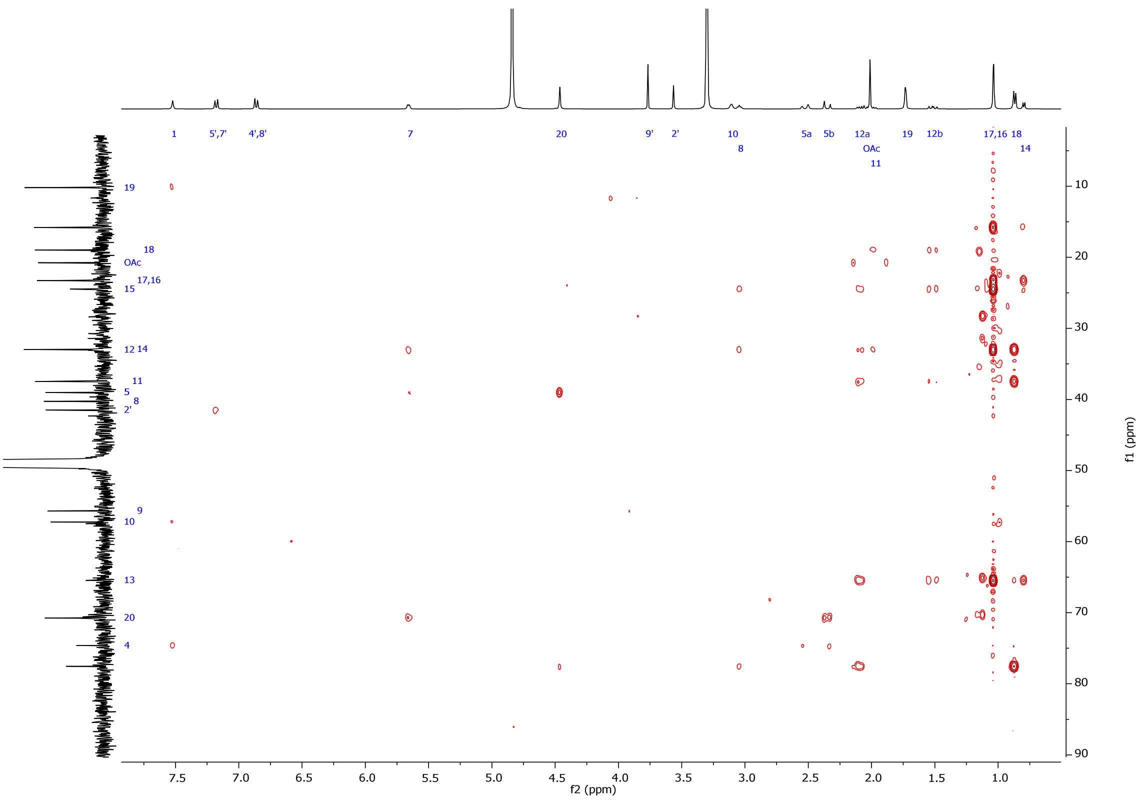

Figure S14h. Expansion (δ_H 7.7-0.5, δ_C 90-3) of HMBC spectrum of AcDPMeOP (**14**) in CD_3OD.

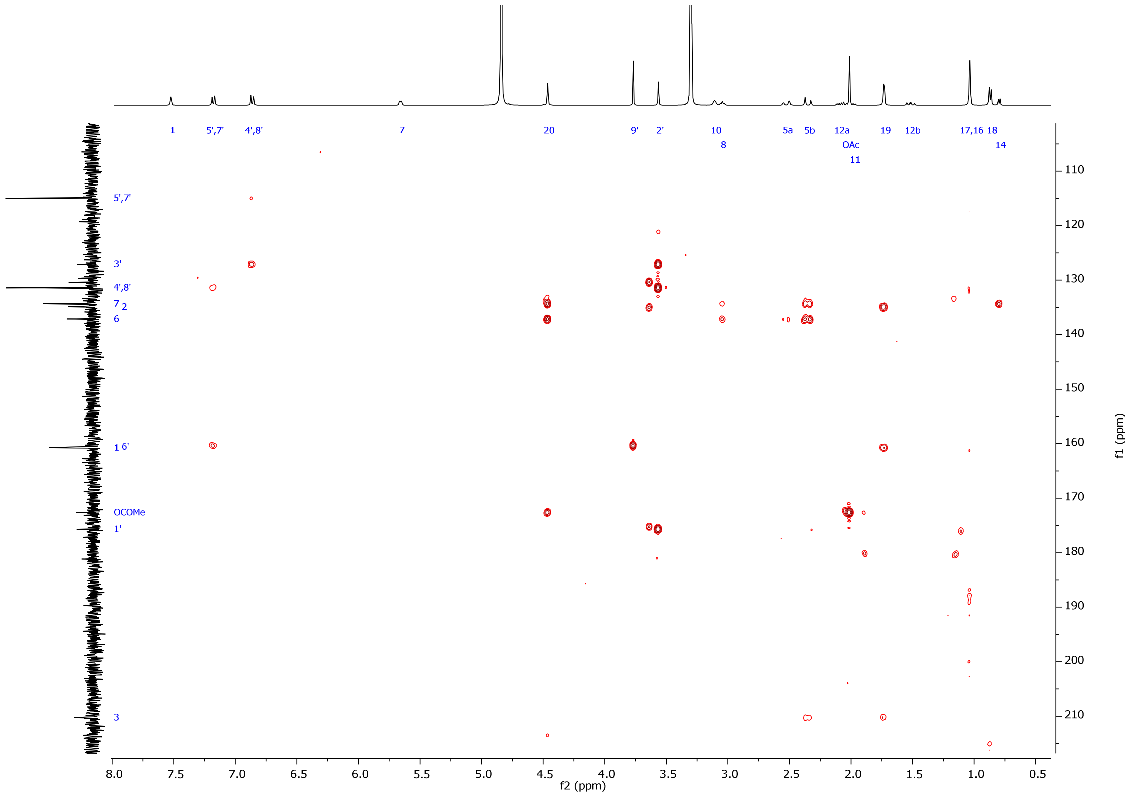

Figure S14i. Expansion (δ_H 8.0-0.5, δ_C 215-105) of HMBC spectrum of AcDPMeOP (**14**) in CD_3OD.

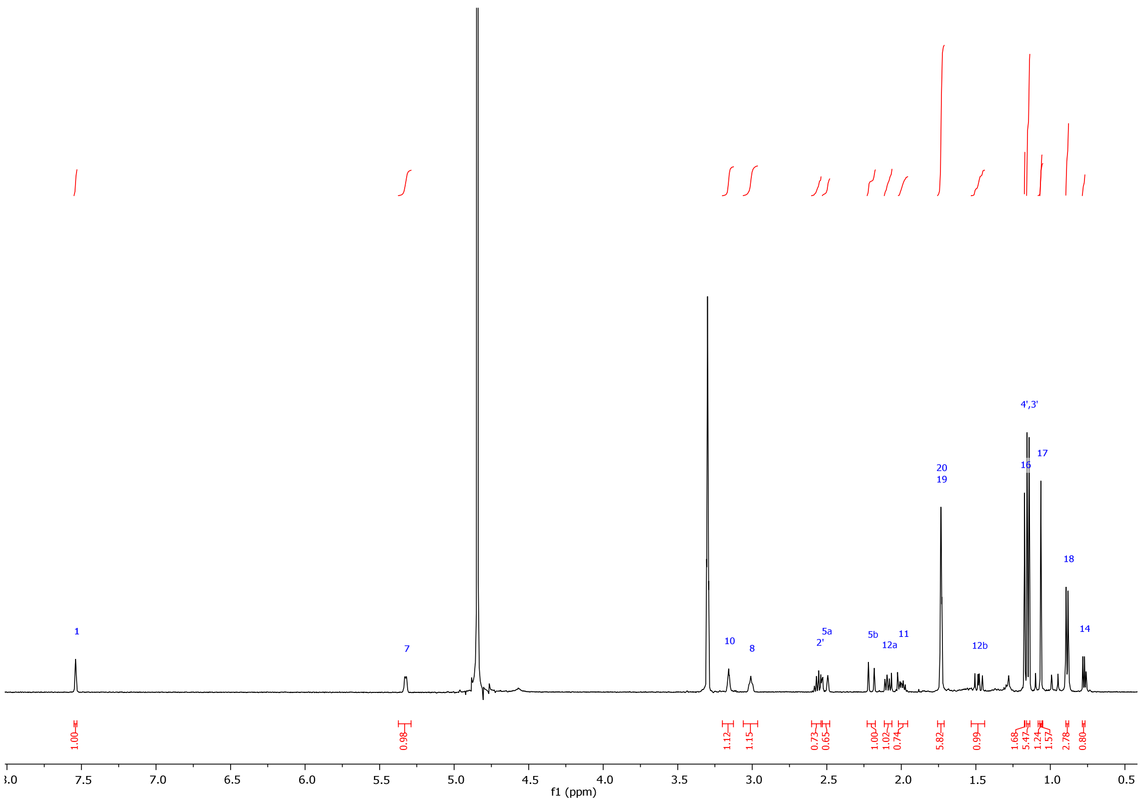

Figure S15a. ^{1}H-NMR spectrum of diDPB (**15**) in CD_3OD (500 mHz).

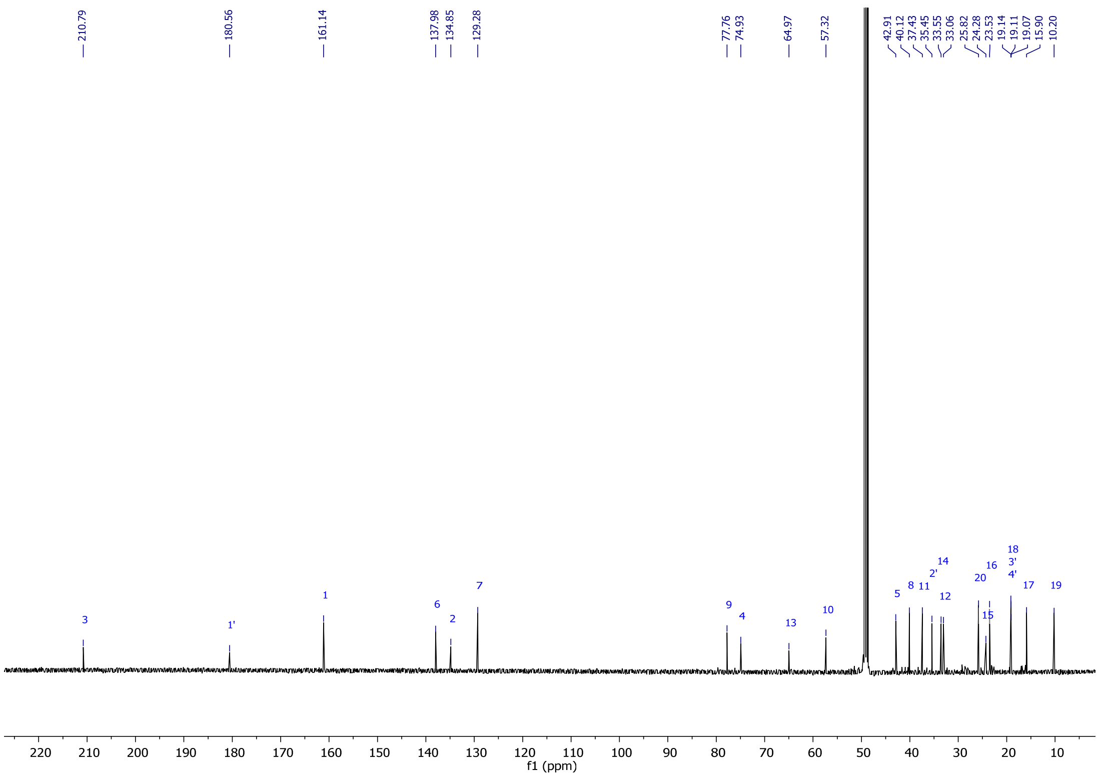

Figure S15b. ^{13}C-NMR spectrum of diDPB (**15**) in CD_3OD (125 MHz)

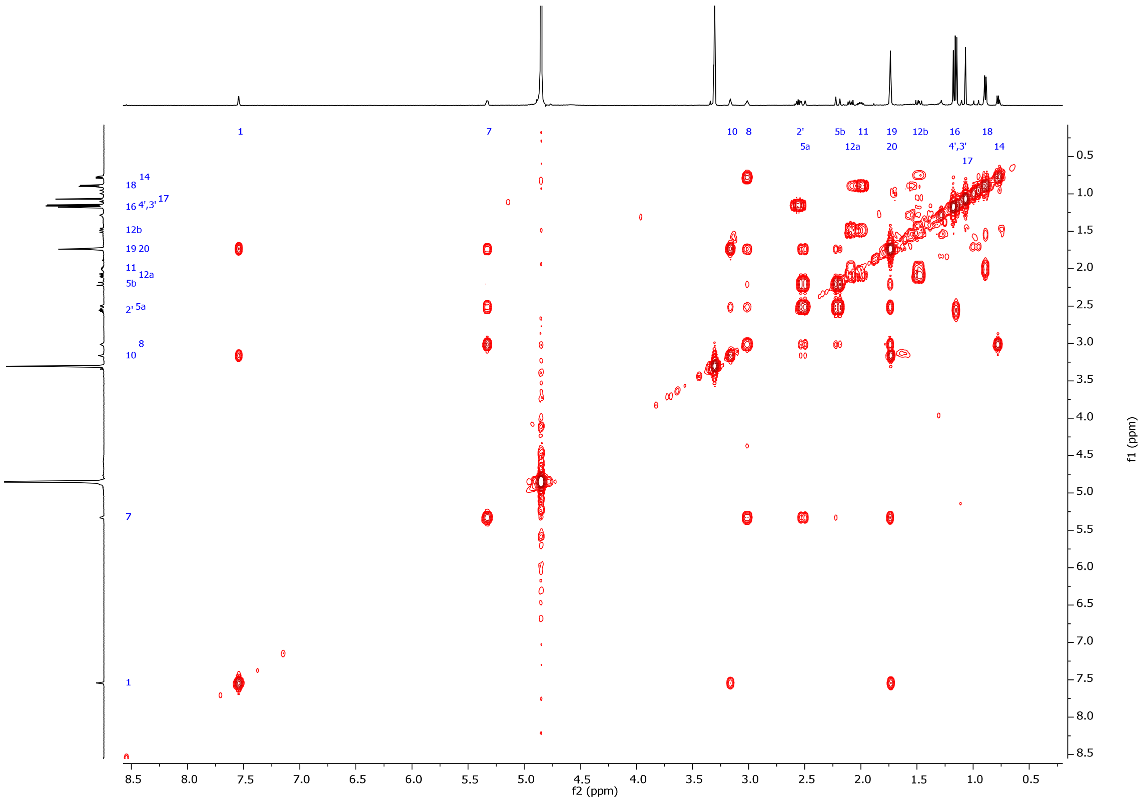

Figure S15c. COSY spectrum of diDPB (**15**) in CD_3OD.

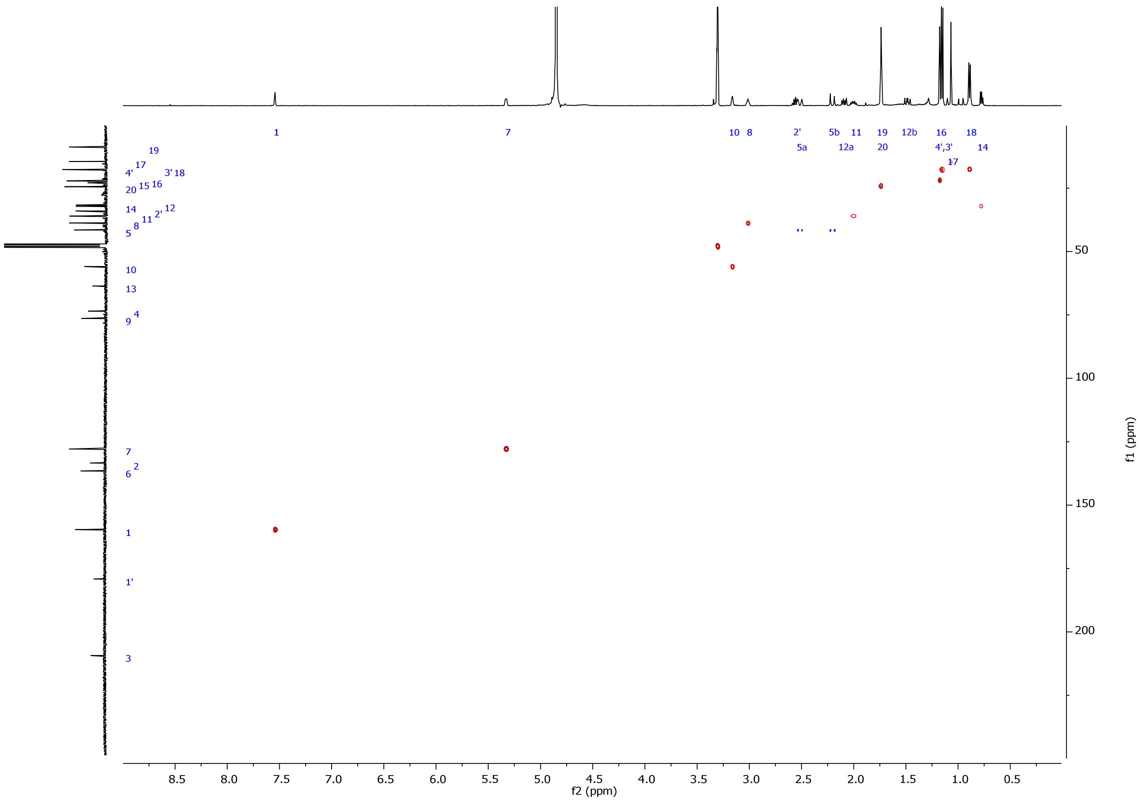

Figure S15d. HSQC spectrum of diDPB (**15**) in CD_3OD.

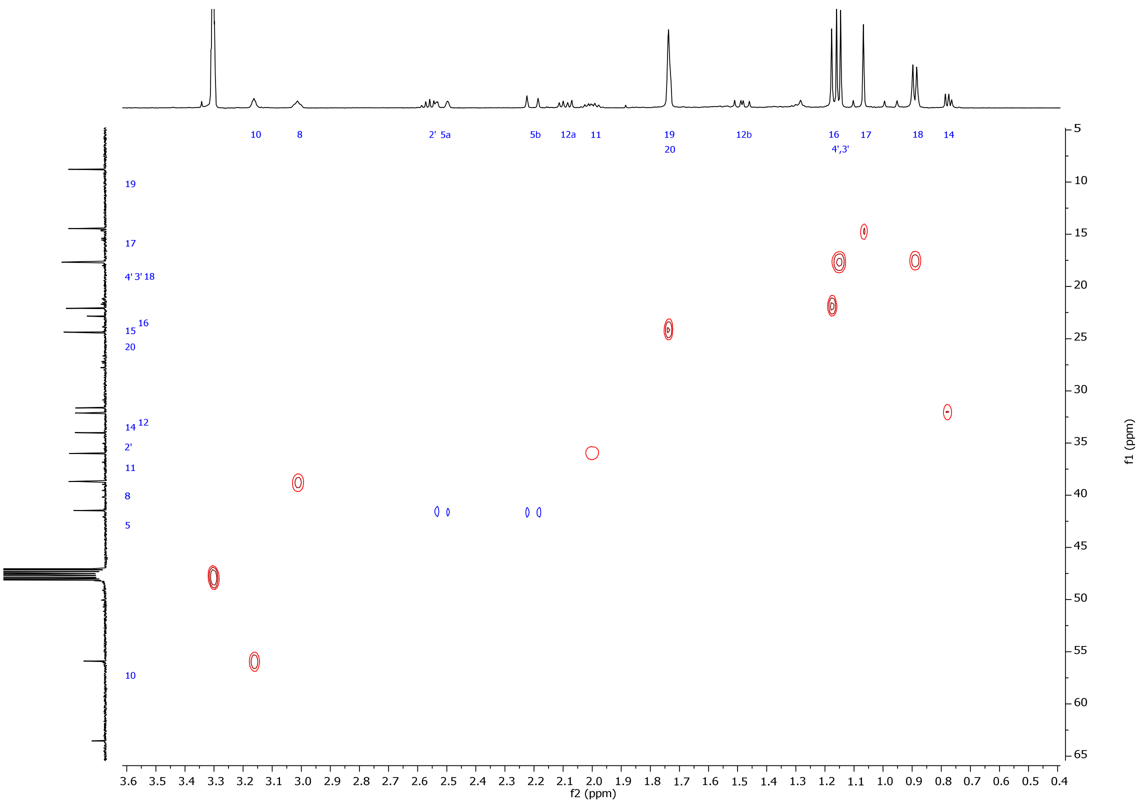

Figure S15e. Expansion (δ_H 3.6-0.4, δ_C 65-5) of HSQC spectrum of diDPB (**15**) in CD_3OD.

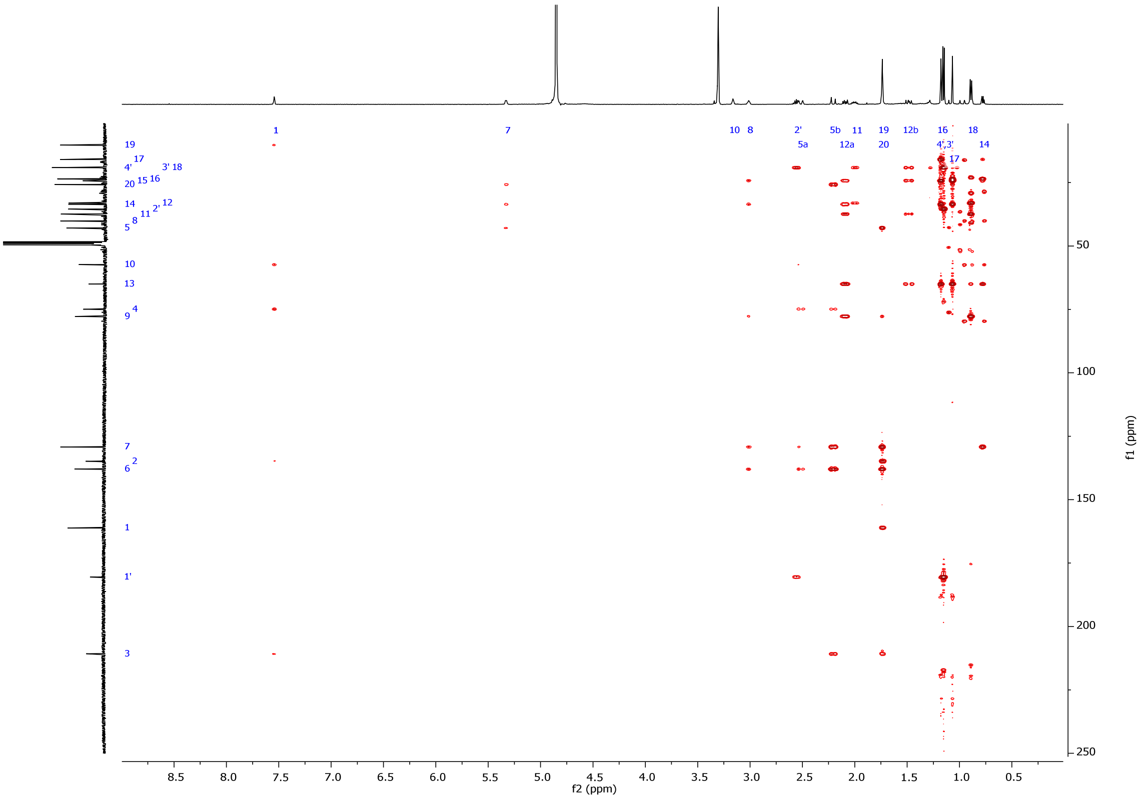

Figure S15f. HMBC spectrum of diDPB (**15**) in CD_3OD.

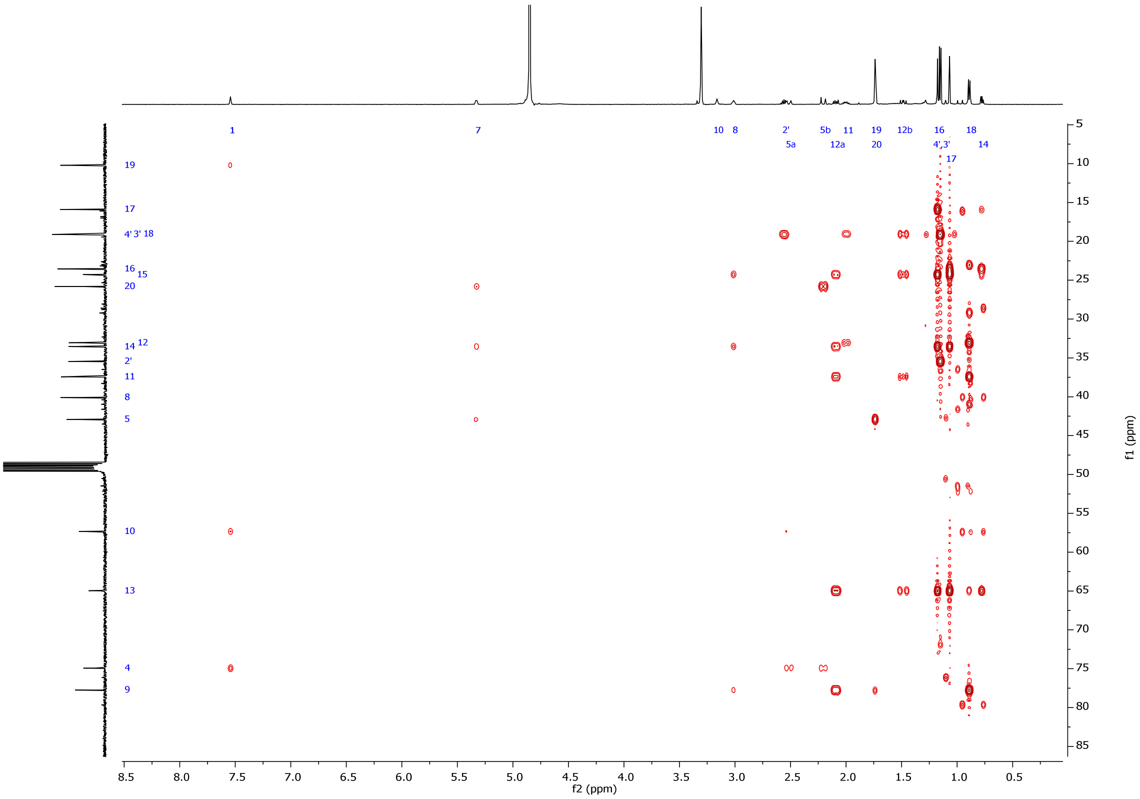

Figure S15g. Expansion (δ_H 8.5-0.3, δ_C 85-5) of HMBC spectrum of diDPB (**15**) in CD_3OD.

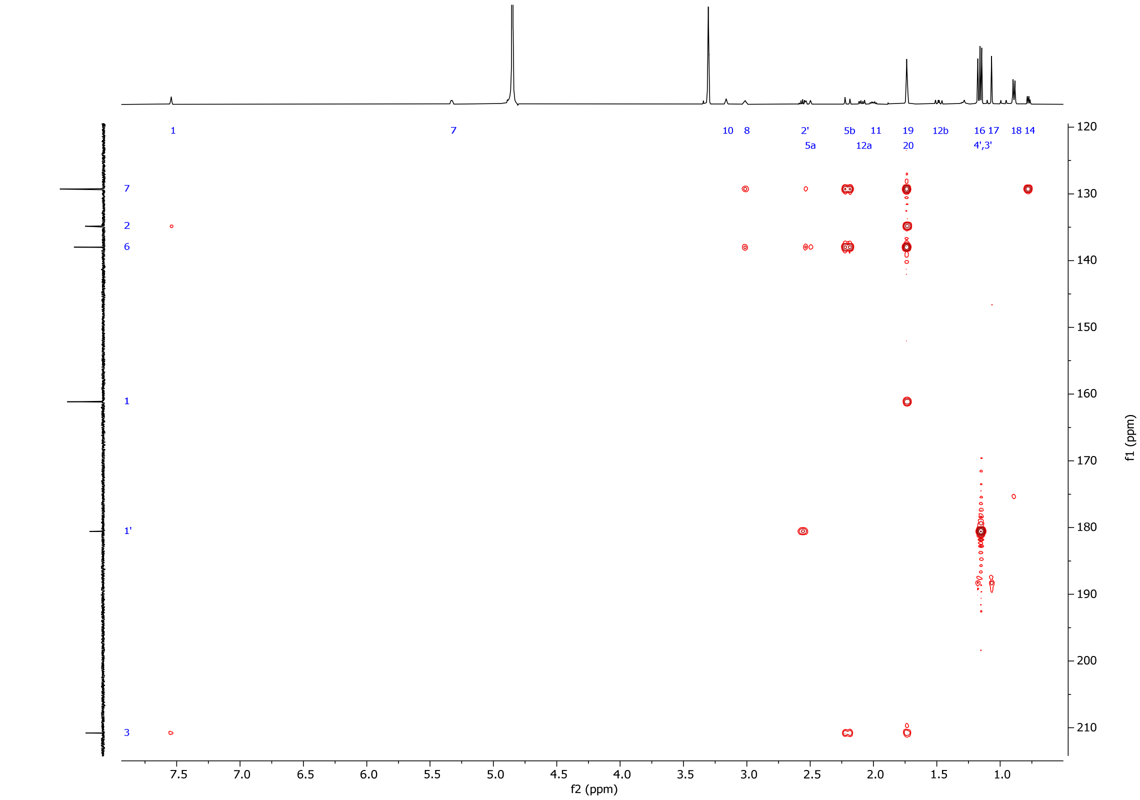

Figure S15h. Expansion (δ_H 8.0-0.3, δ_C 210-120) of HMBC spectrum of diDPB (**15**) in CD_3OD.

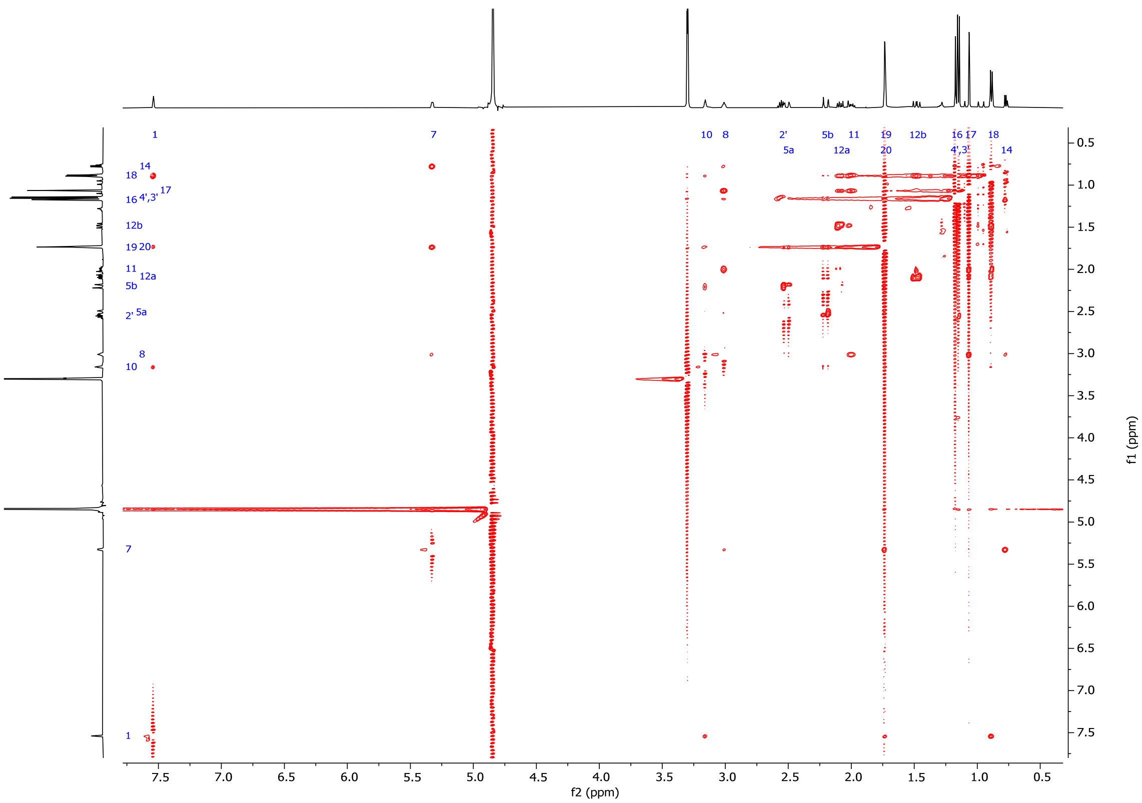

Figure S15i. NOESY2D spectrum of diDPB (**15**) in CD_3OD.

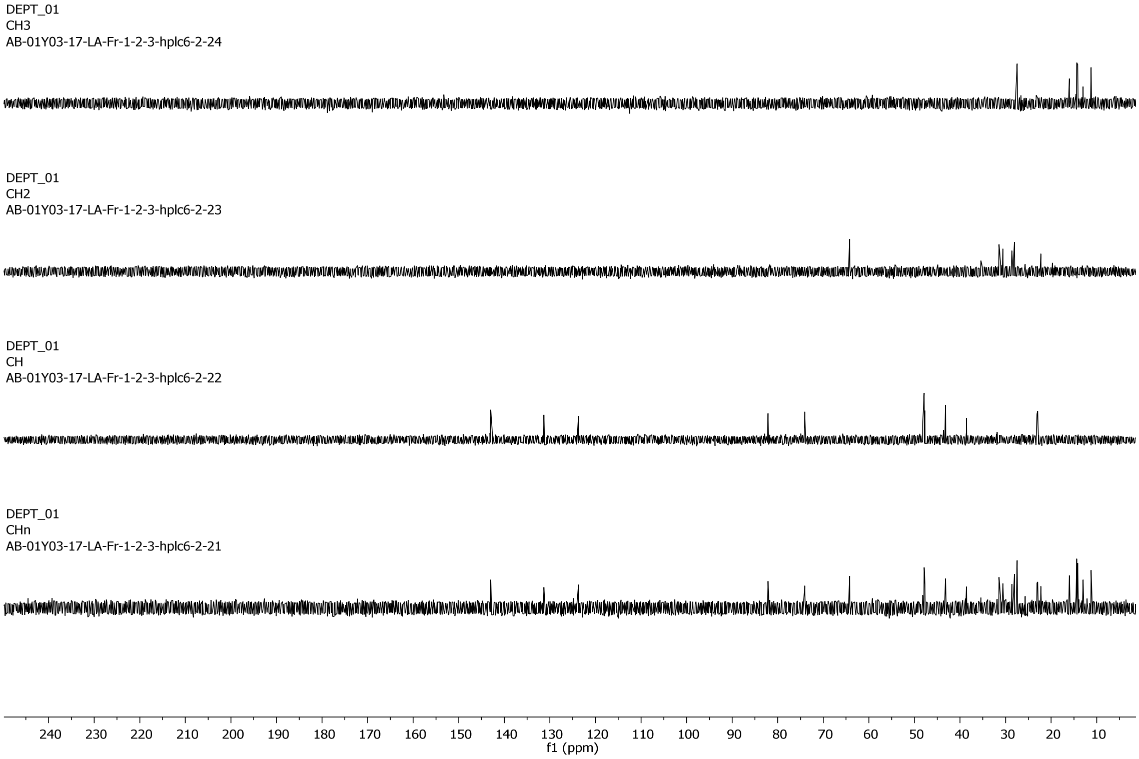

Figure S15j. DEPT spectrum of diDPB (**15**) in CD_3OD.

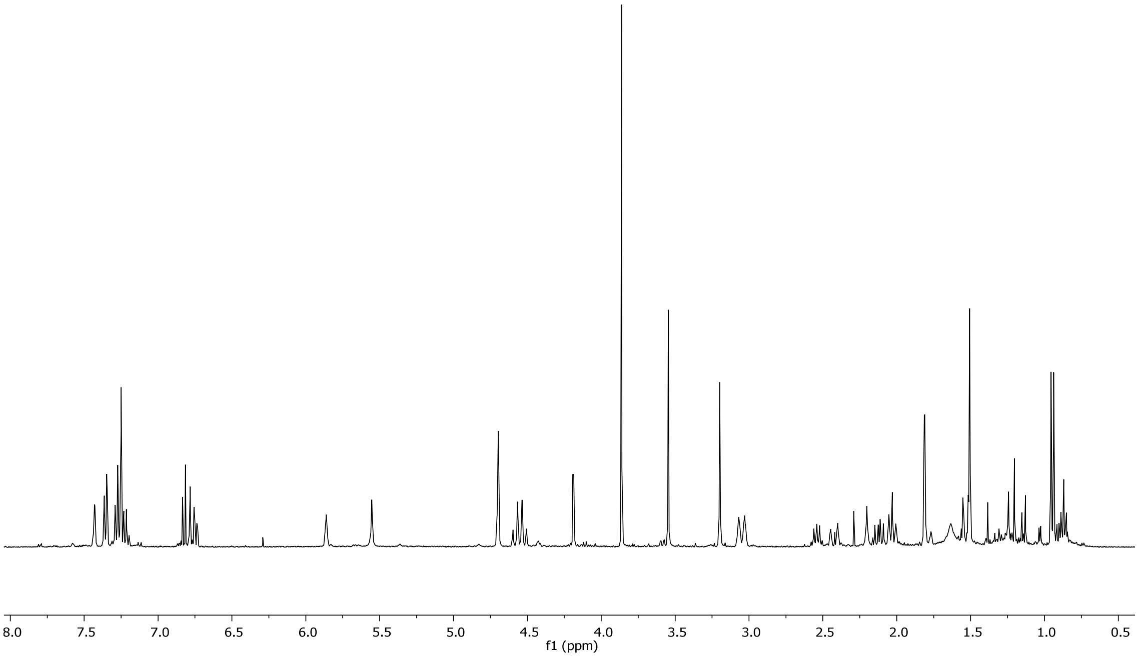

Figure S16a. ^{1}H NMR spectrum of RTX (**16**) in $CDCl_3$ (400 MHz).

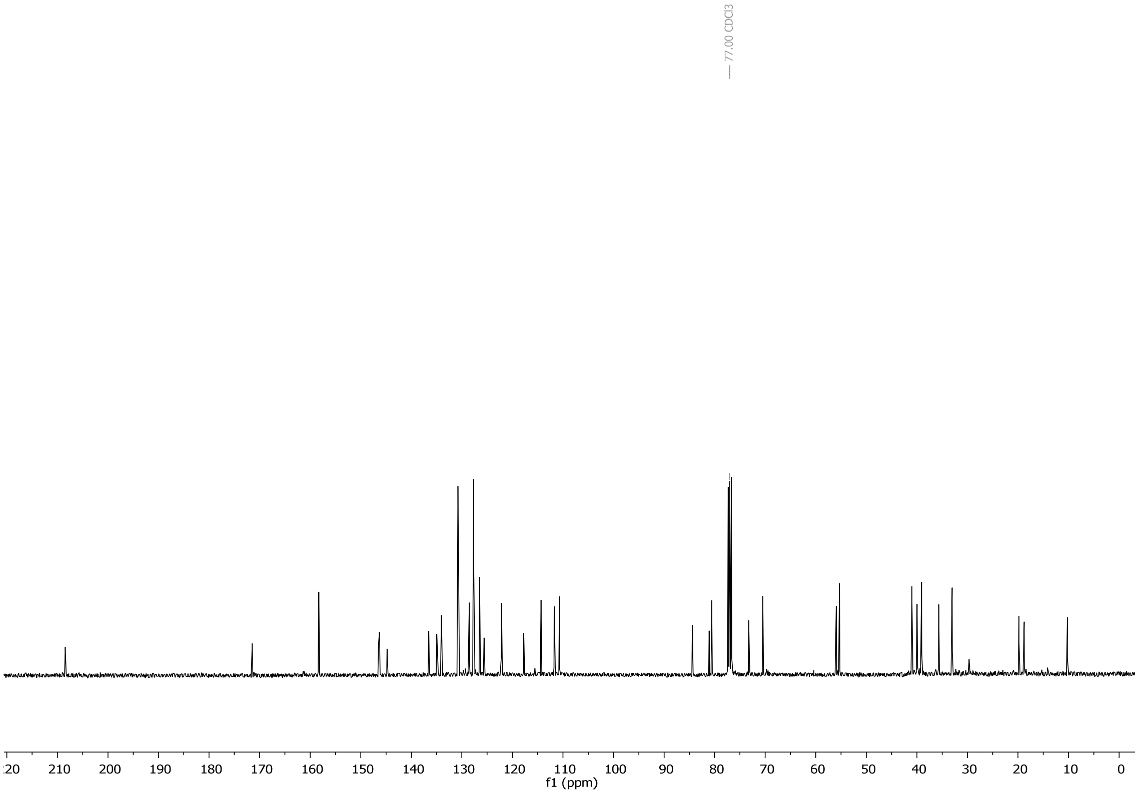

Figure S16b. ^{13}C NMR spectrum of RTX (**16**) in $CDCl_3$ (100 MHz).

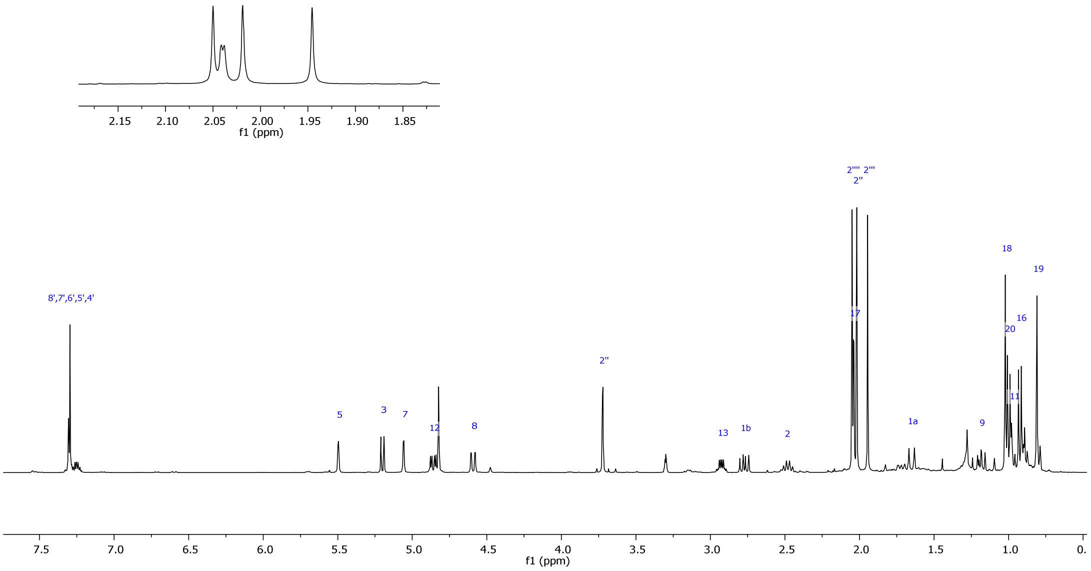

Figure S17a. ^{1}H-NMR spectrum of EOF1 (**17**) in CD_3OD (400 MHz).

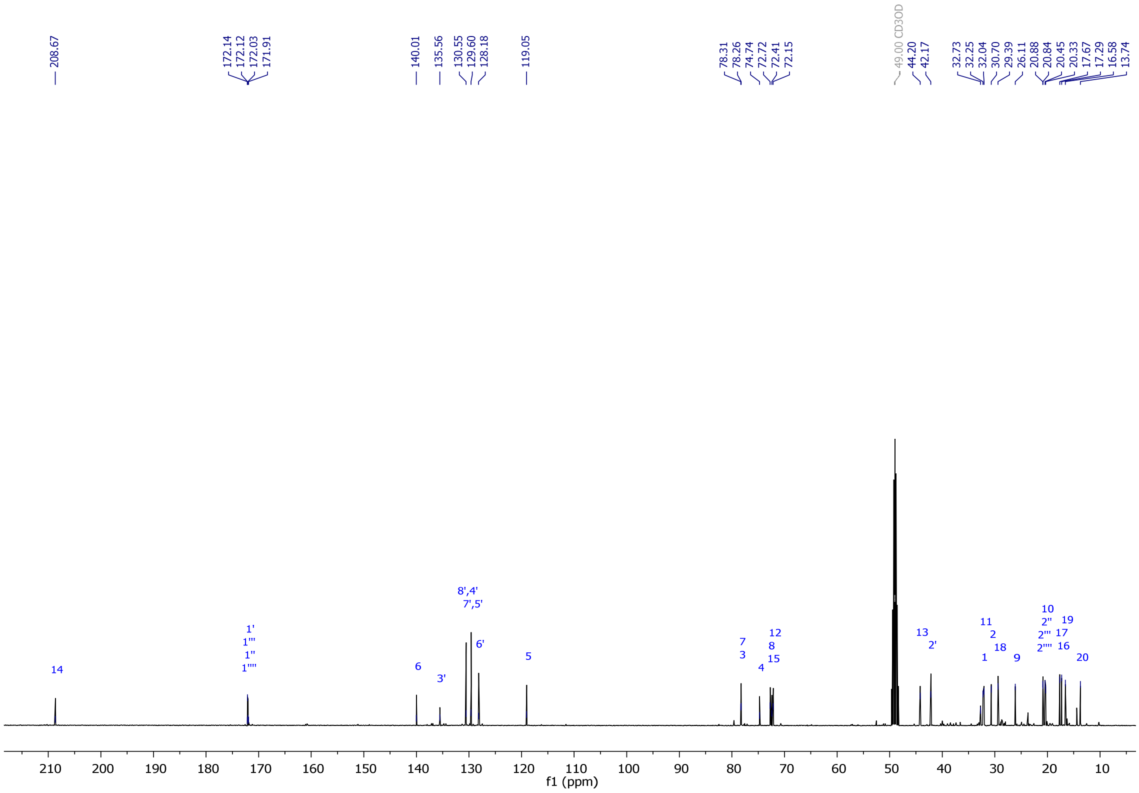

Figure S17b. ^{13}C-NMR spectrum of EOF1 (**17**) in CD_3OD. (100 MHz).

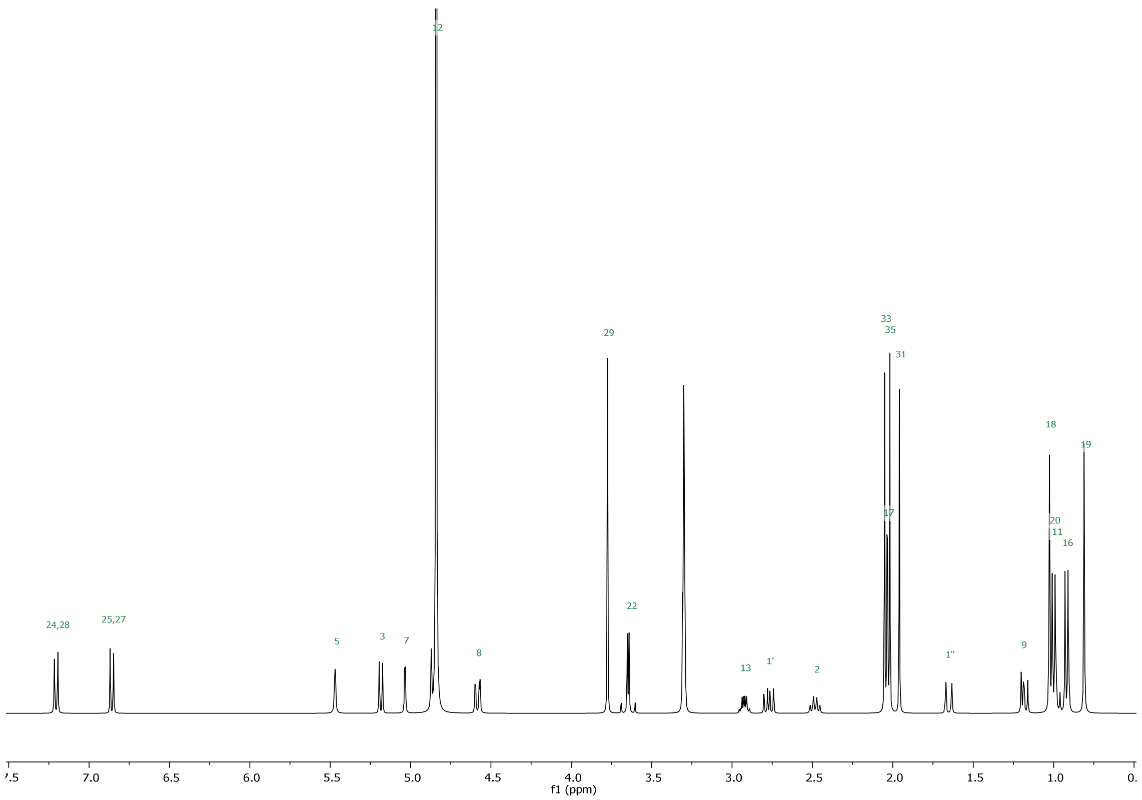

Figure S18a. ^{1}H-NMR spectrum of EOF2 (**18**) in CD_3OD (400 MHz).

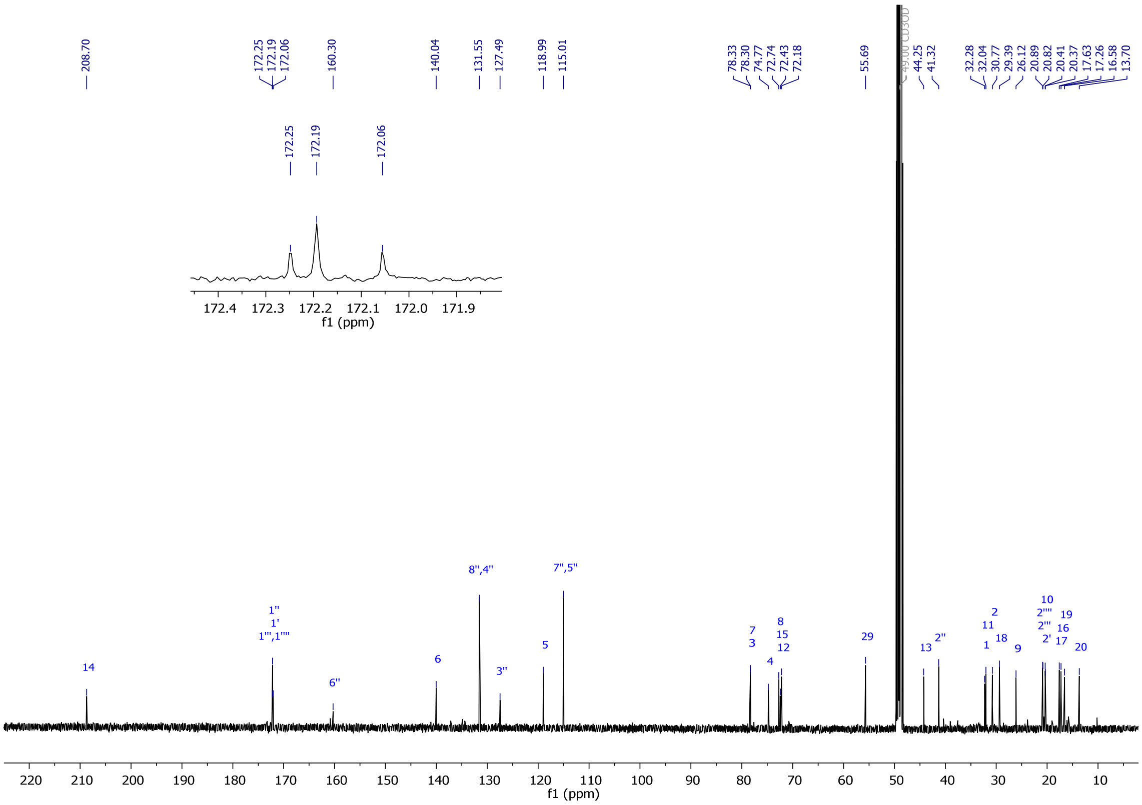

Figure S18b. ^{13}C-NMR spectrum of EOF2 (**18**) in CD_3OD (100 MHz).

2- ECD data

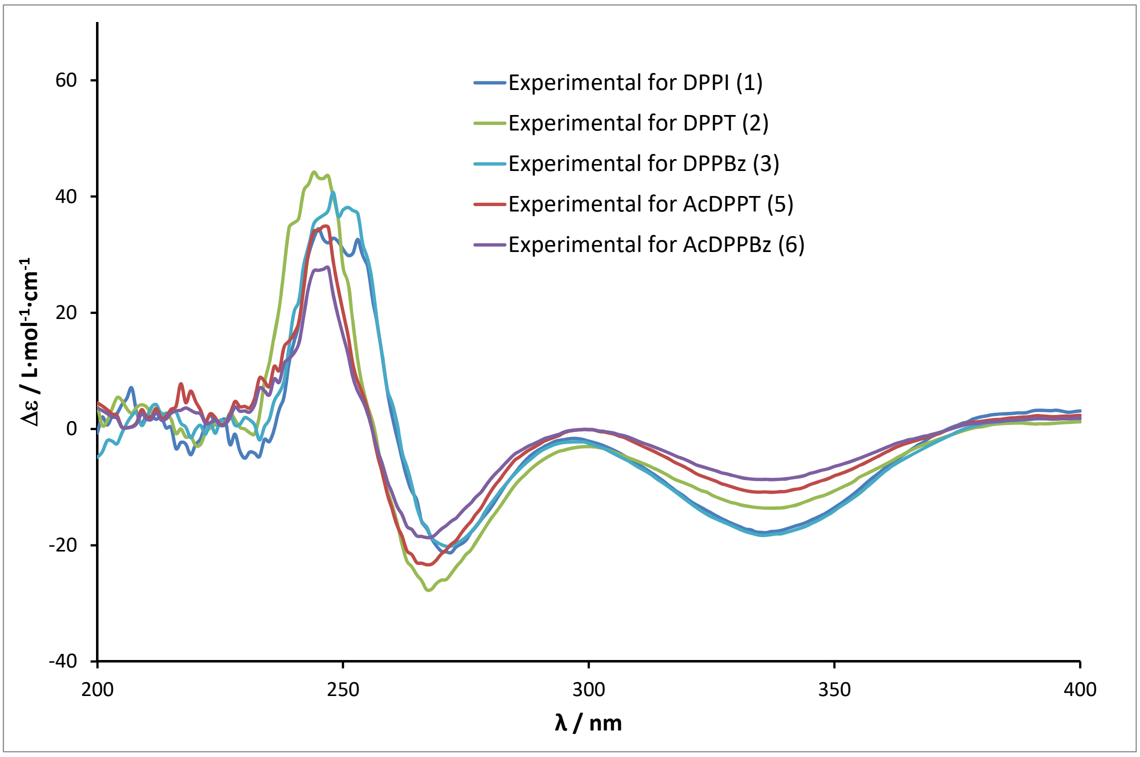

Figure S19. Experimental ECD spectra of DPPI (**1**), DPPT (**2**), DPPBz (**3**), AcDPPT (**5**) and AcDPPBz (**6**).

3- UHPLC-HRMS^E data. Figures.

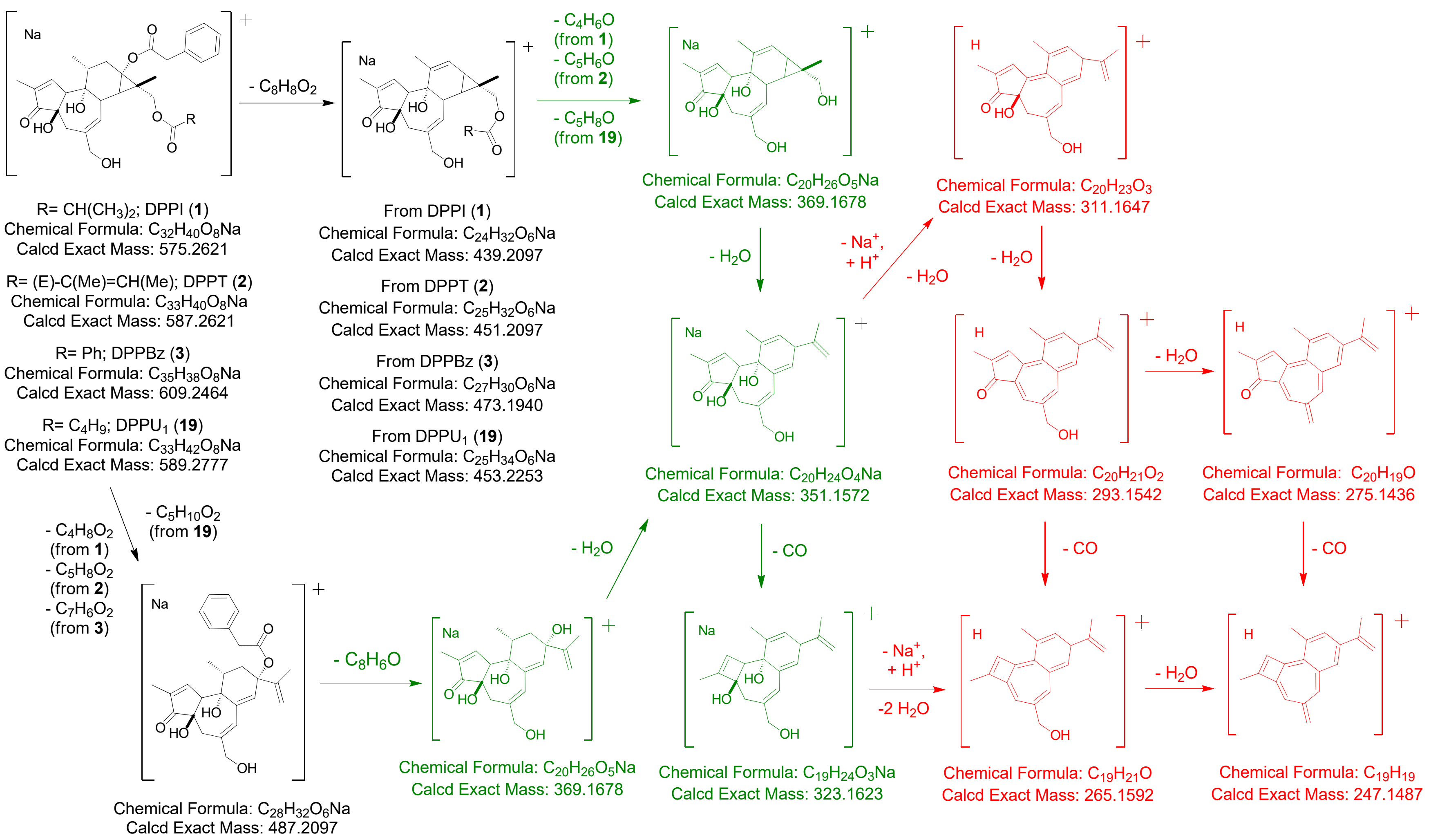

Figure S20. Proposed fragmentation route for selected ions on HRMS^E spectrum (Data Independent Acquisition (DIA)) [1], in ESI positive mode, for DPPI (**1**), DPPT (**2**), DPPBz (**3**) and DPPU$_1$ (**19**). In red, common daughter ions with group B compounds (see section 2.2 and Figure S22).

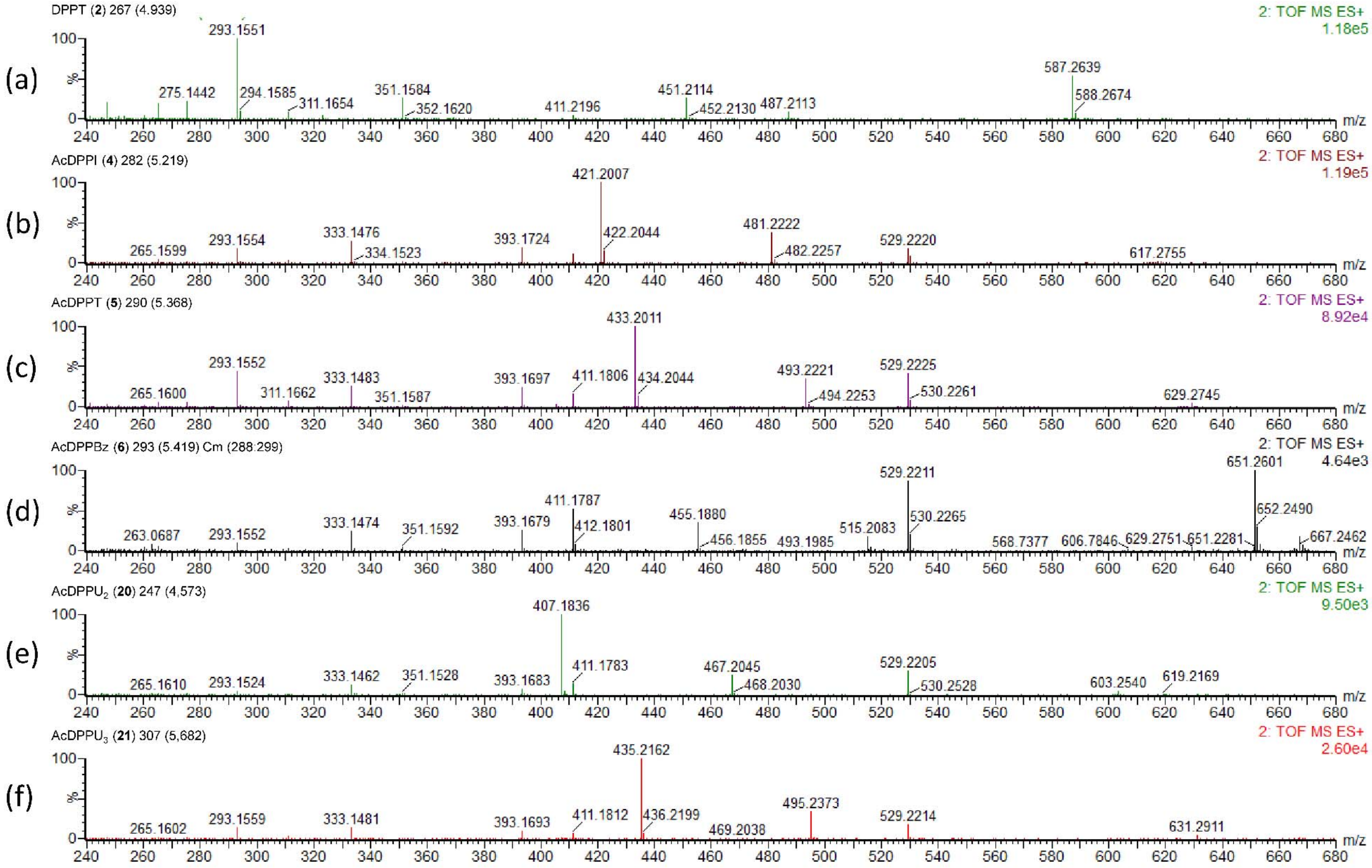

Figure S21. Comparison of HRMS^E spectra (DIA) [1] for (**a**) DPPT (**2**), (**b**) AcDPPI (**4**), (**c**) AcDPPT (**5**), (**d**) AcDPPBz (**6**), (**e**) AcDPPU$_2$ (**20**) and (**f**) AcDPPU$_3$ (**21**), m/z range 240-680 (data acquired in ESI positive mode with a ramp trap collision energy of the high-energy function set at 60-120 eV).

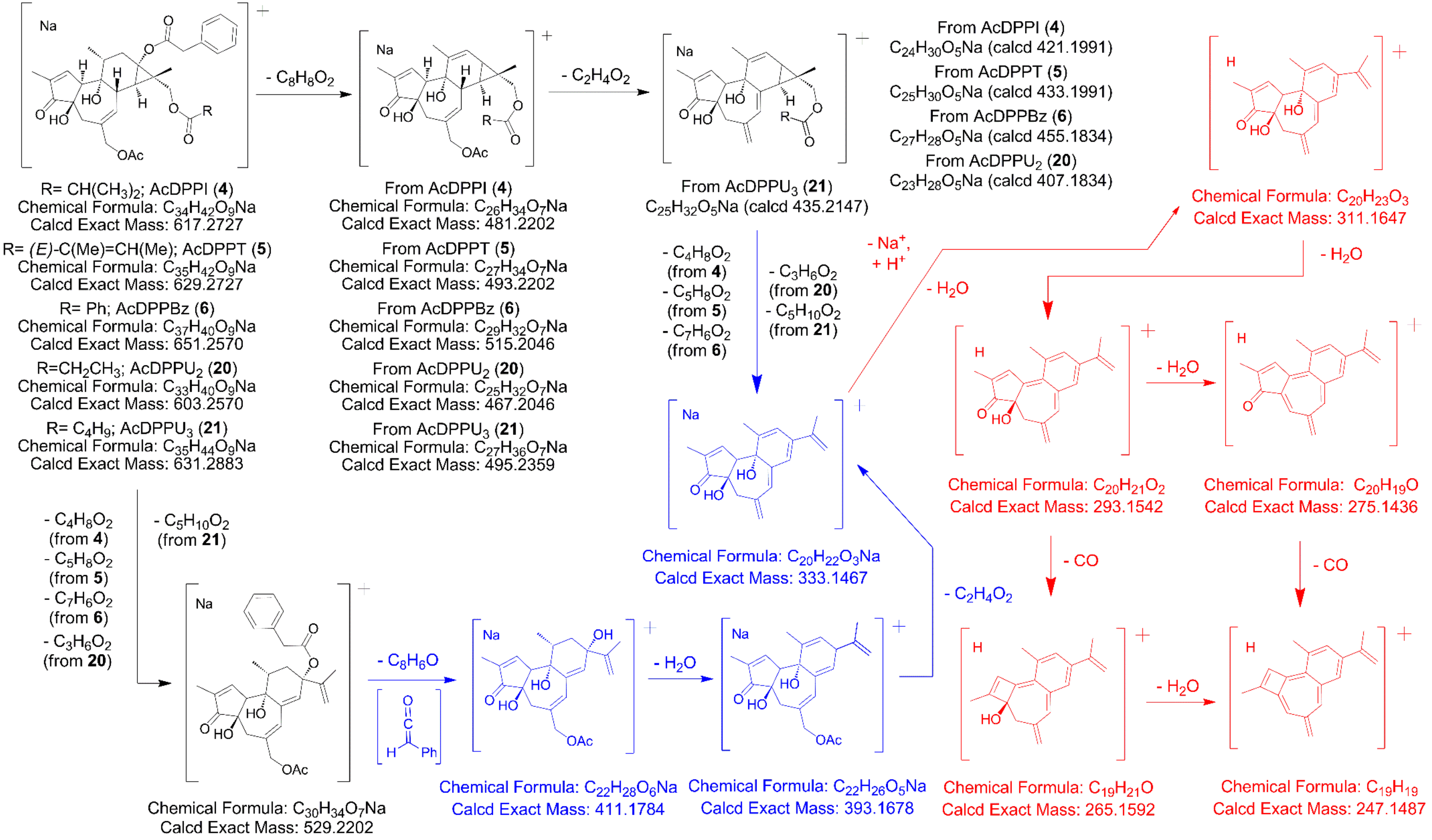

Figure S22. Proposed fragmentation route for selected ions on HRMS^{E} spectrum (DIA) [1], in ESI positive mode, for AcDPPI (**4**), AcDPPT (**5**), AcDPPBz (**6**), AcDPPU$_2$ (**20**) and AcDPPU$_3$ (**21**). In red, common daughter ions with group A compounds (see section 2.2 and Figure S20).

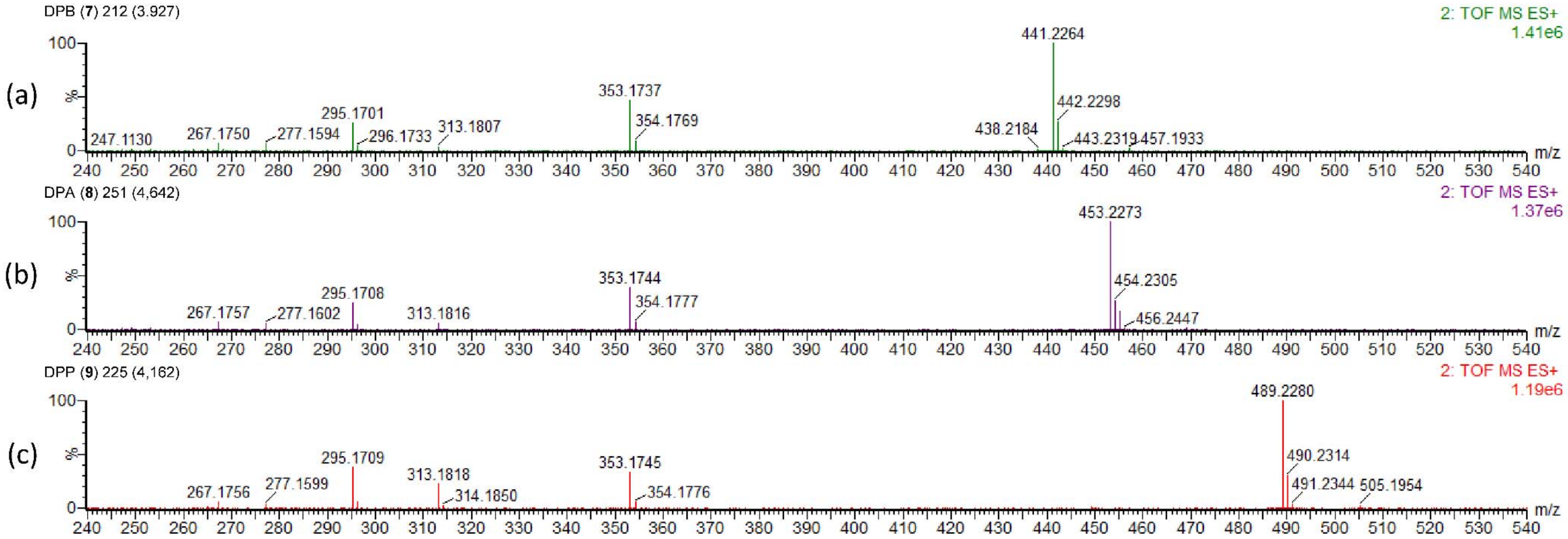

Figure S23. Comparison of HRMS^E spectra (DIA) [1] for (**a**) DPB (**7**), (**b**) DPA (**8**) and (**c**) DPP (**9**), m/z range 240-540 (data acquired in ESI positive mode with a ramp trap collision energy of the high-energy function set at 10-40 eV).

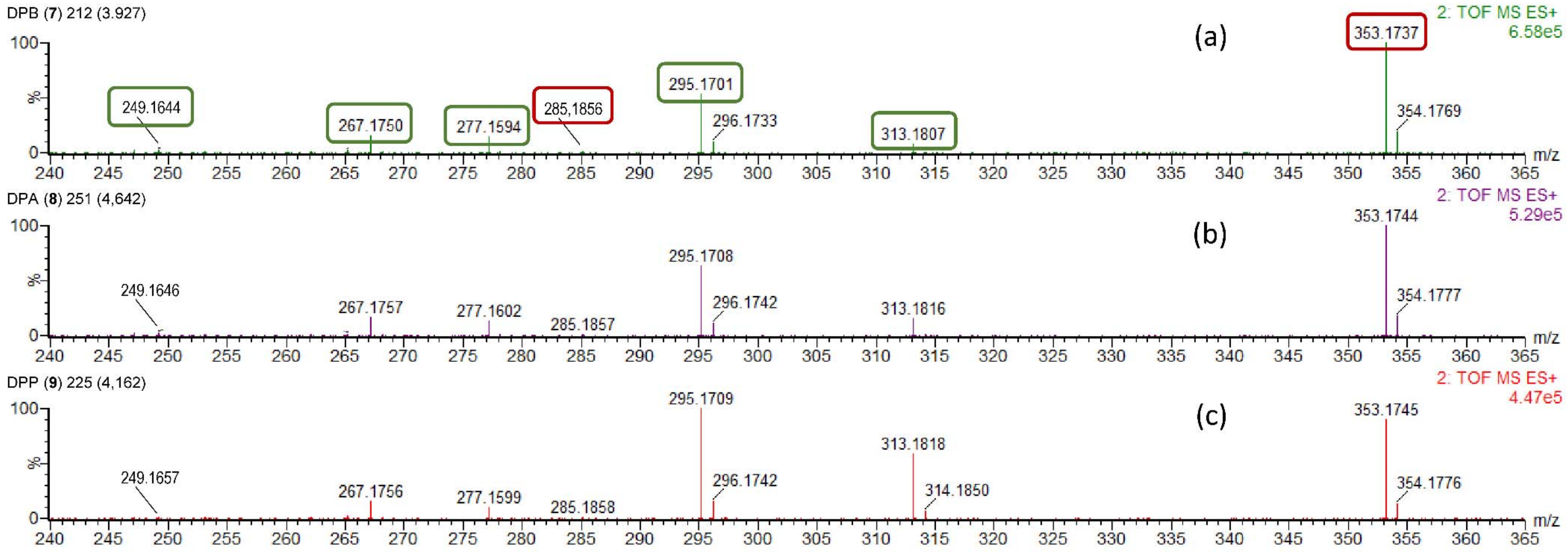

Figure S24. Comparison of HRMS^E spectra (DIA) [1] for: (a) DPB (7), (b) DPA (8) and, (c) DPP (9) X23); m/z range 240-365 (m/z range 240-540 in Figure S23) (data acquired in positive ionization with a ramp trap collision energy of the high-energy function set at 10-40 eV). Compounds **7**, **8** and **9** show daughter ions from their corresponding $[M+Na]^+$ molecular ions (see Figure S23) in their HRMS^E spectra at m/z 313.1804, 295.1698, 277.1592, 267.1749 and 249.1643 (calculated) (in green in Scheme 3, Figures S24a and S25) which could be assigned to losses of water (1, 2 and 3 molecules), 2 molecules of water and CO and 3 molecules of water and CO, respectively, from a precursor ion at m/z 353.1729 (calculated) (in red in Scheme 3, Figures S24a and S25) (Table S2). Last mentioned ion, which is relatively abundant in HRMS^E spectra of DPB (**7**) DPA (**8**) and DPP (**9**) (Table S2, Figures S23 and S24) originates from a loss of an ester group at C-13 from parent molecular ions in each compound (Figure S23). See proposed fragmentation route for selected ions mentioned above in Scheme 3 and Figure S25, together with colour key

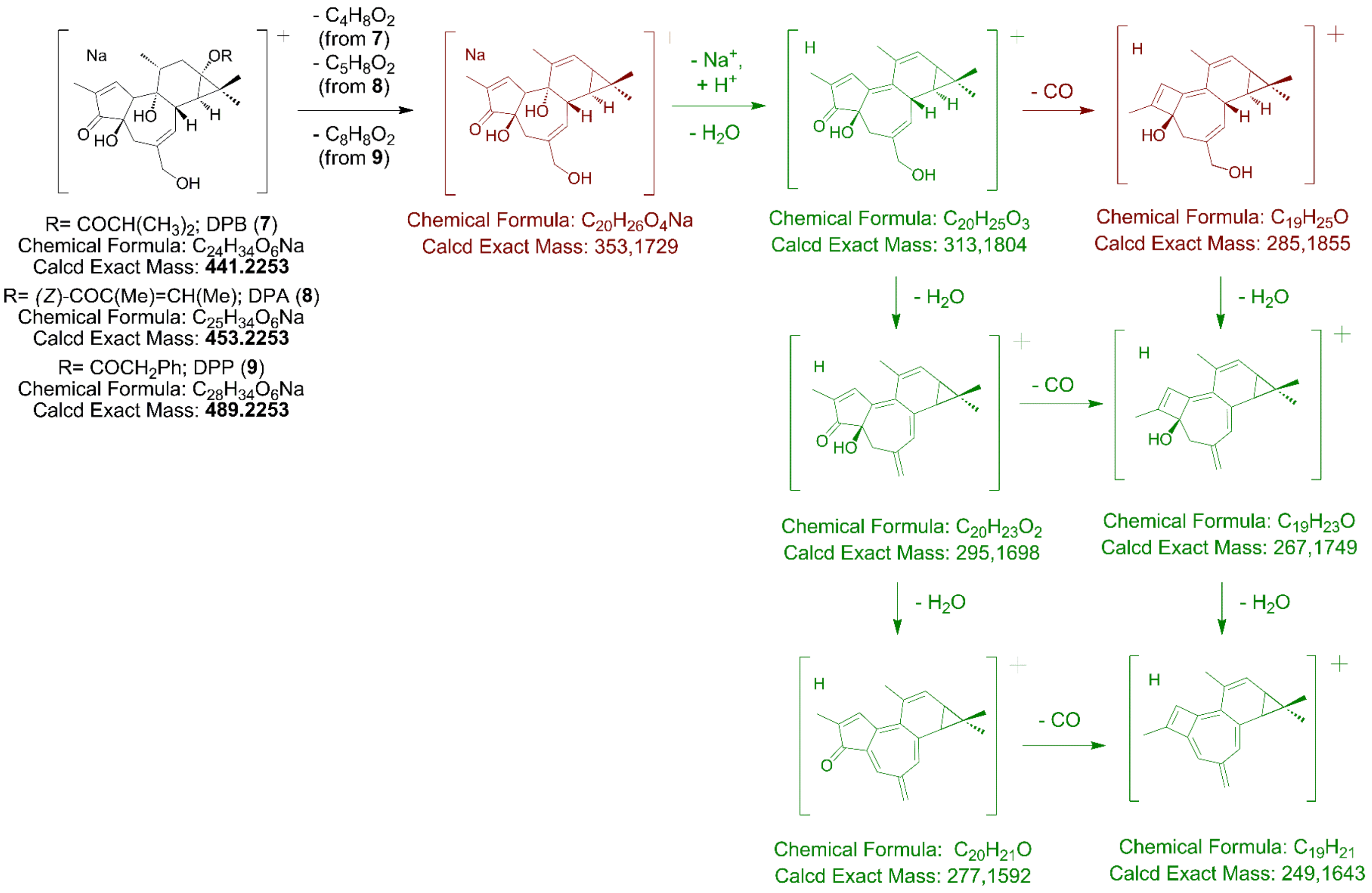

Figure S25. Proposed fragmentation route for selected ions on HRMS^E spectrum (DIA) [1], in ESI positive mode, for DPB (**7**), DPA (**8**) and DPP (**9**). In green, common daughter ions with group D compounds (see section 2.2 and Figure S29).

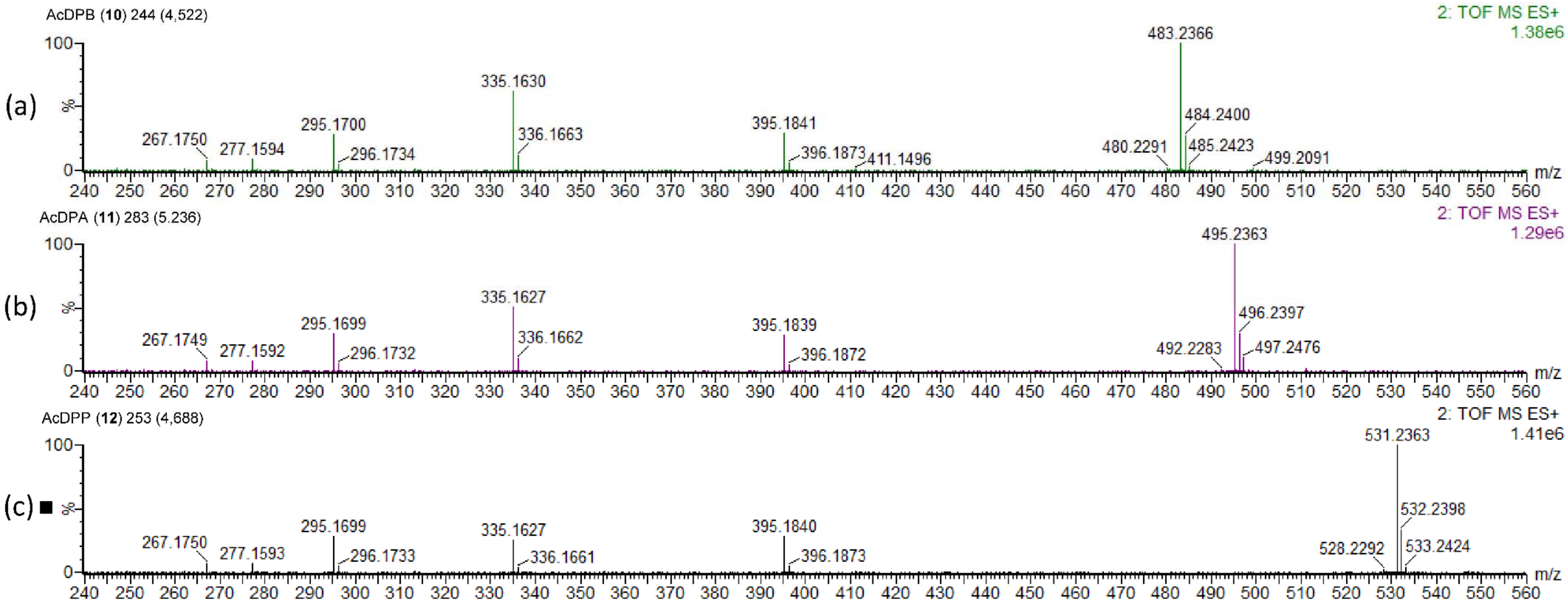

Figure S26. Comparison of HRMS^{E} spectra (DIA) [1] for (**a**) AcDPB (**10**), (**b**) AcDPA (**11**), and (**c**) AcDPP (**12**), m/z range 240-560 (data acquired in ESI positive mode with a ramp trap collision energy of the high-energy function set at 10-40 eV).

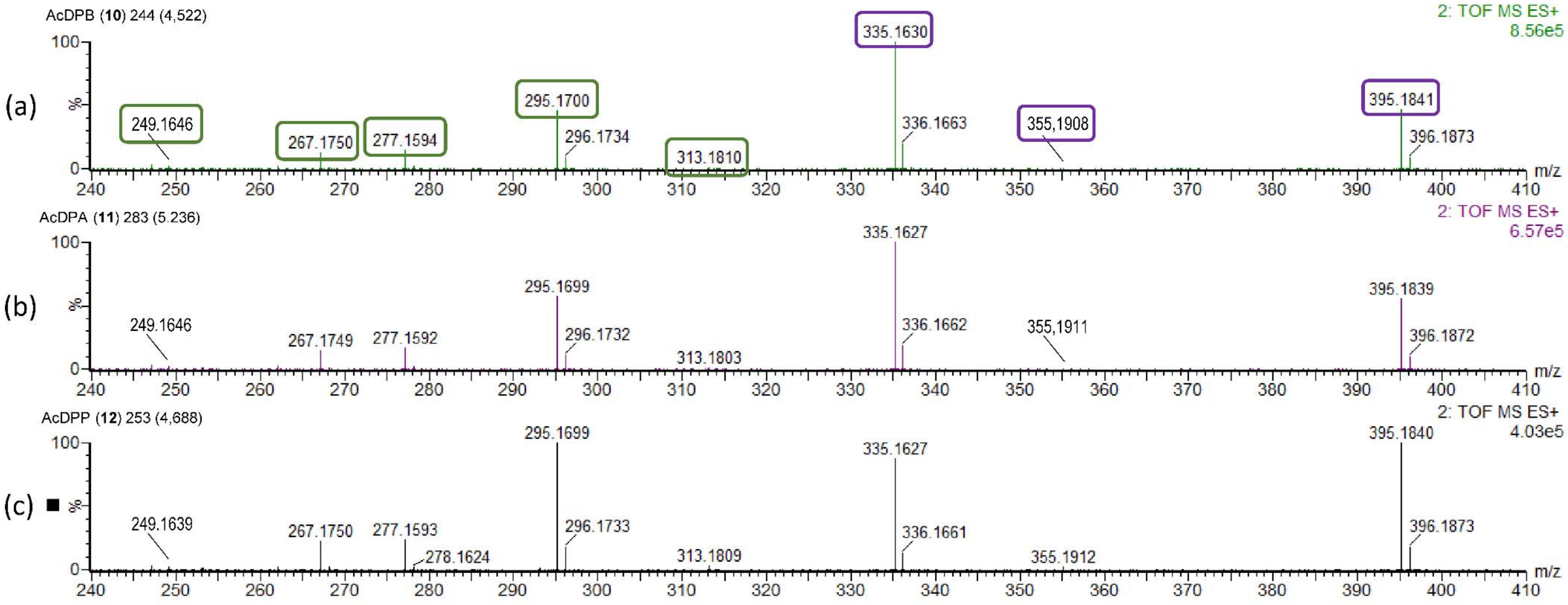

Figure S27. Comparison of HRMS^E spectra (DIA) [1] for (**a**) AcDPB (**10**), (**b**) AcDPA (**11**), and (**c**) AcDPP (**12**), m/z range 240-410 (data acquired in ESI positive mode with a ramp trap collision energy of the high-energy function set at 10-40 eV).

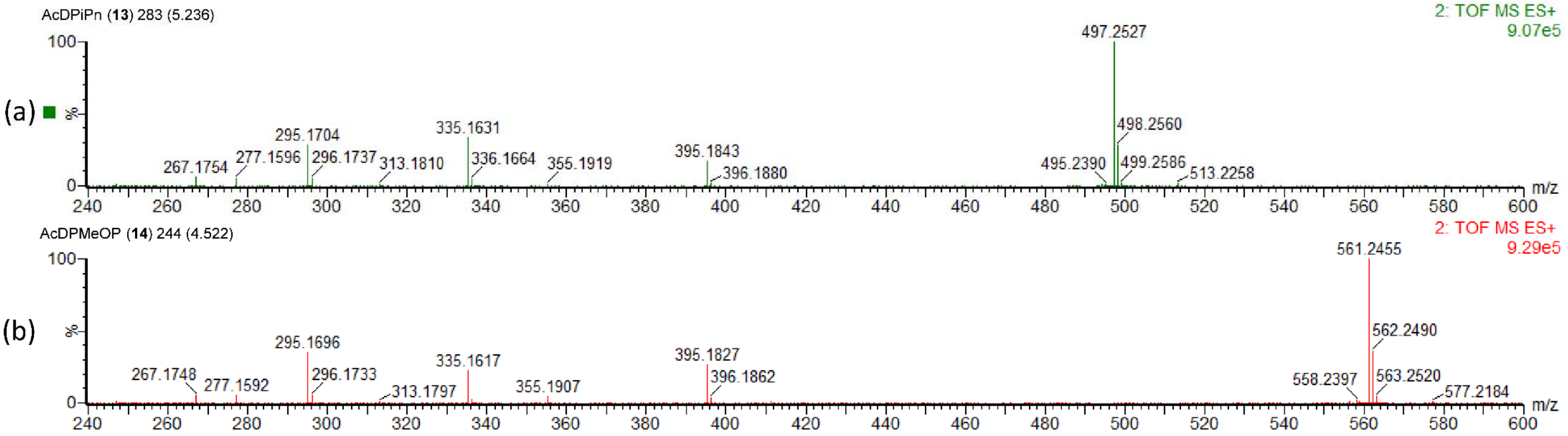

Figure S28. Comparison of HRMS^E spectra (DIA) [1] for (**a**) AcDPiPn (**13**) and (**b**) AcDPMeOP (**14**), m/z range 240-600 (m/z range 240-410 in **Figure 7**) (data acquired in ESI positive mode with a ramp trap collision energy of the high-energy function set at 10-40 eV).

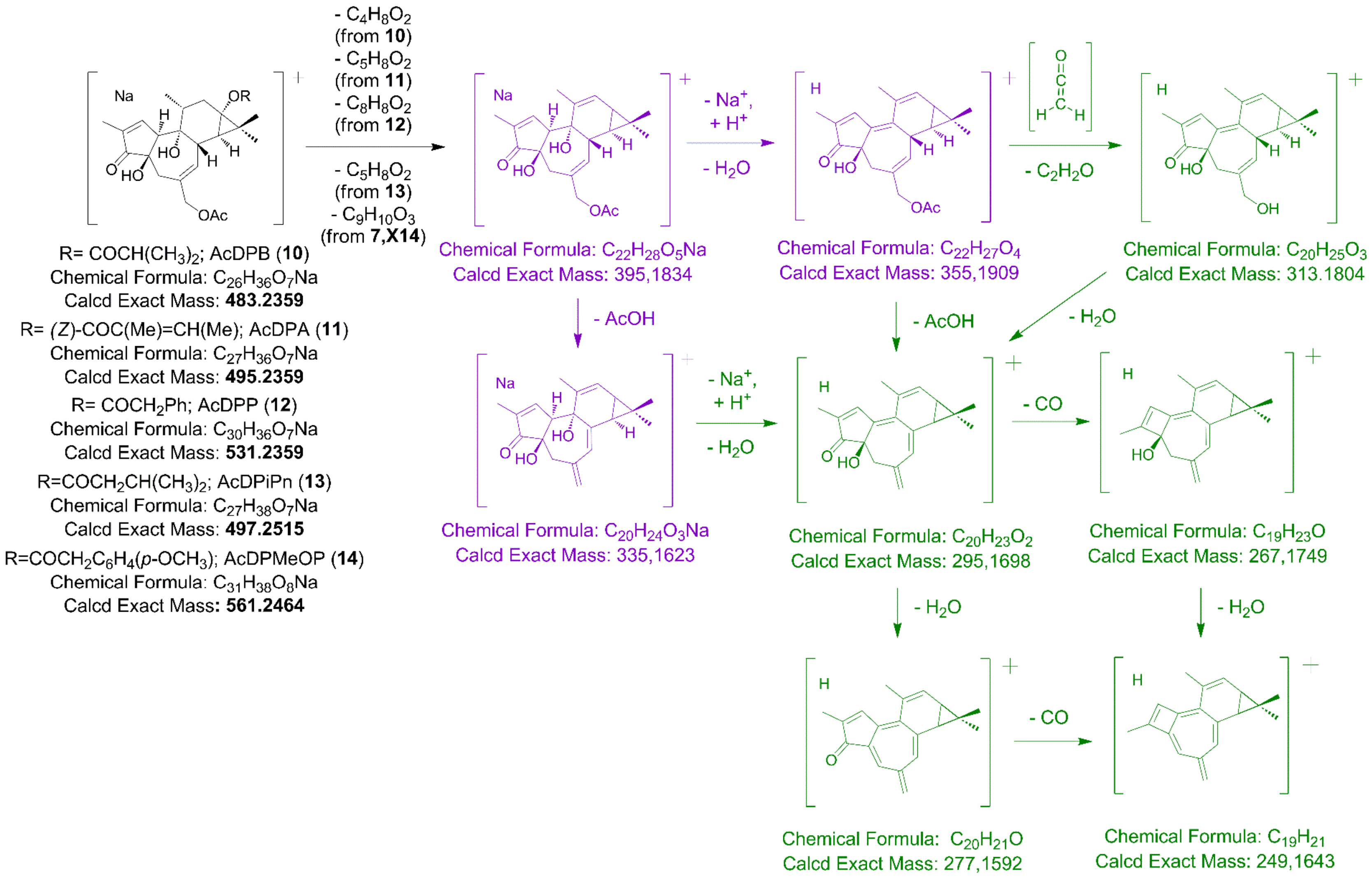

Figure S29. Proposed fragmentation route for selected ions on HRMS^E spectrum (DIA) [1], in ESI positive mode, for AcDPB (**10**), AcDPA (**11**), AcDPP (**12**), AcDPiPn (**13**) and AcDPMeOP (**14**). In green, common daughter ions with group C compounds (see section 2.2 and Figure S25).

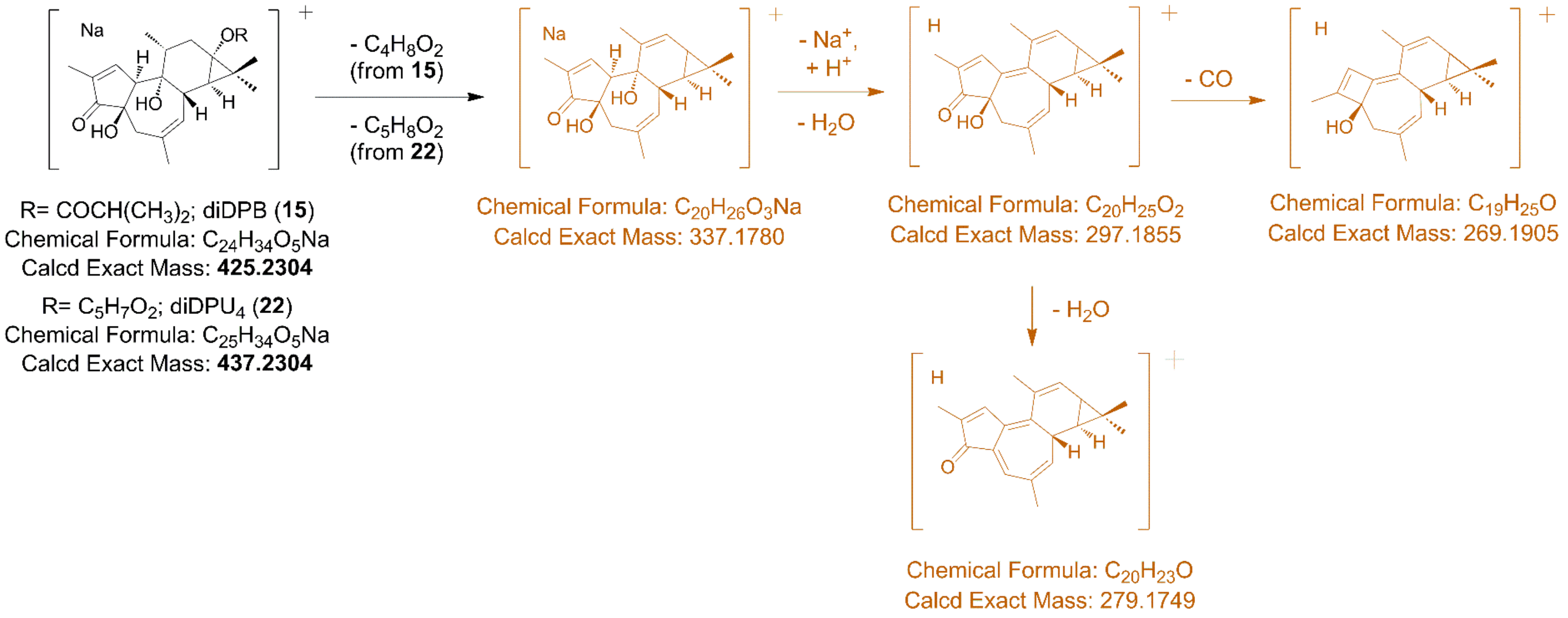

Figure S30. Proposed fragmentation route for selected ions on HRMS^{E} spectrum (DIA) [1], in ESI positive mode, for diDPB (**15**) and diDPU$_4$ (**22**).

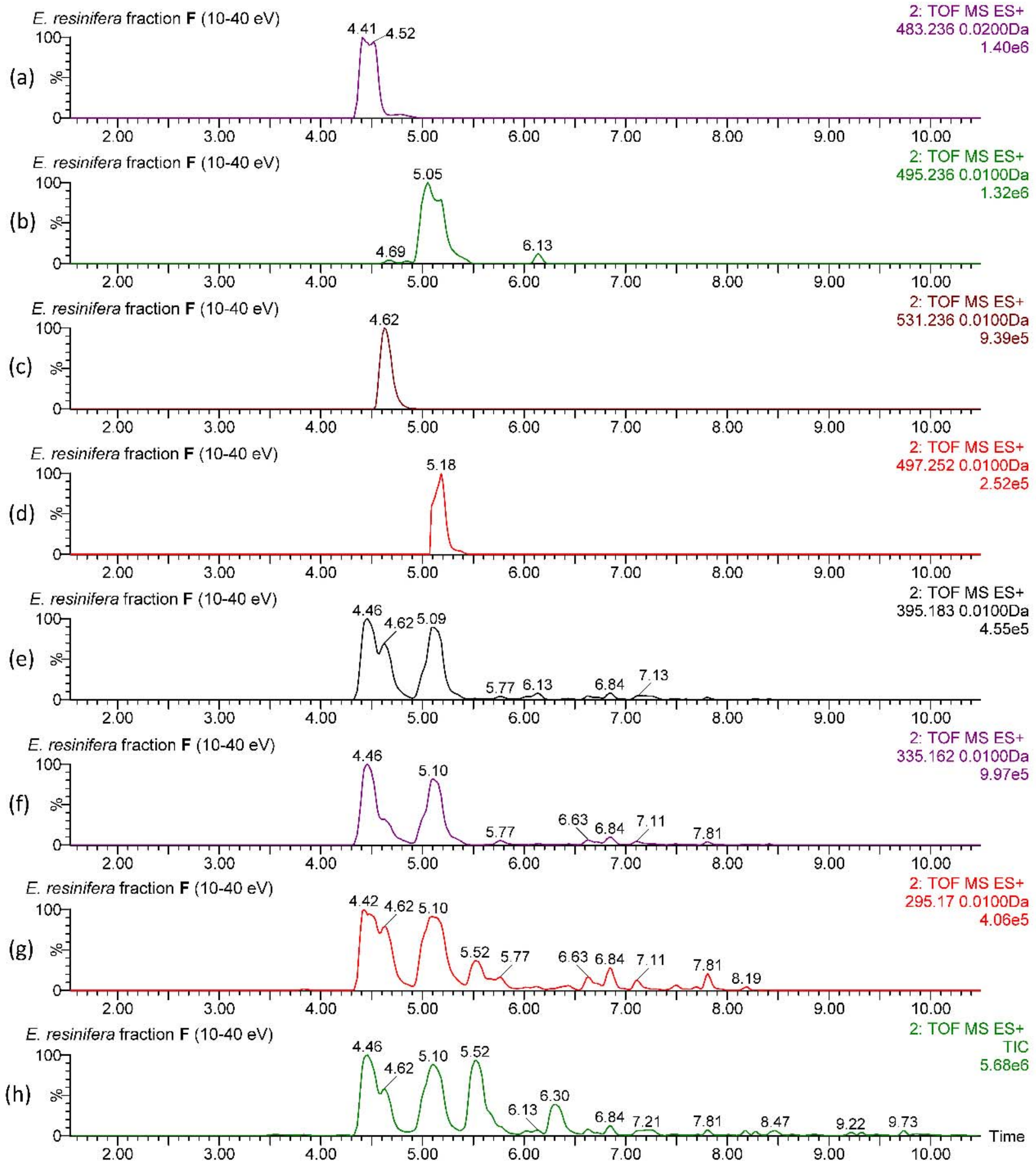

Figure S31. Total Ion Current (TIC) and eXtracted Ion Chromatograms (XICs), at selected *m/z* previously observed in high energy HRMS^E of 12-deoxyphorbol 20-acetate-13-acyl derivatives, obtained from UHPLC-HRMS^E experiments for chromatographic fraction F of *E. resinifera* (data acquired in ESI positive mode with a ramp trap collision energy of the high-energy function set at 10-40 eV; high-energy function (2: TOF MS ES+)). XICs for 12-deoxyphorbol 20-acetate-13-acyl derivatives [M+Na]$^+$ molecular ions: *m/z* 483.2359 calculated mass for (**a**) $C_{26}H_{36}O_7Na$ (AcDPB (**10**)); (**b**) *m/z* 495.2359 calculated mass for $C_{27}H_{36}O_7Na$ (AcDPA (**11**)); (**c**) *m/z* 531.236, calculated mass for $C_{30}H_{36}O_7Na$ (AcDPP (**12**)) and (**d**) *m/z* 497.2515, calculated mass for $C_{27}H_{38}O_7Na$ (AcDPiPn (**13**)). XICs for selected featured ions of 12-deoxyphorbol 20-acetate-13-acyl derivatives HRMS^E spectra (see Scheme 4 and Figure S29) at: (**e**) *m/z* 395.1834; (**f**) *m/z* 335.1623; (**g**) *m/z* 295.1698. (**h**) UHPLC-HRMS^E chromatographic profile (TIC) of fraction F from *E. resinifera* extract.

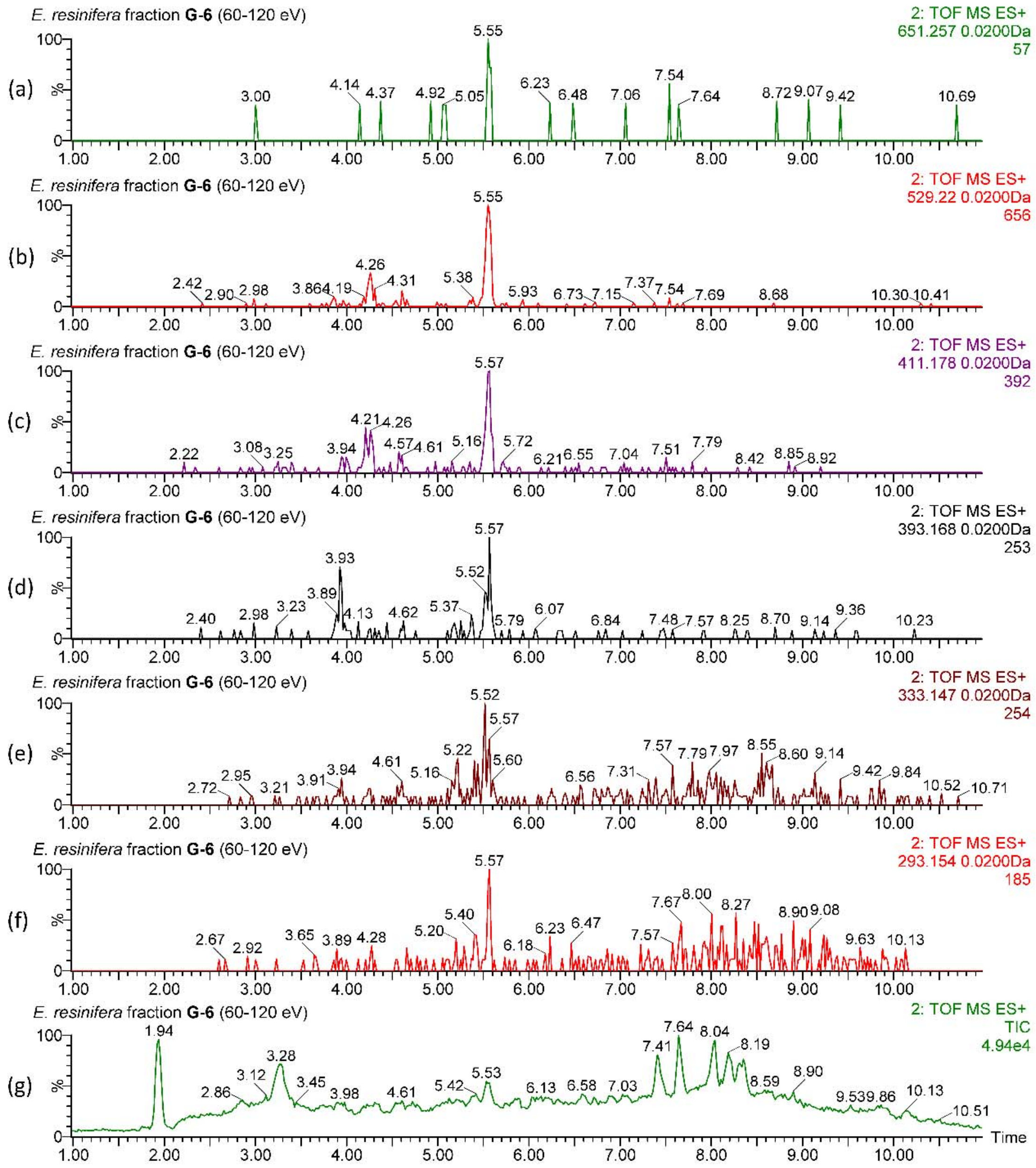

Figure S32. Total Ion Current (TIC) and eXtracted Ion Chromatograms (XICs), at selected m/z previously observed in high energy HRMS^E of 12-deoxy-16-hydroxyphorbol 20-acetate-13,16-diacyl derivatives, obtained from UHPLC-HRMS^E experiments for chromatographic fraction G-6 of *E. resinifera* (data acquired in ESI positive mode with a ramp trap collision energy of the high-energy function set at 60-120 eV; high-energy function (2: TOF MS ES$^+$)). XIC for 12-deoxy-16-hydroxyphorbol 20-acetate-16-benzoate-13-phenylacetate (AcDPPBz (**6**)) [M+Na]$^+$ molecular ion: (**a**) *m/z* 651.2565, calculated mass for $C_{37}H_{40}O_9Na$. XICs for selected characteristic ions of 16-hydroxy-12-deoxyphorbol 20-acetate-13,16-diacyl derivatives HRMS^E spectra (see Scheme 2 and Figure S22) at (**b**) *m/z* 529.2202, (**c**) *m/z* 411.1784, (**d**) *m/z* 393.1678, (**e**) *m/z* 333.1467 and *m/z* (**f**) 293.1542. (**g**) TIC of UHPLC-HRMS^E chromatographic profile of fraction G-6 from *E. resinifera* extract.

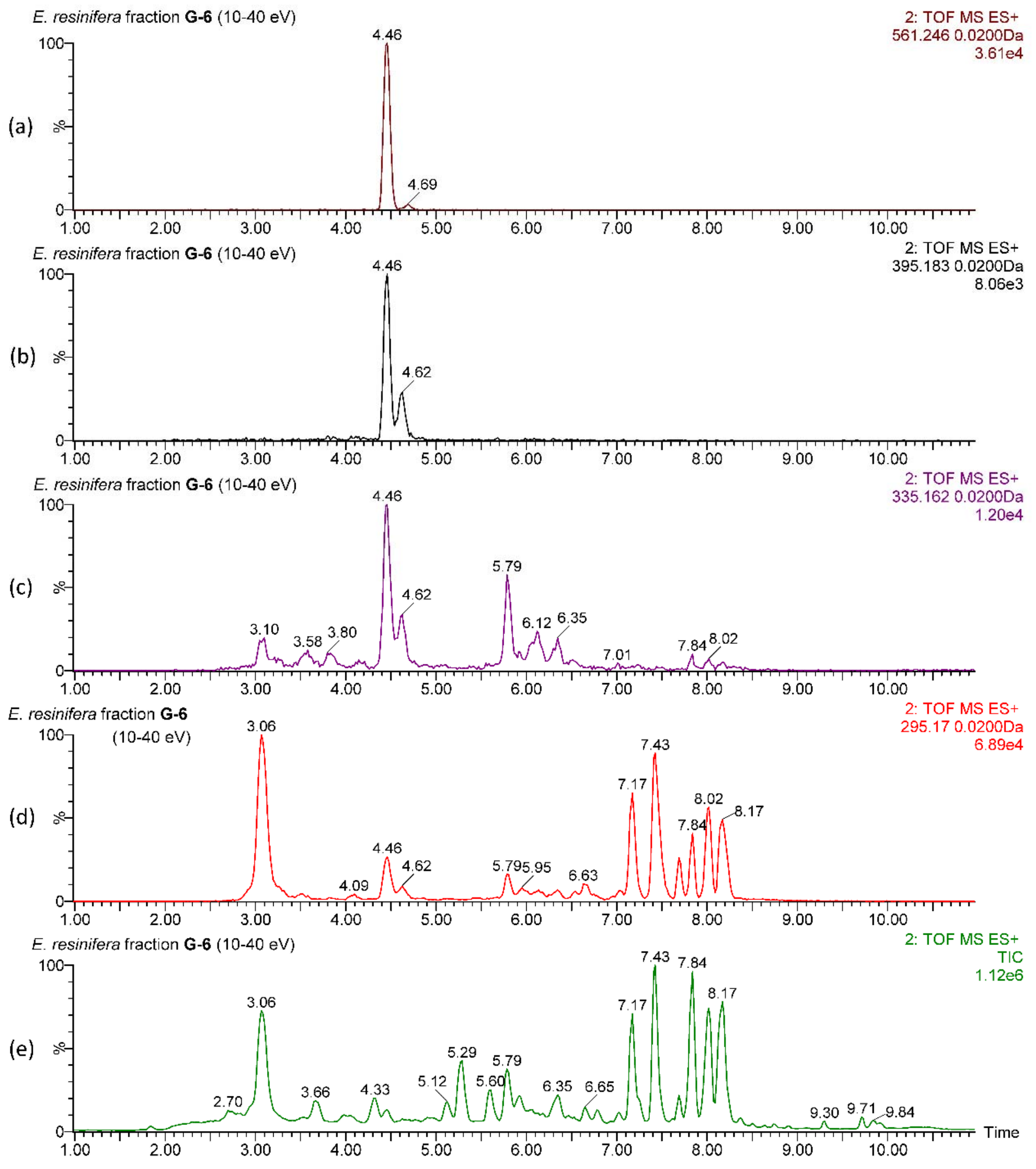

Figure S33. Total Ion Current (TIC) and eXtracted Ion Chromatograms (XICs), at selected *m/z* previously observed in high energy HRMS^E of 12-deoxyphorbol 20-acetate-13-acyl derivatives, obtained from UHPLC-HRMS^E experiments for chromatographic fraction G-6 of *E. resinifera* (data acquired in ESI positive mode with a ramp trap collision energy of the high-energy function set at 10-40 eV; high-energy function (2: TOF MS ES+)). XIC for 12-deoxyphorbol 20-acetate-13-(*p*-methoxyphenyl)acetate (AcDPMeOP (**14**)) [M+Na]$^+$ molecular ion: (**a**) *m/z* 561.2464 calculated mass for $C_{31}H_{38}O_8Na$. XICs for selected featured ions of 12-deoxyphorbol 20-acetate-13-acyl derivatives HRMS^E spectra (see Scheme 4 and Figure S29) at: (**b**) *m/z* 395.1834; (**c**) *m/z* 335.1623; (**d**) *m/z* 295.1698. (**e**) UHPLC-HRMS^E chromatographic profile (TIC) of fraction G-6 from *E. resinifera* extract.

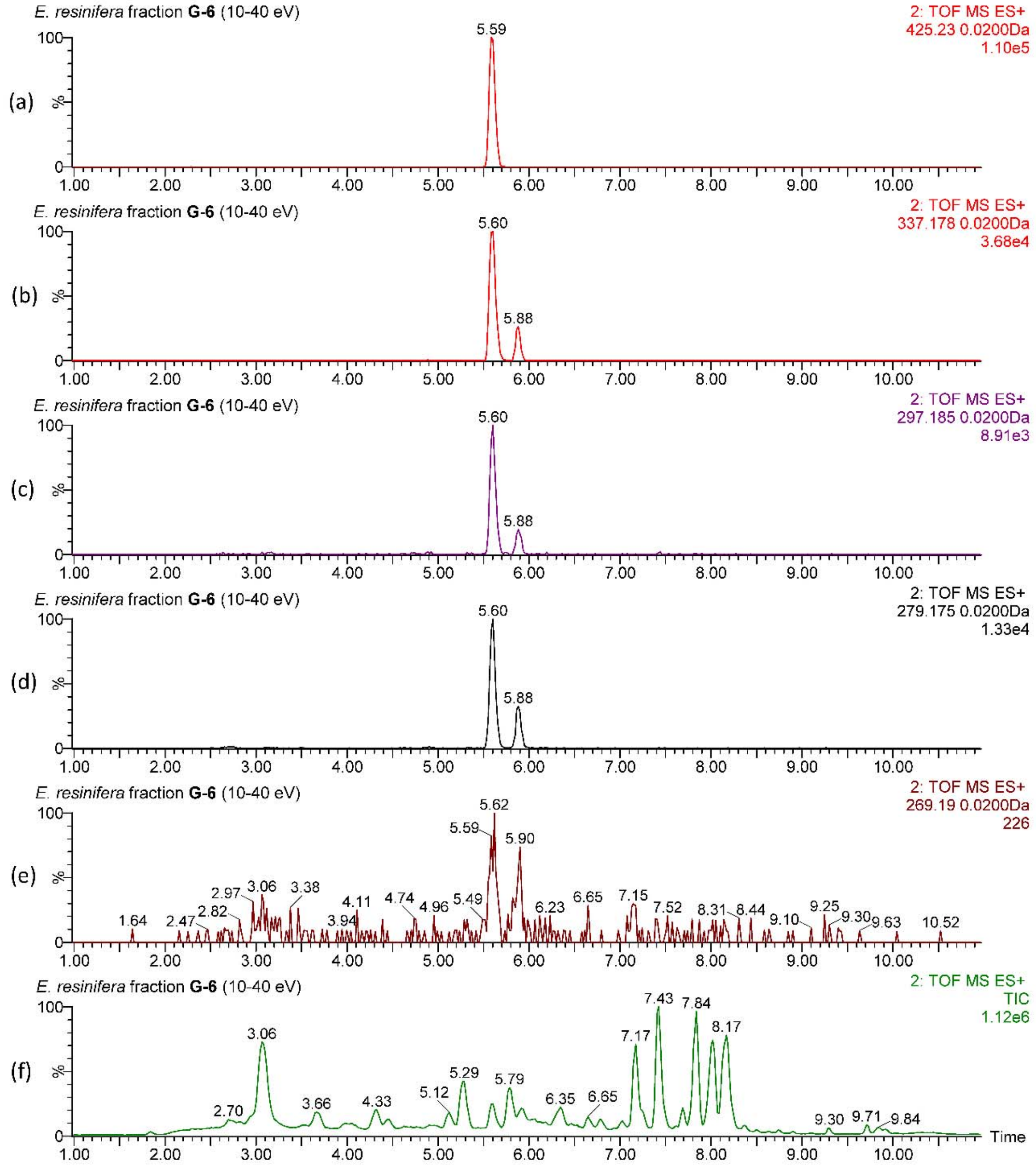

Figure S34. Total Ion Current (TIC) and eXtracted Ion Chromatograms (XICs), at selected *m/z* previously observed in high energy HRMS^E of 12,20-dideoxyphorbol 13-acyl derivatives, obtained from UHPLC-HRMS^E experiments for chromatographic fraction G-6 of *E. resinifera* (data acquired in ESI positive mode with a ramp trap collision energy of the high-energy function set at 10-40 eV; high-energy function (2: TOF MS ES+)). XIC for 12,20-dideoxyphorbol 13-isobutyrate (diDPB (**15**)) [M+Na]$^+$ molecular ion: (**a**) *m/z* 425.2304 calculated mass for $C_{24}H_{34}O_5Na$. XICs for selected featured ions of 12,20-dideoxyphorbol 13-acyl derivatives HRMS^E spectra (see Scheme 5 and Figure S30) at: (**b**) *m/z* 337.1780; (**c**) *m/z* 297.1855; (**d**) *m/z* 279.1749 and (**e**) *m/z* 269.1905. (**f**) UHPLC-HRMS^E chromatographic profile (TIC) of fraction G-6 from *E. resinifera* extract.

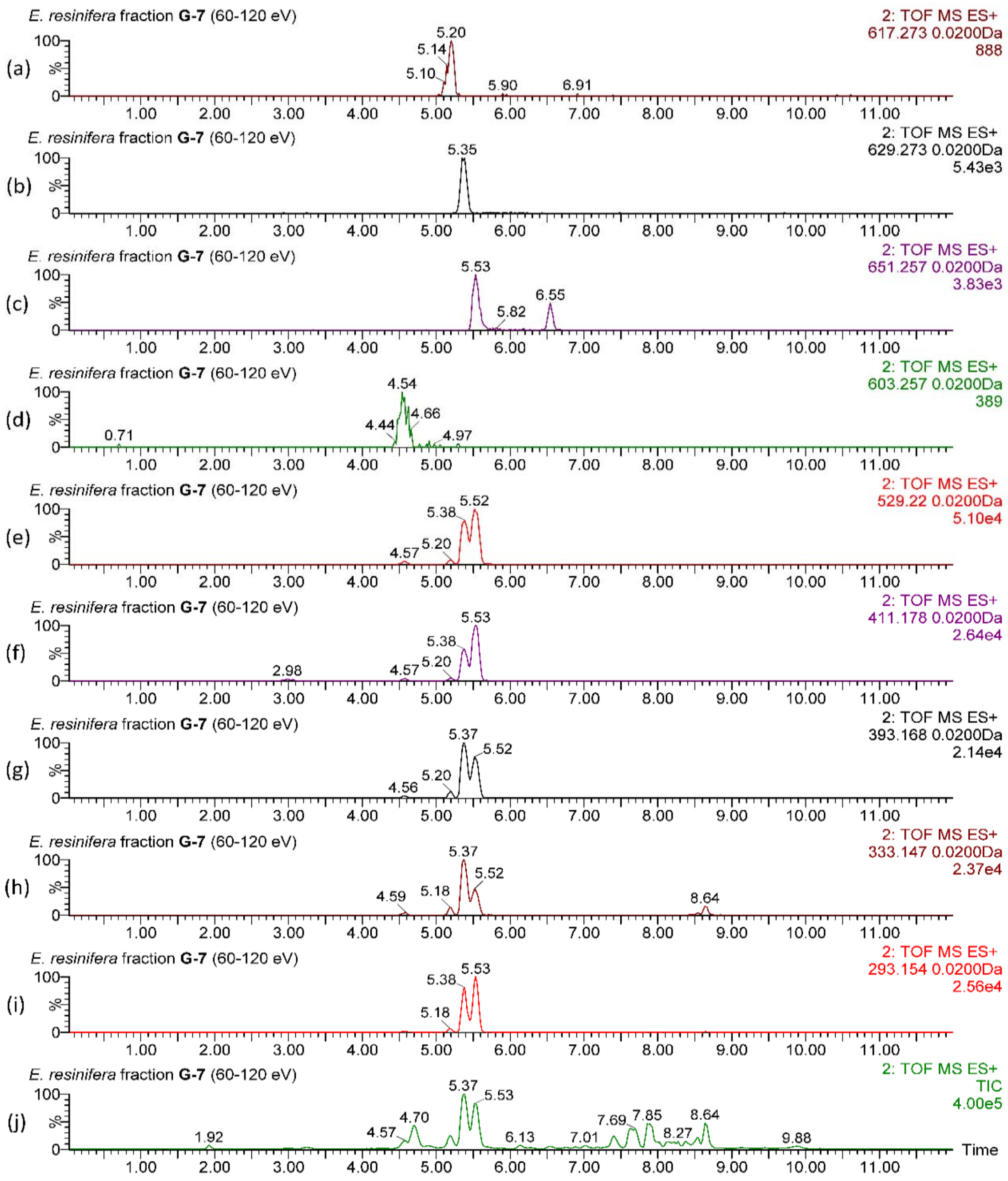

Figure S35. Total Ion Current (TIC) and eXtracted Ion Chromatograms (XICs), at selected m/z previously observed in high energy HRMS^E of 12-deoxy-16-hydroxyphorbol 20-acetate-13,16-diacyl derivatives, obtained from UHPLC-HRMS^E experiments for chromatographic fraction G-7 of *E. resinifera* (data acquired in ESI positive mode with a ramp trap collision energy of the high-energy function set at 60-120 eV; high-energy function (2: TOF MS ES$^+$)). XIC for 12-deoxy-16-hydroxyphorbol 20-acetate-13,16-diacyl derivatives [M+Na]$^+$ molecular ions: (**a**) *m/z* 617.2727, calculated mass for $C_{34}H_{42}O_9Na$ (AcDPPI (**4**)); (**b**) *m/z* 629.2727, calculated mass for $C_{35}H_{42}O_9Na$ (AcDPPT (**5**)); (**c**) *m/z* 651.2565, calculated mass for $C_{37}H_{40}O_9Na$ (AcDPPBz (**6**)); (**d**) *m/z* 603.2570, calculated mass for $C_{33}H_{40}O_9Na$ (AcDPPU$_2$ (**20**)). XICs for selected characteristic ions of 16-hydroxy-12-deoxyphorbol 20-acetate-13,16-diacyl derivatives HRMS^E spectra (see Scheme 2 and Figure S22) at (**e**) *m/z* 529.2202, (**f**) *m/z* 411.1784, (**g**) *m/z* 393.1678, (**h**) *m/z* 333.1467 and (**i**) *m/z* 293.1542. (**j**) TIC of UHPLC-HRMS^E chromatographic profile of fraction G-7 from *E. resinifera* extract.

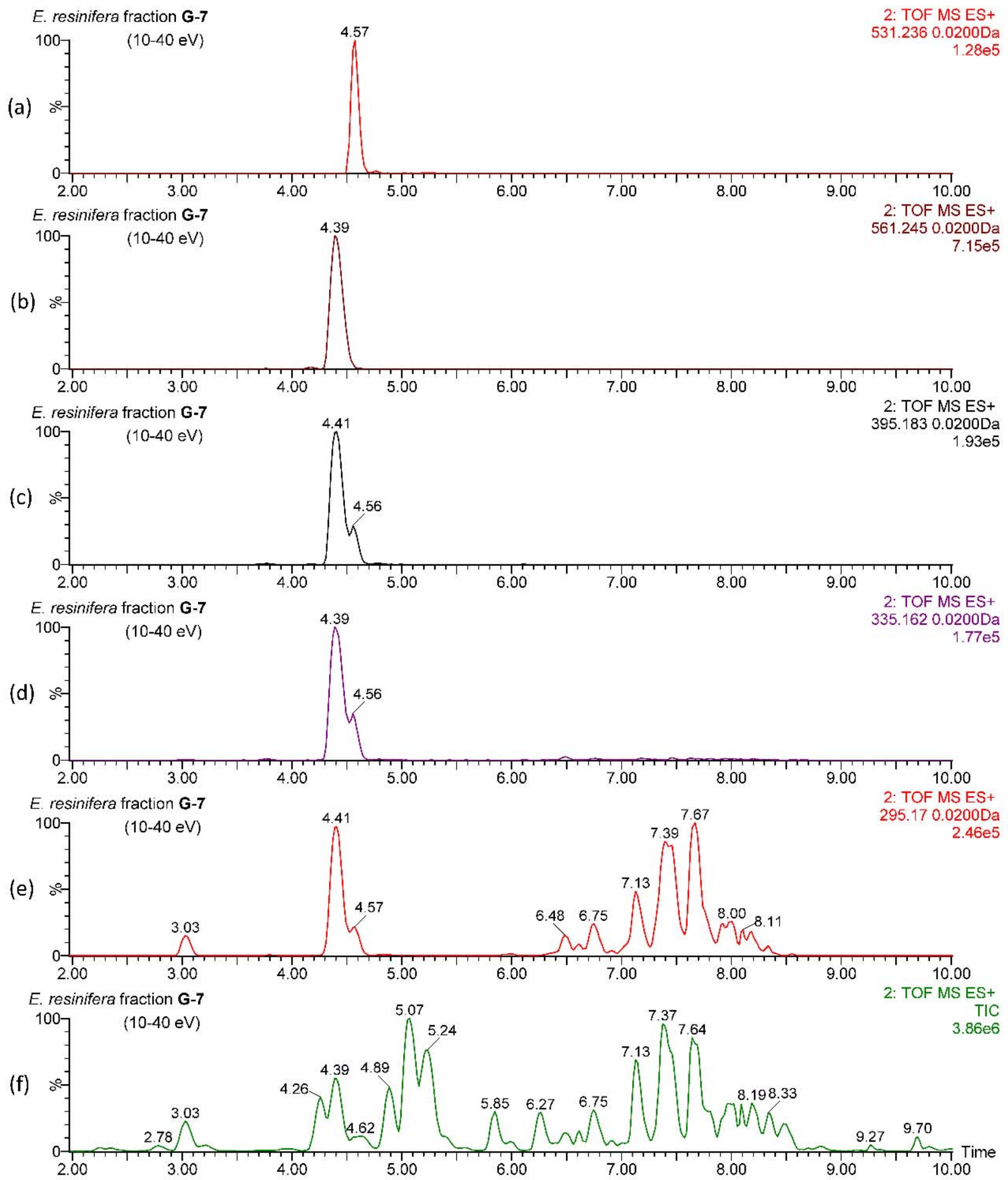

Figure S36. Total Ion Current (TIC) and eXtracted Ion Chromatograms (XICs), at selected *m/z* previously observed in high energy HRMS^E of 12-deoxyphorbol 20-acetate-13-acyl derivatives, obtained from UHPLC-HRMS^E experiments for chromatographic fraction G-7 of *E. resinifera* (data acquired in ESI positive mode with a ramp trap collision energy of the high-energy function set at 10-40 eV; high-energy function (2: TOF MS ES+)). XICs for 12-deoxyphorbol 20-acetate-13-acyl derivatives $[M+Na]^+$ molecular ions: (**a**) *m/z* 531.236, calculated mass for $C_{30}H_{36}O_7Na$ (AcDPP (**12**)); (**b**) *m/z* 561.2464 calculated mass for $C_{31}H_{38}O_8Na$ (AcDPMeOP (**14**)). XICs for selected featured ions of 12-deoxyphorbol 20-acetate-13-acyl derivatives HRMS^E spectra (see Scheme 4 and Figure S29) at: (**c**) *m/z* 395.1834; (**d**) *m/z* 335.1623; (**e**) *m/z* 295.1698. (**f**) UHPLC-HRMS^E chromatographic profile (TIC) of fraction G-7 from *E. resinifera* extract.

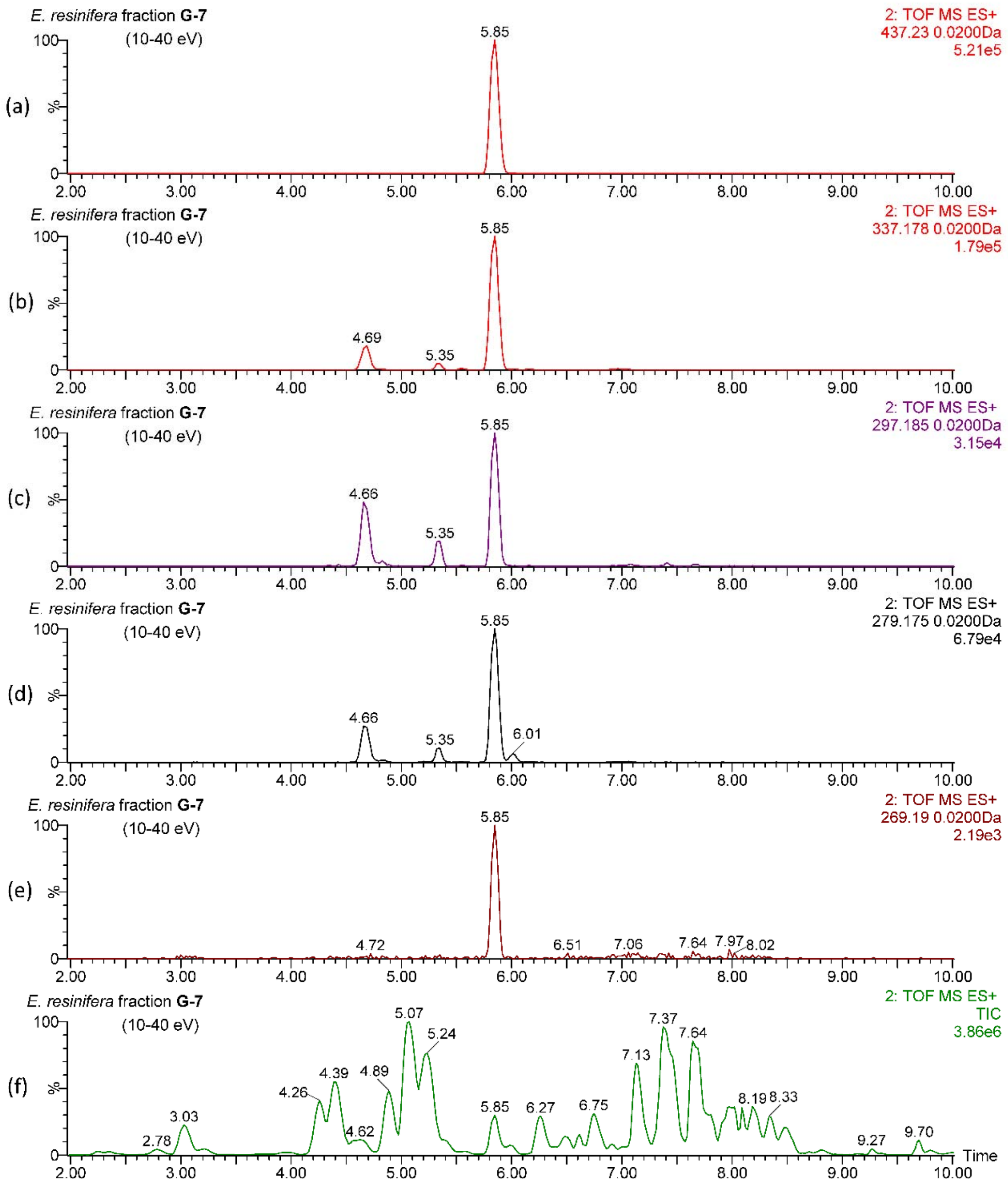

Figure S37. Total Ion Current (TIC) and eXtracted Ion Chromatograms (XICs), at selected *m/z* previously observed in high energy HRMS^E of 12,20-dideoxyphorbol 13-acyl derivatives, obtained from UHPLC-HRMS^E experiments for chromatographic fraction G-7 of *E. resinifera* (data acquired in ESI positive mode with a ramp trap collision energy of the high-energy function set at 10-40 eV; high-energy function (2: TOF MS ES+)). XIC for 12,20-dideoxyphorbol 13-acyl derivatives [M+Na]$^+$ molecular ion: (**a**) *m/z* 437.2304 calculated mass for $C_{25}H_{34}O_5Na$ (diDPU$_4$ (**22**)). XICs for selected featured ions of 12,20-dideoxyphorbol 13-acyl derivatives HRMS^E spectra (see Scheme 5 and Figure S30) at: (**b**) *m/z* 337.1780; (**c**) *m/z* 297.1855; (**d**) *m/z* 279.1749 and (**e**) *m/z* 269.1905. (**f**) UHPLC-HRMS^E chromatographic profile (TIC) of fraction G-7 from *E. resinifera* extract.

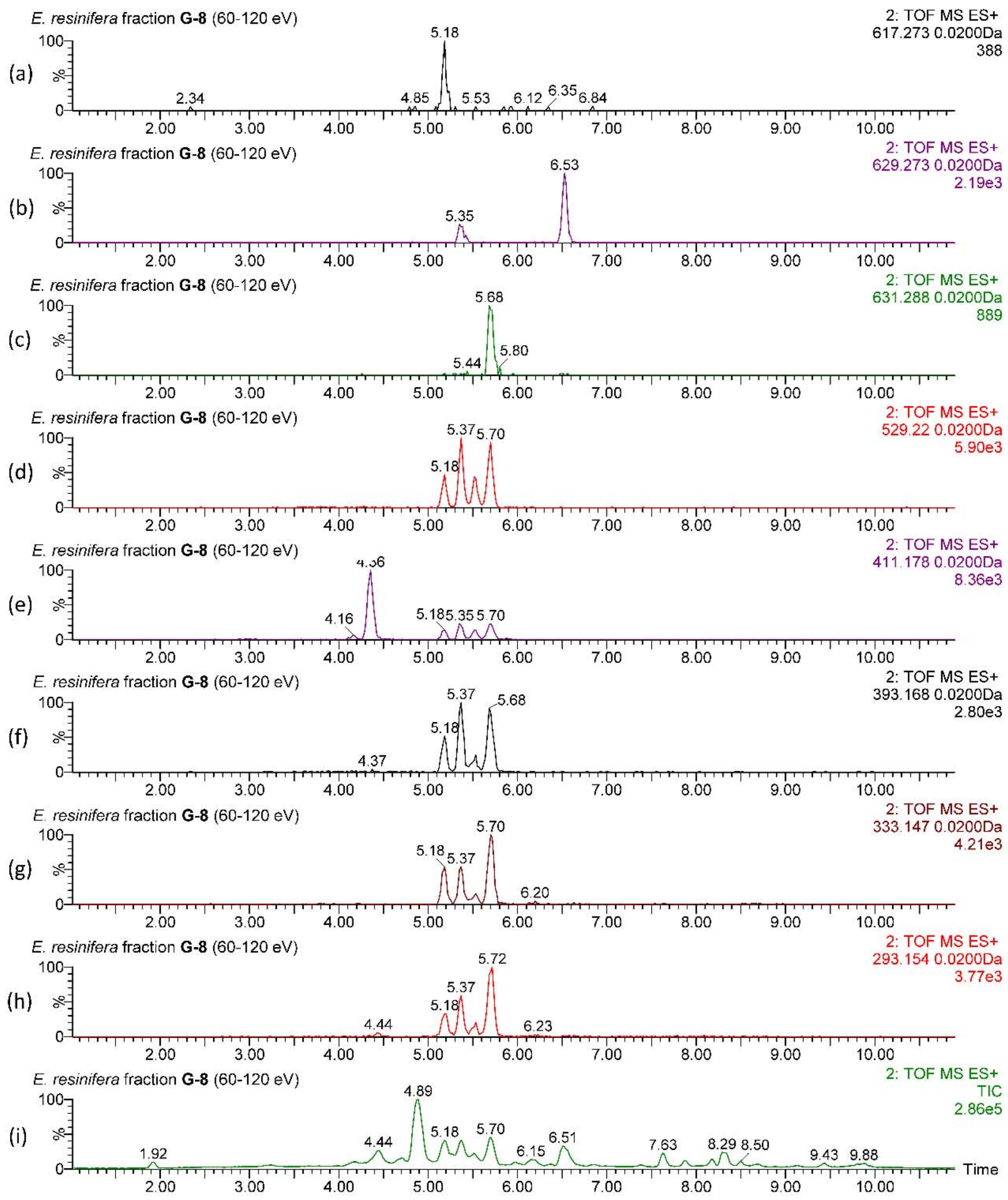

Figure S38. Total Ion Current (TIC) and eXtracted Ion Chromatograms (XICs), at selected m/z previously observed in high energy HRMS^E of 12-deoxy-16-hydroxyphorbol 20-acetate-13,16-diacyl derivatives, obtained from UHPLC-HRMS^E experiments for chromatographic fraction G-8 of *E. resinifera* (data acquired in ESI positive mode with a ramp trap collision energy of the high-energy function set at 60-120 eV; high-energy function (2: TOF MS ES$^+$)). XIC for 12-deoxy-16-hydroxyphorbol 20-acetate-13,16-diacyl derivatives [M+Na]$^+$ molecular ions: (**a**) *m/z* 617.2727, calculated mass for $C_{34}H_{42}O_9Na$ (AcDPPI (**4**)); (**b**) *m/z* 629.2727, calculated mass for $C_{35}H_{42}O_9Na$ (AcDPPT (**5**)); (**c**) *m/z* 631.2883, calculated mass for $C_{35}H_{44}O_9Na$ (AcDPPU$_3$ (**21**)). XICs for selected characteristic ions of 16-hydroxy-12-deoxyphorbol 20-acetate-13,16-diacyl derivatives HRMS^E spectra (see Scheme 2 and Figure S22) at (**d**) *m/z* 529.2202, (**e**) *m/z* 411.1784, (**f**) *m/z* 393.1678, (**g**) *m/z* 333.1467 and (**h**) *m/z* 293.1542. (**i**) TIC of UHPLC-HRMS^E chromatographic profile of fraction G-8 from *E. resinifera* extract.

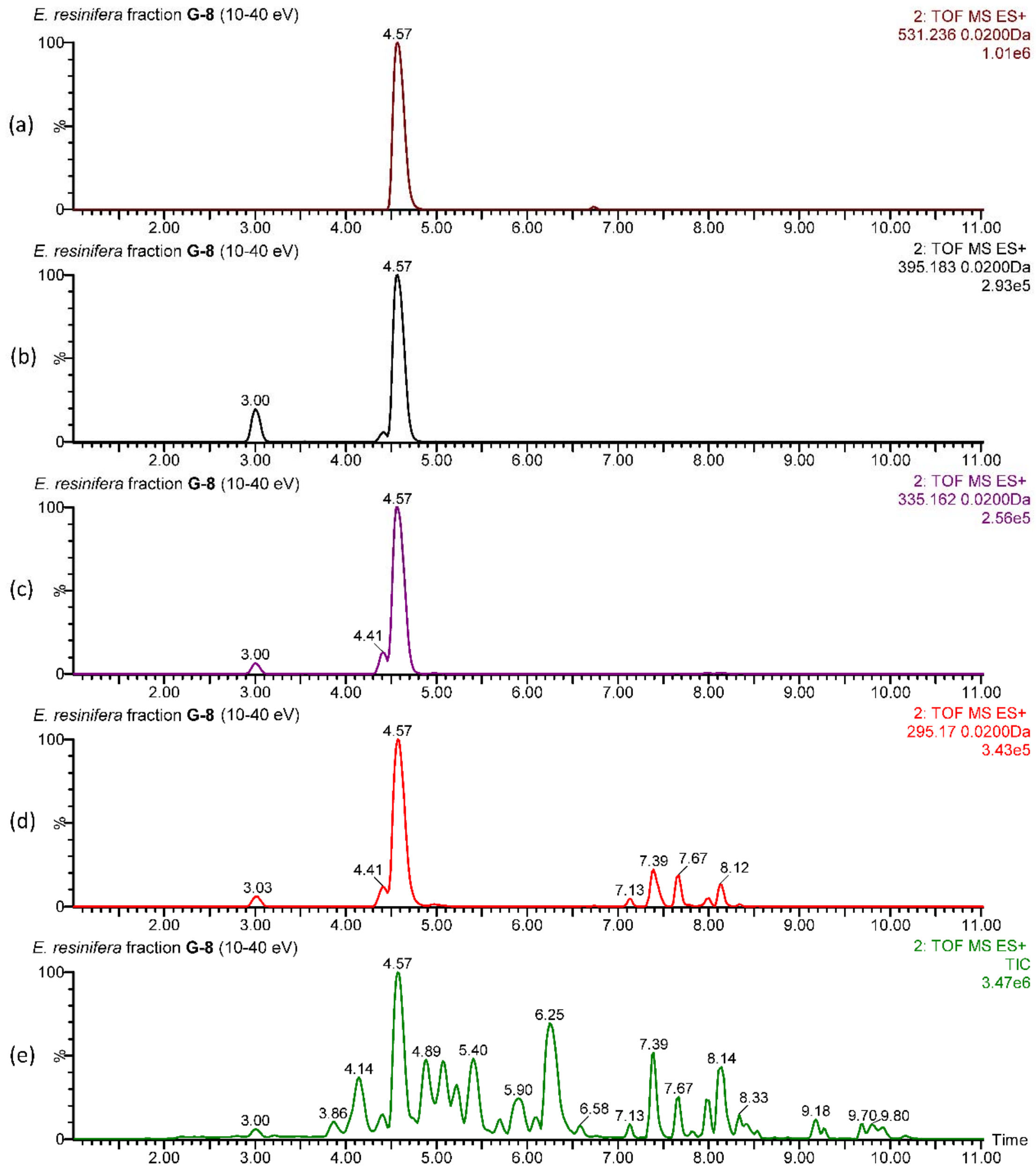

Figure S39. Total Ion Current (TIC) and eXtracted Ion Chromatograms (XICs), at selected *m/z* previously observed in high energy HRMS^E of 12-deoxyphorbol 20-acetate-13-acyl derivatives, obtained from UHPLC-HRMS^E experiments for chromatographic fraction G-8 of *E. resinifera* (data acquired in ESI positive mode with a ramp trap collision energy of the high-energy function set at 10-40 eV; high-energy function (2: TOF MS ES+)). XIC for 12-deoxyphorbol 20-acetate-13-phenylacetate (AcDPP (**12**)) $[M+Na]^+$ molecular ion: (**a**) *m/z* 531.2359 calculated mass for $C_{30}H_{36}O_7Na$. XICs for selected featured ions of 12-deoxyphorbol 20-acetate-13-acyl derivatives HRMS^E spectra (see Scheme 4 and Figure S29) at: (**b**) *m/z* 395.1834; (**c**) *m/z* 335.1623; (**d**) *m/z* 295.1698. (**e**) UHPLC-HRMS^E chromatographic profile (TIC) of fraction G-8 from *E. resinifera* extract.

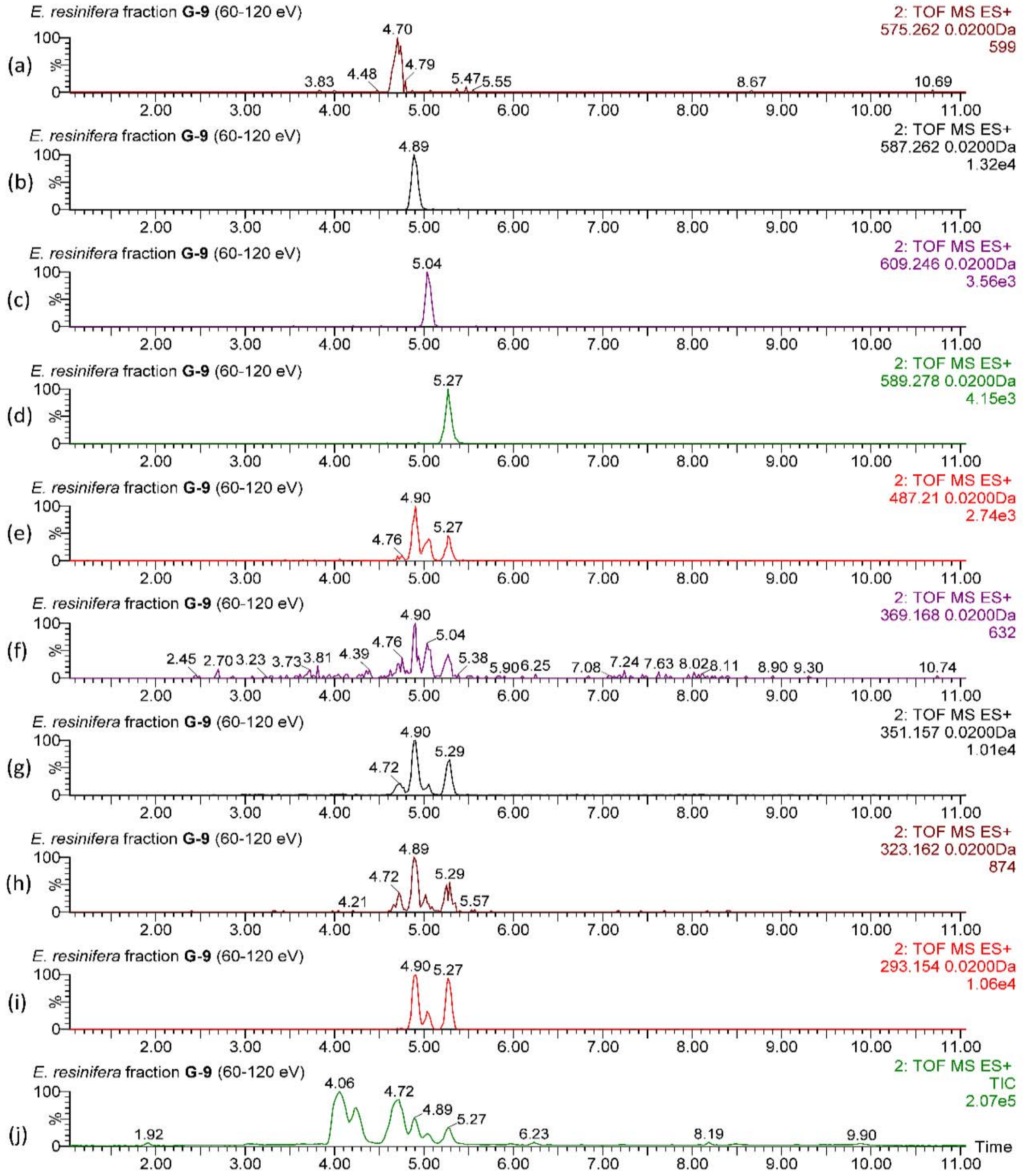

Figure S40. Total Ion Current (TIC) and eXtracted Ion Chromatograms (XICs), at selected m/z previously observed in high energy HRMS^E of 12-deoxy-16-hydroxyphorbol 13,16-diacyl derivatives, obtained from UHPLC-HRMS^E experiments for chromatographic fraction G-9 of *E. resinifera* (data acquired in ESI positive mode with a ramp trap collision energy of the high-energy function set at 60-120 eV; high-energy function (2: TOF MS ES$^+$)). XIC for 12-deoxy-16-hydroxyphorbol 13,16-diacyl derivatives [M+Na]$^+$ molecular ions: (**a**) *m/z* 575.2621, calculated mass for $C_{32}H_{40}O_8Na$ (DPPI (**1**)); (**b**) *m/z* 587.2621, calculated mass for $C_{33}H_{40}O_8Na$ (DPPT (**2**)); (**c**) *m/z* 609.2464, calculated mass for $C_{35}H_{38}O_8Na$ (DPPBz (**3**)); (**d**) *m/z* 589.2777, calculated mass for $C_{33}H_{42}O_8Na$ (DPPU$_1$ (**19**)). XICs for selected characteristic ions of 12-deoxy-16-hydroxyphorbol 13,16-diacyl derivatives HRMS^E spectra (see Scheme 1 and Figure S20) at (**e**) *m/z* 487.2097, (**f**) *m/z* 369.1678, (**g**) *m/z* 351.1572, (**h**) *m/z* 323.1623 and (**i**) *m/z* 293.1542. (**j**) TIC of UHPLC-HRMS^E chromatographic profile of fraction G-9 from *E. resinifera* extract.

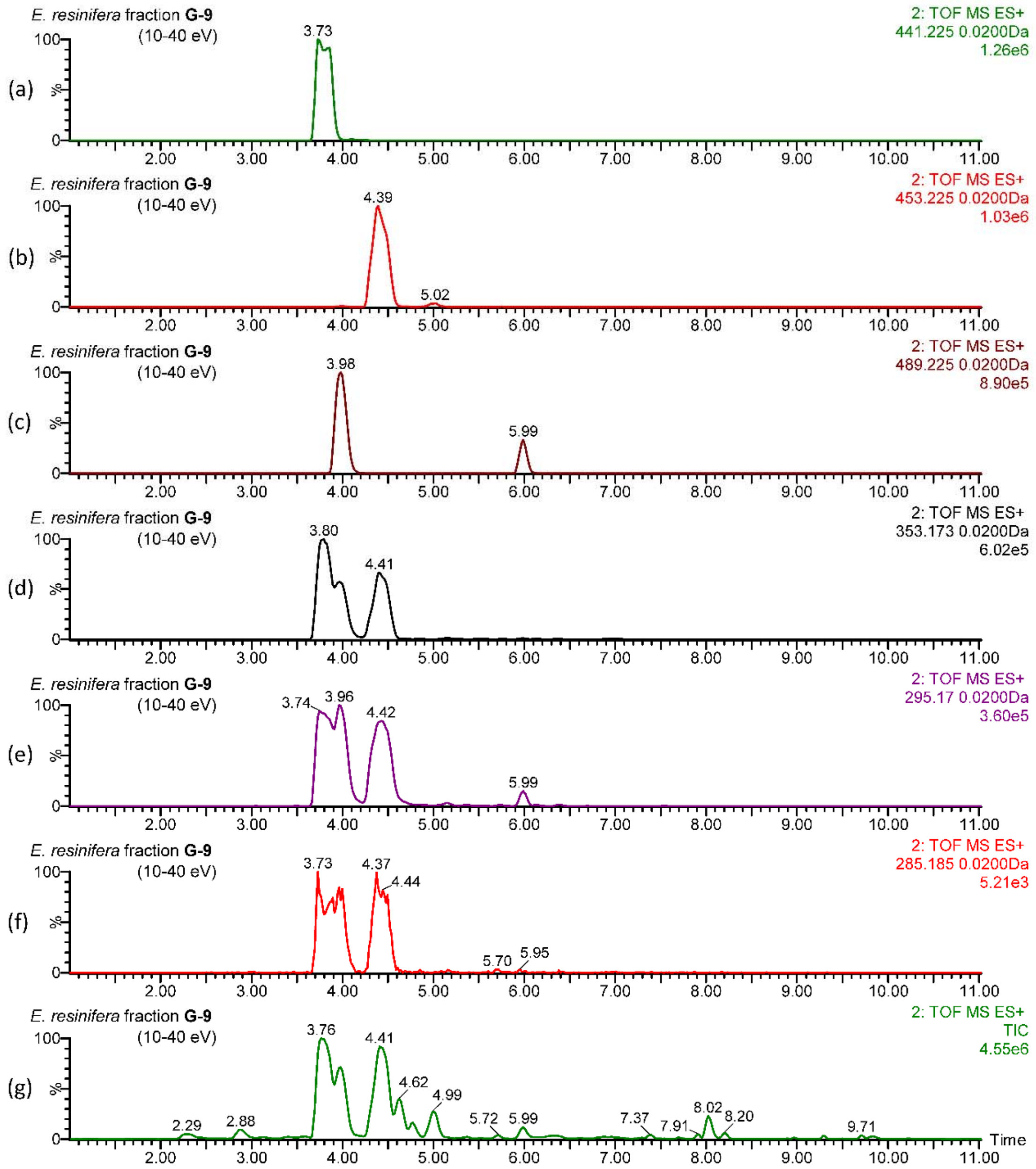

Figure S41. Total Ion Current (TIC) and eXtracted Ion Chromatograms (XICs), at selected *m/z* previously observed in high energy HRMS^E of 12-deoxyphorbol 13-acyl derivatives, obtained from UHPLC-HRMS^E experiments for chromatographic fraction G-9 of *E. resinifera* (data acquired in ESI positive mode with a ramp trap collision energy of the high-energy function set at 10-40 eV; high-energy function (2: TOF MS ES+)). XICs for 12-deoxyphorbol 13-acyl derivatives [M+Na]$^+$ molecular ions: (**a**) *m/z* 441.2253 calculated mass for $C_{24}H_{34}O_6Na$ (DPB (**7**)); (**b**) *m/z* 453.2253 calculated mass for $C_{25}H_{34}O_6Na$ (DPA (**8**)) and (**c**) *m/z* 489.2253, calculated mass for $C_{28}H_{34}O_6Na$ (DPP (**9**)). XICs for selected featured ions of 12-deoxyphorbol 13-acyl derivatives HRMS^E spectra (see Scheme 3 and Figure S25) at: (**d**) *m/z* 395.1834; (**e**) *m/z* 335.1623; (**f**) *m/z* 295.1698. (**g**) UHPLC-HRMS^E chromatographic profile (TIC) of fraction from *E. resinifera* extract.

4- UHPLC-HRMS^E data. Tables.

Table S1. Selected ions of high energy HRMS^E experiment (DIA) [1], of DPPI (**1**) and DPPT (**2**) (data acquired in ESI positive ionization with a ramp trap collision energy of the high-energy function set at 60-120 eV).

**Compound**	**Observed m/z (%)**	**Calculated m/z**	**Δm (mDa)**	**Elemental composition**	**Annotation**
DPPI (**1**)	575.2619 (18)	575.2621	-0.2	$C_{32}H_{40}O_8Na$	$[M+Na]^+$
	487.2097 (13)	487.2097	0.0	$C_{28}H_{32}O_6Na$	$[M-C_4H_8O_2+Na]^+$
	439.2099 (44)	439.2097	+0.2	$C_{24}H_{32}O_6Na$	$[M-C_8H_8O_2+Na]^+$
	369.1688 (3)	369.1678	+1.0	$C_{20}H_{26}O_5Na$	$[M-C_8H_8O_2-C_4H_6O+Na]^+$
	351.1575 (32)	351.1572	+0.3	$C_{20}H_{24}O_4Na$	$[M-C_8H_8O_2-C_4H_6O-H_2O+Na]^+$
	323.1627 (8)	323.1623	+0.4	$C_{19}H_{24}O_3Na$	$[M-C_8H_8O_2-C_4H_6O-H_2O-CO+Na]^+$
	311.1651 (12)	311.1647	+0.4	$C_{20}H_{23}O_3$	$[M-C_8H_8O_2-C_4H_6O-2H_2O+H]^+$
	293.1542 (100)	293.1542	0.0	$C_{20}H_{21}O_2$	$[M-C_8H_8O_2-C_4H_6O-3H_2O+H]^+$
	275.1434 (23)	275.1436	-0.2	$C_{20}H_{19}O$	$[M-C_8H_8O_2-C_4H_6O-4H_2O+H]^+$
	265.1588 (25)	265.1592	-0.4	$C_{19}H_{21}O$	$[M-C_8H_8O_2-C_4H_6O-3H_2O-CO+H]^+$
	247.1484 (23)	247.1487	-0.3	$C_{19}H_{19}$	$[M-C_8H_8O_2-C_4H_6O-4H_2O-CO+H]^+$
DPPT (**2**)	587.2639 (53)	587.2621	+1.8	$C_{33}H_{40}O_8Na$	$[M+Na]^+$
	487.2113 (9)	487.2097	+1.6	$C_{28}H_{32}O_6Na$	$[M-C_5H_8O_2+Na]^+$
	451.2114 (26)	451.2097	+1.7	$C_{25}H_{32}O_6Na$	$[M-C_8H_8O_2+Na]^+$
	369.1700 (1)	369.1678	+2.2	$C_{20}H_{26}O_5Na$	$[M-C_8H_8O_2-C_5H_6O+Na]^+$
	351.1584 (25)	351.1572	+1.2	$C_{20}H_{24}O_4Na$	$[M-C_8H_8O_2-C_5H_6O-H_2O+Na]^+$
	323.1636 (4)	323.1623	+1.3	$C_{19}H_{24}O_3Na$	$[M-C_8H_8O_2-C_5H_6O-H_2O-CO+Na]^+$
	311.1654 (8)	311.1647	+0.7	$C_{20}H_{23}O_3$	$[M-C_8H_8O_2-C_5H_6O-2H_2O+H]^+$
	293.1551 (100)	293.1542	+0.9	$C_{20}H_{21}O_2$	$[M-C_8H_8O_2-C_5H_6O-3H_2O+H]^+$
	275.1442 (21)	275.1436	+0.6	$C_{20}H_{19}O$	$[M-C_8H_8O_2-C_5H_6O-4H_2O+H]^+$
	265.1597 (19)	265.1592	+0.5	$C_{19}H_{21}O$	$[M-C_8H_8O_2-C_5H_6O-3H_2O-CO+H]^+$
	247.1493 (20)	247.1487	+0.6	$C_{19}H_{19}$	$[M-C_8H_8O_2-C_5H_6O-4H_2O-CO+H]^+$

Table S2. Selected ions of high energy HRMS^E experiment (DIA) [1], of DPB (**7**), DPA (**8**) and DPP (**9**) (data acquired in ESI positive ionization with a ramp trap collision energy of the high-energy function set at 10-40 eV).

Compound	Observed m/z (%)	Calculated m/z	Δm (mDa)	Elemental composition	Annotation
DPB (**7**)	441.2264 (100)	441.2253	+1.1	$C_{24}H_{34}O_6Na$	$[M+Na]^+$
	353.1737 (47)	353.1729	0.8	$C_{20}H_{26}O_4Na$	$[M-C_4H_8O_2+Na]^+$
	313.1807 (4)	313.1804	+0.3	$C_{20}H_{25}O_3$	$[M-C_4H_8O_2-H_2O+H]^+$
	295.1701 (25)	295.1698	+0.3	$C_{20}H_{23}O_2$	$[M-C_4H_8O_2-2H_2O+H]^+$
	285.1856 (0.3)	285.1855	+0.1	$C_{19}H_{25}O_2$	$[M-C_4H_8O_2-H_2O-CO+H]^+$
	277.1594 (7)	277.1592	+0.2	$C_{20}H_{21}O$	$[M-C_4H_8O_2-3H_2O+H]^+$
	267.1750 (7)	267.1749	+0.1	$C_{19}H_{23}O$	$[M-C_4H_8O_2-2H_2O-CO+H]^+$
	249.1644 (1)	249.1643	+0.1	$C_{19}H_{21}$	$[M-C_4H_8O_2-3H_2O-CO+H]^+$
DPA (**8**)	453.2273 (100)	453.2253	+2.0	$C_{25}H_{34}O_6Na$	$[M+Na]^+$
	353.1744 (39)	353.1729	+1.5	$C_{20}H_{26}O_4Na$	$[M-C_5H_8O_2+Na]^+$
	313.1816 (6)	313.1804	+1.2	$C_{20}H_{25}O_3$	$[M-C_5H_8O_2-H_2O+H]^+$
	295.1708 (24)	295.1698	+1.0	$C_{20}H_{23}O_2$	$[M-C_5H_8O_2-2H_2O+H]^+$
	285.1857 (0.4)	285.1855	+0.2	$C_{19}H_{25}O_2$	$[M-C_5H_8O_2-H_2O-CO+H]^+$
	277.1602 (5)	277.1592	+1.0	$C_{20}H_{21}O$	$[M-C_5H_8O_2-3H_2O+H]^+$
	267.1757 (6)	267.1749	+0.8	$C_{19}H_{23}O$	$[M-C_5H_8O_2-2H_2O-CO+H]^+$
	249.1646 (1)	249.1643	+0.3	$C_{19}H_{21}$	$[M-C_5H_8O_2-3H_2O-CO+H]^+$
DPP (**9**)	489.2280 (100)	489.2253	+2.7	$C_{28}H_{34}O_6Na$	$[M+Na]^+$
	353.1745 (34)	353.1729	+1.6	$C_{20}H_{26}O_4Na$	$[M-C_8H_8O_2+Na]^+$
	313.1818 (22)	313.1804	+1.4	$C_{20}H_{25}O_3$	$[M-C_8H_8O_2-H_2O+H]^+$
	295.1709 (37)	295.1698	+1.1	$C_{20}H_{23}O_2$	$[M-C_8H_8O_2-2H_2O+H]^+$
	285.1858 (0.5)	285.1855	+0.3	$C_{19}H_{25}O_2$	$[M-C_8H_8O_2-H_2O-CO+H]^+$
	277.1599 (4)	277.1592	+0.7	$C_{20}H_{21}O$	$[M-C_8H_8O_2-3H_2O+H]^+$
	267.1756 (6)	267.1749	+0.7	$C_{19}H_{23}O$	$[M-C_8H_8O_2-2H_2O-CO+H]^+$
	249.1657 (0.3)	249.1643	+1.4	$C_{19}H_{21}$	$[M-C_8H_8O_2-3H_2O-CO+H]^+$

Table S3. Selected ions of high energy HRMS^E experiment (DIA) [1], of AcDPB (**10**), AcDPA (**11**) and AcDPP (**12**) (data acquired in ESI positive ionization with a ramp trap collision energy of the high-energy function set at 10-40 eV).

Compound	Observed m/z (%)	Calculated m/z	Δm (mDa)	Elemental composition	Annotation
AcDPB (**10**)	483.2366 (100)	483.2359	+0.7	$C_{26}H_{36}O_7Na$	$[M+Na]^+$
	395.1841 (29)	395.1834	+0.7	$C_{22}H_{28}O_5Na$	$[M-C_4H_8O_2+Na]^+$
	355.1908 (0.1)	355.1909	-0.1	$C_{22}H_{27}O_4$	$[M-C_4H_8O_2-H_2O+H]^+$
	335.1620 (62)	335.1623	-0.3	$C_{20}H_{24}O_3Na$	$[M-C_4H_8O_2-AcOH+Na]^+$
	313.1810 (1)	313.1804	+0.6	$C_{20}H_{25}O_3$	$[M-C_4H_8O_2-H_2O-C_3H_2O+H]^+$
	295.1700 (28)	295.1698	+0.2	$C_{20}H_{23}O_2$	$[M-C_4H_8O_2-AcOH-H_2O+H]^+$
	277.1594 (9)	277.1592	+0.2	$C_{20}H_{21}O$	$[M-C_4H_8O_2-AcOH-2H_2O+H]^+$
	267.1750 (8)	267.1749	+0.1	$C_{19}H_{23}O$	$[M-C_4H_8O_2-AcOH-H_2O-CO+H]^+$
	249.1646 (1)	249.1643	+0.3	$C_{19}H_{21}$	$[M-C_4H_8O_2-AcOH-2H_2O-CO+H]^+$
AcDPA (**11**)	495.2363 (100)	495.2359	+0.4	$C_{27}H_{36}O_7Na$	$[M+Na]^+$
	395.1839 (28)	395.1834	+0.5	$C_{22}H_{28}O_5Na$	$[M-C_5H_8O_2+Na]^+$
	355.1911 (0.2)	355.1909	+0.2	$C_{22}H_{27}O_4$	$[M-C_5H_8O_2-H_2O+H]^+$
	335.1627 (51)	335.1623	+0.4	$C_{20}H_{24}O_3Na$	$[M-C_5H_8O_2-AcOH+Na]^+$
	313.1803 (1)	313.1804	-0.1	$C_{20}H_{25}O_3$	$[M-C_5H_8O_2-H_2O-C_3H_2O+H]^+$
	295.1699 (29)	295.1698	+0.1	$C_{20}H_{23}O_2$	$[M-C_5H_8O_2-AcOH-H_2O+H]^+$
	277.1592 (8)	277.1592	+0.0	$C_{20}H_{21}O$	$[M-C_5H_8O_2-AcOH-2H_2O+H]^+$
	267.1749 (8)	267.1749	+0.0	$C_{19}H_{23}O$	$[M-C_5H_8O_2-AcOH-H_2O-CO+H]^+$
	249.1646 (1)	249.1643	+0.3	$C_{19}H_{21}$	$[M-C_5H_8O_2-AcOH-2H_2O-CO+H]^+$
AcDPP (**12**)	531.2363 (100)	531.2359	+0.4	$C_{30}H_{36}O_7Na$	$[M+Na]^+$
	395.1840 (29)	395.1834	+0.6	$C_{22}H_{28}O_5Na$	$[M-C_8H_8O_2+Na]^+$
	355.1912 (0.6)	355.1909	+0.3	$C_{22}H_{27}O_4$	$[M-C_8H_8O_2-H_2O+H]^+$
	335.1627 (25)	335.1623	+0.4	$C_{20}H_{24}O_3Na$	$[M-C_8H_8O_2-AcOH+Na]^+$
	313.1809 (1)	313.1804	+0.5	$C_{20}H_{25}O_3$	$[M-C_8H_8O_2-H_2O-C_3H_2O+H]^+$
	295.1699 (29)	295.1698	+0.1	$C_{20}H_{23}O_2$	$[M-C_8H_8O_2-AcOH-H_2O+H]^+$
	277.1593 (7)	277.1592	+0.1	$C_{20}H_{21}O$	$[M-C_8H_8O_2-AcOH-2H_2O+H]^+$
	267.1750 (6)	267.1749	+0.1	$C_{19}H_{23}O$	$[M-C_8H_8O_2-AcOH-H_2O-CO+H]^+$
	249.1639 (1)	249.1643	-0.4	$C_{19}H_{21}$	$[M-C_8H_8O_2-AcOH-2H_2O-CO+H]^+$

References

1. Plumb, R.S.; Johnson, K.A.; Rainville, P.; Smith, B.W.; Wilson, I.D.; Castro-Perez, J.M.; Nicholson, J.K. UPLC/MSE; a new approach for generating molecular fragment information for biomarker structure elucidation. *Rapid Commun. Mass Spectrom.* **2006**, *20*, 1989–1994, doi:https://doi.org/10.1002/rcm.2550.
